# The effects of inversion polymorphisms on patterns of neutral genetic diversity

**DOI:** 10.1093/genetics/iyad116

**Published:** 2023-06-22

**Authors:** Brian Charlesworth

**Affiliations:** Institute of Ecology and Evolution, School of Biological Sciences, University of Edinburgh, Edinburgh EH9 3FL, UK

**Keywords:** inversion polymorphisms, genetic diversity, sequence divergence, recombination rate, population subdivision

## Abstract

The strong reduction in the frequency of recombination in heterozygotes for an inversion and a standard gene arrangement causes the arrangements to become partially isolated genetically, resulting in sequence divergence between them and changes in the levels of neutral variability at nucleotide sites within each arrangement class. Previous theoretical studies on the effects of inversions on neutral variability have assumed either that the population is panmictic or that it is divided into 2 populations subject to divergent selection. Here, the theory is extended to a model of an arbitrary number of demes connected by migration, using a finite island model with the inversion present at the same frequency in all demes. Recursion relations for mean pairwise coalescent times are used to obtain simple approximate expressions for diversity and divergence statistics for an inversion polymorphism at equilibrium under recombination and drift, and for the approach to equilibrium following the sweep of an inversion to a stable intermediate frequency. The effects of an inversion polymorphism on patterns of linkage disequilibrium are also examined. The reduction in effective recombination rate caused by population subdivision can have significant effects on these statistics. The theoretical results are discussed in relation to population genomic data on inversion polymorphisms, with an emphasis on *Drosophila melanogaster*. Methods are proposed for testing whether or not inversions are close to recombination–drift equilibrium, and for estimating the rate of recombinational exchange in heterozygotes for inversions; difficulties involved in estimating the ages of inversions are also discussed.

## Introduction

Naturally occurring genetic factors that massively reduce the rate of crossing over in *Drosophila melanogaster* when heterozygous were discovered by A.H. Sturtevant over 100 years ago (Sturtevant 1917), who later showed them to be inversions of segments of chromosomes (Sturtevant 1926). Inversion polymorphisms were long regarded as a curiosity of species of *Drosophila* and other “higher” Diptera, where they can readily be detected cytologically using the polytene chromosomes of the larval salivary glands. The largest body of information concerning the properties of inversions has been accumulated in studies of numerous *Drosophila* species (Krimbas and Powell 1992a; Kapun and Flatt 2019). The recent application of genome sequencing technology to the natural populations of many organisms, including humans, has revealed that inversions are much more abundant than what was previously thought. They are sometimes associated with striking phenotypic polymorphisms, such as the social chromosomes of ants and the behavioral and color polymorphisms of the ruff and white-throated sparrow (Wellenreuther and Bernatchez 2018; Villoutreix *et al*. 2021). This has led a surge of interest in the evolutionary significance of inversion polymorphisms; however, the *Drosophila* studies suggest that the vast majority of polymorphic inversions have little or no effects on visible phenotypes, although effects on quantitative traits such as body size and some fitness components have been detected (Krimbas and Powell 1992a; Kapun and Flatt 2019).

It is evident that the genetic isolation of different gene arrangements due to the suppression of crossing over in heterokaryotypes must play a major role in the processes that lead to the evolution of inversion polymorphisms, although the nature of these processes is still an open research question, which may well have multiple answers (Krimbas and Powell 1992b; Wellenreuther and Bernatchez 2018; Kapun and Flatt 2019; Villoutreix *et al*. 2021). The present paper is concerned with the consequences of the suppression of crossing over in heterozygotes for an inversion and a standard arrangement (heterokaryotypes) for the levels of diversity within and between the 2 arrangements at neutral nucleotide sites contained inside or close to the genomic region covered by the inversion. A critical parameter in determining these genomic features is the rate of recombinational exchange between arrangements in heterokaryotypes, the “gene flux” of Navarro *et al*. (1997). Evidence about this rate has been provided by genetic studies of recombination in heterokaryotypes. An important mechanism by which crossing over is suppressed or reduced in frequency during *Drosophila* female meiosis in heterozygotes for a paracentric inversion and the standard arrangement was proposed by Sturtevant and Beadle (1936), whose genetic data suggested that single crossovers produce dicentric and acentric chromosomes that fail to be included in the egg nucleus.

Recent studies of heterozygous *Drosophila* inversions suggest a near-total suppression of crossing over within the regions covered by a heterozygous inversion and for a substantial distance outside it (Koury 2023; Li *et al*. 2023). Recombination suppression is, however, often incomplete, with gene conversion and/or double crossing over causing exchanges of alleles between arrangements in heterokaryotypes (Sturtevant and Beadle 1936; Chovnick 1973; Krimbas and Powell 1992b; Navarro *et al*. 1997; Crown *et al*. 2018; Korunes and Noor 2019; Li *et al*. 2023). The conversion of double-strand breaks into noncrossover-associated gene conversion events apparently plays a major role in the suppression of crossing over, as well as allowing exchange to occur via gene conversion (Gong *et al*. 2005; Crown *et al*. 2018; Li *et al*. 2023). But the low rate of occurrence of such gene conversion events and double crossovers (a consensus value for *Drosophila* is about 10^–5^ per base pair in female meiosis: Korunes and Noor 2019) means that different arrangements are likely to become substantially genetically differentiated from each other, as a result of the interplay between mutation, genetic drift, and recombination (Ishii and Charlesworth 1977; Navarro *et al*. 1997, 2000; Andolfatto *et al*. 2001; Guerrero *et al*. 2012).

Several lines of evidence, including studies of changes in inversion frequencies in experimental populations, temporal fluctuations in inversion frequencies, and clinal patterns of inversion frequencies, as well as direct measurements of fitness components, show that many inversions are maintained at intermediate frequencies by natural selection (Krimbas and Powell 1992b; Kapun and Flatt 2019; Mérot *et al*. 2020). An initial complete loss of variability within haplotypes carrying a newly arisen, selectively favored inversion (the hitchhiking effect of Maynard Smith and Haigh 1974), will be eroded by the occurrence of new mutations that spread as a result of genetic drift and recombinational exchange with the standard arrangement. Once an inversion subject to balancing selection has established itself at an intermediate frequency within a population, there will be a gradual approach to mutation–drift–recombination equilibrium with respect to neutral or nearly neutral variants (Navarro *et al*. 2000). It is therefore important to consider the effects on variability of both the approach to equilibrium and the equilibrium situation.

The equilibrium properties of an inversion polymorphism with respect to neutral variability are similar to those for neutral loci linked to a single locus subject to balancing selection, first modeled by Ohta and Kimura (1970) using diffusion equations and by Strobeck (1983) using identity probabilities. Over the following 2 decades, subsequent modeling work using the structured coalescent process shed further light on patterns of neutral variability at sites linked to loci under balancing selection or divergent local selection, e.g. Kaplan et al. (1988), Hudson and Kaplan (1988), Hudson (1990), Takahata (1990), Charlesworth *et al*. (1997), Nordborg (1997), and Navarro *et al*. (2000), reviewed by Charlesworth *et al*. (2003). These studies showed that, when a balanced polymorphism has been maintained for a time that is much longer than the mean coalescent time for a neutral locus, we expect to see linkage disequilibrium (LD) between the target of selection and neutral sites closely linked to the target of selection, LD among these neutral sites, sequence divergence between haplotypes carrying the different alleles at the target of selection, and enhanced variability in the population as a whole around the target of selection.

These patterns are all reflections of the same process of divergence by drift and mutation among the partially isolated populations represented by the alleles at the target of selection, with a strong analogy with the outcome of mutation and drift in a geographically structured population (Hudson 1990; Charlesworth *et al*. 2003). Inversions are only special because of the strong suppression of recombination in inversion heterozygotes, which extends to regions outside the inversion that are close to the inversion breakpoints (Navarro *et al*. 1997; Crown *et al*. 2018; Korunes and Noor 2019; Koury 2023; Li *et al*. 2023). The patterns just described are thus much more likely to be detected with inversions than with single locus polymorphisms, due to the much larger region of the genome involved.

A basic finding of the early theoretical work was that significant associations between a balanced polymorphism and variants at a neutral locus at statistical equilibrium under drift and recombination are only likely to be observed if the rate of recombinational exchange in double heterozygotes for the loci involved is of the order of the reciprocal of the effective population size, *N_e_*, as is also the case for a pair of neutral loci (Hill and Robertson 1968; Ohta and Kimura 1969, 1971). Andolfatto *et al*. (2001) applied the theoretical results of Navarro *et al*. (2000) to the available data on *D. melanogaster* inversions and concluded that large effects of inversions on patterns of variability are likely to be seen only for loci close to inversion breakpoints, where exchange is probably most strongly inhibited, and that the observed patterns suggested that the inversions in question were of relatively recent origin compared with the age of the species.

Interest in the question of the effect of inversion polymorphisms on patterns of variability at sites that are either inside inversions or closely linked to inversion breakpoints has increased with the advent of whole genome sequencing, which has both greatly increased our ability to detect and characterize inversions and allows much more fine-scaled analyses of patterns of variability in genomic regions associated with inversions, e.g. Corbett-Detig and Hartl (2012), Cheng *et al.* (2012), Mérot *et al*. (2021), and Kapun *et al*. (2023). Recent population genomic analyses of several classic inversion polymorphisms in *D. melanogaster* suggest the following patterns (Corbett-Detig and Hartl 2012; Kapun *et al*. 2023):

1. There is a modest increase in overall nucleotide site diversity in the regions covered by inversions and adjacent to them, reflecting a low level of sequence divergence between arrangements relative to the genome-wide average diversity.
2. If genetic differentiation between inverted and standard arrangements relative to within-arrangement diversity is measured by *F_ST_*-like statistics, a much stronger effect is seen, with mean between-arrangement *F_ST_* values of the order of 0.1 to 0.2 in the interior of the inversion and in the regions adjacent to the inversions, with a sharp increase in regions close to the breakpoints.
3. For a low-frequency inversion such as *In(3R)P* in the Zambian population (Kapun *et al*. 2023), there is a lower than genome-wide average diversity at sites within the inversion and a higher than average diversity within the standard arrangement.

Much more information of this kind is likely to become available, and its interpretation requires a solid basis in population genetics theory. A number of theoretical investigations on the effect of balanced polymorphisms on variability at linked sites have extended the older work described above, without, however, greatly modifying the basic conclusions, e.g. Innan and Nordborg (2003), Guerrero *et al*. (2012), Rousset *et al*. (2014), and Zeng *et al*. (2021). A limitation of most of the theoretical work on the effects of selectively maintained polymorphisms on neutral diversity is that it assumes a single, randomly mating population, with the exception of Charlesworth *et al*. (1997), Nordborg (1997), Guerrero *et al*. (2012), and Rousset *et al*. (2014), who considered the case of divergent and/or balancing selection in a pair of populations. Nordborg and Innan (2003) examined a more general model of population structure but relied on coalescent process simulations to generate predictions.

While it is often considered that population subdivision in organisms like *Drosophila* is likely to have only minor effects on genetic diversity, given the generally low levels of *F_ST_* among populations (Singh and Rhomberg 1987; Schaeffer 2002; Lack *et al*. 2015, 2016), there is evidence from studies of allelism of recessive lethals that local deme sizes in *Drosophila* are somewhat restricted in size, with limited migration among them (Wright *et al*. 1942; Mukai and Yamaguchi 1974; Ives and Band 1986); this conclusion has recently been confirmed by a resequencing study of a single US population over time (Lange *et al*. 2022). It is known that population subdivision with a large number of demes increases the amount of LD among neutral loci when genomes are sampled from the same deme (Wakeley and Lessard 2003), because population subdivision increases local homozygosity, thereby reducing the effectiveness of recombination. This effect should also apply to associations between a locus under balancing selection and linked neutral sites. It is therefore important to examine the consequences of such subdivision for the effect of a diallelic balanced polymorphism on variability at a linked neutral site, and this is a major focus of the present paper.

For brevity, the locus under selection is referred here to as exhibiting an inversion polymorphism but the results apply to any Mendelian locus with 2 alleles maintained by balancing selection. An island model of a metapopulation of large size, divided into a finite number of demes of equal size, is assumed, with the same migration rate between all pairs of demes. As shown by Wakeley and Aliacar (2001), the properties of such a model are likely to provide a good approximation to more realistic scenarios, such as a 2-dimensional stepping stone model, provided that the number of demes is large. In order to obtain simple results for equilibrium populations, it is assumed that the inversion is the derived state and that selection on the inversion is sufficiently strong that it has risen quickly to an equilibrium frequency that is constant across demes. The properties of variability at the neutral locus, LD between the neutral locus and the inversion, and the extent of divergence between karyotypes at drift–mutation–recombination equilibrium are studied first, followed by an examination of the approach to equilibrium. Here, “karyotype” is used to denote the state of a haplotype with respect to the arrangement which it carries.

Recursion relations for mean pairwise coalescent times are used to obtain simple approximate expressions for the expected diversity and divergence statistics relevant to an inversion polymorphism at equilibrium under recombination and drift, and for the approach to their equilibrium values following the sweep of an inversion to a stable intermediate frequency. The effects of an inversion polymorphism on patterns of LD are also examined. The reduction in effective recombination rate caused by population subdivision can have significant effects on these statistics, and hence on estimates of the ages of inversions. Methods are proposed for testing whether or not inversions are close to recombination–drift equilibrium, and for estimating the rate of recombinational exchange in heterozygotes for inversions; a new method for determining the variances of pairwise coalescence times is also described. It is concluded that many of the observed patterns of diversity at putatively neutral sites associated with inversion polymorphisms in *D. melanogaster* are consistent with their being close to mutation–recombination–drift equilibrium.

## The model and its analysis

Assume that an autosomal inversion (*In*) is maintained at a frequency *x* and the standard arrangement (*St*) has frequency *y* = 1 − *x.* Without loss of generality, we can assume *x*$\leq \frac{1}{2}$ when considering equilibrium results; if this is not the case, then, *In* and *St* can simply be interchanged. Parameters for *In* and *St* are denoted by subscripts 1 and 2, respectively. The population is assumed to be divided into local populations (demes) that are at equilibrium under mutation, genetic drift and migration at loci independent of the inversion. A Wright–Fisher model of reproduction is assumed, so that the effective population size of a deme is equal to its adult population size. An island model with a large number of demes, *d*, each with population size *N* is assumed, so that the migration effective population size (Nagylaki 1998) is *N_T_* = *Nd.* Migration between populations occurs at rate *m* per generation.

The level of equilibrium neutral differentiation between demes at autosomal loci that are independent of the inversion is measured by *F_ST_* (Wright 1951), which is defined here as 1 minus the ratio of the mean coalescent time for pairs of alleles sampled from within a population to the mean coalescent time for pairs of alleles sampled randomly from the population as whole. With a large number of demes, $F_{ST}\approx 1/(1+4Nm)$ (Charlesworth 1998).

Random mating within local populations and with respect to arrangement status is assumed. The migration and recombination parameters, *m* and *r*, are assumed to be so small that second-order terms can be neglected. At a given neutral site within the region covered by the arrangement (or just outside it), recombinational exchange between arrangements caused by gene conversion and/or double crossing over occurs at rate *r* per generation. In the gametes produced by a heterokaryotype, *In*/*St*, there is a probability *r* that a given neutral site associated with the *In* haplotype came from the *St* haplotype and that the homologous site associated with the *St* haplotype came from the *In* haplotype. It is likely that the value of *r* will depend on the location of the site within the inversion, with the largest values for sites within the inversion that are remote from the breakpoints, due to the effects of the breakpoints in disturbing synapsis (Navarro *et al*. 1997), although the extent to which gene conversion events are influenced by proximity to the breakpoints is uncertain (Li *et al*. 2023). Sites outside the inversion will experience increasingly high rates of recombination with distance from the breakpoints as the effect of crossover suppression dies out (Koury 2023).

Inversion-carrying and standard haplotypes are denoted by indices 1 and 2, respectively. We need to distinguish between a pair of haplotypes that are sampled from the same deme (denoted by subscript *w*), and a pair of haplotypes sampled from 2 different demes (denoted by subscript *b*). The expected coalescent time for a sample of class *i*/*j* for a within-deme sample is denoted by *t_ijw_*, and the equivalent for a between-deme sample is *t_ijb_*. For a between-deme sample, the probability that a migrant haplotype came from the same deme as the nonmigrant haplotype is 1/(*d −* 1) and the probability that it came from a different deme is (*d −* 2)/(*d −* 1). Simple recursions for the *t*'s can be obtained, which are given by Equation (A1) of the Appendix. Further simplification is provided by neglecting the products of 1*/Nx* and 1/*Ny* with *m* and *r* (Equation A2).

It is convenient to scale the migration rate and recombination rate by 4 times the deme size, writing *M* = 4*Nm* and *R* = 4*Nr*. In addition, coalescent times can be expressed relative to the expected coalescent time for a pair of alleles sampled from the same deme at a locus that is independent of the inversion, *T_Sn_* = 2*N_T_* = 2*dN*. Upper case *T*'s are used to denote *t*'s divided by *T_Sn_*. As shown in the Appendix, manipulation of the resulting equations leads to simple explicit approximate expressions for the equilibrium values of the *T_ijb_* and *T_ijw_*, assuming a large number of demes and *M* >> *R* (Equations A5 and A6).

It is useful to consider the mean scaled coalescent time for pairs of alleles sampled randomly across karyotypes. For alleles from different demes, this is given by


(1a) $$T_{Tb}=2xyT_{12b}+x^{2}T_{11b}+y^{2}T_{22b}$$

Following Charlesworth *et al*. (1997), the mean scaled mean coalescent time for a pair of alleles sampled within karyotypes, but from different demes, is


(1b) $$T_{Sb}=xT_{11b}+yT_{22b}$$

For measuring differentiation between sequences associated with alleles maintained by selection, a between-karyotype analogue of *F_ST_* was defined by Charlesworth *et al*. (1997), which is analogous to the *K_ST_* measure of Hudson, Boos *et al*. (1992):


(2a) $$F_{ATb}=1\;\text{–}\frac{T_{Sb}}{T_{Tb}}$$

A related quantity, analogous to the < *F_ST_* > statistic of Hudson, Slatkin *et al*. (1992), is


(2b) $${\tilde{F}}_{ATb}=1\;\text{–}\frac{T_{Sb}}{T_{12b}}$$

For equilibrium under recombination and drift, for which *T*_12_ > 1, ${\tilde{F}}_{ATb}$ > *F_ATb_*, since *T*_12*b*_ > *T_Tb_*.

It should be borne in mind that different authors use different estimators for *F_ST_* or *F_AT_*; the widely used methods of Weir and Cockerham (1984) and Hudson, Slatkin *et al*. (1992), which are mathematically equivalent, have much larger expected values than *K_ST_* when the number of populations being compared is small (Charlesworth 1998; Gammerdinger *et al*. 2020), so that caution needs to be used when comparing *F_ST_* or *F_AT_* estimates from different studies.

Corresponding expressions apply to alleles sampled within demes, replacing subscript *b* by *w*. The Maruyama invariance principle for structured coalescent processes with conservative migration (Maruyama 1974; Nagylaki 1982) implies that at equilibrium, we have


(3) $$T_{Sw}=xT_{11w}+yT_{22w}=1$$

This result breaks down when *N_T_r* is close to zero; with *r* = 0, the 2 karyotypes behave as separate populations, with migration effective population sizes of *N_T_x* and *N_T_y*, respectively, so that *T_Sw_* = *x*^2^ + *y*^2^.

Equation (3) implies that the equilibrium expressions for *F_ATw_* and ${\tilde{F}}_{ATw\;}$ simplify to


(4a) $$F_{ATw}=1\;\text{–}\frac{1}{T_{Tw}}$$

(4b) $${\tilde{F}}_{ATw}\;=1\;\text{–}\frac{1}{T_{12w}}$$

For equilibrium populations, therefore, the 2 *F*-statistics for within-population/between-karyotype differentiation contain no more information than do *T_Tw_* and *T*_12*w*_, respectively; we have *T_Tw_* = 1/(1 − *F_ATw_*) and *T*_12*w*_ = 1/(1 − ${\tilde{F}}_{ATw}$). For applications of these formulae to population genomic data, the divergence statistics corresponding to the *T*'s need to be scaled by an estimate of mean neutral nucleotide site diversity at sites independent of the inversion, or by the weighted mean of the 2 within-karyotype diversities, as described in the *Discussion, Interpreting population genomic data on inversion polymorphisms*. The latter has the advantage that potential differences in mutation rates among different genomic regions are eliminated.

When the scaled migration rate *M* tends to infinity and *F_ST_* tends to zero, the case of a panmictic population with population size *N_T_* is approached and the subscripts *w* and *b* can be dropped. The recursion relations for the within-deme statistics can be used for this case (Equation A2), setting *m* to zero and the deme size to *N_T_*. Application of the above method to this case, writing *ρ* = 4*N_T_r* for the scaled recombination rate, and assuming that *ρ* > 0, gives the following expressions for equilibrium:


(5a) $$\;T_{12}=\;1+2\rho^{-1}$$

(5b) $$T_{11}\;=[\;x(1+2y)+\rho xy]/(1+\rho xy)$$

(5c) $$T_{22}\;=[\;y(1+2x)+\rho xy]/(1+\rho xy)$$

These expressions are equivalent to Equations (A3) and (A4) of Nordborg (1997), Equation (6) of Navarro *et al*. (2000), and Equation (8) of Zeng *et al*. (2021), which were derived by more complex methods. Consistent with Equation (3), *T_S_* = *xT*_11_ + *yT*_22_ = 1 at equilibrium, so that


(5d) $$\;T_{T}\;=1+4xy\rho^{-1}+xy(x-y{)}^{2}(1+\rho xy{)}^{-1}$$

(5e) $$F_{AT}=1-\frac{1}{1+4xy\rho^{-1}+xy{\left(x-y\right)}^{2}{\left(1+\rho xy\right)}^{-1}}$$

(5f) $${\tilde{F}}_{AT}=\frac{2}{2+\rho }$$

## Theoretical results: equilibrium populations

### General considerations

Navarro *et al*. (2000) have described results for the case of an equilibrium panmictic population, which is the limiting case when *F_ST_* = 0. The focus here is therefore on subdivided populations, using the approximations for the equilibrium *T*'s given by Equations (A5) and (A6), which assume large *d* and *m* >> *r*. When comparing the theoretical results with observations, it is useful to note that the *T*'s under the infinite sites model of Kimura (1971) are proportional to the corresponding mean pairwise diversities or divergences per nucleotide site, taken over large numbers of neutral sites with the same mutation and recombination rates (e.g. Hudson 1990). The mean levels of nucleotide site diversity and divergence between arrangements within *D. melanogaster* populations are such that the infinite sites model fits the data reasonably well (Corbett-Detig and Hartl 2012; Langley *et al*. 2012; Kapun *et al*. 2023), so that the *T* values and their ratios presented in the figures below indicate the corresponding expected diversity and divergence values when interpreting data on inversion polymorphisms. Most multicellular organisms have similar or lower diversity values (Buffalo 2021), and so should present even less of a problem of interpretation.

### Numerical results for subdivided populations

The results presented here are intended to represent a set of populations in a single geographical region that is isolated from other regions, with a within-deme neutral nucleotide site diversity value (*π*) similar to that found in population genomic surveys. The results from the *Drosophila* Genome Nexus Project, which has assembled genome sequence data for a large number of *D. melanogaster* genomes from natural populations (Lack *et al*. 2015, 2016), show that *F_ST_* between pairs of populations within a region is generally low, around 0.05 or even less, and is only about 0.2 between continents (Fig. 3 of Lack *et al*. 2016). *F_ST_* values in animals (Roux *et al*. 2016) and outcrossing flowering plants (Charlesworth 2003) rarely exceed 0.25, so that *F_ST_* in the figures was restricted to the range 0–0.25 for purposes of illustration, consistent with the approximations used to generate the results.

In what follows, special attention is paid to the results with *F_ST_* = 0.05, although the mean pairwise *F_ST_* for inversion-free genomes between the Zambian *D. melanogaster* population (ZI) and 2 populations from South Africa (SP and SI) is only 0.007 (Lack *et al*. 2016, Fig. 3). This suggests that panmixia is a good approximation for populations in this region, which is thought be close to the center of origin of the species (Sprengelmeyer *et al*. 2020). The ZI population was a focus of the intensive study of the *In(3R)P* inversion by Kapun *et al*. (2023), discussed below in relation to the theoretical results. It is important to note that, under the assumptions made here, the equations for the *T*'s involve only the parameters *M* = 4*Nm* and *ρ* = 4*N_T_r* = 4*Ndr*; in turn, with large *d*, we have *M* ≈ (1 − *F_ST_*)/*F_ST_*, where *F_ST_* corresponds to the *K_ST_* measure of differentiation among local populations for neutral loci independent of the inversion. Under these conditions, if *F_ST_*, *r*, and *N_T_* are specified, the results are not affected by the number of demes, the deme size (*N*), or the migration rate (*m*).

**Fig. 1.**
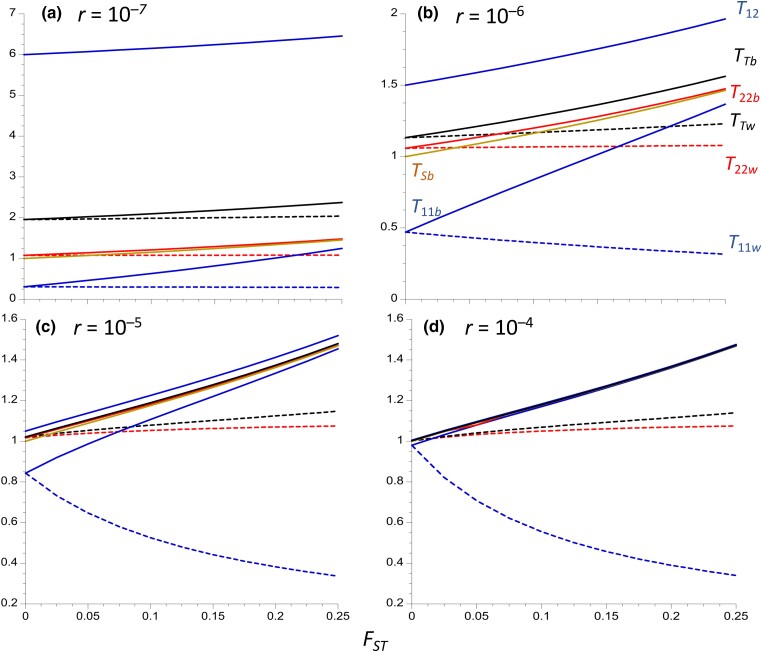
Equilibrium expected coalescence times (relative to 2*N_T_*) for an inversion polymorphism where the inversion is maintained at a constant frequency of 0.1. Four different recombination rates in heterokaryotypes (*r*) are modeled, as indicated at the top left of each panel. An island model of population structure is assumed, with 200 demes and a total population size of *N_T_* = 10^6^, so that individual demes have a population size of *N* = 5,000. The *X* axis is the equilibrium *F_ST_* for neutral sites unlinked to the inversion. Subscripts 1 and 2 denote alleles sampled from the inversion and standard arrangement, respectively; subscripts *w* and *b* denote alleles sampled from the same and from separate demes, respectively; subscript *T* denotes pairs of alleles sampled without regard to karyotype. The dashed curves represent within-deme coalescent times, and the solid curves are between-deme coalescent times; blue is *T*_12_ and *T*_11_, black is *T_T_*, blue is *T*_11_, and red is *T*_22_ (for *T*_12_, there is no significant difference between with- and between-deme values.) The mean within-karyotype values for between-deme samples (*T_Sb_*) is the solid beige curve; the within-deme equivalent (*T_Sw_*) is equal to 1 for all *r* and *F_ST_* values and is not displayed.

**Fig. 2.**
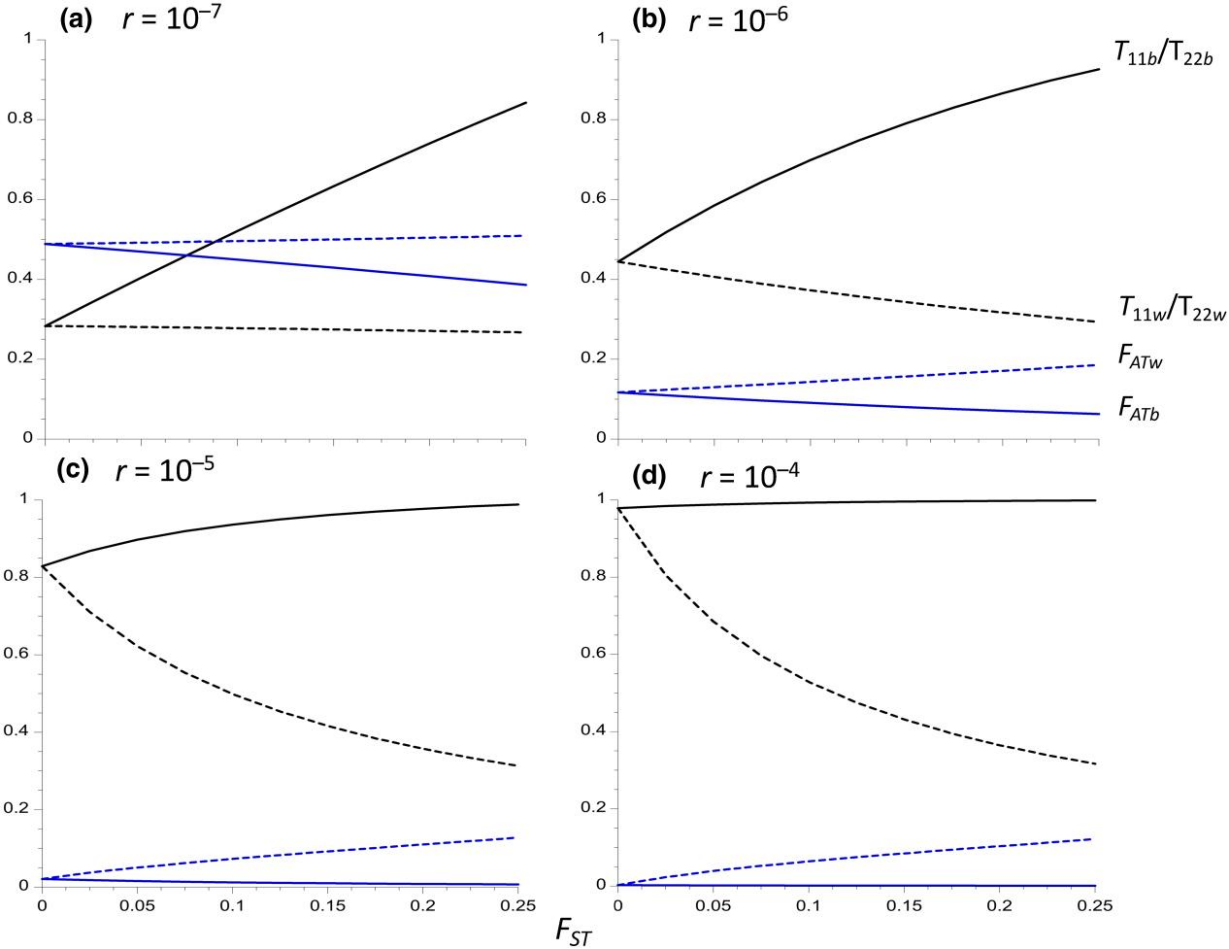
Equilibrium values of *F_ATw_* (blue dashed curves), *F_ATb_* (blue solid curves), *T*_11*w*_/*T*_22*w*_ (black dashed curves), and *T*_11*b*_/*T*_22*b*_ (black solid curves), for an inversion polymorphism where the inversion is maintained at a constant frequency of 0.1. The population and recombination parameters in Fig. 1 are used.

Results are displayed for 4 different values of the rate of recombination (*r*) between a neutral site and the arrangement in *In*/*St* heterokaryotypes, which fall within the range reported for single inversions in *Drosophila* (Navarro *et al*. 1997; Korunes and Noor 2019). Given that *r* is expected to be highest in the central regions of inversions, and lowest near their breakpoints (Navarro *et al*. 1997, 2000), increased *r* in the figures can be interpreted as reflecting an increased distance from a breakpoint. Figure 1 plots several expected pairwise coalescent times for within- and between-deme samples against *F_ST_* for the case of an inversion frequency of 0.1. Figure 2 shows the corresponding ratios of *T*_11_/*T*_22_ for within- and between-deme samples, as well as the *F_AT_* statistics. Supplementary Figs. 1 and 2 in the file Supplemental Figures give the results for an inversion frequency of 0.5.

The mean coalescent time between *In* and *St* relative to 2*N_T_*, *T*_12_, is almost the same for within-deme pairs of alleles as for alleles from separate demes (see Equations A6d and A6e), so that only the between-deme values are shown. This property reflects the fact that, with large *d*, the probability that 2 alleles sampled from 2 distinct demes were derived from the same deme in the recent past (where they have an opportunity to recombine) is very small compared with the probability that alleles sampled from the same deme were derived from separate demes (and cannot recombine); given that *m* is assumed to be >>*r*, migration is the major factor affecting alleles sampled from the same deme. This causes the properties of pairs of alleles sampled from the same or different demes (other than their probabilities of coalescing) to converge; there is, of course, no immediate chance of coalescence for 2 alleles sampled from different arrangements.

*T*_12_ provides a measure of the expected net sequence divergence between a pair of alleles from the 2 different karyotypes, relative to the expected within-deme neutral diversity for loci independent of the inversion, or to the expected neutral diversity averaged over *In* and *St* haplotypes sampled randomly from the same deme. This because these diversity measures are both equal to *θ* = 4*N_T_u*, where *u* is the mutation rate per base pair (Nagylaki 1998)—see Equation (3). For *u* = 5 × 10^–9^ (Assaf *et al*. 2017) and *N_T_* = 10^6^, a value of *T*_12_ = 1 corresponds to a sequence divergence of *T*_12_*θ*/2 = 0.01 per base pair, which is approximately equal to the within-population synonymous site diversity in *D. melanogaster* ancestral range populations (Langley *et al*. 2012). As can be seen in Fig. 1, *T*_12_ is quite close to 1 for *r* = 10^–5^ or 10^–4^ with *F_ST_* ≤ 0.05 but takes much larger values for the 2 lower recombination rates. *T*_12_ increases with *F_ST_*, but only slowly at the 2 lower recombination rates, and is at most about 1.5 for the 2 higher *r* values.

Unless *r* is very low, as may be the case near inversion breakpoints (Navarro *et al*. 1997), the equilibrium level of sequence divergence between *In* and *St* is thus likely to be less than 50% larger than the genome-wide mean neutral diversity, corresponding to an ${\tilde{F}}_{AT}\;$ of 0.33. Equation (A6d) shows that the effect of *F_ST_* on *T*_12_ is caused by the increase in divergence between alleles sampled from different karyotypes and different populations as the between-deme coalescent times increase. The effect of recombination on *T*_12_ is controlled by the reciprocal of *ρ*, the recombination rate scaled by 4*N_T_*, which is invariant with respect to the level of population subdivision under the assumptions used here.

A related statistic is the net mean coalescent time for a pair of alleles sampled randomly within demes without regard to karyotype (*T_Tw_*), which determines the overall mean within-deme *π* for nucleotide sites located within the region covered by the inversion. Figure 1 and Supplementary Fig. 1 show that *T_Tw_* is much less sensitive to *F_ST_* than *T*_12_ but increases slightly with increasing *F_ST_*, especially at high recombination rates, reflecting the fact that it is heavily influenced by the within-karyotype coalescent times *T*_11*w*_ and *T*_22*w*_, which either change in opposite directions as *F_ST_* increases (for *x* = 0.1) or remain constant (for *x* = 0.5). This also means that *T_Tw_* < *T*_12_, even for an intermediate frequency inversion, so that the corresponding diversity statistic provides a less useful index of between-karyotype differentiation than the between-karyotype divergence. The corresponding statistic for alleles sampled from different demes (*T_Tb_*) has similar properties but is always somewhat larger in magnitude, especially at high *F_ST_* values, due to the inflation of between-deme coalescent times with restricted migration. Unless *r* is very low, an inversion polymorphism at recombination–drift equilibrium is not expected to have a large effect on the overall level of sequence diversity in the population unless there is a high degree of population subdivision, consistent with the results for chromosome arm 3R of *D. melanogaster* shown in Figure 4 of Corbett-Detig and Hartl (2012).

When the inversion is present at a low frequency, as in Figs. 1 and 2, the mean coalescent times for pairs of alleles sampled within the inversion (*T*_11_) are smaller than those for alleles sampled within *St* (*T*_22_), due to the lower effective population size of carriers of *In*; this also applies to the corresponding *π* values (Nordborg 1997; Navarro *et al*. 2000). The converse is true if *In* is more frequent than *St*. This difference disappears when *In* and *St* have equal frequencies, since their effective sizes are necessarily equal, and is smaller, the smaller the difference in frequencies. Figure 2 shows that the ratio *T*_11*w*_/*T*_22*w*_ decreases considerably as *F_ST_* increases when *r* is relatively large, whereas *T*_11*b*_/*T*_22*b*_ increases slightly. Conversely, *T*_11*b*_/*T*_22*b*_ increases considerably with *F_ST_* when *r* is small, but *T*_11*w*_/*T*_22*w*_ hardly changes. *T*_11*w*_/*T*_22*w*_ is, however, always greater than the value of 0.11 expected from the relative frequencies of *In* and *St*, reflecting the effect of recombination in reducing allele frequency differences; consistent with this property, both *T*_11*w*_/*T*_22*w*_ and *T*_11*b*_/*T*_22*b*_ increase with *r*.

All other things being equal, therefore, the departure of estimates of *T*_11*w*_/*T*_22*w*_ from the ratio of the frequencies of *In* and *St* provides an inverse measure of the extent of differentiation between karyotypes when the frequency ratio departs considerably from unity. The behavior of *T*_11*w*_/*T*_22*w*_ as a function of *r* reflects the fact that, for a low-frequency inversion, *T*_11*w*_ increases considerably with increasing *r* for a given *F_ST_* whereas *T*_22*w*_ decreases slightly, as seen in Fig. 1. This complementary behavior arises from the fact that *xT*_11*w*_ + *yT*_22*w*_ = 1 at equilibrium, by Maruyama's invariance principle (Equation 3). An increase in *F_ST_* results in a reduced effective rate of recombination for within-deme samples, due to increased homozygosity (Wakeley and Lessard 2003) so that *T*_11*w*_ decreases with *F_ST_*, whereas *T*_22*w*_ increases. *T*_11*b*_ and *T*_22*b*_ both increase with *F_ST_*, especially with high *r* values, reflecting the effects of reduced migration rates on coalescence times; for between-deme samples, population subdivision of the kind modeled here does not affect the effective recombination rate for between-deme samples (Wakeley and Lessard 2003).

The between-karyotype analogues of *F_ST_*, *F_AT_*, and ${\tilde{F}}_{AT}\;$ (see Equations 4) are often used as a measure of the extent of genetic differentiation among karyotypes, rather than the absolute divergence measures just discussed. As noted by Charlesworth *et al*. (1997) in the context of a single-locus balanced polymorphism (see also Zeng *et al*. 2021), *F_AT_* is equivalent to the $\sigma_{d\;}^{2}$ measure of LD of Ohta and Kimura (1971), treating the neutral site and karyotype as a pair of loci, but it is considerably less tedious to estimate from population genomic data. Its properties thus differ somewhat from those of *T*_12_ or *T_Tw_*, especially as it is heavily influenced by the within-karyotype coalescent time in the numerator of Equation (4a) (Charlesworth 1998). Figure 2 and Supplementary Fig. 2 show that *F_ATw_* and *F_ATb_* are only weakly affected by the extent of population subdivision at the lower 2 recombination rates, with *F_ATw_* increasing slightly with *F_ST_* whereas *F_ATb_* decreases; *F_ATw_* is always larger than *F_ATb_*, especially when *F_ST_* and *r* are large, and is thus more useful as a measure of between-karyotype differentiation. This difference reflects the effect of population subdivision on recombination in within-deme samples and lack of such an effect for between-deme samples, described above in connection with the ratio *T*_11*w*_/*T*_22*w*_. Both statistics are highly sensitive to *r*, with small values at the 2 higher *r* values, especially for *F_ATb_*. ${\tilde{F}}_{ATw}\;$ for equilibrium populations is given by 1 − 1/*T*_12_ (Equations 4b and 5f), so that it behaves in a similar fashion to *T*_12_ as a function of *r* and *F_ST_* and is therefore not shown here.

A moderately high level of population subdivision in an equilibrium system thus makes it much easier to detect between-karyotype differentiation at low recombination rates, as measured by *F_ATw_*, ${\tilde{F}}_{ATw}$, or *T*_12_, but makes *F_ATb_* a less useful measure. Comparisons of the mean between-karyotype divergence (either within or between demes) with the mean within-deme and within-karyotype diversity for samples at loci independent of the inversion (corresponding to a *T_Sn_* of 1), or with the mean within-deme and within-karyotype diversity (corresponding to a *T_Sw_* of 1), are probably the most useful measures of the extent of between-karyotype divergence, if equilibrium can be assumed.

It is of some interest to compare the values of *F_AT_* for a polymorphism maintained at constant frequencies with the value of $\sigma_{d}^{2}$ for a pair of neutral loci at statistical equilibrium under recombination and drift in a subdivided population. Table 1 shows results for the within-deme measure of *F_AT_* compared with $\sigma_{d}^{2}$ for within-deme samples calculated from the equations in the Appendix to Wakeley and Lessard (2003). For the intermediate and highest recombination rates (*ρ* = 4 and *ρ* = 40), *F_AT_* for *x* = 0.5 is very close to $\sigma_{d}^{2}$, whereas *F_AT_* for *x* = 0.1 is substantially smaller, except for the panmictic case (*F_ST_* = 0); all 3 variables increase considerably with increasing *F_ST_*, reflecting the reduced effective recombination rate when there is extensive population subdivision (Wakeley and Lessard 2003). In contrast, for the lowest recombination rate (*ρ* = 0.4), *F_AT_* for *x* = 0.1 is close to $\sigma_{d}^{2}$ and there is only a small increase in the 3 measures with *F_ST_*. For a pair of neutral loci, $\sigma_{d}^{2}$ for a sample of haplotypes taken from separate demes (which is equivalent to random sampling from the whole population with large *d*) is independent of the extent of subdivision with the model used here (Wakeley and Lessard 2003), in contrast to the behavior of *F_ATb_* shown in Figs. 1 and 2. The results for neutral loci thus shed only limited light on what is to be expected for an inversion polymorphism maintained at a constant frequency. The discrepancy between the statistics for the neutral case and the case with a constant inversion frequency could in principle be used as a test for selection, although this would require accurate knowledge of *r*.

**Table 1.** Comparisons of within-deme *F_AT_* for a balanced polymorphism vs $\sigma_{d}^{2}$ for a pair of neutral loci.

	*ρ* = 0.4			*ρ* = 4			*ρ* = 40		
*F_ST_*	*F_AT_* *x* = 0.1	*F_AT_* *x* = 0.5	$\sigma_{d}^{2}$	*F_AT_* *x* = 0.1	*F_AT_* *x* = 0.5	$\sigma_{d}^{2}$	*F_AT_* *x* = 0.1	*F_AT_* *x* = 0.5	$\sigma_{d}^{2}$
0	0.489	0.714	0.380	0.117	0.200	0.156	0.021	0.024	0.023
0.05	0.492	0.718	0.419	0.130	0.232	0.200	0.051	0.072	0.070
0.10	0.496	0.723	0.454	0.143	0.265	0.241	0.073	0.120	0.114
0.15	0.500	0.728	0.488	0.157	0.299	0.281	0.092	0.167	0.156
0.20	0.504	0.733	0.521	0.171	0.333	0.320	0.110	0.216	0.197
0.25	0.509	0.739	0.552	0.186	0.368	0.358	0.128	0.263	0.238

The scaled recombination rates of *ρ* = 0.4, 4, and 40 correspond to *r* = 10^–7^, 10^–6^, and 10^–5^, respectively, when *N_T_* = 10^6^, as assumed in the figures.

As expected from Equation (5a), a comparison of Figs. 1 and 2 with Supplementary Figs. 1 and 2 shows that *T*_12_ is not greatly affected by the inversion frequency; in contrast, *F_ATw_* and *F_ATb_* are larger with equal frequencies of the 2 karyotypes than when the inversion is either rare or very common. This reflects the smaller *T_T_* values with extreme inversion frequencies, as can be seen from Equation (5d) for the panmictic case, where *T_T_* is approximately 1 + 2*ρ*^−1^ for *x* = 0.5 but approaches 1 + *x*(4*ρ*^−1^ + 1) as *x* tends to 0. This is another reason for using between-karyotype divergence relative to mean within-deme diversity as a measure of differentiation between karyotypes.

## Theoretical results: approach to equilibrium

### General considerations

The above results assume both that the inversion has reached its equilibrium frequency and that there has been sufficient time for the effects of coalescence and recombination to equilibrate. In reality, as was assumed in early hitchhiking models of associations between electrophoretic variants and inversions (Ishii and Charlesworth 1977), a polymorphic inversion is likely to have arisen on a single unique haplotype drawn at random from the initial population. Having survived early stochastic loss, it will have approached its equilibrium frequency over a time that is inversely related to the strength of selection acting on it. Molecular characterizations of inversion breakpoints in *D. melanogaster* and *Drosophila subobscura* strongly support the unique origin hypothesis (Corbett-Detig and Hartl 2012; Orengo *et al*. 2019; Kapun *et al*. 2023). After this selective equilibrium has been approached, there will be another extended period during which mutation–recombination–drift equilibrium is approached. In the case of a panmictic population, both of these episodes can be included in the same model, using phase-type theory (Zeng *et al*. 2021).

But the population genetics of the spread of a new mutation in a subdivided population, and its hitchhiking effects on linked neutral sites, is much more complex (Barton *et al*. 2013) and has not been applied to the case of balancing selection. In the present treatment, the initial approach to the equilibrium inversion frequency is assumed to occur effectively instantaneously, so that only the second phase of the approach to equilibrium is studied. Clearly, this is likely to cause the time taken to approach equilibrium and the effects of recombination during this period to be underestimated, due to the additional time needed for a mutation to spread through a subdivided population compared with panmixia, even if the habitat is 2 dimensional rather than 1 dimensional (Barton *et al*. 2013).

By using this simplifying assumption, we can set *T*_22*w*_ = *T*_22*b*_ = 0, *T*_12*w*_ = *T*_11*w*_ = 1, and *T*_12*b*_ = *T*_11*b*_, with *T*_11*b*_ ≈ 1/(1 − *F_ST_*) when *d* is large. Using these as initial values, the change per generation in the deviations of the *T*'s from their equilibrium values can be calculated by means of the matrix iteration ***x****_n_* = ***A x****_n_*  _− 1_, where ***x****_n_* is the column vector of deviations of the *T*'s in generation *n* from their equilibrium values and the matrix ***A*** is defined at the end of section 1 of Supplementary File 1. This approach breaks down in the absence of recombination, since in this case, the divergence between *In* and *St* increases without limit as time increases; a separate treatment of this case is given in sections 6 and 7 of Supplementary File 1.

In order to speed up calculations, a relatively small total population size (10^5^ for the case of a subdivided population and 10^4^ for the equivalent panmictic population) was assumed, with rescaling of parameters to keep their products with *N_T_* identical with those used for the equilibrium results. Insight into the asymptotic rate of approach to equilibrium when *d* is large can be obtained from the eigenvalues of the ***A*** matrix. As shown in section 3 of Supplementary File 1, if second-order terms in 1/*N*, *r*, and *m* are neglected, the structure of ***A*** is such that its 6 eigenvalues (3 in the panmictic case) are each approximately equal to 1 of its diagonal elements, i.e. to 1 − 2*m* − 2*ry* − 1/(2*Nx*), 1 − 2*m*/*d* − 2*ry*, 1 − 2*m* − 2*rx* − 1/(2*Ny*), 1 − 2 *m*/*d* − 2*rx*, 1 − 2*m* − *r*, and 1 − 2*m*/*d* – *r*. The asymptotic rate of approach to equilibrium is controlled by the largest of these quantities; which of the 6 is the largest is determined by the relative values of *Nx*, *Ny*, *rx*, *ry*, and *m*. Since *m* >> *r* and *d* >> 1 with the approximations used here, either 1 – 2*m*/*d* – 2*ry* or 1 – 2*m*/*d* – 2*rx* will be the largest eigenvalue, showing that the asymptotic rate of approach to equilibrium is largely controlled by the product of 2*r* and the smaller of the 2 frequencies *x* and *y* when *d* is very large. In the case of a panmictic population, the system reduces to a 3-dimensional one with eigenvalues approximately equal to 1 – 2*ry* – 1/(2*Nx*), 1 – 2*rx* – 1/(2*Ny*), and 1 – *r*, so that a similar conclusion applies.

The timescale of the approach of the coalescence times to equilibrium after the inversion has approached its equilibrium frequency is thus of the order of the inverse of *rx* (if *x* ≤ 0.5) or *ry* (if *x* > 0.5), unless *r* is very close to zero—see sections 6 and 7 of Supplementary File 1 for this case. It is nearly independent of population structure with large *d* and *m* >> *r*. The full solution for ***x****_n_* in generation *n* in terms of the representation of ***A^n^*** by the eigenvalues and eigenvectors of ***A*** is given in section 3 of Supplementary File 1. In practice, however, it is simpler to iterate the basic matrix recursion—it takes only a few seconds to iterate several million generations on a laptop computer.

It is also of interest to examine the initial rates of change of the *T*s using the starting point of an instantaneous sweep to equilibrium described above. The corresponding initial values of *F_ATw_* and *F_ATb_* are then both equal to 1 − *y*/(2*xy* + *y*^2^) = *x*/(1 + *x*); ${\tilde{F}}_{ATw}\;$ = ${\tilde{\;F}}_{ATb}$ = *x.* The initial *F_AT_* statistics can thus be substantially different from zero despite the absence of any divergence between *In* and *St*, due to the assumed lack of variability within inverted chromosomes. This is seen in other systems such as X–Y comparisons when a newly evolved Y chromosome lacks variability (Bergero *et al*. 2019; Toups *et al*. 2019; Gammerdinger *et al*. 2020).

Normalizing Equation (A2) by dividing each term by 2*N_T_*, neglecting second-order terms in the products of the deviations of the *T*'s from their equilibrium values with *m*, *r*, and 1/*N*, and exploiting the fact that 4*Nm* ≈ (1 − *F_ST_*)/*F_ST_* in order to simplify Equations (A6c–A6e), the following simple relations hold for the initial changes per generation in the *T*'s:


(6a) $$\Delta T_{11w}\approx \frac{1}{2N_{T}}+2ry$$

(6b) $$\Delta T_{11b}\approx \frac{1}{2N_{T}}+\frac{2ry}{\left(1-F_{ST}\right)}$$

$$\Delta T_{22w}\approx \frac{1}{2N_{T}}+\frac{2mF_{ST}}{\left(1-F_{ST}\right)}-\frac{1}{2Ny}$$

(6c) $$\;=\frac{1}{2N_{T}}+\frac{1}{2N}-\frac{1}{2Ny}(\mathrm{for}m>0)$$

(6d) $$\Delta T_{22b}\approx \frac{1}{2N_{T}}-\frac{2m}{d(1-F_{ST})}=\frac{1}{2N_{T}}-\;\frac{1}{2N_{T}}=0$$

$$\Delta T_{12w}\approx \frac{1}{2N_{T}}+\frac{2mF_{ST}}{\left(1-F_{ST}\right)}-rx$$

(6e ) $$\;=\frac{1}{2N_{T}}+\frac{1}{2N}-rx\;(\mathrm{for}\;m>0)$$

(6f) $$\Delta T_{12b}\approx \frac{1}{2N_{T}}-\frac{rx}{\left(1-F_{ST}\right)}-\frac{2mF_{ST}}{d(1-F_{ST})}\approx \frac{1}{2N_{T}}-\frac{rx}{\left(1-F_{ST}\right)}$$

Using the results in section 4 of Supplementary File 1, we also have the following:


(7a) $$\Delta F_{ATw}\approx -\frac{x}{{\left(1+x\right)}^{2}}[\frac{1}{2N_{T}y}-\frac{1}{Ny}+2r(1+x)]$$

(7b) $$\Delta F_{ATb}\approx -\frac{x}{{\left[1+x(1-F_{ST})\right]}^{2}}\left[\frac{\left(1-F_{ST}\right)}{2N_{T}}+2r(1-2x)\right]$$

**Fig. 3.**
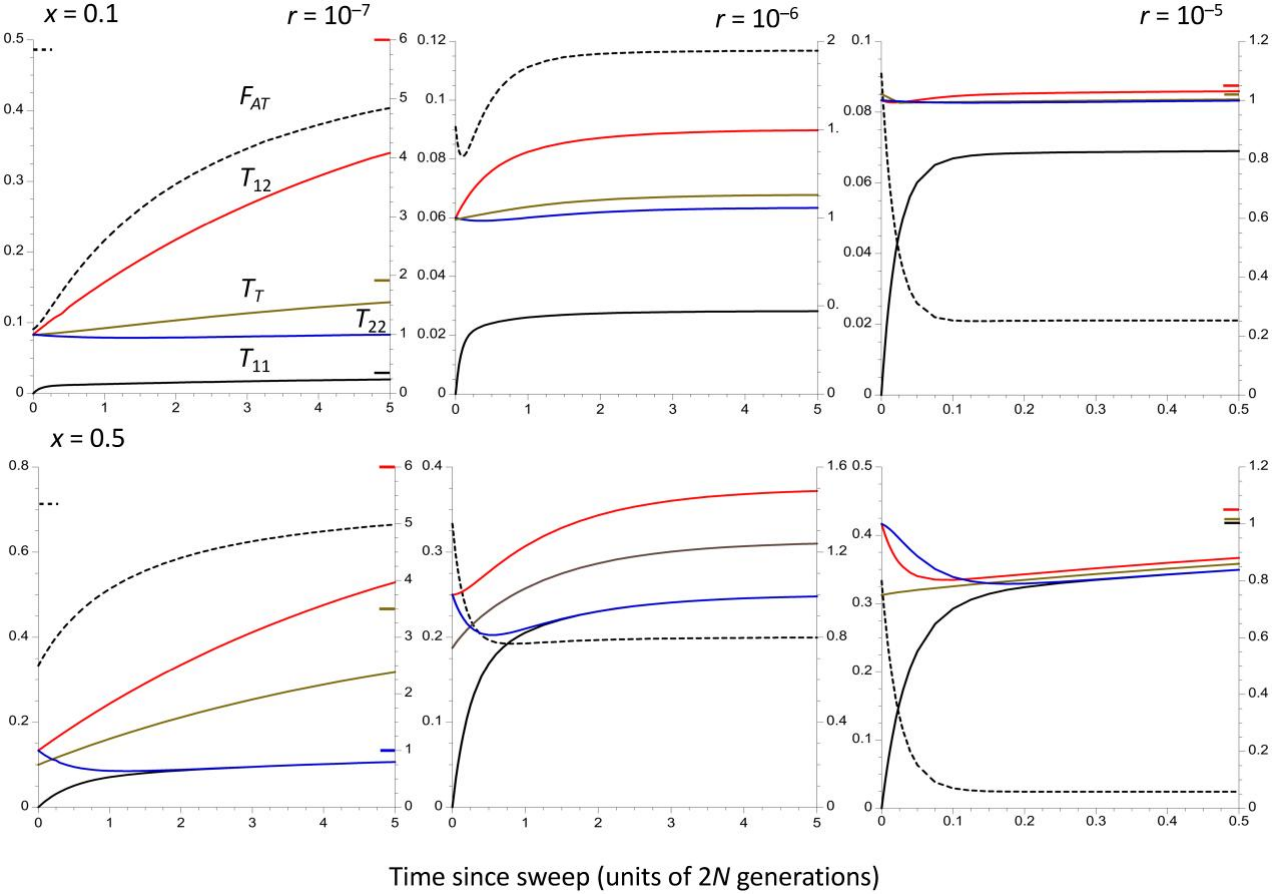
The trajectories of change in the population statistics for the case of a panmictic population of size *n* = 10^6^, assuming that the time taken to approach the equilibrium inversion frequency is negligible compared with the coalescent time of 2*N* generations. The *X* axes display times in units of coalescent time following the sweep to equilibrium. Three different recombination rates in heterokaryotypes are shown, as well as 2 different frequencies of the inversion (0.1 in the upper panels and 0.5 in the lower panels). The dashed curves are *F_AT_*, whose values are displayed on the left-hand *Y* axes. The solid curves are mean coalescent times, measured relative to 2*N* (right-hand *Y* axes); red is *T*_12_, brown is *T_T_*, black is *T*_11_, and blue is *T*_22_. For the highest rate of recombination (*r* = 10^–5^), only the first *N* generations are shown, in order to capture the rapid changes at the start of the process. The colored bars inside the *Y* axes indicate the equilibrium values of the corresponding statistics, for cases when these are substantially different from the final values of the statistics.

**Fig. 4.**
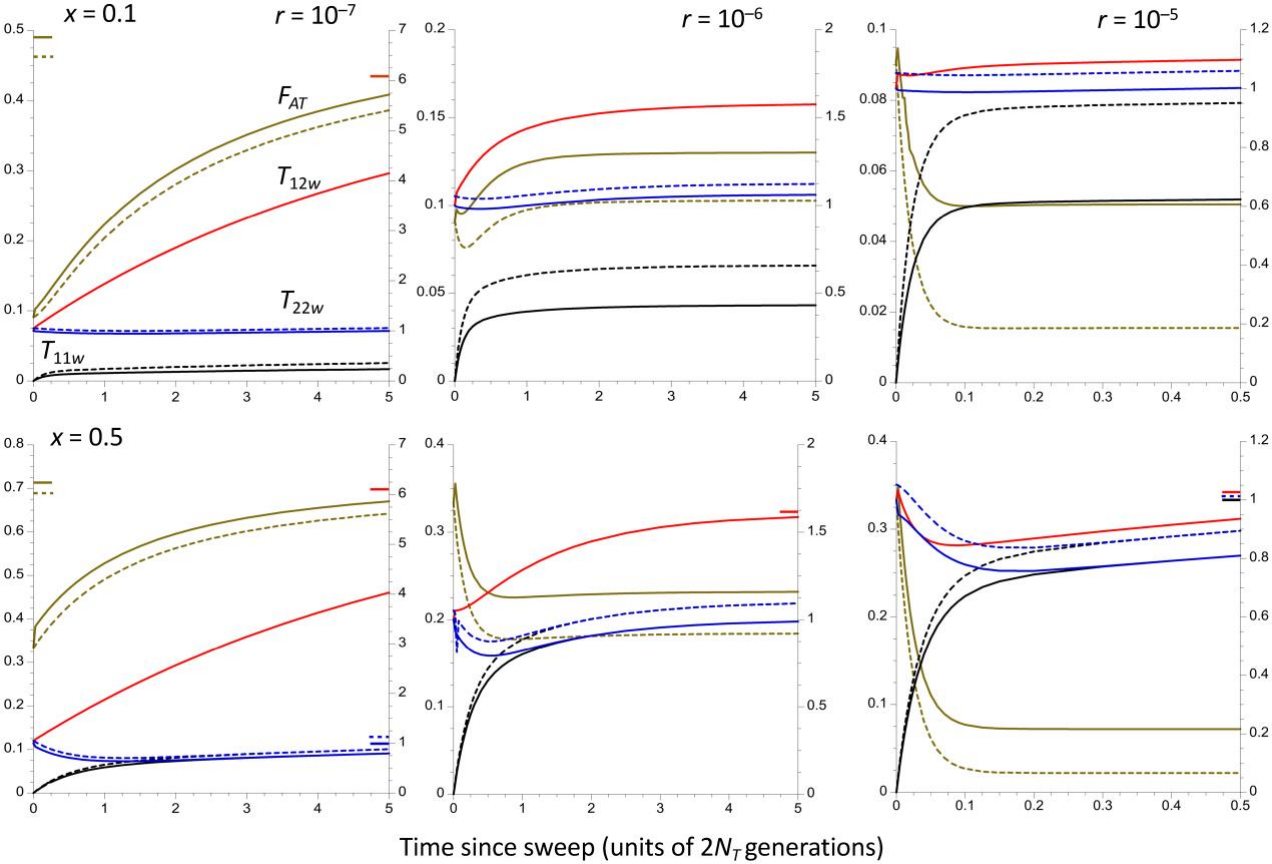
The trajectories of change in the population statistics for the case of a subdivided (island model) population of total size *N_T_* = 10^6^, with 200 demes of size *N* = 500 and an *F_ST_* = 0.05 (4*Nm* = 19) for neutral sites independent of the inversion. It is assumed that the time taken to approach the equilibrium inversion frequency is negligible compared with the coalescent time of 2*N_T_* generations. The *X* axes display times in units of coalescent time following the sweep to equilibrium. Three different recombination rates in heterokaryotypes are shown, as well as 2 different frequencies of the inversion (0.1 in the upper panels and 0.5 in the lower panels). The solid curves represent within-population statistics, and the dashed curves are between-population statistics. The values of *F_ATw_* and *F_ATb_* (brown curves) are given by the left-hand *Y* axes. The other curves are mean coalescent times, measured relative to 2*N_T_*; red is *T*_12*w*_ (*T*_12*b*_ behaves almost identically, except for its higher initial value and slower rate of increase when the time since the sweep is <0.005); black is *T*_11_ and blue is *T*_22_ (right-hand *Y* axes). For the highest rate of recombination (*r* = 10^–5^), only the first *N_T_* generations are shown. The colored bars inside the *Y* axes indicate the equilibrium values of the corresponding statistics, for cases when these are substantially different from the final values of the statistics.

**Fig. 5.**
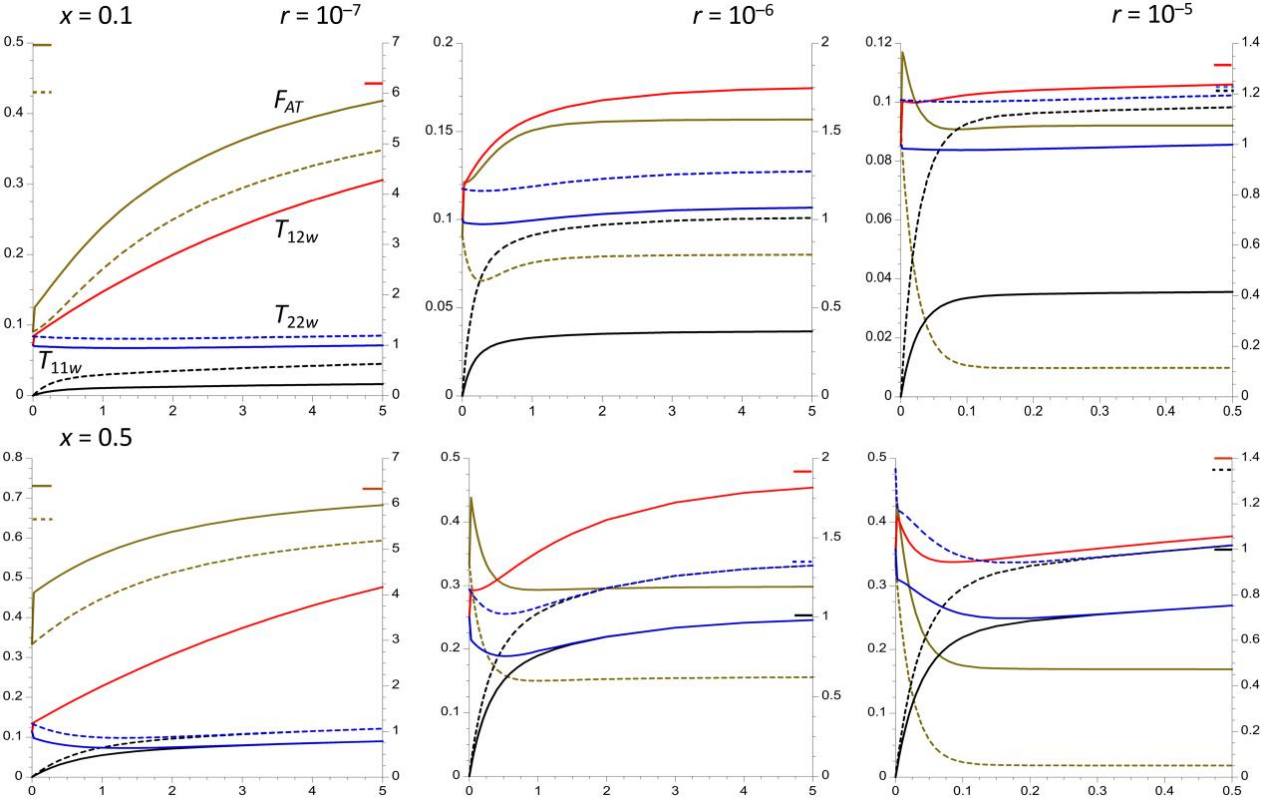
This is the same as Fig. 4, except that *F_ST_* = 0.15 (4*Nm* = 5.67).

These results require *rx* and *ry* to both exceed 1/2*N_T_* and hence are invalid for very low rates of recombination, as in the case of *ρ* = 0.4 shown in Figs. 3–5 below.

Equations (6a), (6c), (6e), and (7a) can be applied to the panmictic case, by setting the right-hand terms involving *m* and 1/2*N* (but not 1/2*Ny*) to zero, and equating *N* and *N_T_*.

### Results for a single randomly mating population

The results for a single, randomly mating population of size *N* = *N_T_* are considered first, assuming an instantaneous sweep of a new inversion to its equilibrium frequency. This case was previously studied by Navarro *et al*. (2000) using coalescent simulations. Some representative results are shown in Fig. 3 for 3 different recombination rates adjusted to the values for *N* = 10^6^, i.e. scaled recombination rates of *ρ* = 4*Nr* = 0.4, 4, and 400, and 2 different equilibrium frequencies, *x* = 0.1 and *x* = 0.5. Time is measured relative to 2*N* = 2*N_T_*, and the results are thus invariant with respect to *N* for constant *ρ* (unless *N* is sufficiently small that the assumptions of the coalescent process are violated). The accuracy of the approximations used to generate these results was checked by coalescent simulations and found to be excellent (see Supplementary Table 1). Further details of the results based on the recursion relations are given in Supplementary Table 3.

As shown above, the initial value of *F_AT_* after an instantaneous sweep of the inversion to its equilibrium frequency is simply *x*/(1 + *x*). *F_AT_* does not necessarily change monotonically over time, as can be seen for the case of *x* = 0.1 with *r* = 10^–6^, where it first decreases and then increases. With the highest recombination rate, the final direction of change of *F_AT_* can be opposite to that for *T*_12_ because its initial value is greater than its equilibrium value, so that *F_AT_* declines over time, reflecting the negative term in *r* in Equation (7a). In contrast, *T*_12_ increases on the approach to equilibrium, after an initial decline when *r* is large, as predicted by Equation (6e) (see the results for *x* = 0.5 and *r* = 10^–5^).

As expected intuitively, and as is consistent with Equation (6a), the mean coalescent time for the inversion (*T*_11_) always increases with time towards its equilibrium value, reflecting the effect of recombination in causing it to share ancestry with the standard sequence and the fact that the inversion has increased in number from a completely bottlenecked haploid population size of 1. Even with the lowest frequency of recombination (*ρ* = 0.4), there is a relatively fast approach of *T*_11_ towards its equilibrium value compared with *F_AT_* and *T*_12_, over a timescale that is much less than the neutral coalescent time. Except for the lowest recombination rate, relatively large values of *F_AT_* and *T*_12_ (when compared with the values for the higher 2 recombination rates) are approached over this timescale, reflecting their large equilibrium values. With the lowest recombination rate, however, there is a long period of time when *F_AT_* and *T*_12_ are both much lower than their equilibrium values. Consistent with Equation (6c), *T*_22_ decreases initially for all 3 recombination rates, although very slowly if *Ny* is close to *N*. In some cases (e.g. with *x* = 0.5 and *r* = 10^–5^), *F_AT_* changes nonmonotonically, with an initial decrease followed by an increase. This reflects the fact that, for these parameter values, Equation (7a) predicts an initial decrease in *F_AT_*, whereas its final value is greater than its initial value.

A simple analytical solution to the trajectories of the mean coalescent times for the case of no recombination can also be derived (see section 6 of Supplementary File 1). In this case, *T*_12_ increases linearly with time on the coalescent timescale (Equation S5a), whereas *T*_11_ and *T*_22_ experience an exponential decay of their deviations from their respective equilibrium values of *x* and *y*, with rate constants *x* and *y*. In the case of an instantaneous sweep of *In* to its equilibrium frequency, this implies that *T*_11_ always increases over time and *T*_22_ always decreases. For sufficiently large *T*, these expressions imply that *F_AT_* increases monotonically towards 1, due to the fact that *T*_12_ increases without being bound in the absence of recombination. Approximations to these expressions for small *T* show that *F_AT_* always increases initially (see Equations (S5e)–(S5i) in Supplementary File 1).

### Results for a subdivided population

The numerical values of the population statistics were generated by using scaled recombination and migration parameters that matched those used for Figs. 1 and 2. Figs. 4 and 5 show the results for 2 different values of *F_ST_* at neutral sites independent of the inversion, corresponding to scaled migration rates of 4*Nm* = 19 and 5.67, respectively. Further details are given in Supplementary Tables 4 and 5.

The results are broadly similar to those for the panmictic case described above. The most notable difference is that there is a short initial period with rapid increases in *T*_12*w*_ and *F_ATw_*. For *F_ATw_*, this period is followed by a monotonic decrease if the equilibrium value of *F_ATw_* is lower than the maximum values it achieves, or (in the case of *x* = 0.1 and *r* = 10^–6^) a decrease followed by an increase, when its equilibrium value exceeds its minimum value. In contrast, *T*_12*b*_ and *F_ATb_* behave initially much like *T*_12_ and *F_AT_* in the panmictic case. For the case of *r* = 0 and a large number of demes (*d*), Equations (S9c) and (S9d) with very small *dMT* show that *T*_12*w*_ ≈ 1 + *dT*, whereas *T*_12*b*_ ≈ (1 + *M*)/*M* + *T*.

The numerical results used in Figs. 4 and 5 show, however, that *T*_12*w*_ and *T*_12*b*_ quickly converge and have nearly identical trajectories after scaled time *T* = 0.005 (about 10,000 generations with the parameters used here). It is easily shown from Equations (A2e) and (A2f) for the case of no recombination and an instantaneous sweep of the inversion that


(8) $$\;T_{12b}(T)-T_{12w}(T)\approx [T_{12b}(0)-T_{12w}(0)]\exp(-dMT)=\;M^{-1}\exp(-dMT)$$

The 2 measures of *T*_12_ thus converge rapidly when *r* = 0; since it is assumed here that migration is a more powerful force than recombination, this is true more generally.

Similarly, Equation (S20e) shows that the coefficient of *T* in the numerator of the expression for *F_ATw_* with no recombination involves *dx*, so that *F_ATw_* also increases very fast initially when the number of demes is large, especially if *x* is close to 1. In contrast, there is no contribution from *d* to the expression for *F_ATb_* (Equation S20f); *F_ATb_* starts, however, from a higher initial value than *F_ATw_* because of population subdivision.

When the recombination rate is sufficiently high, Equations (6) and (7) for the initial rates of change in the population statistics can be applied. Δ*T*_12*w*_ in Equation (6e) involves the sum of 1/(2*N_T_*) and 1/(2*N*), whereas the term in 1/(2*N*) is absent from the corresponding expressions for Δ*T*_12*b*_ as well as Δ*T*_12_ in the panmictic case. Since 1/(2*N*) is larger than 1/(2*N_T_*) by a factor of *d*, this term has a large effect on the initial rate of change of *T*_12*w*_. Similarly, the expression for Δ*F_ATw_* (Equation 7a) involves the positive term (1 – 1/2*d*)/*Ny*, whereas the corresponding expression for Δ*F_ATb_* involves (1 – *F_ST_*)/(2*Nd*), which is negative. These results show how it is possible for the 2 measures to change initially in opposite directions.

## Discussion

### General considerations

The results described here are broadly consistent with a previous theory on the patterns of diversity associated with balanced polymorphisms (Hudson and Kaplan 1988; Hudson 1990; Takahata 1990; Charlesworth *et al*. 1997; Nordborg 1997; Navarro *et al*. 2000; Charlesworth *et al*. 2003; Innan and Nordborg 2003; Nordborg and Innan 2003; Guerrero *et al*. 2012; Kirkpatrick and Guerrero 2014; Zeng *et al*. 2021) but extend it in several ways. From the purely technical point of view, the recursion relations for mean pairwise coalescence times in a structured population (Nagylaki 1998) provide a simple and computationally efficient method for calculating the expected values of population statistics and their trajectories, on the assumption that the alleles at the target of selection are maintained at constant frequencies. The only previous application of this method to balanced polymorphisms appears to have been that by Zeng *et al*. (2021) for the case of a single population.

Here, this method has been extended to an autosomal balanced polymorphism in a subdivided population, for the simple case of a finite island model of *d* demes of equal size *N*, under a Wright–Fisher model of drift for which the effective population size of a single deme equals *N*. The “migration effective population size” (Nagylaki 1998), which determines the mean coalescent time for a pair of alleles drawn from the same deme at a locus independent of the balanced polymorphism, is then given by *N_T_* = *Nd*, provided that *Nm* is not very close to 0. This result applies more widely to all conservative migration models, where the mean allele frequency across demes is not changed by migration (Nagylaki 1982, 1998). For more general drift models, *N* can be replaced by *N_e_*, the effective population size of a deme, given certain restrictions (Charlesworth and Charlesworth 2010, p.327). At recombination–drift equilibrium, the mean coalescent time for this sampling scheme is also equal to the mean within-karyotype coalescent time, if coalescent times for the 2 arrangements are weighted by the arrangement frequencies (Equation 3) and *N_T_r* is not very close to 0. For this reason, the various coalescent times used here have been expressed relative to 2*N_T_*.

For simplicity, the rest of the discussion will refer only to 2 arrangements with respect to an inversion polymorphism, but identical results apply to other types of diallelic polymorphisms. In principle, it is possible to extend this approach to more general situations, such as polymorphisms for multiple different arrangements, sex chromosomes, and nonrandom mating populations, as well as changes in population size. Its main drawback is that the main results are limited to expected pairwise coalescent times and do not provide information on features such as the site frequency spectra of neutral sites linked to the target of balancing selection. For these properties, either coalescent simulations or more complex analytical approaches, such as the phase-type theory of Zeng *et al*. (2021), are needed. An extension of Nagylaki’s (1998) recursion equation approach can be used for obtaining the variances and higher moments of pairwise coalescent times, as described in section 8 of Supplementary File 1. The statistical properties of mean pairwise diversity and divergence measures over large numbers of nucleotide are discussed in the light of the variances obtained in this way in section 9 of Supplementary File 1.

### Interpreting population genomic data on inversion polymorphisms

The results on equilibrium-expected coalescent times for pairs of alleles (Figs. 1 and 2 and Supplementary Figs. 1 and 2) suggest that the scaled expected coalescent time *T*_12_ for a pair of alleles sampled from the 2 different arrangements (*In* and *St*) provides the most meaningful basis for interpreting their level of sequence divergence. When the number of demes is large, Equation (A6e) shows that there is essentially no difference in equilibrium *T*_12_ between alleles sampled from the same deme versus alleles from 2 different demes, at least when *F_ST_* at neutral sites independent of the inversion is moderate, for the reasons described in the section *Numerical results for subdivided populations*.

The numerical results on the time courses of the *T_ij_*'s with population subdivision (Supplementary Tables 4 and 5) show that the lack of dependence of *T*_12_ on the nature of the sample (within or between demes) holds very soon after the equilibrium frequency of the inversion has been reached. In addition, except for the lowest scaled recombination rate considered here (*ρ* = 0.4), the ratio of *T*_12_ to the weighted mean scaled coalescent time within karyotypes and within demes (*T_Sw_*) approaches its equilibrium value quite fast, even when both variables deviate considerably from their equilibrium values. For example, with panmixia, *x* = 0.1, and *ρ* = 4, the equilibrium value of *T*_12_/*T_S_* is 1.5 (*T_S_* = 1); at time *T* = 0.050, *T*_12_ = 1.039, *T_S_* = 0.916, and *T*_12_/*T_S_* = 1.14; at *T* = 0.5, *T*_12_ = 1.250, *T_S_* = 0.926, and *T*_12_/*T_S_* = 1.37. With *ρ* = 40, the equilibrium *T*_12_/*T_S_* is 1.05; at *T* = 0.050, *T*_12_/*T_S_* = 1.039; by *T* = 0.1, *T*_12_/*T_S_* = 1.04. It is, however, not always the case that *T*_12_/*T_S_* is initially lower than its equilibrium value; for *x* = 0.5 and *ρ* = 4, its initial value is 2 compared with 1.73 at *T* = 0.1 and a final value of 1.55.

These findings are important for the interpretation of population genomic data for several reasons. First, if there is evidence of significant population subdivision at neutral loci independent of the inversion, the measure of sequence divergence between *In* and *St*, estimated by Nei's *d_xy_* (Nei 1987), is best obtained from the mean of within-population samples, since its expectation is equal to 2*N_T_T*_12_*u* and 4*N_T_u* is equal to the expected within-population neutral *π* in the absence of an inversion polymorphism. Second, *T*_12_/*T_Sw_* (which is equal to *T*_12_ for an equilibrium population) can be estimated by dividing an estimate of *d_xy_* either by an estimate of the mean within-karyotype, within-population diversity (*π*_S*w*_), or by an estimate of the corresponding mean *π* at independent neutral sites (*π_nw_*). This is because the expectations of both these statistics are equal to 4*N_T_u* under the infinite sites model of mutation (for the same mutation rate), unless there is a very low frequency of recombination or the inversion has arisen very recently. To control for possible differences in mutation rate, it is preferable to use the ratio of an estimate of *d_xy_* to an estimate of *π_Sw_* in order to obtain *T*_12_. As discussed above in the section *The model and its analysis*, the theoretical value of the commonly used statistic ${\tilde{F}}_{ATw},\;$ obtained by applying the < *F_ST_* > statistic of Hudson, Slatkin *et al*. (1992) to *In* versus *St* haplotypes is equivalent to 1 − *T_Sw_*/*T*_12_. However, reciprocals have undesirable statistical properties if means are to be taken over sets of sequences; it is thus preferable to estimate *T*_12_ directly.

This raises the question of whether recombinational exchange in heterokaryotypes occurs at a sufficient rate that the condition *T_Sw_* = 1 is likely to hold. At least for simple inversions (with only a single pair of breakpoints), there is ample experimental evidence for recombination between *In* and *St* at sites within and adjacent to the inversion in species of *Drosophila*, much of which appears to be caused by gene conversion rather than double crossovers (Chovnick 1973; Korunes and Noor 2019; Koury 2023; Li *et al*. 2023), with *r* = 10^–5^ per base pair per generation in female meiosis being a commonly accepted typical rate for central regions of inversions (Chovnick 1973; Korunes and Noor 2019); for autosomal loci, the lack of recombination in males means that the effective *r* is half of this value. Complex inversions, with 3 or more breakpoints, might be expected to have much lower rates of exchange than simple inversions, but heterozygotes for multiply inverted chromosomes in *D. melanogaster* have been found to experience noncrossover-associated gene conversion events at rates that are even higher than those in the absence of inversions (Crown *et al*. 2018), so that this expectation may not be well founded.

The occurrence of such “gene flux” (Navarro *et al*. 1997) is consistent with numerous observations of substantial nucleotide site diversity within *In* karyotypes, but at a reduced level compared with genome-wide diversity. In many *Drosophila* examples, *F_AT_* and/or ${\tilde{F}}_{AT}$ increase and within-inversion diversity levels decrease, towards the inversion breakpoints (Andolfatto *et al*. 2001; Nobréga *et al*. 2008; Corbett-Detig and Hartl 2012; Kapun *et al*. 2016, 2023), suggesting that flux is lowest near the breakpoints and highest towards the center of the inversion; an exception is the polymorphism in *D. subobscura* for the overlapping pair of inversions O_3+4_ versus the standard arrangement O_st_ (Munté *et al*. 2005; Papaceit *et al*. 2013).

Patterns of this kind do not, however, necessarily imply a complete absence of exchange at the breakpoints. Rozas *et al*. (1999) obtained population genetic evidence for gene conversion events near the breakpoints of arrangements in the O chromosome system of inversions in *D. subobscura*, and Li *et al*. (2023) found no evidence for an effect of proximity to breakpoints for the *dl-49* inversion of *D. melanogaster* on the rate of gene conversion. There are, however, mechanistic reasons for believing that exchange rates will be lowest at the breakpoints and highest in the center of a simple inversion (Navarro *et al*. 1997). Population genomic data, which reveal the effects of recombination over very long time spans compared with laboratory crosses, offer an excellent opportunity for exploring the effects of breakpoints on recombination, as described in the next section.

### Estimating the exchange parameter

If equilibrium can be assumed, estimates of between-arrangement divergence and within-arrangement diversities can be compared with the theoretical predictions for the corresponding variables, in order to obtain estimates of *r* (mutation rate estimates are also needed to estimate *r* rather than *ρ*). Figures 1 and 2 show that, for a low-frequency inversion, *T*_11*w*_/*T*_22*w*_, as well as *T*_12_, are strongly affected by the level of population subdivision, unless recombination is rare or absent. Even a modest change from *F_ST_* = 0 to *F_ST_* = 0.05 causes this ratio to shift from close to 0.8 to approximately 0.6 when *r* = 10^–5^ and the inversion frequency is 0.1 (Figure 2), reflecting the effect of population subdivision in multiplying the effective rate of recombination within populations by a factor of 1 − *F_ST_* (Wakeley and Lessard 2003). Estimates of *F_ST_* ideally need to be included in any attempts to estimate *r*. Another difficulty here is that the current model assumes a long-term constancy of the inversion frequency over time, as well as an absence of among population variation in inversion frequencies between populations. Many studies of *Drosophila* and other groups have revealed major differences among populations in the frequencies of polymorphic inversions, often clinal in nature, which are likely to reflect locally varying selection pressures on genes contained in the inversion, e.g. Krimbas and Powell (1992b), Aulard *et al*. (2002), Cheng *et al.* (2012), Kapun and Flatt (2019), and Mérot *et al*. (2021). Further work is needed to evaluate the consequences of relaxing the assumption of constancy.

This raises the question of whether equilibrium can safely be assumed. Studies of inversion polymorphisms in many *Drosophila* species suggest that they rarely persist beyond species boundaries (Krimbas and Powell 1992a). There are examples of very close relatives with totally different sets of inversion polymorphisms and many fixed differences with respect to gene arrangements, e.g. *Drosophila miranda* versus *Drosophila pseudoobscura* (Bartolomé and Charlesworth 2006). It is thus quite likely a priori that the times of origin of many polymorphic inversions are only a small fraction of the mean neutral coalescent time, 2*N_T_*, as was proposed by Andolfatto *et al*. (2001) in their review of early data on the molecular population genetics of *Drosophila* inversions.

Figures 3–5 show that for the 2 higher recombination rates 10^–6^ and 10^–5^ corresponding to scaled recombination rates (*ρ*) of 4 and 40, the times for statistics such as *T*_11_, *T*_12_, and *F_AT_* to approach their equilibrium values after the inversion has approached its equilibrium frequency are either commensurate with 2*N_T_* (*r* = 10^–6^) or much smaller (*r* = 10^–5^), consistent with the theoretical prediction that the timescale of approach to equilibrium in terms of generations is of the order of 1/*rx* when *x* < 0.5 both for a panmictic population and a subdivided population with a large number of demes (see section 2 of Supplementary File 1). In the absence of recombination, however, diversity within the inversion recovers over a timescale of 2*xN_T_* generations for a panmictic population and 2(1 + *Mx*)*N_T_*/*M* generations for a subdivided population (see Equations S5b and S18a), so that the signature of the selective sweep of the inversion on within-karyotype diversity can persist for a long time, as has been noted previously (Navarro *et al*. 2000; Zeng *et al*. 2021). In addition, the fact that the ratio *T*_12_/*T_Sw_* converges on its equilibrium value much faster than the absolute *T_ij_*'s (see the above section *Interpreting population genomic data on inversion* polymorphisms) means that it is not necessary to assume that the population is very close to equilibrium when using the corresponding divergence to diversity ratio as a statistic.

If recombination in heterokaryotypes is as frequent as is suggested by the data on *Drosophila* (Korunes and Noor 2019), the assumption of recombination–drift equilibrium may thus often be reasonably accurate as a predictor of observed patterns of population genomic statistics for genomic regions covered by an inversion polymorphism, at least for sites that are located well away from inversion breakpoints. The equilibrium assumption can be tested by comparing the within-*In* neutral diversity level with the diversity at neutral sites independent of the inversion (or against the within-*St* diversity). A very recent sweep of the inversion, leaving the system far from equilibrium, would cause diversity across the whole length of the inversions to be much lower than a fraction *x* of the diversity outside the inversion or a fraction *x*/(1 − *x*) of diversity within the standard arrangement—see the curves in Figs. 3–5. It would also be expected to leave a signature of an excess of low-frequency variants at neutral sites compared with what is seen at comparable sites independent of the inversions (Navarro *et al*. 2000; Andolfatto *et al*. 2001; Zeng *et al*. 2021), although the effects of background selection and selective sweeps within the low recombination environment created by a low-frequency inversion could also cause such a pattern, which is also affected by population size changes (Charlesworth and Jensen 2021). The inversion *In(1)Be* in African populations of *D. melanogaster* is an example of a very recent spread of an inversion (Corbett-Detig and Hartl 2012).

The study by Kapun *et al*. (2023) of *In(3R)P* in the Zambian population (ZI) of *D. melanogaster* provides an example of how to apply the theoretical results. As mentioned in the section *Numerical results for subdivided populations*, panmixia can be probably be assumed for this case, which implies that Equations (5) can be used for the expected coalescence times. Let *X* = *T*_11_/*T*_22_. Using Equations (5b) and (5c), simple algebra yields the following formula for *ρ*, the scaled recombination rate:


(9) $$\;\rho =\frac{X(1-2x)}{(1-X)xy}$$

Figure 2 of Kapun *et al*. (2023) shows that the mean nucleotide site diversity (across all classes of nucleotide sites) is significantly lower (0.00792) for the *In(3R)P* haplotypes than for the standard haplotypes (0.00979), giving an estimate of *T*_11_/*T*_22_ = 0.00792/0.00979 = 0.81 for the region covered by the inversion; *x* ≈ 0.11 for this population (Kapun and Flatt 2019, Supplementary Table 1). Substituting these numbers into Equation (10) yields an estimate of 34 for *ρ*, corresponding to *r* = 5.3 × 10^–6^ with *N_e_* = 1.6 × 10^6^, the estimate for this population obtained by Johri *et al*. (2020), which is close to the estimate of 5 × 10^–6^ from crossing experiments after adjustment for the lack of recombination in males (Korunes and Noor 2019). The estimate of diversity for regions outside the inversion on chromosome 3 is 0.00854, giving an estimate of *T*_11_/*T_S_* = (0.11 × 0.00792 + 0.89 × 0.00979)/0.00854 of 1.12, slightly higher than the theoretical value of 1 for equilibrium; this discrepancy probably reflects different levels of selective constraints among the genome regions being compared and/or the fact that inversions suppress exchange well outside their breakpoints (Koury 2023; Li *et al*. 2023).

Figure 4 of Kapun *et al*. (2023) shows that ${\tilde{F}}_{AT}\approx 0.1$ for the central region of *In(3R)P* in the Zambian population. Equation (3a) implies that


(10) $$\rho =\frac{2(1-{\tilde{F}}_{AT})}{{\tilde{F}}_{AT}}$$

This expression yields an estimate of *ρ* = 18, considerably less than the above value. This may be due to the fact that the estimate of ${\tilde{F}}_{AT}$ involves taking the mean of a reciprocal over 100-kb windows, which biases it toward low values compared with using the mean of estimates of *T*_12_.

The empirical results on *In(3R)P* are thus consistent with this inversion being close to recombination–drift equilibrium, with a mean *r* for central regions of the inversion of approximately 5 × 10^–6^. The noticeable increase in within-inversion diversity towards the middle of the inversion (Supplementary Fig. 1 of Kapun *et al*. 2023) and the increase in ${\tilde{F}}_{AT}\;$ near the breakpoints (their Fig. 4) strongly indicate that flux rates are reduced close to the breakpoints. The analysis of the diversity data near the breakpoints described below suggests, however, that this reduction is not total.

### Estimating the ages of inversions: divergence between *In* and *St*

If exchange was indeed completely suppressed around inversion breakpoints, divergence between *In* and *St* for sequences close to the breakpoints could be used to estimate the date of origin of an inversion (Hasson and Eanes 1996; Andolfatto *et al*. 2001; Cáceres *et al*. 2001; Corbett-Detig and Hartl 2012). In this case, it can no longer be assumed that *T_Sw_* = 1 for the sequences concerned. Given the reservations about whether such suppression of exchange is absolute, however, caution should be used in making such inferences.

If, as is usually assumed, the new arrangement had a unique origin, *T*_12_ in the absence of exchange is equal to the value for a pair of randomly chosen alleles in the ancestral population. In the case of a panmictic Wright–Fisher population of constant size, *T*_12_ at time *T* since the origin of the inversions is given by 1 + *T* (Equation S5a). With population subdivision, this expression is no longer accurate; if *dMT* >> 1 and *d* is large, *T*_12_ ≈ (1 − *F_ST_*)^−1^ + *T* for both within- and between-deme measures of *T*_12_ (see the approximation to Equation (S9b)). The first term here corresponds to the mean coalescent time for pairs of alleles sampled from different demes. For species like *D. melanogaster*, with *N_T_* ≈ 10^6^ for populations in the ancestral species range (Lack *et al*. 2015), a *T* value of 0.1 corresponds to 200,000 generations; with *F_ST_* = 0.05, (1 − *F_ST_*)^−1^ is approximately 50% of this, so that ignoring this term could create a substantial error in the estimated time of origin. The situation would clearly be worse for a species with a higher level of population subdivision.

Under these conditions and assuming population size stability, if there is population subdivision, the neutral diversity estimated from pairs of alleles that are independent of the inversion and are sampled randomly across populations (*π_nT_*) can be treated as a proxy for the corresponding mean coalescence time and subtracted from *d_xy_*. The ratio of this corrected value to the neutral diversity statistic then provides an estimate of *T* as defined here. For a very recent origin of the inversion, full Equation (S9b) would usually have to be applied if there is significant population subdivision. The simple approximations of Equations (S9c) and (S9d) could be used when *dMT* << 1, which corresponds to an extremely recent origin when *M* ≥ 1, as is likely to be the case for between-population differences within species of most outcrossing species of animals and plants (Charlesworth 2003; Roux *et al*. 2016).

In practice, different authors have used different methods for estimating *T* from divergence between arrangements. Taking some of the pioneering studies as examples, Andolfatto *et al*. (1999) used the number of fixed differences between *In* and *St*, which is strongly affected by sample size. Cáceres *et al*. (2001), Hasson and Eanes (1996), and Corbett-Detig and Hartl (2012) used *d_xy_* corrected by subtraction of the within-*St* diversity. Somewhat different values will be generated by each of these methods. Difficulties clearly arise if there is evidence for recent strong population expansions or contractions, so that the current population statistics cannot be equated to their values at the time of origin of *In*. There are also likely to be considerable statistical errors, especially as the *d_xy_* values are often very small for *D. melanogaster* inversions (see Supplementary Table 2 in Corbett-Detig and Hartl 2012).

### Estimating the ages of inversions: diversity within *In*

The nucleotide site diversities within arrangements also provide information on the age of an inversion, assuming an absence of exchange. If the inversion frequency is low, however, the within-inversion within-deme diversity (*π*_11*w*_ = 2*N_T_T*_11*w*_*u*) is only a small fraction of the diversity within the standard sequence or at sites independent of the inversion (see Fig. 1), so that a reduced level of within-inversion diversity does not necessarily imply a recent origin of the inversion. In the case of a subdivided population, provided that *dT* >> *x*(1 + *Mx*), Equation (S18a), which corresponds to the exponential growth model used for simulation-based estimates of inversion age by Corbett-Detig and Hartl (2012), implies that


(11) $$T\approx -x\;\ln(x-T_{11w})$$

This expression is independent of *M* and is only meaningful if *T*_11*w*_ < *x*. Given the values of *x*, mean diversity within the inversion (*π*_11*w*_), and *π_nw_* as defined above, it is possible to estimate *T* from this formula, equating *π*_11*w*_/*π_nw_* to *T*_11*w*_. For example, Table 1 of Andolfatto *et al*. (1999) provides estimates of diversity values of 0.0009 and 0.0125 for a breakpoint of *In(2L)t* and for independent sites, respectively, in a *D. melanogaster* population with an inversion frequency of 0.23. *T*_11*w*_ is thus estimated as 0.0009/0.0125 = 0.072, giving *T* = 0.42. Andolfatto *et al*. (1999) estimated the time to the most common recent ancestor of the sequences around the breakpoint from the standard neutral coalescent formula as 0.15 in the present notation; since this method ignores the effect of the recovery from the sweep of the inversion on the gene tree, it overestimates the opportunity for diversity to recover from its complete loss and hence underestimates the time since the origin of the inversion.

Similar calculations can be applied to the data in Fig. 2 and Supplementary Fig. S1b of Kapun *et al*. (2023) on the Zambian population of *D. melanogaster* for sites within 100 kb of the distal and proximal breakpoints of *In(3R)P*. These yield estimates of *T*_11*w*_ of 0.59 and 0.11. These are highly discordant, reflecting the much lower diversity at the proximal breakpoint, which is also seen in the European and North American samples. Given the estimated frequency of 0.11 for *In(3R)P* in this population (Kapun and Flatt 2019, Supplementary Table 1), these estimates of *T*_11*w*_ are inconsistent with a total absence of exchange near the breakpoints. The corresponding values of *T*_22*w*_ are 1.28 and 1.13, yielding estimates of *T_Sw_* of 1.20 and 1.02, respectively, so that the data are reasonably consistent with recombination–drift equilibrium for sites close to the breakpoints, although the flux rates must be substantially lower than the mean of 5 × 10^–6^ estimated for the central region of the inversion (see the above section of the Discussion, *Estimating the exchange parameter*).

The numerical results in Supplementary Table 1 for the panmictic case suggest *r* values around 10^–7^ and 10^–6^ for the distal and proximal breakpoints, respectively. Caution should, however, be used in interpreting these conclusions, as they are highly sensitive to the frequency of the inversion in question and there is considerable continent-wide variation in the frequency of inversions such as *In(3R)P* within Africa (Kapun and Flatt 2019, Supplementary Table 1); Sprengelmeyer *et al*. (2020) estimated a frequency of 0.23 for a small Zambian sample. However, the data for Zambian, Bechuanaland, and Swaziland samples in Table 1 of Aulard *et al*. (2002) yield an overall frequency of 0.11 with s.e. of 0.024, so that a frequency around 0.10 for this region of Africa seems reasonable.

An alternative has been to assume that the recent sweep of an inversion results in a star phylogeny, so that the expected pairwise diversity within the inversion (in the absence of exchange) is equal to 2*Tu* (Rogers 1995); Rozas *et al*. (1999) and Nobréga *et al*. (2008) used this method to estimate the ages of several inversions of *D. subobscura*. Since a star phylogeny must have the smallest mean divergence between a pair of alleles compared with a gene tree with 1 or more coalescent events after the origin of the inversion, this method overestimates the age of the inversion.

### The relevance of *F_A_*_T_ and ${\tilde{F}}_{AT}$

Another point concerns the interpretation of *F_AT_*, the analogue of *F_ST_* for differentiation between *In* and *St*, and the related statistic ${\tilde{F}}_{AT}$—see Equation (4). These statistics are sometimes loosely referred to as measures of the extent of divergence between *In* and *St*, e.g. Guerrero *et al*. (2012), Kennington and Hoffmann (2013), and Kapun and Flatt (2019). As noted in the section *Theoretical results: Approach to equilibrium, General considerations*, the fact that *F_AT_* and ${\tilde{F}}_{AT}\;$ do not necessarily measure the extent of divergence between karyotypes is brought out by their high initial values immediately after an instantaneous sweep to an equilibrium frequency of *x*, for which *F_AT_* = *x*/(1 + *x*) and ${\tilde{F}}_{AT}$ = *x*. This reflects the fact that *In* and *St* are initially no more divergent on average than a random pair of sequences, but there is no diversity within the inversion. Furthermore, in the presence of recombination, *F_AT_* can reach an equilibrium level that is lower than its initial value whereas *T*_12_ increases above 1 (e.g. Figure 4, lower middle panel), so that the 2 statistics can change in opposite directions over time (see also Zeng *et al*. 2021, Fig. 8).

As described earlier, *F_AT_* is equivalent to the measure of LD $\sigma_{d}^{2}\;$ of Ohta and Kimura (1971), which is approximately equal to *R*^2^, the squared correlation coefficient between 2 loci of Hill and Robertson (1968), so that the use of *F_AT_* is effectively equivalent to estimating LD between SNPs and karyotype. This equivalence does not hold for ${\tilde{F}}_{AT}$, however, which yields much larger values than *F_AT_* for equilibrium situations (Charlesworth 1998; Gammerdinger *et al*. 2020), and has frequently been used to characterize differentiation between arrangements (e.g. Corbett-Detig and Hartl 2012, Kapun *et al*. 2023). This difference arises because the theoretical value of ${\tilde{F}}_{AT}$ is equal to 1 − *T_S_*/*T*_12_ whereas *F_AT_* = 1 − *T_S_*/*T_T_* with *T_T_* < *T*_12_ at recombination–drift equilibrium (see Equations 4). The difference between them can be considerable; Table 2 of Laayouni *et al*. (2003) shows both statistics for inversion polymorphisms of *Drosophila buzzatii*, with approximately 4-fold larger values of ${\tilde{F}}_{AT}\;$ compared with *F_AT_.* If data on diversity within inverted and standard haplotypes are available, as well as an estimate of *x*, it is possible to determine *F_AT_* from an estimate of ${\tilde{F}}_{AT}\;$ and vice-versa. In the case of *In(3R)P* in the Zambian population of *D. melanogaster*, the mean ${\tilde{F}}_{AT}$ value of 0.1 combined with the diversity values in Fig. 2 of Kapun *et al*. (2023) gives mean *F_AT_* ≈ 0.033, close to a direct estimate (Thomas Flatt and Martin Kapun, personal communication). The magnitude of LD between single nucleotide variants (SNPs) and an inversion polymorphism can therefore be rather small, even when there is noticeable sequence differentiation between arrangements.

The larger expected equilibrium value of ${\tilde{F}}_{AT}$ compared with *F_AT_*, as well as its closer relationship with the divergence between arrangements, makes it a more powerful measure of the extent of differentiation between arrangements relative to within-arrangement diversity. It is also more convenient for use with population genomic data based on haploid genome sequences, as is the case for much of the *Drosophila* Genome Nexus data (Lack *et al*. 2015, 2016) and for the data in Kapun *et al*. (2023), because it does not require the reconstruction of the frequencies of diploid genotypes used for calculating the mean diversity over a random set of individuals. As pointed out above, however, direct estimates of *T*_12_ have better statistical properties.

### LD among neutral variants associated with inversions

As just discussed, estimates of *F_AT_* can be used to estimate LD between neutral variants such as silent site SNPs and an inversion polymorphism. It is also of interest to examine LD between the SNPs themselves, as this has been used to infer the existence of inversion polymorphisms in nonmodel organisms from evidence for large blocks of LD in specific regions of the genome (e.g. Faria *et al*. 2019; Mérot *et al*. 2021). It is difficult to obtain exact analytical results on LD in structured populations (Wakeley and Lessard 2003), but some approximations are easily obtained. From Equation (A9b) of Charlesworth *et al*. (1997), if LD within arrangements is small compared with LD between neutral sites and the inversion polymorphism, $\sigma_{d}^{2}\;$ for a pair of SNPS is approximately equal to the product of $\sigma_{d}^{2}\;$ for each SNP and the inversion polymorphism. The standard formula for a partial correlation coefficient implies that this is also true for the *R*^2^ statistic of Hill and Robertson (1968), if there is little or no correlation between SNPs within arrangements. If there is LD within arrangements, these products somewhat underestimate the corresponding statistics for the pairs of SNPs. If there is a major effect of divergence between arrangements on LD between SNPs and karyotype, there should be little dependence of $\sigma_{d}^{2}\;$ or *R*^2^ on the physical distance between SNPs across the region where crossing over is suppressed by the inversion, in contrast to what is expected for within-karyotype patterns of LD.

Supplementary Fig. 3 of Kapun *et al*. (2023) shows a pattern of elevated *R*^2^ among SNPs across the entire region of *In*(*3R*)*P* in the Zambian population, contrasted with the rapid decay of LD with physical distance between SNPs within inverted and standard haplotypes. As expected from the product formula, the magnitude of individual *R*^2^ values is modest in this case—the estimate of 0.033 for *F_AT_* given above gives an expected *R*^2^ of approximately 0.001 for a pair of SNPs in genomic regions affected by the inversion polymorphism. Much stronger patterns are, however, seen in the non-African samples studied by Kapun *et al*. (2023), which probably reflect the effects of population bottlenecks or recent selection. This example illustrates the point that substantial associations between SNPs and an inversion polymorphism may exist but could be hard to detect simply from LD patterns in samples without prior knowledge of the existence of the inversion polymorphism. Conversely, false signals of an inversion polymorphism could be generated from localized clusters of LD in bottlenecked populations (e.g. Haddrill *et al*. 2005). Of course, if there are virtually complete associations between SNPs and arrangements, as in the inversion polymorphisms of *Coelopa frigida* (Mérot *et al*. 2021), there will be a strong pattern of localized LD blocks (see their Fig. 2).

### Effects of inversions on neutral divergence between populations

For the case of populations with a large number of demes, the extent of population differentiation at a neutral locus associated with an inversion can be measured by *F_ST_* as defined here (which in this case is nearly the same as the < *F_ST_* > statistic of Hudson, Slatkin *et al*. 1992) for standard and inverted haplotypes, denoted here by *F_ST_*,*_St_* and *F_ST_*,*_In_*, respectively—see Equation (S23). Provided that *m* >> *r*, which is likely to be true for sites within the inversion or close to the breakpoints, the equilibrium values of *F_ST_*,*_In_* and *F_ST_*,*_St_* are equal to 1/(1 + *Mx*) and 1/(1 + *My*), respectively, which are somewhat surprisingly independent of *r* (Equations (A6h) and (A6i)). These expressions are similar in form to that for a subdivided population in the absence of a polymorphism, with *M* replaced by *Mx* and *My*. For *r* = 0, it can be shown that these values are reached almost instantaneously once the inversion has reached its equilibrium frequency (Equation S23). Numerical examples show that this is also true for *r* > 0. The rapid equilibration of *F_ST_* in subdivided populations is well known (Crow and Aoki 1984; Pannell and Charlesworth 2000).

These results imply that within-arrangement *F_ST_* or < *F_ST_* > statistics should be larger for loci within the inversion than for loci that are independent of the inversion, independently of location with respect to the inversion breakpoints. A low-frequency inversion should thus show a considerable inflation in between-population *F*-statistics, even in the absence of differences in karyotype frequencies between populations, whereas only a small effect should be seen for the standard arrangement; such a pattern was reported by Kennington and Hoffmann. (2013) for the *D. melanogaster* inversion *In(2L)t*. Caution should therefore be used in interpreting such a pattern as evidence for spatial differences in selection pressures.

In contrast, if inversion frequencies vary considerably between populations because of divergent selection, the LD between neutral sites and karyotype will cause among-population differentiation at neutral sites (Charlesworth *et al*. 1997; Guerrero *et al*. 2012), as has been found in some studies of *Drosophila* (Pegueroles *et al*. 2013) and other taxa (Faria *et al*. 2019; Mérot *et al*. 2021). If exchange rates increase with distance from breakpoints, then higher values of *F_ST_*, *_In_* and *F_ST_*, *_St_* (or the corresponding < *F_ST_* > statistics) are expected towards the centers of inversions, as has been observed in some cases, such as Inv2La in *Anopheles gambiae* (Cheng *et al.* 2012).

## Conclusions

The theoretical results described here provide pointers on how to interpret population genomic data on inversion polymorphisms, with caveats about methods for estimating the ages of inversions. They are subject to several important limitations. In particular, they are based on expectations for pairwise coalescence times. A study of the variances of pairwise diversity and divergence statistics suggests that averages taken over the many thousands of sites in the central regions of several megabase inversions that are several megabases in size have high statistical precision (see sections 8 and 9 of Supplementary File 1), but a rigorous statistical framework such as maximum likelihood inference based on the structured coalescent process (e.g. Lohse *et al*. 2011) remains to be developed. In addition, many simplifying assumptions have been made, including ignoring the consequences of selection on loci responsible for maintaining the inversion polymorphisms, as well as the effects of Hill–Robertson interference in the low recombination environment characteristic of low-frequency inversions. There is, therefore, plenty of scope for further work.

## Supplementary Material

iyad116_Supplementary_Data

## Data Availability

No new data or reagents were generated by this research. The codes for the computer programs used to produce the results described above are available in Supplementary File 2. Supplemental material available at GENETICS online.

## Appendix

### Recursion relations for expected coalescence times

The dynamics of coalescent events are determined jointly by migration, gene conversion, and genetic drift. To determine pairwise coalescent times, we need to consider cases where 2 *In* haplotypes are sampled (a sample of class 1/1), 2 *St* haplotypes are sampled (a class 2/2 sample), and 1 of each type is sampled (a class 1/2 sample). The method of Nagylaki (1998) is used for this purpose, which provides a simple way of determining expected coalescent times (see also Charlesworth and Charlesworth 2010, p.316). For 1/1 samples, the probability that the focal neutral site in 1 of the 2 haplotypes was associated with *St* in the previous generation is 2*ry* (ignoring second-order terms in *ry*), since the chance that a given *In* haplotype was combined with an *St* haplotype is *y* and either of the 2 *In* haplotypes could have experienced a recombination event. There is a probability 2*m* that 1 of the 2 haplotypes was present in a different deme from the current 1, and a probability of 1/(2*Nx*) that the 2 haplotypes coalesced in the previous generation if they were both in the same deme, given that haplotypes from different demes cannot coalesce. Similar considerations apply to 2/2 samples, except that *x* and *y* are interchanged. For 1/2 samples, there is a probability *y* that the *In* haplotype was associated with an *St* haplotype in the previous generation, so the probability that the focal neutral site in *In* derives from *St* is *ry*. Similarly, there is a probability *rx* that the focal neutral site in the *St* haplotype derives from *In*; the net probability of the focal site coming from a different karyotype from its current one is *r.* No coalescence of the 2 haplotypes is possible in this case, but there is a probability 2*m* that one of them has moved from a different deme.

By ignoring second-order terms in *m* and *r*, the following recursion relations for the mean coalescence times defined in the main text can be obtained, with primes indicating their values in the next generation:

(A1a)
$$t_{11w}^{\prime }=[1-2m-2ry]\left[\frac{1}{2Nx}+\left(1-\frac{1}{2Nx}\right)(1+t_{11w})\right]+2m(1+t_{11b})+2ry(1+t_{12w})$$

$$t_{11b}^{\prime }=[1-2m-2ry]t_{11b}+\frac{2m}{\left(d-1\right)}\left[\frac{1}{2Nx}+\left(1-\frac{1}{2Nx}\right)(1+t_{11w})\right]$$

(A1b)
$$\;+\;\frac{2m(d-2)}{\left(d-1\right)}(1+t_{11b})+2ry(1+t_{12b})$$

(A1c)
$$t_{22w}^{\prime }=[1-2m-2rx]\left[\frac{1}{2Ny}+\left(1-\frac{1}{2Ny}\right)(1+t_{22w})\right]+2m(1+t_{22b})+2rx(1+t_{12w})$$

$$t_{22b}^{\prime }=[1-2m-2rx]t_{22b}+\frac{2m}{\left(d-1\right)}\left[\frac{1}{2Ny}+\left(1-\frac{1}{2Ny}\right)(1+t_{22w})\right]$$

(A1d)
$$\;+\;\frac{2m(d-2)}{\left(d-1\right)}(1+t_{22b})+2rx(1+t_{12b})$$

(A1e)
$$t_{12w}^{\prime }=[1-2m-r][\left(1+t_{12w}\right)]+2m(1+t_{12b})+r(1+xt_{11w}+yt_{22w})\;\;\;\;\;$$

$$t_{12b}^{\prime }=[1-2m-r][\left(1+t_{12b}\right)]+\frac{2m}{\left(d-1\right)}(1+t_{12w})+\frac{2m(d-2)}{\left(d-1\right)}(1+t_{12b})\;$$

(A1f)
$$\;+\;r(1+xt_{11w}+yt_{22w})$$

These equations can be simplified by neglecting products of 1*/Nx* and 1/*Ny* with *m* and *r*, and rearranging. This yields the simplified recursion relations:

(A2a)
$$t_{11w}^{\prime }\approx 1+\left[1-\frac{1}{2Nx}-2m-2ry\right]t_{11w}+2mt_{11b}+2ryt_{12w}$$

(A2b)
$$t_{11b}^{\prime }\approx 1+\frac{2m}{\left(d-1\right)}t_{11w}+\left[1-\frac{2m}{\left(d-1\right)}-2ry\right]t_{11b}+2ryt_{12b}$$

(A2c)
$$t_{22w}^{\prime }\approx 1+\left[1-\frac{1}{2Ny}-2m-2rx\right]t_{22w}+2mt_{22b}+2rxt_{12w}$$

(A2d)
$$t_{22b}^{\prime }\approx 1+\frac{2m}{\left(d-1\right)}t_{22w}+\left[1-\frac{2m}{\left(d-1\right)}-2rx\right]t_{22b}+2rxt_{12b}$$

(A2e)
$$t_{12w}^{\prime }=1+rxt_{11w}+ryt_{22w}+[1-2m-r]t_{12w}+2mt_{12b}$$

(A2f)
$$\;t_{12b}^{\prime }=1+rxt_{11b}+ryt_{22b}+\frac{2m}{\left(d-1\right)}t_{12w}+\left[1-\frac{2m}{\left(d-1\right)}-r\right]t_{12b}$$

### Expressions for equilibrium mean coalescence times

Writing *M* = 4*Nm* and *R* = 4*Nr*, we obtain the following expressions for equilibrium, equating the *t*’s and *t*s:

(A3a)
$$\;t_{11w}(Mx+Ry+1)=2Nx+Mxt_{11b}+Rxyt_{12w}$$

(A3b)
$$\;t_{11b}[2m+2(d-1)ry]=d-1+2mt_{11w}+2(d-1)ryt_{12b}\;$$

(A3c)
$$\;t_{22w}(My+Rx+1)=2Nx+Myt_{22b}+Rxyt_{12w}$$

(A3d)
$$\;t_{22b}[2m+2(d-1)rx]=d-1+2mt_{22w}+2(d-1)rxt_{12b}$$

(A3e)
$$\;\;\;t_{12w}(2m+r)=1+2mt_{12b}+r(xt_{11w}+yt_{22w})\;\;$$

(A3f)
$$\;\;t_{12b}[2m+(d-1)r]=d-1+2mt_{12w}+(d-1)r(xt_{11b}+yt_{22b})$$

This is a set of 6 linear equations that can be solved exactly (see File S1, section 1). However, it is possible to obtain informative approximate expressions by assuming that *d* is very large, with 2*m* >> *r* but *dr* >> 2*m*, which are plausible conditions for many real-life cases. This means that the terms in *R* or *r* on the left-hand sides of Equations (A3a), (A3c), and (A3e) can be neglected, as well as the terms in *R* or *r* on the right-hand sides of Equations (A3a), (A3c), and (A3e); in addition, *d* − 1 can be replaced by *d* with negligible loss of accuracy.

Using these approximations, we have:

(A4a)
$$t_{12w}\approx (2m{)}^{-1}+t_{12b}$$

The intuitive interpretation of this expression is that, if recombination is infrequent compared with migration, a class 1/2 pair of alleles will wait an average of 1/(2*m*) generations before 1 of them migrates to a different deme, after which their expected coalescent time is *t*_12*b*_. The various *t* values are of the order of 2*N_T_* (see below), so that for large *d* and moderate *F_ST_* values, 1/*m* is small compared with the *t*'s, and the term in *m* in this equation can be neglected.

Equations (A3a) and (A3c) can then be approximated as follows:

$$\;t_{11w}(Mx+1)\approx 2Nx+Mxt_{11b}+4Nxy[1+r(xt_{11b}+yt_{22b})]$$

(A4b)
$$\approx 2Nx(1+2y)+Mxt_{11b}$$

$$\;t_{22w}(My+1)\approx 2Ny+Myt_{22b}+4Nxy[1+r(xt_{11b}+yt_{22b})]\;$$

(A4c)
$$\approx 2Ny(1+2x)+Myt_{22b}$$

From Equations (A3c) and (A4a), we obtain

$$\;t_{11b}[2m+2dry]\approx d+2mt_{11w}+2dry(r^{-1}+xt_{11b}+yt_{22b})$$

so that

$$\;t_{11b}[2m+2dry^{2}]\approx d(1+2y)+2mt_{11w}+2dry^{2}t_{22b}$$

Similarly,

$$\;t_{22b}[2m+2dryx^{2}]\approx d(1+2yx)+2mt_{22w}+2dryx^{2}t_{11b}$$

Using Equations (A4b) and (A4c), after some algebra, we obtain

(A4d)
$$t_{11b}[2m+2dry^{2}(Mx+1)]\approx d(1+2y)(Mx+1)+2dry^{2}(Mx+1)t_{22b}$$

(A4e)
$$t_{22b}[2m+2drx^{2}(My+1)]\approx d(1+2x)(My+1)+2drx^{2}(My+1)t_{11b}$$

For further work, it is convenient to scale the *t*'s by division by the expected coalescent time for a pair of neutral alleles sampled from the same deme, *T_Sn_* = 2*N_T_* = 2*dN*. Times on this scale are denoted by uppercase *T*'s. The corresponding scaled recombination rate is denoted by *ρ* = 4*N_T_r*, which replaces 2*r* in Equations (A4d) and (A4e) when using the rescaled *t*'s; 2*m* is also replaced by *dM*, and *d* can be cancelled from both sides of these equations, which then give a pair of linear equations in *T*_11*b*_ and *T*_22*b*_. After some further algebra, their solution can be written in the standard form for a pair of linear equations as follows:

(A5a)
$$T_{11b}\approx (a_{22}b_{1}-a_{12}b_{2})/det,T_{22b}\approx (a_{11}b_{2}-a_{21}b_{1})/det$$

(A5b)
$$det=(a_{11}a_{22}-a_{12}a_{21})$$

(A5c)
$$\;a_{11}=M(Mx+1{)}^{-1}+\rho y^{2},\;a_{12}=-\rho y^{2},\;a_{21}=-\rho x^{2},\;\;a_{22}=M(My+1{)}^{-1}+\rho x^{2}\;\;$$

(A5d)
$$b_{1}=(1+2y),\;\;\;\;\;b_{2}=(1+2x)\;\;$$

Use of the expressions for the *a_ij_* yields the following formulae:

(A6a)
$$det=M\{M(Mx+1{)}^{-1}+M(My+1{)}^{-1}+\;\rho [x^{2}(Mx+1{)}^{-1}+y^{2}(My+1{)}^{-1}]\}$$

(A6b)
$$\;T_{11b}\approx [(1+2y)M{\left(My+1\right)}^{-1}+\rho ]/det$$

(A6c)
$$T_{22b}\approx [(1+2x)M{\left(Mx+1\right)}^{-1}+\rho ]/det$$

These expressions can be substituted into the scaled versions of Equations (A4d)–(A4e) to obtain the complete approximate solution to the set of equations for the *T*'s:

(A6d)
$$\;T_{12b}\approx \;2\rho^{-1}+xT_{11b}+yT_{22b}$$

(A6e)
$$\;T_{12w}\approx T_{12b}$$

(A6f)
$$\;T_{11w}(Mx+1)\approx d^{-1}x(1+2y)+MxT_{11b}$$

(A6g)
$$T_{22w}(My+1)\approx d^{-1}y(1+2x)+MyT_{22b}$$

If *d* >> 1, the terms in *d* in Equations (A6f) and (A6g) can be neglected, so that

(A6h)
$$\;T_{11w}\approx Mx(1+Mx{)}^{-1}T_{11b}$$

(A6i)
$$\;T_{22w}\approx My(1+My{)}^{-1}T_{22b}$$

For calculating the approach to the equilibrium described by Equation (A6), it is convenient to eliminate the terms in 1 on the right-hand sides of Equation (A2) by using the vector of deviations of the *T*'s from their equilibrium values, and applying a matrix ***A*** that contains the multipliers of the *T*'s in Equation (A2). Details are given in File S1, section 7.
