# Supplementary material for: The effects of inversion polymorphisms on patterns of neutral genetic diversity
Source: Genetics. 2023 Jun 22;224(4):iyad116. doi: 10.1093/genetics/iyad116 (PMC10411593; doi:10.1093/genetics/iyad116)
Supplement: iyad116_Supplementary_Data [file iyad116_supplementary_data.zip › File_S1_GENETICS-2023-306220.pdf]

File S1

S1 The exact equilibrium solution with population subdivision

The full solution for the equilibrium T's can be found from Equations (A3) as follows, considering each row in turn and after dividing both sides by $2N_T$.

$$T_{11w} = c_0 + c_1T_{11b} + c_2T_{12w}, c_0 = \frac{x}{dg_1}, \; c_1 = \frac{Mx}{g_1}, c_2 = \frac{Rxy}{g_1}, \; g_1 = Mx + Ry + 1 \quad \text{(S1a)}$$

$$T_{11b} = c_3 + c_4T_{12w} + c_5T_{12b}, c_3 = \frac{[(1-d^{-1}) + c_0M]}{g_2}, c_4 = \frac{c_2M}{g_2}, c_2 = \frac{(\rho - R)y}{g_1},$$

$$g_2 = M(1 - c_1) + (\rho - R)y \quad \text{(S1b)}$$

$$T_{22w} = c_6 + c_7T_{22b} + c_8T_{12w}, c_6 = \frac{y}{dg_1}, c_7 = \frac{Mx}{g_1}, c_8 = \frac{Rxy}{g_3}, g_3 = My + Rx + 1 \quad \text{(S1c)}$$

$$T_{22b} = c_9 + c_{10}T_{12w} + c_{11}T_{12b}, c_9 = \frac{[(d-1)d^{-1} + c_6M]}{g_4}, c_{10} = \frac{c_8M}{g_4}, c_{11} = \frac{(\rho - R)x}{g_4},$$

$$g_4 = M(1 - c_7) + (\rho - R)x \quad \text{(S1d)}$$

These equations can be used to eliminate all the T's other than T_{12w} and T_{12b} from Equations (A1e) and (A1f), yielding a pair of linear equations:

$$b_{11}T_{12w} + b_{12}T_{12b} = e_1, \qquad b_{21}T_{12w} + b_{22}T_{12b} = e_2 \quad \text{(S1e)}$$

where

$$e_1 = d^{-1} + \tfrac{1}{2}R[x(c_0 + c_1c_3) + y(c_6 + c_2c_9)], \; e_2 = 1 - d^{-1} + \tfrac{1}{2}\rho(xc_3 + yc_9)$$

$$b_{11} = M + \tfrac{1}{2}R[1 - x(c_2 + c_1c_4) - y(c_8 + c_7c_{11})], \; b_{12} = -\tfrac{1}{2}R[xc_1c_5 + yc_7c_{11}]$$

$$b_{21} = -[M + \tfrac{1}{2}\rho(xc_4 + yc_{10})], b_{22} = M + \tfrac{1}{2}\rho(1 - xc_5 - yc_{11})$$

This set of equations has the following standard form solution:

$$T_{12w} \approx (b_{22}e_1 - b_{12}e_2)/det, \quad T_{12b} \approx (b_{11}e_2 - b_{21}e_1)/det \quad \text{(S1f)}$$

$$det = (b_{11}b_{22} - b_{12}b_{21}) \quad \text{(S1g)}$$

S2 The approach to the approximate equilibrium solutions

Let the column vector $\boldsymbol{x} = (\delta T_{11w}, \delta T_{11b}, \delta T_{22w}, \delta T_{22b}, \delta T_{12w}, \delta T_{12b})^T$, where δ denotes the deviation from the equilibrium value of the element of the vector in question. Let $N_1 = 2Nx$, and $N_2 = 2Ny$.

With large d, the 6 x 6 matrix $\boldsymbol{A}$ that describes the approach of $\boldsymbol{x}$ to a vector of zero elements, by iterating $\boldsymbol{x}_n = \boldsymbol{A}\boldsymbol{x}_{n\text{-}1}$, consists of the following elements:

$1–2m–2ry–1/N_1$	$2m$	0	0	$2ry$	0
$2m/d$	$1–2m/d–2ry$	0	0	0	$2ry$
0	0	$1–2m–2rx–1/N_2$	$2m$	$2rx$	0
0	0	$2m/d$	$1–2m/d–2rx$	0	$2rx$
rx	0	ry	0	$1–2m–r$	$2m$
0	rx	0	ry	$2m/d$	$1–2m/d–r$

S3 Approximations for the eigenvalues and eigenvectors of *A*

An approximation for the set of eigenvalues λ_i (i = 1 – 6) of $\boldsymbol{A}$ can be obtained as follows, by noting that all its elements a_{ij} other than the diagonals a_{ii} are zero or of the order of m or r, so that second-order terms in non-diagonal elements can be neglected. Ignoring the zero elements in the first row of $\boldsymbol{A}$, the characteristic equation of $\boldsymbol{A}$, det($\boldsymbol{A} - \lambda\boldsymbol{I}$), can be expanded as $(a_{11} - \lambda)A_{11} - a_{12}A_{12} + a_{15}A_{15}$, where A_{ij} denotes the minor of element a_{ij}.

Similarly, $A_{11} = (a_{22} - \lambda)B_{22} - a_{34}B_{34} + a_{35}B_{35}$, where B_{ij} is the minor of a_{ij} in the submatrix corresponding to the determinant A_{11}. Writing $O(\varepsilon)$ for terms of order m or r, and repeating this procedure on the B_{ij}, it is easily seen that $B_{22} = (a_{22} - \lambda)(a_{33} - \lambda)(a_{44} - \lambda)(a_{55} - \lambda)(a_{66} - \lambda)$, $B_{34} = O(\varepsilon)$, and $B_{35} = O(\varepsilon^2)$. Since $a_{34} = O(\varepsilon)$, the only significant term in $(a_{11} - \lambda)A_{11}$ is thus $(a_{11} - \lambda)(a_{22} - \lambda)(a_{33} - \lambda)(a_{44} - \lambda)(a_{55} - \lambda)(a_{66} - \lambda)$.

We also have $A_{12} = a_{21}C_{21} - a_{26}C_{26}$, where C_{ij} is the minor of a_{ij} in the submatrix corresponding to the determinant A_{34}. Since both a_{21} and a_{26} are $O(\varepsilon)$, their product with a_{12} can be neglected. Similarly, A_{15} can be expanded as $a_{21}D_{21} - a_{22}D_{22} + a_{26}D_{26}$; the products of $a_{21}D_{21}$ and $a_{26}D_{22}$ with a_{15} can thus be neglected. D_{22} can in turn be expanded as $a_{33}E_{33} + a_{34}E_{34}$ where $E_{33} = 0$ and $E_{34} = O(\varepsilon)$.

The final result, therefore, is that det($\boldsymbol{A} - \lambda\boldsymbol{I}$) = $\Pi_i (a_{ii} - \lambda) + O(\varepsilon^2)$. This means that the six eigenvalues of $\boldsymbol{A}$ can be equated to the corresponding diagonal elements, which are of order 1 – $O(\varepsilon)$.

This in turn allows approximations for the right and left eigenvectors of $\boldsymbol{A}$, and hence its spectral expansion, to be determined, giving a complete (but approximate) analytic solution to the trajectory of deviations of the t's from the equilibrium values. The relevant components of

these vectors can be obtained from the equations $\boldsymbol{A}z_i = \lambda_i z_i$ and $\boldsymbol{y}_i\boldsymbol{A} = \lambda_i \boldsymbol{y}_i$, where z and $\boldsymbol{y}$ refer to right and left eigenvectors, respectively, by successive elimination of elements of the vectors.

Using a similar procedure to that leading to Equation (S1), by considering only the non-zero elements of $\boldsymbol{A}$, examining each row $\boldsymbol{A}$ of in succession and writing $\lambda = 1 - \alpha$ for a given λ, we have:

$$z_1 = k_{12}z_2 + k_{15}z_2, \quad k_{12} = \frac{a_{12}}{1-\alpha-a_{11}}, \quad k_{15} = \frac{a_{15}}{1-\alpha-a_{11}} \tag{S2a}$$

$$z_2 = k_{25}z_5 + k_{26}z_6, \quad k_{25} = \frac{a_{21}k_{15}}{1-\alpha-a_{22}-a_{21}k_{12}}, \quad k_{26} = \frac{k_{26}}{1-\alpha-a_{22}-a_{21}k_{12}} \tag{S2b}$$

$$z_3 = k_{34}z_4 + k_{35}z_5, \quad k_{34} = \frac{a_{34}}{1-\alpha-a_{33}}, \quad k_{35} = \frac{a_{35}}{1-\alpha-a_{33}} \tag{S2c}$$

$$z_4 = k_{45}z_5 + k_{46}z_{56}, \quad k_{45} = \frac{a_{43}k_{35}}{1-\alpha-a_{44}-a_{43}k_{34}}, \quad k_{46} = \frac{a_{46}}{1-\alpha-a_{44}-a_{43}k_{34}} \tag{S2d}$$

$$z_5 = k_{56}z_6, \quad k_{56} = \frac{1-\alpha-a_{66}-a_{62}k_{26}-a_{64}k_{46}}{a_{62}k_{25}+a_{64}k_{45}+a_{65}} \tag{S2e}$$

All elements of z can thus be evaluated explicitly as ratios with respect to z_6. The same procedure can be carried out for the components of the left eigenvector, $\boldsymbol{y}$; the coefficients corresponding to the k_{ij} above are identical in form, except that the elements of the transpose of $\boldsymbol{A}$ are used. Constructing the matrices $\boldsymbol{Z}$ and $\boldsymbol{Y}$, whose columns and rows respectively are given by the sets of column vectors z_i and row vectors $\boldsymbol{y}_i$ corresponding to each eigenvalue, we have the standard result $\boldsymbol{A}^n = \boldsymbol{Z}\boldsymbol{\Lambda}^n\boldsymbol{Y}$, where $\boldsymbol{\Lambda}$ is the diagonal matrix whose non-zero elements are equal to the eigenvalues of $\boldsymbol{A}$, and the eigenvectors are normalized such that the product $\boldsymbol{y}_i z_i = 1$.

Similar results can be obtained for the panmictic case, using rows and columns 1, 3 and 5 of $\boldsymbol{A}$, with $m = 0$.

S4 Approximations for the initial per generation changes in the F_{AT}'s

The first-order approximation for ΔF_{ATw} is as follows:

$$\Delta F_{ATw} \approx \Delta T_{11w}\partial_{T_{11w}}F_{ATw} + \Delta T_{22w}\partial_{T_{22w}}F_{ATw} + \Delta T_{12w}\partial_{T_{12w}}F_{ATw} \tag{S3a}$$

Here, the derivatives are evaluated at the initial conditions: $T_{22w} = T_{22b} = 0$, $T_{12w} = T_{22w} = 1$, and $T_{22b} = T_{12b} \approx 1/(1 - F_{ST})$. Using the relation $F_{ATw} = 1 - T_{Sw}/T_{Tw}$, and the definitions of T_{Sw} and T_{Tw} given in the main text (Equations 1 - 4), we obtain the following expressions:

$$\partial_{T_{11w}} T_{Sw} = x,\ \partial_{T_{22w}} T_{Sw} = y,\ \partial_{T_{12w}} T_{Sw} = 0; \partial_{T_{11w}} T_{Tw} = y^2; \partial_{T_{22w}} T_{Tw} = yx^2; \partial_{T_{12w}} T_{Tw} = 2xy \quad \text{(S3b)}$$

Substituting these relations into Equations (6) and (S3), and using the fact that the derivative of $F_{ATw} = 1 - T_{Sw}/T_{Tw}$ with respect to an arbitrary variable u is $\frac{T_S \partial_u T_{Tw} - T_{Tw} \partial_u T_{Sw}}{T_T^2}$, yields Equation (7a), after some simplification.

A similar procedure can be used for F_{ATb} noting that terms involving the derivative of T_{22b} can be ignored, because $\Delta T_{22b} \approx 0$ by Equation (6d). It is useful to note that the initial value of T_{Tb} can be written as $(1 - F_{ST})^{-1} y[1 + x(1 - F_{ST})]$. We have:

$$\partial_{T_{11b}} T_{Sb} = x,\ \partial_{T_{11b}} T_{Sb} = y;\ \partial_{T_{11b}} T_{Tb} = y^2; \partial_{T_{12b}} T_{Tb} = 2xy(1 - F_{ST})^{-1} \quad \text{(S3c)}$$

This yields Equation (7b).

S5 Inaccuracy in the Approximate Expressions for Equilibrium

Denote the true equilibrium vector of *T's* by $\tilde{\boldsymbol{x}} = \boldsymbol{x}^* - \boldsymbol{\delta}$, where $\boldsymbol{x}^*$ is the approximate value. Let the true value of $\boldsymbol{x}$ in generation n be $\boldsymbol{x}_n$, where $n = 0$ for the initial generation under consideration. Let $\boldsymbol{A}$ be the true value of the recursion matrix discussed above. The true equilibrium satisfies $\boldsymbol{A}\tilde{\boldsymbol{x}} + \boldsymbol{I} = \tilde{\boldsymbol{x}}$, where $\boldsymbol{I}$ is the unit matrix. For generation 1, we can write:

$$\boldsymbol{x}_1 - \boldsymbol{x}^* = \boldsymbol{x}_1 - \tilde{\boldsymbol{x}} + \boldsymbol{\delta} = \boldsymbol{A}\boldsymbol{x}_0 + \boldsymbol{I} - (\boldsymbol{A}\tilde{\boldsymbol{x}} + \boldsymbol{I}) - \boldsymbol{\delta} = \boldsymbol{A}[\boldsymbol{x}_0 - (\tilde{\boldsymbol{x}} + \boldsymbol{\delta})] + \boldsymbol{\delta}(\boldsymbol{A} - \boldsymbol{I}) \quad \text{(S4a)}$$

In the next generation,

$$\begin{aligned}\boldsymbol{x}_2 - \boldsymbol{x}^* &= \boldsymbol{A}\boldsymbol{x}_1 + \boldsymbol{I} - (\boldsymbol{A}\tilde{\boldsymbol{x}} + \boldsymbol{I}) - \boldsymbol{\delta} = \boldsymbol{A}\{\boldsymbol{A}[\boldsymbol{x}_0 - (\tilde{\boldsymbol{x}} + \boldsymbol{\delta})] + \boldsymbol{\delta}(\boldsymbol{A} - \boldsymbol{I})\} + \boldsymbol{\delta}(\boldsymbol{A} - \boldsymbol{I}) = \\ &= \boldsymbol{A}^2[\boldsymbol{x}_0 - (\tilde{\boldsymbol{x}} + \boldsymbol{\delta})] + (\boldsymbol{A} + \boldsymbol{I})\boldsymbol{\delta}(\boldsymbol{A} - \boldsymbol{I}) \quad \text{(S4b)}\end{aligned}$$

Continuing with the iteration, the general expression is:

$$\boldsymbol{x}_n - \boldsymbol{x}^* = \boldsymbol{A}^n[\boldsymbol{x}_0 - (\widetilde{\boldsymbol{x}} + \boldsymbol{\delta})] + (\boldsymbol{A}^{n-1} + \boldsymbol{A}^{n-2} + \cdots + \boldsymbol{I})\boldsymbol{\delta}(\boldsymbol{A} - \boldsymbol{I}) \qquad \text{(S4c)}$$

From the analysis in section S3, the term in $\boldsymbol{A}^n$ must tend to zero as n increases, at least as far as first-order terms are concerned, so only the series in $\boldsymbol{A}$ need be considered. If we write $\boldsymbol{P}_i$ for the matrix formed by the vector product $z_i\boldsymbol{y}_i$, such that $\boldsymbol{A}^j = \sum_i \lambda_i^j \boldsymbol{P}_i$, for large n this term approaches $\sum_i \frac{1}{(1-\lambda_i)} \boldsymbol{P}_i$, provided that $|\lambda_i| < 1$, as must be the case from the results in section 3. This implies that the iteration of $\boldsymbol{x} - \boldsymbol{x}^*$ by the matrix $\boldsymbol{A}$, as used in the numerical studies of the approach to equilibrium will yield a final value whose magnitude is of the same order as the error $\boldsymbol{\delta}$ in the approximate equilibrium. If this value is small, the error involved in the approximation must also be small.

S6 Time courses of changes in coalescent times in the absence of recombination in a panmictic population

In this case, there is no distinction between within- and between-deme coalescence times, and the subscripts w and b can be dropped. The mean between-karyotype coalescent time after a time T has elapsed (where both quantities are scaled relative to the neutral coalescence time $2N_T$), is simply a linear function of T:

$$T_{12}(T) = T_{12}(0) + T \qquad \text{(S5a)}$$

The mean coalescence times within-karyotypes obey the rule for the approach to their equilibrium values described in section S2 above. In this case, the relevant expressions simplify to:

$$T_{11}(T) = x + [T_{11}(0) - x]\exp(-T/x) \qquad \text{(S5b)}$$

$$T_{22}(T) = y + [T_{22}(0) - y]\exp(-T/y) \qquad \text{(S5c)}$$

If the sweep of the inversion to its equilibrium frequency is assumed to be instantaneous, we have $T_{11}(0) = 0$ and $T_{12}(0) = T_{22}(0) = 1$; we also have $T_T(0) = 2xy + y^2 = y(1 + x)$, so that $F_{AT}(0) = x/(1 + x)$. For arbitrary T, $T_S(T) = x^2[1 - \exp(-T/x)] + y[y + x\exp(-T/y)]$ and $T_T(T) = 2xy(1 + T) + x^2[1 - \exp(-T/x)] + y^2[y + x\exp(-T/y)]$.

These relations can be used to determine the time-course of F_{AT} following an instantaneous sweep, using the relation:

$$F_{AT}(T) \approx 1 - \frac{T_S(T)}{T_T(T)} \qquad \text{(S5d)}$$

For sufficiently large T, the exponential terms in the above equations can be neglected, and $T_S(T)/\, T_T(T) \approx (1 - 2xy)/(2xyT)$. This implies that, as expected, F_{AT} always increases towards 1 once a large T value has been reached. For small T, the first-order approximations $\exp(-z) \approx 1 - z$ and $1/(1 + T) \approx 1 - T$ yield the following formulae:

$$T_{11}(T) \approx T \qquad \text{(S5e)}$$

$$T_{22}(T) \approx 1 - (x/y)T \qquad \text{(S5f)}$$

$$T_S(T) \approx y \qquad \text{(S5g)}$$

$$T_T(T) \approx y(1 + x) + xT \qquad \text{(S5h)}$$

Substituting these approximations into Equation (S5d) and simplifying, we have:

$$F_{AT}(T) \approx \frac{x}{(1+x)}[1 + \frac{x}{y(1+x)}T] \qquad \text{(S5i)}$$

The multiplicand of T is always positive for $0 < x < 0$, implying that F_{AT} always increases initially with time.

S7 Time courses of changes in coalescent times in the absence of recombination in a subdivided population

With no recombination, the recursion relations for T_{12w} and T_{12b}, which can be obtained from Equations (A3) reduce to:

$$\boldsymbol{X}(t) = \boldsymbol{c} + \boldsymbol{A}\,\boldsymbol{X}(t - 1) \qquad \text{(S6a)}$$

where $\boldsymbol{X}(t)$ is the column vector with elements $T_{12w}(t)$ and $T_{12b}(t)$; $\boldsymbol{c}$ is the column vector whose elements are both equal to $1/(2N_T)$; $\boldsymbol{A}$ is the 2 x 2 matrix with elements $a_{11} = 1 - 2m$, $a_{12} = 1 - 2m$, $a_{21} = 2m/(d - 1)$ and $a_{22} = 1 - 2m/(d - 1)$.

Iteration of this equation yields the following expression:

$$\boldsymbol{X}(t) = \boldsymbol{A}^t\boldsymbol{X}(0) + \sum_{i=1}^{t} \boldsymbol{A}^{t-i}\, \boldsymbol{c} \qquad \text{(S6b)}$$

The powers of $\boldsymbol{A}$ can be evaluated using the spectral expansion of $\boldsymbol{A}$ in terms of its eigenvalues and eigenvectors. The eigenvalues of $\boldsymbol{A}$ satisfy the following characteristic equation:

$$\lambda^2 - \lambda[2\text{–}\, 2m(1 + d'^{-1}) + 1 - 2m(1 + d'^{-1}) \qquad \text{(S7)}$$

where for convenience $d - 1$ is denoted by d'.

This equation has roots $\lambda_0 = 1$ and $\lambda_1 = 1 - 2m(1 + d'^{-1})$, with corresponding right and left eigenvectors $\boldsymbol{u}_i$ and $\boldsymbol{v}_i$, respectively ($i = 0, 1$). Use of the identities $\boldsymbol{A}\boldsymbol{u}_i = \lambda_i\boldsymbol{u}_i$ and $\boldsymbol{v}_i\boldsymbol{A} = \lambda_i\boldsymbol{v}_i$ give the result $u_{01} = u_{02}$ for the 1st and 2nd elements of $\boldsymbol{u}_0$, respectively. Similarly, $u_{11} = (-d')\, u_{12}$, $v_{01} = v_{02}/d$ and $v_{11} = -v_{12}$. In order to use these in the spectral expansion of $\boldsymbol{A}$, we arbitrarily set $u_{01} = u_{02} = 1$ and $u_{11} = 1$, with $u_{12} = -\, d'^{-1}$, and use the normalization requirement $\boldsymbol{u}_i^{Tr}\boldsymbol{v}_i = 1$, where the superscript Tr denotes transposing a column vector into a row vector. This gives $v_{01} = (1 + d')^{-1}$, $v_{02} = v_{11} = (1 + d'^{-1})^{-1}$, $v_{12} = -\,(1 + d'^{-1})^{-1}$. Using the formula for the spectral decomposition of a matrix, $\boldsymbol{A} = \sum_i \lambda_i \boldsymbol{u}_i\, \boldsymbol{v}_i$, we obtain:

$$\boldsymbol{A}^t = \begin{pmatrix} (1+d')^{-1} & \left(1+d'^{-1}\right)^{-1} \\ (1+d')^{-1} & \left(1+d'^{-1}\right)^{-1} \end{pmatrix} + [1-2m(1+d'^{-1})]^t \begin{pmatrix} \left(1+d'^{-1}\right)^{-1} & -\left(1+d'^{-1}\right)^{-1} \\ -d'^{-1}\left(1+d'^{-1}\right)^{-1} & d'^{-1}\left(1+d'^{-1}\right)^{-1} \end{pmatrix} \qquad \text{(S8a)}$$

For large d, this expression reduces to:

$$\boldsymbol{A}^t \approx \begin{pmatrix} 0 & 1 \\ 0 & 1 \end{pmatrix} + [1-2m)]^t \begin{pmatrix} 1 & -1 \\ 0 & 0 \end{pmatrix} = \begin{pmatrix} (1-2m)^t & 1-(1-2m)^t \\ 0 & 1 \end{pmatrix} \qquad \text{(S8b)}$$

This equation implies that:

$$\sum_{i=1}^{t} \boldsymbol{A}^{t-i} \approx \begin{pmatrix} \frac{1-(1-2m)^t}{2m} & t - \frac{1-(1-2m)^t}{2m} \\ 0 & t \end{pmatrix} \qquad \text{(S8c)}$$

If time is transformed to the coalescent timescale, $T = t/(2N_T)$, $(1 - 2m)^t$ can be approximated by $\exp(-\, MdT)$. On the assumption of an instantaneous approach of the inversion to its equilibrium frequency, so that the initial vector $\boldsymbol{X}(0) = (1, (1 - F_{ST})^{-1})^{Tr} = (1, (1{+}M)/M)^{Tr}$ Equations (S6b), (S8b) and (S8c) yield the following expression:

$$\boldsymbol{X}(T) \approx \begin{pmatrix} \exp(-dMT) & 1-\exp(-dMT) \\ 0 & 1 \end{pmatrix} (1,\ (1-F_{ST})^{-1})^{Tr} + \begin{pmatrix} \frac{[1-\exp(-dMT)]}{2m} & (2N_T T) - \frac{[1-\exp(-dMT)]}{2m} \\ 0 & 2N_T T \end{pmatrix} \boldsymbol{c} \qquad \text{(S9a)}$$

which reduces to:

$$\boldsymbol{X}(T) \approx \ \exp^{-dMT} + (1-\exp^{-dMT})(1-F_{ST})^{-1} + T,\ (1-F_{ST})^{-1} + T\ )^{Tr} \qquad \text{(S9b)}$$

When $dMT >> 1$, this expression implies that both T_{12w} and $T_{12\mathrm{b}} \approx 1/(1-F_{ST}) + T \approx (1+M)/M + T$. For $dMT << 1$, T_{12w} increases faster with T than does T_{12b}, which always increases in direct proportion to T, especially when M is small. This can be seen by using the first-order approximation $\exp(-z) \approx 1-z$ in Equation (S9b), which yields:

$$T_{12w} \approx 1 - MT + dMT(1-F_{ST})^{-1} + T = 1 + (d+1)T \qquad \text{(S9c)}$$

$$T_{12b} \approx \frac{(1+M)}{M} + T \qquad \text{(S9d)}$$

The deviations of $T_{11w}(t)$ and $T_{11b}(t)$ from their equilibrium values, $T_{11w} = x$ and $T_{11\,b} = x/(1-F_{ST,\,In})$, where $F_{ST,\,In}$ = is the value of F_{ST} for the inversion subpopulation are described by the following matrix equation:

$$\boldsymbol{Y}(t) = \boldsymbol{B}^t\, \boldsymbol{Y}(0) \qquad \text{(S10a)}$$

where $\boldsymbol{Y}(t)$ is the column vector $(\delta T_{11w}(t), \delta T_{11\mathrm{b}}(t))^{Tr}$. We have:

$$\boldsymbol{B} = \begin{pmatrix} 1-\frac{(1+Mx)}{2Nx} & 2m \\ \frac{2m}{d'} & 1-\frac{2m}{d'} \end{pmatrix} \qquad \text{(S10b)}$$

The characteristic equation of $\boldsymbol{B}$ can be written as:

$$\lambda^2 - \lambda\left[2-\frac{(1+Mx)}{2Nx} - 2md'^{-1}\right] + 1 + \frac{m}{Nd'x} - \frac{(1+Mx)}{2Nx} - 2md'^{-1} = 0 \qquad \text{(S11)}$$

This equation has the following roots:

$$\lambda_0 = \frac{1}{2}\{2-\frac{(1+Mx)}{2Nx} - 2md'^{-1} + \sqrt{\left[\frac{(1+Mx)}{2Nx} + \frac{2m}{d'}\right]^2 - \frac{4m}{Nd'x}}\ \} \qquad \text{(S12a)}$$

$$\lambda_1 = \frac{1}{2}\{2-\frac{(1+Mx)}{2Nx} - 2md'^{-1} - \sqrt{\left[\frac{(1+Mx)}{2Nx} + \frac{2m}{d'}\right]^2 - \frac{4m}{Nd'x}}\ \} \qquad \text{(S12b)}$$

The quantity inside the square term can be written as:

$$\left[\frac{(1+Mx)}{2Nx} + \frac{2m}{d'}\right]^2 (1-\varepsilon) \qquad \text{(S13a)}$$

where $$\varepsilon = \frac{4m}{Nd'x}\left[\frac{(1+Mx)}{2Nx} + \frac{2m}{d'}\right]^{-2} \qquad \text{(S13b)}$$

Unless Nx is close to 1, $\varepsilon << 1$, and $\sqrt{1-\varepsilon} \approx 1 - \frac{1}{2}\varepsilon$, so that the eigenvalues can be approximated by:

$$\lambda_0 \approx 1-\frac{m}{Nd'x}\left[\frac{(1+Mx)}{2Nx} + \frac{2m}{d'}\right]^{-1} = 1-\alpha \qquad \text{(S14a)}$$

$$\lambda_1 \approx 1-\frac{(1+Mx)}{2Nx} - \frac{2m}{d'} + \frac{m}{Nd'x}\left[\frac{(1+Mx)}{2Nx} + \frac{2m}{d'}\right]^{-1} = 1 - \frac{(1+Mx)}{2Nx} - \frac{2m}{d'} + \alpha \qquad \text{(S14b)}$$

where

$$\alpha = \frac{m}{Nd'x}\{\frac{[1+M(x+d'^{-1})]}{2Nx}\}^{-1} = \frac{M}{2Nd'}[1+M(x+d'^{-1})]^{-1} \qquad \text{(S14c)}$$

For large d, we have:

$$\alpha \approx \frac{M}{2N_T(1+Mx)} \qquad \text{(S14d)}$$

This expression implies that the asymptotic rate of approach to equilibrium on the coalescent timescale of $2N_T$ generations is approximately $M/(1 + Mx)$.

The elements of the corresponding right and left eigenvectors, $\boldsymbol{u}'_i$ and $\boldsymbol{v}'_i$, are given by the following expressions:

$$\frac{u'_{02}}{u'_{01}} = (2m)^{-1}[\frac{(1+Mx)}{2Nx} - \alpha] \approx \frac{(1+Mx)}{Mx} \qquad \text{(S15a)}$$

$$\frac{u'_{12}}{u'_{11}} = -(2m)^{-1}(\frac{2m}{d'} - \alpha) \approx -\,d'^{-1} \qquad \text{(S15b)}$$

$$\frac{v'_{02}}{v'_{01}} = d'(2m)^{-1}[\frac{(1+Mx)}{2Nx} - \alpha] \approx d'\,\frac{(1+Mx)}{Mx} \qquad \text{(S15c)}$$

$$\frac{v'_{12}}{v'_{11}} = -d'(2m)^{-1})(\frac{2m}{d'} - \alpha) \approx -1 \quad \text{(S15d)}$$

As before, we can arbitrarily set $u'_{01} = u'_{11} = 1$, and use the normalizations $\boldsymbol{u}_i'^{Tr}\boldsymbol{v}'_i = 1$ to obtain the following relations:

$$u'_{02} = (2m)^{-1}[\frac{(1+Mx)}{2Nx} - \alpha] \approx \frac{(1+Mx)}{Mx} \quad \text{(S16a)}$$

$$v'_{01} = \{1 + d'[(1 + Mx)(Mx)^{-1}]^2\}^{-1} \quad \text{(S16b)}$$

$$v'_{02} = d'(2m)^{-1}[\frac{(1+Mx)}{2Nx} - \alpha]\{1 + d'[(1 + Mx)(Mx)^{-1}]^2\}^{-1}$$

$$\approx d'\frac{(1+Mx)}{Mx}\{1 + d'[(1 + Mx)(Mx)^{-1}]^2\}^{-1} \quad \text{(S16c)}$$

$$u'_{12} = -(2m)^{-1}(\frac{2m}{d'} - \alpha) \approx -d'^{-1} \quad \text{(S16d)}$$

$$v'_{11} = [1 + d'(2m)^{-2}(\frac{2m}{d'} - \alpha)^2]^{-1} \approx (1 + d'^{-1})^{-1}$$

$$v'_{12} = -v'_{02} \quad \text{(S16e)}$$

The spectral decomposition of the power of a matrix can be used to write $\boldsymbol{B^t} = \sum_i \lambda_i^t \boldsymbol{u}'_i \boldsymbol{v}'_i$. This can be substituted into Equation (S10a) to obtain $T_{11w}(t)$ and $T_{11b}(t)$. When d is large, Equations (S16) yield:

$$\boldsymbol{B}^t \approx \begin{pmatrix} 0 & \frac{Mx}{(1+Mx)} \\ 0 & 1 \end{pmatrix}(1-\alpha)^t + [1 - \frac{(1+Mx)}{2Nx}]^t \begin{pmatrix} 1 & -\frac{Mx}{(1+Mx)} \\ 0 & 0 \end{pmatrix}$$

$$= \begin{pmatrix} \left[1 - \frac{(1+Mx)}{2Nx}\right]^t & \frac{Mx}{(1+Mx)}\{(1-\alpha)^t - \left[1 - \frac{(1+Mx)}{2Nx}\right]^t\} \\ 0 & (1-\alpha)^t \end{pmatrix}$$

$$\approx \begin{pmatrix} \exp[-\frac{(1+Mx)t)}{2Nx}] & \frac{Mx}{(1+Mx)}\{\exp(-\alpha t) - \exp[-\frac{(1+Mx)t}{2Nx}]\} \\ 0 & \exp(-\alpha t) \end{pmatrix} \quad \text{(S17a)}$$

If time is measured in units of coalescent time (T), the last expression can be written as:

$$\boldsymbol{B}^T \approx \begin{pmatrix} \exp[-\frac{d(1+Mx)T}{x}] & \frac{Mx}{(1+Mx)}\{\exp -[\frac{MT}{(1+Mx)}] - \exp[-\frac{d(1+Mx)T}{x}]\} \\ 0 & \exp[-\frac{MT}{(1+Mx)}] \end{pmatrix} \quad \text{(S17b)}$$

The terms involving exp [– $d(1 + Mx)T/x$] decay much faster than those involving exp [– $MT/(1 + Mx)$], unless d/x is of order l or less, and can therefore be neglected when d is large, except when T is of order $1/d$, i.e., for early stages in the process. For large d, the equilibrium values of T_{11w} and T_{11b} are x and approximately $(1 + Mx)/M$, respectively; Equation (S17b) implies that, for $dT >> x/(1 + Mx)$, we have:

$$T_{11w}(T) \approx x + [T_{11b}(0) - \frac{(1+Mx)}{M}]\frac{Mx}{(1+Mx)}\exp[-\frac{MT}{(1+Mx)}] \quad \text{(S18a)}$$

$$T_{11b}(T) \approx \frac{(1+Mx)}{M} + [T_{11b}(0) - \frac{(1+Mx)}{M}]\exp[-\frac{MT}{(1+Mx)}] \quad \text{(S18b)}$$

where $T_{11w}(0) = T_{11b}(0) = 0$ in the case of an instantaneous approach of the inversion to its equilibrium frequency. In this case, Equation (S18a) has the same form as Equation (S5b) for the panmictic case, except that $(1 + Mx)/M$ replaces x in the exponential function. This corresponds to the replacement of the migration effective population size, N_T, which determines the expected coalescent time for pairs of allele sampled within a deme, by the "total effective population size", $N_T/(1 - F_{ST})$, which determines the expected coalescence time for pairs of alleles sample randomly from the whole population {Charlesworth, 2010 #3373}, p.318. Since $x < (1 + Mx)/M$, this corresponds to a slower asymptotic rate of approach to equilibrium in the case of a subdivided population, with the rate being an increasing function of M.

A similar treatment of changes in mean coalescent times can be applied to the *St* subpopulation by replacing $T_{11w}(t)$ and $T_{11b}(T)$ with $T_{22w}(T)$ and $T_{22b}(T)$, and x with y, setting $T_{22w}(0) = 1$ and $T_{11b}(0) = (1 + My)/M$ in the case of an instantaneous approach of the inversion to its equilibrium frequency.

The relevance of these asymptotic rates of change is not, however, clear, since the rates of change caused by the smaller eigenvalue are substantial when d is large, as assumed here, so that the equilibrium values of the T_{iiw} and T_{iib} ($i = 1$ or 2) may be approached quite early on. The properties of the early stages of the process can be examined by approximating the exponentials in Equation (S17), and its equivalent for T_{22w} and T_{22b}, by the first-order terms in the relevant exponential functions, as was done to obtain Equations (S5) for the panmictic case in section S5. But it is important to note that this approximation requires $dT(1+Mx)/x << 1$in Equation (S17b), so that it will be accurate only for $T << x/d(1+Mx)$, a brief period of time.

Applying the initial conditions $\delta T_{11w}(0) = -x$, $\delta T_{11b}(0) = -(1 + Mx)/M$, $\delta T_{22w}(0) = x$ and $\delta T_{22b}(0) = (1+M)M^{-1} - (1+My)M^{-1} = x$ to these equations, some simple algebra yields the following expressions for the case of an instantaneous sweep:

$$T_{11w}(T) \approx \frac{Mx}{(1+Mx)}T \tag{S19a}$$

$$T_{11b}(T) \approx T \tag{S19b}$$

$$T_{22w}(T) \approx 1 - (dxy^{-1})T \tag{S19c}$$

$$T_{22b}(T) \approx \frac{(1+M)}{M} - \frac{Mx}{(1+My)}T \tag{S19d}$$

Equation (S19b) shows that T_{11b} is initially proportional to T, and Equation (S19a) shows that T_{11w} increases with time at a rate that is a fraction $Mx/(1 + Mx)$ of T. Equation (S19c) shows that T_{22w} decreases rapidly with time when d is large, due to the dominance of the relevant term in d in the relevant recursion equation; this means that the decline in T_{22w} dominates the expressions for T_{Sw} and T_{Tw}. These expressions yield the following results for the mean within-karyotype and overall coalescent times, assuming that d is large and $dMT << 1$:

$$T_{Sw}(T) \approx y - dxT \tag{S20a}$$

$$T_{Tw}(T) \approx y(1 + x) - dxyT \tag{S20b}$$

$$T_{Sb}(T) \approx y(1 + M)M^{-1} - x(1 + My)^{-1}T \tag{S20c}$$

$$T_{Tb}(T) \approx y(1 + x)(1 + M)M^{-1} + x[1 + y(1 + M)](1 + My)^{-1}T \tag{S20d}$$

Combined with Equations (S9c) and (S9d), these expressions can be used to obtain approximations for F_{ATw} and F_{ATb} as functions of T when dT is small and d is large. After some algebra, the following expressions are obtained:

$$F_{ATw}(T) \approx \frac{x[y+d(2-x)T]}{y[(1+x)+dxT]} \tag{S20e}$$

$$F_{ATb}(T) \approx \frac{x}{(1+x)}\{1 + \frac{M(1-2x+My)}{y(1+x)(1+M)(1+My)}T\} \tag{S20f}$$

These equations imply that F_{ATw} always increases initially when there is no recombination, for the case of an instantaneous sweep; F_{ATb} also increases initially, provided that $y > (2x–1)/M$, a fairly light condition

It is also of interest to ask how the between-population measure of differentiation for the *In* subpopulation behaves in the absence of recombination. This can be measured by considering the within- and between-population mean coalescence times for pairs of randomly sampled haploid genomes that both carry the inversion, giving $F_{ST,In} = 1 - T_{11w}/T_{11b}$, given that *d* is assumed here to be very large. The general equations for coalescence times then imply that:

$$F_{ST,In}(T) \approx \frac{1}{(1+Mx)[1+g(T)]} \qquad \text{(S21a)}$$

where:

$$g(T) = [\frac{M}{(1+Mx)}T_{11b}(0) - 1]\exp[-\frac{MT}{(1+Mx)}]$$

Similarly,

$$1 - F_{ST,In}(T) \approx \frac{Mx+(1+Mx)g(T)}{(1+Mx)[1+g(T)]} \qquad \text{(S21b)}$$

and

$$h(T) = \frac{1-F_{ST,In}(T)}{F_{ST,In}(T)} - Mx \approx -(1+Mx)g(T) \qquad \text{(S22c)}$$

Equation (S22c) implies that:

$$\frac{d\,h(T)}{dT} \approx -\frac{M\,h(T)}{(1+Mx)} \qquad \text{(S22d)}$$

i.e. the asymptotic proportional rate of change of the natural logarithm of the deviation of the ratio of $1 - F_{ST,In}$ to $F_{ST,In}$ from its equilibrium value of $1/(1 + Mx)$ is equal to $-M/(1 + Mx)$, which is the same as the asymptotic proportional rates of change of $\delta T_{11\mathrm{w}}(T)$ and $\delta T_{11\mathrm{b}}(T)$.

As in the case of F_{AT}, the relevance of these asymptotic rates of change is unclear. For small *T*, following the same approach as used above for F_{AT}, we have:

$$F_{ST\ In}(T) \approx \frac{1}{(1+Mx)} \qquad \text{(S23)}$$

with the somewhat surprising result that $F_{ST,In}$ is constant and equal to its equilibrium value, to the order of the approximations used here, despite the fact that it is undefined when $T_{11b} = 0$. This arises from the fact that Equation (S19a) shows that T_{11w} for small $T \approx MxT/(1+Mx)$ whereas Equation (S19b) shows that T_{12b} is proportional to *T*, so that $1 - T_{11w} / T_{11b} \approx 1/(1 + Mx)$.

Another way of obtaining this result is to note that, if α in Equation (S17a) is neglected (as is reasonable for small t), the rate of change per generation in T_{11w}, given by $\boldsymbol{B}$, is as follows:

$$\Delta T_{11w}(t) \approx -\frac{(1+Mx)}{2Nx} T_{11w}(t) + \frac{Mx}{(1+Mx)} T_{11b}(t) \qquad \text{(S24a)}$$

This expression is equal to zero when:

$$\frac{T_{11w}(t)}{T_{11b}(t)} = \frac{Mx}{(1+Mx)} \qquad \text{(S24b)}$$

which is equivalent to Equation (S23). The rate of change per generation in T_{11w} is initially very small, so that T_{11w} reaches a quasi-equilibrium such that its ratio with respect to T_{11b}, and hence $F_{ST,In}$, is approximately constant over time.

A similar treatment of F_{ST} can be applied to the *St* subpopulation by replacing $T_{11w}(t)$ and $T_{11b}(T)$ with $T_{22w}(T)$ and $T_{22b}(T)$, and x with y.

S8 Recursion equations for the second moments of the T_{ij} in a single population

The method for obtaining recursion relations for the expected coalescent times (Equations A1) can be extended to their higher moments in a conceptually straightforward way by replacing the $(1 + t_{ij.})$ in Equations (A1) by $(1 + t_{ij.})^k$, where the . subscript is either w or b., and expanding by the binomial theorem. Given expressions for $k = 1$ and 2, recursions for $k = 3, 4$, etc. can be developed.

For simplicity, only the case of $k = 2$ for a single randomly mating population of size N will be considered in detail here, so that the subscripts w and b can be dropped, allowing determination of the variances of the t_{ij}. This yields the following recursion relations, where t_{ij} and t_{ij}^2 denotes the expectations of the relevant coalescent time and its square, respectively:

$$t_{11}'^2 = (1-2ry)\left[\frac{1}{2Nx} + \left(1-\frac{1}{2Nx}\right)\right](1 + 2t_{11} + t_{11}^2) + 2ry(1 + 2t_{12} + t_{12}^2) \qquad \text{(S25a)}$$

$$t_{22}'^2 = (1-2rx)\left[\frac{1}{2Ny} + \left(1-\frac{1}{2Ny}\right)\right](1 + 2t_{22} + t_{22}^2) + 2rx(1 + 2t_{12} + t_{12}^2) \qquad \text{(S25b)}$$

$$t_{12}'^2 = (1-r)(1 + 2t_{12} + t_{12}^2) + rx(1 + 2t_{11} + t_{11}^2) + ry(1 + 2t_{22} + t_{22}^2) \qquad \text{(S25c)}$$

These equations can be iterated from a specified set of initial conditions in conjunction with the corresponding recursion equations for the expectations of the t_{ij} (see Equations A1 and A2) providing exact expressions for the first and second moments of the t_{ij}. Useful approximations for the equilibrium values of the second moments can be obtained by noting that the expected squares of the equilibrium coalescent times are much greater than than the expected values of the equilibrium coalescent times themselves, so that many of the terms in Equations (S25) can then be neglected. At equilibrium, this procedure yields:

$$t_{11}^2 \frac{1+\rho xy}{2Nx} \approx 2t_{11} + 2ryt_{12}^2 \qquad \text{(S26a)}$$

$$t_{22}^2 \frac{1+\rho xy}{2Ny} \approx 2t_{22} + 2rxt_{12}^2 \qquad \text{(S26b)}$$

$$t_{12}^2 \approx xt_{11}^2 + yt_{22}^2 + 2r^{-1}t_{12} \qquad \text{(S26c)}$$

Transforming to the coalescent time-scale of $2N$ generations, these equations become:

$$T_{11}^2(1+\rho xy) \approx 2xT_{11} + \rho xyT_{12}^2 \qquad \text{(S27a)}$$

$$T_{22}^2(1+\rho xy) \approx 2yT_{22} + \rho xyT_{12}^2 \qquad \text{(S27b)}$$

$$T_{12}^2 \approx xT_{11}^2 + yT_{22}^2 + 4\rho^{-1}T_{12} \qquad \text{(S27c)}$$

where the T_{ij} are given by Equations (5)

After some algebra, we obtain the following expressions:

$$T_{11}^2 \approx \frac{2(1+\rho x^2y)[xT_{11}+2xy(1+2\rho^{-1})]+2\rho xy^3T_{22}+4\rho x^2y^3(1+2\rho^{-1})}{(1+\rho x^2y)(1+\rho xy^2)-\rho^2(xy)^3} \qquad \text{(S28a)}$$

$$T_{22}^2 \approx \frac{2(1+\rho xy^2)[yT_{22}+2xy(1+2\rho^{-1})]+2\rho x^3yT_{11}+4\rho x^3y^2(1+2\rho^{-1})}{(1+\rho x^2y)(1+\rho xy^2)-\rho^2(xy)^3} \qquad \text{(S28b)}$$

T_{12}^2 can be obtained by substituting Equations (S28) into Equation (S27c). The variances and standard deviations of the three coalescent times are then easily found from the corresponding first and second moments.

This method of determining the variances of the pairwise coalescence times can be validated by comparing the standard deviations generated by Equations (5) and (S28) with the results of coalescent simulations when the time of sampling is sufficiently large that the process is close to equilibrium. Results for a wide range of ρ values with $x = 0.1$ and 0.5 are shown in Table S2, and display excellent agreement between the analytical and simulation results.

S9 Statistical error in population genomic estimates of diversity and divergence for polymorphic inversions

Tajima (1983, Equation 30) showed that, for a Wright-Fisher population of size N and a sample size of n, the stochasticity of coalescent times generates a variance in π at a single nucleotide site of:

$$\frac{2(n^2+n+3)}{9n(n-1)}\theta^2$$

$$\sim\frac{2}{9}\theta^2 \qquad \text{for moderately large } n \qquad \text{(S29)}$$

where θ is the scaled mutation rate $4Nu$. In other words, the coefficient of variation (CV) of π due to the coalescent process is only somewhat smaller than 1, as would be expected from the exponential distribution of coalescent times in the Kingman coalescent process, and the strong, but not complete, correlations between the coalescence times of different pairs of alleles in the same sample. There is also a variance of $(n+1)\theta/3(n-1)$ arising from the mutational process itself. For $n = 2$, the variance is simply $\theta + \theta^2$.

In general, the variances of the means of pairwise diversity and divergence statistics over large numbers of nucleotide sites, which are discussed in the main text, depend on the extent to which different sites have correlated genealogies; a mean over m totally independent sites has a variance of $1/m$ times the variance for a single site. Most parts of the genome of eukaryote taxa such as *Drosophila* experience significant rates of recombination, so that different sites do not have totally correlated gene genealogies (McVean 2002). In normally recombining regions of the *Drosophila* genome, linkage disequilibrium (LD), as measured by the squared correlation coefficient between pairs of sites (R^2), approaches the baseline level generated by finite sample size once they are a few hundred basepairs apart (e.g., Charlesworth & Charlesworth 2010, p.384). This suggests that the majority of pairs of sites in a window of 100kb, as in the Kapun et al. (2023) dataset on *In(3R)P* of *D. melanogaster*, can be treated as having independent gene trees, given that R^2 is closely related to the covariances

of the lengths of gene trees (McVean 2002). Thus, the stochastic coalescence variances of mean pairwise diversity and divergence statistics for a sequence of m basepairs over a single window in most regions of the *Drosophila* genome are likely to be multiplied by a factor of the order of $1/m$ compared to the single nucleotide site values.

To apply this reasoning to population genomic data on inversion polymorphisms, it is first necessary to determine the variances of the diversity and divergence statistics for a single site when the division of the population into *In* and *St* haplotypes plus any population structuring is taken into account. Each class of event in a structured coalescent process (e.g., coalescence, migration to a new deme) has an exponentially distributed waiting time. However, the distribution along a chain of such events involves convolutions of the distributions of the individual events, and hence is not itself exponential.

In the case of *In(3R)P*, population subdivision can safely be neglected, so that the results in section S8 can be applied. The results in Table S2 show that T_{11} has the largest coefficient of variation among the T_{ij}, but this is less than 5 for the lowest scaled rate of recombination (ρ = 0.4) between *In* and *St* with $x = 0.1$ and less than 2.5 with $x = 0.5$. For $\rho = 4$ or 40, which the analyses in the main text show are consistent with the *Drosophila* data, the standard deviations of all of the T_{ij} are close to their means.

It is also necessary to determine whether LD decays sufficiently fast within inversion haplotypes for the argument about the variance of mean diversity and divergence statistics to be valid. Kapun et al. (2023, Fig. 3) give detailed information for the *In(3R)P* inversion of *D. melanogaster*. For out-of-Africa populations, bottleneck effects have created substantial LD within both standard and inverted haplotypes, so only their panel A for the Zambian population (where the inversion is present at a frequency of approximately 0.1) provides useful information. This panel shows that *St* haplotypes display the same pattern as for normal genomic regions, as might be expected from its high frequency. For *In* haplotypes, LD starts off at a very high value, but approaches the baseline value for sites about 20kb apart.

There are three reasons for expecting the relatively rare *In* haplotypes to experience more LD than commoner *St* haplotypes. First, an inversion with frequency $x << 0.5$ experiences a higher sampling variance for the drift process than *St*, since its effective population size is Nx. Second, the effective recombination rate between a pair of *In* sites (ignoring the low frequency of exchange between *In* and *St*) is rx, where r is the recombination rate between the sites, so that their net scaled recombination rate is $4Nx^2r$. This low effective

recombination rate may intensify Hill-Robertson interference effects (Charlesworth and Jensen 2021), reducing N_e even further. Third, exchange between *In* and *St* in heterokarotypes is analogous to migration between demes in a subdivided population, inducing some LD (Wakeley & Lessard 2003). However, this effect is small for a pair of populations (Wakeley & Lessard 2003) and can be neglected as a first approximation, relative to the much bigger effect of the low frequency of the inversion.

Remarkably, LD within inversion haplotypes of *In(3R)P* seems to be close to the predictions of equilibrium neutral theory. The expected value of R^2 for the normal autosomal genome is given by the equation of Ohta & Kimura (1971):

$$E\{R^2\} \approx \frac{10+\rho}{(11+\rho)(2+\rho)} \qquad \text{(S30)}$$

For the inversion, $\rho = 4Nr$ is replaced by $\rho_1 = 4Nx^2r$.

In *Drosophila*, gene conversion plays an important role in determining r. If we have two sites that are separated by substantially more than the mean tract length of around 440 basepairs, the chance that they recombine is approximately twice the probability that one of them experiences a conversion event, which is typically 10^{-5} in female meiosis (Korunes and Noor 2019). This is offset by the lack of exchange in male meiosis, so the net contribution to r for autosomal loci is 10^{-5}. For crossovers, the typical rate of recombination in female meiosis for a pair of sites separated by l basepairs is approximately $2l$ x 10^{-8}, so the net contribution to r is l x 10^{-8} (Campos and Charlesworth 2019).

For the normal part of the genome, an N_e of approximately 1.6 x 10^6 is a widely accepted estimate for the Zambian population (Johri et al. 2020), so that $\rho \approx 4$ x 10^6 x $r = 64 + 0.064l$, which can substituted into Equation (S30). For sites 1kb apart, $\rho = 128$, E$\{R^2\} \approx 0.007$; for sites 10kb apart, $\rho = 704$, E$\{R^2\} \approx 0.001$. For an inversion with $x = 0.1$, we have $r_1 = 10^{-6} + l$ x 10^{-9}, and $\rho_1 = 0.64 + 0.00064l$. For sites 1kb apart, $\rho_1 = 1.28$ and E$\{R^2\} \approx 0.28$; for sites 10kb apart, $\rho_1 = 7.04$, E$\{R^2\} \approx 0.10$; for sites 20kb apart, $\rho_1 = 13.44$, E$\{R^2\} \approx 0.06$; for sites 40kb apart, $\rho_1 = 26.24$, E$\{R^2\} \approx 0.03$. These values are very similar to what is seen in panel A of Kapun et al. (2023), suggesting that complications due to Hill-Robertson interference do not play a major role in affecting LD within the *In(3R)P* inversion in Zambia.

It follows that, if the means of diversity and divergence statistics for the inversion over a large number of 100kb windows are used, as was done for the central regions of *In(3R)P* by

Kapun et al. (2023), most pairs of sites will be approximately independent of each other (i.e., there is strong statistical mixing: Billingsley [1995]), and their variances will be close to $1/m$ times the variances for individual sites. To allow for the presence of the high degree of correlation between a substantial fraction of pairs within the same window in the *In* sample, one could (probably quite conservatively) elevate the multiplier to $2/m$ (essentially zero correlation is expected between non-adjacent windows).

While exact calculations of the variances of the mean coalescence time for a sample of n alleles are complicated, even for the case of a single population (Tajima 1983), a very conservative procedure is to assume that, for a sample of size n, all combinations of pairwise coalescence times are completely correlated. The variance of the mean of the pairwise coalescent times over all $n(n - 1)/2$ values is then equal to the variance for a single pair. It follows that upper bounds to the variances of the mean diversity and divergence statistics over m sites from the stochasticity of the coalescent process are given by the squares of their expectations, unless ρ is of order 1 or less, giving an upper bound to their coefficients of variation of approximately $1/\sqrt{0.5m}$.

In addition, we need to consider the variance associated with the mutational process itself; for a pair of alleles of type ij under the infinite sites model, this is equal to $\mathrm{E}\{T_{ij}\}\theta$, where θ is the population scaled mutation rate (Tajima 1983). For the mean of a pairwise diversity statistic for a sample of n alleles taken over m sites, the argument used above shows that the variance $\approx 2\mathrm{E}\{T_{ij}\}\theta\ /m$, corresponding to a coefficient of variation of $1/\sqrt{0.5m\theta}$. For $\theta \approx 0.01$, this dominates over the coalescent process stochasticity term, but is still very small for $m = 10^4$. By the central limit theorem for strongly mixed correlated sites (Billingsley 1995), normal deviate tests on pairwise diversity and divergence statistics should be quite robust for data taken over a large number of nucleotide sites, as would be the case for the central region of a several megabase inversion such as *In(3R)P*, with ample power to detect small differences between theoretical predictions and observations.

Difficulties are, however, likely to arise for comparisons involving the relatively small regions associated with inversion breakpoints, where the arguments based on large numbers of sites are likely to fail; simulation-based methods are probably needed for inference and hypothesis testing with these regions. As noted in the text, however, patterns such as an increase in $\tilde{F}_{AT}$ towards inversion breakpoints appear to be repeatable across several independently derived inversions in *D. melanogaster*, suggesting that conclusions based on such patterns are statistically robust.

Literature cited

Billingsley P. 1995. Probability and Measure. 3rd edition. Chichester, UK: John Wiley.

Campos JL, Charlesworth B. 2019. The effects on neutral variability of recurrent selective sweeps and background selection. Genetics 212:287-303.

Charlesworth B, Charlesworth D. 2010. Elements of Evolutionary Genetics. Greenwood Village, CO: Roberts and Company.

Charlesworth B, Jensen JD. 2021. The effects of selection at linked sites on patterns of genetic variability. Ann. Rev. Ecol. Evol. Syst. 52:177-197.

Johri P, Charlesworth B, Jensen, JD. 2020. Toward an evolutionarily appropriate null model: Jointly inferrring demography and purifying selection. Genetics 215:173-192.

Kapun M, Durmaz Mitchell E, Kawecki T, Schmidt P, Flatt T. 2023. An ancestral balanced inversion polymorphism confers global adaptation. Mol. Biol. Evol. In press.

Korunes K, Noor MAF. 2019. Pervasive gene conversion in chromosomal inversion heterozygotes. Mol. Ecol. 28:1302-1315.

McVean GAT. 2002. A genealogical interpretation of linkage disequilibrium. Genetics 62:987-991.

Ohta T, Kimura M. 1971. Linkage disequilibrium between two segregating nucleotide sites under steady flux of mutations in a finite population. Genetics 68:571-580.

Tajima F. 1983. Evolutionary relationship of DNA sequences in a finite population. Genetics 105:437-460.

Wakeley J, Lessard S. 2003. Theory of the effects of population structure and sampling on patterns of linkage disequilibrium applied to genomic data from humans. Genetics 164:1043-1053.
