# Supplementary material for: The effects of inversion polymorphisms on patterns of neutral genetic diversity
Source: Genetics. 2023 Jun 22;224(4):iyad116. doi: 10.1093/genetics/iyad116 (PMC10411593; doi:10.1093/genetics/iyad116)
Supplement: iyad116_Supplementary_Data [file iyad116_supplementary_data.zip › File_S2_GENETICS-2023-306220.docx]

**File S2**

***The effects of inversion polymorphisms on patterns of neutral genetic diversity***

**(B. Charlesworth)**

**These programs are all written in GFortran for the MacIntosh (https://gnuc.org.wiki/GFortranBinariesMacOS)**

**Program Invdiv10**

! PROGRAM FOR DIVERGENCE BETWEEN INVERSION AND STANDARD ARRANGEMENTS (SINGLE POPULATION)

! Coalescent times as functions of gene conversion/recombination rate

! Standard deviations of mean coalescence times are computed

CHARACTER*20 FOUT

WRITE (*,*) 'Output file?'

READ (*,*) FOUT

OPEN (1,FILE=FOUT)

write (1,*) 'Neutral divergence between inversion and standard arrangements in a single population'

write (1,*) ''

WRITE (*,*) 'Inversion frequency?'

READ (*,*) x

write (1,*) 'Inversion frequency= ',x

write (1,*) ''

write(1,*) 'Results for single population'

write(1,*) 'Times are scaled by 2N'

write (1,*) ''

y=1-x

!!!!!!!!!!!!!!!!!!!!!!!!!!!!!!!!!!!!!!!!!!!!!!!!!!!!!!!!!!!!!!!!!!!!!

20 write(*,*) 'Scaled gene conversion rate?'

READ (*,*) rho

if (rho.le.0.01) go to 100

write(1,*) ''

write(1,*) 'Scaled gene conversion rate= ',rho

write(1,*) ''

rho1=rho*x*y

y=1-x

T11=(1+y*(x-y)+rho1)/(1+rho1)

T22=(1-x*(x-y)+rho1)/(1+rho1)

T12=1+(2.0/rho)

RT=T11/T22

! equilibrium mean coalescent times

A1=rho1*x

A2=rho1*y

A3=2*rho1*y**2

A4=(rho1**2)*x*y

A5=1+2.0/rho

A6=4*rho1*x*(y**2)*A5

A7=2*rho1*(x**2)

A8=4*rho1*(x**2)*y*A5

X1=2*(1+A1)*(x*T11+2*x*y*A5)+A3*T22+A6

! write(*,*) 'A1=',A1,' A5=',A5,' A3=',A3,' A6=',A6

X2=(1+A1)*(1+A2)-A4

V11=(X1/X2)-T11**2

s11=sqrt(V11)

X3=2*(1+A2)*(y*T22+2*x*y*A5)+A7*T11+A8

V22=(X3/X2)-T22**2

s22=sqrt(V22)

X4=x*(X1/X2)+y*(X3/X2)+(4.0/rho)*T12

V12=X4-T12**2

s12=sqrt(V12)

TT=2*x*y*T12+(x**2)*T11+(y**2)*T22

TS=x*T11+y*T22

FAT=1-(TS/TT)

write(1,*) 'T11= ',T11,' T12= ',T12,' T22= ',T22

write(1,*) 's11= ',s11,' s12= ',s12,' s22= ',s22

! s means standard deviation

write(1,*) 'TT= ',TT,' TS= ',TS, 'FAT= ',FAT

write(1,*) 'T11/T22= ',RT

write(1,*) ''

go to 20

100 end program Invdiv10

**program Invdiv2**

! PROGRAM FOR DIVERGENCE BETWEEN INVERSION AND STANDARD ARRANGEMENTS

! Coalescent times as functions of gene conversion/recombination rate and Fst in subdivided population

double precision :: AM,d,m,m1,ANE,ANET,x,y,Fst,Fst1,delf,g,g1,g2,delg,a11,a12,a21,a22,b1,b2

double precision :: det,T11b,T22b,T12b,TTb,TSb,FATb,T11w,T22w,T12w,TTw,TSw,FATw,lambda,AM1,AM2,A1,A2

CHARACTER*20 FINP

CHARACTER*20 FOUT

WRITE (*,*) 'Input file?'

READ (*,*) FINP

OPEN (2,FILE=FINP)

WRITE (*,*) 'Output file?'

READ (*,*) FOUT

OPEN (1,FILE=FOUT)

write (1,*) 'Neutral divergence between inversion and standard arrangements in a subdivided population'

write (1,*) ''

READ (2,*) ANE

READ (2,*) d

READ (2,*) x

READ (2,*) Fst

READ (2,*) delf

READ (2,*) ninc1

READ (2,*) g

READ (2,*) delg

READ (2,*) ninc2

write (1,*) 'Local Ne= ',ANE

ANET=d*ANE

write (1,*) 'Total Ne= ',ANET

write (1,*) 'Number of populations = ',d

write (1,*) 'Initial neutral Fst= ',Fst

write (1,*) 'Steps of FST= ',delf

write (1,*) 'Inversion frequency= ',x

write (1,*) 'Initial gene conversion rate= ',g

write (1,*) 'Steps of gene conversion rates (ratios)= ',delg

write (1,*) 'Number of increments= ',ninc2

write (1,*) ''

y=1-x

x1=1+2*y

x2=1+2*x

!!!!!!!!!!!!!!!!!!!!!!!!!!!!!!!!!!!!!!!!!!!!!!!!!!!!!!!!!!!!!!!!!!!!!

do 20 j=1,ninc2

if(j.eq.1) then

g1=g

else

g1=g1*delg

end if

write(1,*) ''

write(1,*) 'Gene conversion rate= ',g1

rho=4*ANET*g1

write(1,*) 'Scaled value= ',rho

T12=1+(2.0/rho)

write(1,*) ''

write(1,*) 'Results for equivalent panmictic population'

write(1,*) 'Times are scaled by 2Nd'

rho1=rho*x*y

y=1-x

T11=(1+y*(x-y)+rho1)/(1+rho1)

T22=(1-x*(x-y)+rho1)/(1+rho1)

TT=2*x*y*T12+(x**2)*T11+(y**2)*T22

TS=x*T11+y*T22

FAT=1-(TS/TT)

RT=T11/T22

write(1,*) 'T11= ',T11,' T12= ',T12,' T22= ',T22

write(1,*) 'TT= ',TT,' TS= ',TS

write(1,*) 'FAT= ',FAT,' T11/T22= ',RT

write(1,*) ''

write(*,*) 'Gene conversion rate= ',g1

write(*,*) 'Scaled value= ',rho

write(*,*) 'Results for equivalent panmictic population'

write(*,*) 'Times are scaled by 2Nd'

write(*,*) 'T11= ',T11,' T12= ',T12,' T22= ',T22

write(*,*) 'TT= ',TT,' TS= ',TS

write(*,*) 'FAT= ',FAT,' T11/T22= ',RT

write(*,*) ''

write (*,*) 'CONT?'

read (*,*) CONT

! allows program to be cancelled if desired

if(CONT.eq.1) go to 100

!!!!!!!!!!!!!!!!!!!!!!!!!!!!!!!!!!!!!!!!!!!!!!!!!!!!!!!!!!!!!!!!!!!!!!

write(1,*) 'Results for subdivided population'

do 10 i=1,ninc1

write(1,*) ''

Fst1=Fst+delf*(i-1)

write(1,*) 'Fst= ',Fst1

AM=(1-Fst1)/Fst1

m=AM/(4*ANE)

write(1,*) 'Scaled migration rate= ',AM,' Migration rate= ',m

write (1,*) ''

g2=d*g1

AM1=AM*x+1

AM2=AM*y+1

a11=(AM/AM1)+rho*(y**2)

a22=(AM/AM2)+rho*(x**2)

a12=0-rho*(y**2)

a21=0-rho*(x**2)

det=(a11*a22-a12*a21)

b1=x1

b2=x2

if(abs(det).le.0.001) then

lambda=(a21*b1-a11*b2)/(a12*b2-a22*b1)

A1=AM2*(x**2)*x1+AM1*(y**2)*y1

A1=A1/d

A2=AM2*(x**2)+AM1*(y**2)*lambda

T11b=(AM1*AM2-A1)/(AM*A2)

T22b=lambda*T11b

go to 25

end if

! lambda is T22b/T11b

T11b=(a22*b1-a12*b2)/det

T22b=(a11*b2-a21*b1)/det

TSb=x*T11b+y*T22b

25 T12b=TSb+(2.0/rho)

TTb=2*x*y*T12b+(x**2)*T11b+(y**2)*T22b

FATb=1-(TSb/TTb)

RT=T11b/T22b

write(1,*) 'Alleles sampled from different populations'

write(1,*) 'T11b= ',T11b,' T12b= ',T12b,' T22b= ',T22b

write(1,*) 'TTb= ',TTb,' TSb= ',TSb

write(1,*) 'FATb= ',FATb,' T11/T22= ',RT

write(1,*) ''

T11w=((x*x1/d)+AM*x*T11b)/AM1

T22w=((y*x2/d)+AM*y*T22b)/AM2

T12w=(1/(d*AM))+T12b

TSw=x*T11w+y*T22w

TTw=2*x*y*T12w+(x**2)*T11w+(y**2)*T22w

FATw=1-(TSw/TTw)

RT=T11w/T22w

write(1,*) 'Alleles sampled from the same population'

write(1,*) 'T11w= ',T11w,' T12w= ',T12w,' T22w= ',T22w

write(1,*) 'TTw= ',TTw,' TSw= ',TSw

write(1,*) 'FATw= ',FATw,' T11/T22= ',RT

write(1,*) ''

10 continue

20 continue

100 end program Invdiv2

**program Invcoal4**

! PROGRAM FOR DIVERGENCE BETWEEN INVERSION AND STANDARD ARRANGEMENTS

! Coalescent times in panmictic population as functions of gene conversion/recombination rate

! Coalescent simulation of approach to equilibrium

real :: x,y,T11,T22,T12,FAT,T0,x0,x1,T1S,T1SS,T2S,T2SS,T3S,T3SS

real :: rho,lam1r,lam1c,lam2r,lam2c,lam1,lam2,lam3,lamt

real :: expdev

integer :: i,j1,j2,j3,nrep

CHARACTER*20 FINP

CHARACTER*20 FOUT

WRITE (*,*) 'Input file?'

READ (*,*) FINP

OPEN (2,FILE=FINP)

WRITE (*,*) 'Output file?'

READ (*,*) FOUT

OPEN (1,FILE=FOUT)

write (1,*) 'Neutral divergence between inversion and standard arrangements in a panmictic population'

write (1,*) 'Coalescent simulation of approach to equilibrium'

write (1,*) 'Times scaled by 2x population size'

write (1,*) ''

READ (2,*) x

READ (2,*) rho

READ (2,*) nrep

write (1,*) 'Inversion frequency= ',x

write (1,*) 'Scaled recombination rate= ',rho

write (1,*) 'Number of replicates= ',nrep

write (1,*) ''

write (*,*) 'Inversion frequency= ',x

write (*,*) 'Scaled recombination rate= ',rho

write (*,*) 'Number of replicates= ',nrep

write (*,*) ''

y=1-x

lam1r=rho*y

lam2r=rho*x

lam1c=1.0/x

lam2c=1.0/y

lam1=lam1r+lam1c

lam2=0.5*rho

lam3=lam2r+lam2c

anrep=nrep

!!!!!!!!!!!!!!!!!!!!!!!!!!!!!!!!!!!!!!!!!!!!!!!!!!!!!!!!!!!!!!!!!!!!!

T11=0

T22=1

T12=1

TT=2*x*y*T12+(x**2)*T11+(y**2)*T22

TS=x*T11+y*T22

FAT=1-y/(1-x**2)

write(1,*) 'Initial state of population'

write(1,*) 'T11= ',T11,' T12= ',T12,' T22b= ',T22

write(1,*) 'TT= ',TT,' TS= ',TS

write(1,*) 'FAT= ',FAT,' T11/T22= ',RT

write(1,*) ''

T12=1+(2.0/rho)

write(1,*) ''

write(1,*) 'Equilibrium results for panmictic population'

write(1,*) 'Times are scaled by 2Ne'

rho1=rho*x*y

xx1=1-2*y

T11=(1+rho1+y*xx1)/(1+rho1)

T22=(1+rho1-x*xx1)/(1+rho1)

TT=2*x*y*T12+(x**2)*T11+(y**2)*T22

TS=x*T11+y*T22

FAT=1-(TS/TT)

RT=T11/T22

write(1,*) 'T11= ',T11,' T12= ',T12,' T22= ',T22

write(1,*) 'TT= ',TT,' TS= ',TS

write(1,*) 'FAT= ',FAT,' T11/T22= ',RT

write(1,*) ''

write(*,*) 'T11= ',T11,' T12= ',T12,' T22= ',T22

write(*,*) 'TT= ',TT,' TS= ',TS

write(*,*) 'FAT= ',FAT,' T11/T22= ',RT

write(*,*) ''

write (*,*) 'CONT?'

read (*,*) CONT

! allows program to be cancelled if desired

if(CONT.eq.1) go to 100

!!!!!!!!!!!!!!!!!!!!!!!!!!!!!!!!!!!!!!!!!!!!!!!!!!!!!!!!!!!!!!!!!!!!!!

call init_random_seed()

anrep=nrep

write(*,*) 'anrep=',anrep

sqnrep=sqrt(anrep)

10 write (*,*) 'Time to origin of inversion?'

read (*,*) T0

write (*,*) ''

if(T0.le.0.0001) go to 100

write (1,*) ''

write (1,*) 'Time to origin of inversion= ',T0

write (1,*) ''

call coal11(lam1r,lam1c,lam1,lam2r,lam2c,lam2,lam3,T0,nrep,T1S,T1SS)

T11m=T1S/anrep

T11v=T1SS-(T1S**2)/anrep

T11v=T11v/(anrep-1.0)

sd11=sqrt(T11v)

se11=sd11/sqnrep

call coal12(lam1r,lam1c,lam1,lam2r,lam2c,lam2,lam3,T0,nrep,T2S,T2SS)

T12m=T2S/anrep

T12v=T2SS-(T2S**2)/anrep

T12v=T12v/(anrep-1.0)

sd12=sqrt(T12v)

se12=sd12/sqnrep

call coal22(lam1r,lam1c,lam1,lam2r,lam2c,lam2,lam3,T0,nrep,T3S,T3SS)

T22m=T3S/anrep

T22v=T3SS-(T3S**2)/anrep

T22v=T22v/(anrep-1.0)

sd22=sqrt(T22v)

se22=sd22/sqnrep

write(1,*) 'Mean of T11= ',T11m, ' s.d.= ',sd11

write(1,*) 's.e.= ',se11

write(1,*) ''

write(1,*) 'Mean of T12= ',T12m, ' s.d.= ',sd12

write(1,*) 's.e.= ',se12

write(1,*) ''

write(1,*) 'Mean of T22= ',T22m, ' s.d.= ',sd22

write(1,*) 's.e.= ',se22

write(1,*) ''

a=2*x*y

b=x**2

c=y**2

TT=a*T12m+b*T11m+c*T22m

TS=x*T11m+y*T22m

FAT=1-TS/TT

VTT=(a**2)*T12v+(b**2)*T11v+(c**2)*T22v

VTS=(x**2)*T11v+(y**2)*T22v

CTS=(b*x)*T11v+(c*y)*T22v

! covariance of TS and TT

sdTT=sqrt(VTT)

seTT=sdTT/sqnrep

sdTS=sqrt(VTS)

seTS=sdTS/sqnrep

VFAT=(VTS/TT**2)+(VTT*(TS**2/TT**4))

VFAT=VFAT-2*CTS*(TS/TT**3)

sdFAT=sqrt(VFAT)

seFAT=sdFAT/sqnrep

write(1,*) 'Mean of TS= ',TS,' s.d.= ',sdTS

write(1,*) 's.e.= ',seTS

write(1,*) ''

write(1,*) 'Mean of TT= ',TT,' s.d.= ',sdTT

write(1,*) 's.e.= ',seTT

write(1,*) ''

write(1,*) 'Mean of FAT= ',FAT,' s.d.= ',sdFAT

write(1,*) 's.e.= ',seFAT

write(1,*) ''

go to 10

100 end program Invcoal4

subroutine coal11(lam1r,lam1c,lam1,lam2r,lam2c,lam2,lam3,T0,nrep,T1S,T1SS)

real:: x0,x1,x2,x3,x4,lam1r,lam1c,lam1,lam2r,lam2c,lam2,lam3

real:: T1,T2,T3,T1S,T1SS,pc1,pc2,pr1

integer :: j1,j2,j3,nrep

! simulates process for determining distribution of T11

pc1=lam1c/lam1

pc2=lam2c/lam3

pr1=0.5*lam2r/lam2

! write(*,*) 'pc1=',pc1,'pc2=',pc2,'pr1= ',pr1

! write(*,*) ''

T1S=0

T1SS=0

do 10 k=1,nrep

x0=0.0

! write(*,*) 'rep=',k

! process is initiated as 1/1

15 call coal1(x0,x1,lam1r,lam1c,lam1,T0,T1,j1,pc1)

! write(*,*) 'Process is 11'

! write(*,*) 'x0= ',x0

if(j1.eq.1) then

! write(*,*) 'T1 ',T1

! write(*,*) 'Termination'

! write(*,*) ''

T1S=T1S+T1

T1SS=T1SS+T1**2

go to 10

! process is terminated for this replicate

end if

20 call coal2(x1,x2,lam1r,lam2r,lam2,T0,T2,j2,pr1)

! process is now 1/2

! write(*,*) 'Process is 12'

! write(*,*) 'x1= ',x1

x0=x2

if(j2.eq.1) then

call coal0(x0,x3)

T1S=T1S+x3

T1SS=T1SS+x3**2

! process is now pre-inversion standard coalescent and terminates

go to 10

end if

if(j2.eq.2) go to 15

! process is now 1/1

call coal3(x0,x3,lam2r,lam2c,lam3,T0,T3,j3,pc2)

! process is now 2/2

! write(*,*) 'Process is 22'

! write(*,*) 'x0= ',x0

x0=x3

if(j3.eq.1) then

call coal0(x0,x4)

T1S=T1S+x4

T1SS=T1SS+x4**2

! process is now pre-inversion standard coalescent and terminates

go to 10

end if

if(j3.eq.2) then

T1S=T1S+T3

T1SS=T1SS+T3**2

! write(*,*) 'T3 ',T3

! write(*,*) 'Termination'

! write(*,*) ''

go to 10

! process is terminated for this replicate

else

x1=x3

go to 20

! process is now 1/2

end if

10 continue

end subroutine coal11

subroutine coal12(lam1r,lam1c,lam1,lam2r,lam2c,lam2,lam3,T0,nrep,T2S,T2SS)

real:: x0,x1,x2,x3,x4,x5,lam1r,lam1c,lam1,lam2r,lam2c,lam2,lam3,T2S,T2SS,pc1,pc2,pr1

real:: T1,T2,T3,T0

integer :: j1,j2,j3,nrep

! simulates process for determining distribution of T12

pc1=lam1c/lam1

pc2=lam2c/lam3

pr1=0.5*lam2r/lam2

! write(*,*) 'pc1=',pc1,'pc2=',pc2,'pr1= ',pr1

! write(*,*) ''

T2S=0

T2SS=0

do 10 k=1,nrep

x0=0.0

! write(*,*) 'rep=',k

15 call coal2(x0,x1,lam1r,lam2r,lam2,T0,T2,j2,pr1)

! process is initiated as 1/2

! write(*,*) 'Process is 12'

! write(*,*) 'x1= ',x1

x0=x1

if(j2.eq.1) then

call coal0(x0,x3)

T2S=T2S+x3

T2SS=T2SS+x3**2

! process is now pre-inversion standard coalescent and terminates

go to 10

end if

if(j2.eq.2) go to 30

! process is now 1/1

call coal3(x0,x2,lam2r,lam2c,lam3,T0,T3,j3,pc2)

! process is now 2/2

! write(*,*) 'Process is 22'

! write(*,*) 'x0= ',x0

x0=x2

if(j3.eq.1) then

call coal0(x0,x4)

T2S=T2S+x4

T2SS=T2SS+x4**2

! process is now pre-inversion standard coalescent and terminates

go to 10

end if

if(j3.eq.2) then

T2S=T2S+T3

T2SS=T2SS+T3**2

! write(*,*) 'T3 ',T3

! write(*,*) 'Termination'

! write(*,*) ''

go to 10

! process is terminated for this replicate

else

go to 15

! process is now 1/2

end if

30 call coal1(x0,x5,lam1r,lam1c,lam1,T0,T1,j1,pc1)

! process is now 1/1

! write(*,*) 'Process is 11'

! write(*,*) 'x0= ',x0

if(j1.eq.1) then

T2S=T2S+T1

T2SS=T2SS+T1**2

! write(*,*) 'T1 ',T1

! write(*,*) 'Termination'

! write(*,*) ''

go to 10

! process is terminated for this replicate

end if

x0=x5

! process is now 1/2

go to 15

10 continue

end subroutine coal12

subroutine coal22(lam1r,lam1c,lam1,lam2r,lam2c,lam2,lam3,T0,nrep,T3S,T3SS)

real:: x0,x1,x2,x3,x4,x5,lam1r,lam1c,lam1,lam2r,lam2c,lam2,lam3

real:: T1,T2,T3,T3S,T3SS,pc1,pc2,pr1

integer :: j1,j2,j3,nrep

! simulates process for determining distribution of T22

pc1=lam1c/lam1

pc2=lam2c/lam3

pr1=0.5*lam2r/lam2

! write(*,*) 'pc1=',pc1,'pc2=',pc2,'pr1= ',pr1

! write(*,*) ''

T3S=0

T3SS=0

do 10 k=1,nrep

x0=0.0

! write(*,*) 'rep=',k

! process is initiated as 2/2

15 call coal3(x0,x3,lam2r,lam2c,lam3,T0,T3,j3,pc2)

! write(*,*) 'Process is 22'

! write(*,*) 'x0= ',x0

x0=x3

if(j3.eq.1) then

call coal0(x0,x4)

T3S=T3S+x4

T3SS=T3SS+x4**2

! process is now pre-inversion standard coalescent and terminates

go to 10

end if

if(j3.eq.2) then

T3S=T3S+T3

T3SS=T3SS+T3**2

! write(*,*) 'T3 ',T3

! write(*,*) 'Termination'

! write(*,*) ''

go to 10

! process is terminated for this replicate

else

x1=x3

! process is now 1/2

end if

20 call coal2(x1,x2,lam1r,lam2r,lam2,T0,T2,j2,pr1)

! process is now 1/2

! write(*,*) 'Process is 12'

! write(*,*) 'x1= ',x1

x0=x2

if(j2.eq.1) then

call coal0(x0,x5)

T3S=T3S+x5

T3SS=T3SS+x5**2

! process is now pre-inversion standard coalescent and terminates

go to 10

end if

if(j2.eq.3) go to 15

! process is now 2/2

call coal1(x0,x1,lam1r,lam1c,lam1,T0,T1,j1,pc1)

! write(*,*) 'Process is 11'

! write(*,*) 'x0= ',x0

if(j1.eq.1) then

! write(*,*) 'T1 ',T1

! write(*,*) 'Termination'

! write(*,*) ''

T3S=T3S+T1

T3SS=T3SS+T1**2

go to 10

! process is terminated for this replicate

end if

! process is now 1/2

go to 20

10 continue

end subroutine coal22

subroutine coal0(x0,x1)

real:: x0,x1,lam0,expdev,z

! simulates coalescent events within St before inversion occurred

lam0=1.0

z=expdev(lam0)

! returns exponential variate with parameter lam0

x1=x0+z

! process terminates with coalescent time x1

10 end subroutine coal0

subroutine coal1(x0,x1,lam1r,lam1c,lam1,T0,T1,j,pc1)

real:: x0,x1,lam1r,lam1c,lam1,T0,T1,r,expdev,z,pc1

integer :: j

! simulates recombination and coalescent events within 1/1 class

z=expdev(lam1)

! returns exponential variate with parameter lam1

x1=x0+z

! write(*,*) 'Inside coal1'

! write(*,*) 'z= ',z,'x1= ',x1

if(x1.ge.T0) then

T1=T0

j=1

! forced coalescence at time T0

go to 10

end if

call random_number(r)

! write(*,*) 'pc1',pc1,'r=',r

if(r.le.pc1) then

! coalescent event occurs

j=1

T1=x1

! coalescence at time x1

else

j=2

! movement to class 1/2

end if

10 end subroutine coal1

subroutine coal2(x0,x1,lam1r,lam2r,lam2,T0,T2,j,pr1)

real :: x0,x1,lam1r,lam2r,lam2,T0,T2,r,expdev,z,pr1

integer :: j

! simulates recombination events within 1/2 class

z=expdev(lam2)

! returns exponential variate with parameter lam2

x1=x0+z

! write(*,*) 'Inside coal2'

! write(*,*) 'z=',z,'x0=',x0,'x1=',x1

if(x1.ge.T0) then

x1=T0

j=1

! indicates movement to pre-inversion state

go to 10

end if

call random_number(r)

! write(*,*) 'pr1=',pr1,'r=',r

if(r.le.pr1) then

j=2

! this indicates movement to 1/1 class

else

! this indicates movement to 2/2 class

j=3

! times to these events are stored as x1

end if

10 end subroutine coal2

subroutine coal3(x0,x1,lam2r,lam2c,lam3,T0,T3,j,pc2)

real :: x0,x1,lam2r,lam2c,lam3,T0,T3,r,expdev,z,pc2

integer :: j

! simulates recombination and coalescent events within 1/1 class

z=expdev(lam3)

! returns exponential variate with parameter lam3

x1=x0+z

! write(*,*) 'Inside coal3'

! write(*,*) 'z=',z,'x0=',x0,'x1=',x1

if(x1.ge.T0) then

x1=T0

j=1

! indicates movement to pre-inversion state

go to 10

end if

call random_number(r)

! write (*,*) 'pc2',pc2,'r=',r

if(r.le.pc2) then

! coalescent event occurs

j=2

T3=x1

! coalescence at time x1

else

j=3

! movement to class 1/2

end if

10 end subroutine coal3

function expdev(lam)

! exponential variate with parameter lambda

real :: expdev,lam,r,x

call random_number(r)

x=r

expdev=0.0-log(1-x)/lam

end function expdev

subroutine init_random_seed()

use iso_fortran_env, only: int64

implicit none

integer, allocatable :: seed(:)

integer :: i, n, un, istat, dt(8), pid

integer(int64) :: t

call random_seed(size = n)

allocate(seed(n))

! First try if the OS provides a random number generator

open(newunit=un, file="/dev/urandom", access="stream", &

form="unformatted", action="read", status="old", iostat=istat)

if (istat == 0) then

read(un) seed

close(un)

else

! Fallback to XOR:ing the current time and pid. The PID is

! useful in case one launches multiple instances of the same

! program in parallel.

call system_clock(t)

if (t == 0) then

call date_and_time(values=dt)

t = (dt(1) - 1970) * 365_int64 * 24 * 60 * 60 * 1000 &

+ dt(2) * 31_int64 * 24 * 60 * 60 * 1000 &

+ dt(3) * 24_int64 * 60 * 60 * 1000 &

+ dt(5) * 60 * 60 * 1000 &

+ dt(6) * 60 * 1000 + dt(7) * 1000 &

+ dt(8)

end if

pid = getpid()

t = ieor(t, int(pid, kind(t)))

do i = 1, n

seed(i) = lcg(t)

end do

end if

call random_seed(put=seed)

contains

! This simple PRNG might not be good enough for real work, but is

! sufficient for seeding a better PRNG.

function lcg(s)

integer :: lcg

integer(int64) :: s

if (s == 0) then

s = 104729

else

s = mod(s, 4294967296_int64)

end if

s = mod(s * 279470273_int64, 4294967291_int64)

lcg = int(mod(s, int(huge(0), int64)), kind(0))

end function lcg

end subroutine init_random_seed

**program Invdiv6**

! PROGRAM FOR DIVERGENCE BETWEEN INVERSION AND STANDARD ARRANGEMENTS

! Coalescent times in panmictic population as functions of gene conversion/recombination rate

! Time course of approach to equilibrium

double precision :: x,y,g,T11,T22,T12,xx1

double precision :: T11e,T22e,T12e,TT,TS,FAT

double precision :: a(3,3),x1(3),x2(3),del(3),delsum,rho,rho1

CHARACTER*20 FINP

CHARACTER*20 FOUT

WRITE (*,*) 'Input file?'

READ (*,*) FINP

OPEN (2,FILE=FINP)

WRITE (*,*) 'Output file?'

READ (*,*) FOUT

OPEN (1,FILE=FOUT)

write (1,*) 'Neutral divergence between inversion and standard arrangements in a panmictic population'

write (1,*) 'Time course of approach to equilibrium'

write (1,*) 'Times scaled by 2x population size; changes accelerated by a specified factor'

write (1,*) ''

READ (2,*) ANE

READ (2,*) x

READ (2,*) g

READ (2,*) ngen

READ (2,*) nint

READ (2,*) tfac

write (1,*) 'Ne= ',ANE

write (1,*) 'Inversion frequency= ',x

write (1,*) 'Gene conversion rate= ',g

write (1,*) 'Maximum number of times= ',ngen

write (1,*) 'Print interval= ',nint

write (1,*) 'Multiplication factor for changes= ',tfac

write (1,*) ''

write (*,*) 'Ne= ',ANE

write (*,*) 'Inversion frequency= ',x

write (*,*) 'Gene conversion rate= ',g

write (*,*) 'Maximum number of times= ',ngen

write (*,*) 'Print interval= ',nint

write (*,*) 'Multiplication factor for changes= ',tfac

write (*,*) ''

y=1-x

xx1=1-2*y

!!!!!!!!!!!!!!!!!!!!!!!!!!!!!!!!!!!!!!!!!!!!!!!!!!!!!!!!!!!!!!!!!!!!!

rho=4*ANE*g

write (1,*) ' Scaled gene conversion rate= ',rho

T12=1+(2.0/rho)

write(1,*) ''

write(1,*) 'Equilibrium results for panmictic population'

write(1,*) 'Times are scaled by 2Ne'

rho1=rho*x*y

T11=(1+rho1+y*xx1)/(1+rho1)

T22=(1+rho1-x*xx1)/(1+rho1)

TT=2*x*y*T12+(x**2)*T11+(y**2)*T22

TS=x*T11+y*T22

FAT=1-(TS/TT)

RT=T11/T22

write(1,*) 'T11= ',T11,' T12= ',T12,' T22= ',T22

write(1,*) 'TT= ',TT,' TS= ',TS

write(1,*) 'FAT= ',FAT,' T11/T22= ',RT

write(1,*) ''

write(*,*) 'T11= ',T11,' T12= ',T12,' T22= ',T22

write(*,*) 'TT= ',TT,' TS= ',TS

write(*,*) 'FAT= ',FAT,' T11/T22= ',RT

write(*,*) ''

write (*,*) 'CONT?'

read (*,*) CONT

! allows program to be cancelled if desired

if(CONT.eq.1) go to 100

!!!!!!!!!!!!!!!!!!!!!!!!!!!!!!!!!!!!!!!!!!!!!!!!!!!!!!!!!!!!!!!!!!!!!!

T11e=T11

T22e=T22

T12e=T12

AN1=2*ANE*x

AN2=2*ANE*y

! Section on approach to equilibrium

! Matrix components

a(1,1)=1-2*g*y-1.0/AN1

a(1,2)=0

a(1,3)=2*g*y

a(2,1)=0

a(2,2)=1-2*g*x-1.0/AN2

a(2,3)=2*g*x

a(3,1)=g*x

a(3,2)=g*y

a(3,3)=1-g

write(1,*) 'Initial state of population'

T11=0

T22=1

T12=1

TS=x*T11+y*T22

TT=2*x*y*T12+(x**2)*T11+(y**2)*T22

FAT=1-(TS/TT)

RT=T11/T22

write(1,*) ''

write(1,*) 'T11= ',T11,' T12= ',T12,' T22b= ',T22

write(1,*) 'TT= ',TT,' TS= ',TS

write(1,*) 'FAT= ',FAT,' T11/T22= ',RT

write(1,*) ''

! initial deviations from equilibrium

x1(1)=0-T11e

x1(2)=1-T22e

x1(3)=1-T12e

icount=0

do 10 i=1,ngen

icount=icount+1

! counter for determining whether or not to print

if(icount.eq.nint) then

write(1,*) ''

at=i*tfac/(2*ANE)

write(1,*) 'Time (relative to 2Ne) = ',at

T11=x1(1)+T11e

T22=x1(2)+T22e

T12=x1(3)+T12e

TS=x*T11+y*T22

TT=2*x*y*T12+(x**2)*T11+(y**2)*T22

FAT=1-(TS/TT)

RT=T11/T22

write (1,*) ''

write(1,*) 'T11= ',T11,' T12= ',T12,' T22b= ',T22

write(1,*) 'TT= ',TT,' TS= ',TS

write(1,*) 'FAT= ',FAT,' T11/T22= ',RT

write(1,*) ''

icount=0

end if

do 20 j1=1,3

x2(j1)=0

do 25 k1=1,3

x2(j1)=x2(j1)+x1(k1)*a(j1,k1)

! matrix multiplication

25 continue

20 continue

delsum=0.0

do 40 i1=1,3

del(i1)=(x2(i1)-x1(i1))*tfac

delsum=delsum+del(i1)**2

x1(i1)=x1(i1)+del(i1)

! accelerates changes

40 continue

! delsum=sqrt(delsum)

! if(delsum.le.0.0001) go to 50

! terminates run if changes are small

10 continue

write(1,*) 'Final state of population'

write(1,*) 'Time= ',i

write (1,*) ''

write(1,*) 'T11= ',T11,' T12= ',T12,' T22b= ',T22

write(1,*) 'TT= ',TT,' TS= ',TS

write(1,*) 'FAT= ',FAT,' T11/T22= ',RT

write(1,*) ''

write(1,*) ''

100 end program Invdiv6

**program Invdiv4**

! PROGRAM FOR DIVERGENCE BETWEEN INVERSION AND STANDARD ARRANGEMENTS

! Coalescent time in metapopulation as function of gene conversion/recombination rate and Fst

! Time course of approach to equilibrium

double precision :: AM,d,d2,m,ANE,ANET,x,y,Fst,Fst1,g,g2,a11,a12,a21,a22,b1,b2,T11,T22,T12,xx1,xx2,xx3

double precision :: det,T11b,T22b,T12b,TTb,TSb,FATb,T11w,T22w,T12w,TTw,TSw,FATw,lambda,AM1,AM2,A1,A2

double precision :: a(6,6),x1(6),x2(6),del(6),delsum,AN1,AN2,rho,rho1,T11be,T22be,T12be,T11we,T22we,T12we

CHARACTER*20 FINP

CHARACTER*20 FOUT

WRITE (*,*) 'Input file?'

READ (*,*) FINP

OPEN (2,FILE=FINP)

WRITE (*,*) 'Output file?'

READ (*,*) FOUT

OPEN (1,FILE=FOUT)

write (1,*) 'Neutral divergence between inversion and standard arrangements in a subdivided population'

write (1,*) 'Time course of approach to equilibrium'

write (1,*) 'Times scaled by 2x population size; changes accelerated by a specified factor'

write (1,*) ''

READ (2,*) ANE

READ (2,*) d

READ (2,*) x

READ (2,*) Fst

READ (2,*) g

READ (2,*) ngen

READ (2,*) nint

READ (2,*) tfac

write (1,*) 'Local Ne= ',ANE

ANET=d*ANE

write (1,*) 'Total Ne= ',ANET

write (1,*) 'Number of populations = ',d

write (1,*) 'Neutral Fst= ',Fst

write (1,*) 'Inversion frequency= ',x

write (1,*) 'Gene conversion rate= ',g

write (1,*) 'Maximum number of times= ',ngen

write (1,*) 'Print interval= ',nint

write (1,*) 'Multiplication factor for changes= ',tfac

write (1,*) ''

write (*,*) 'Total Ne= ',ANET

write (*,*) 'Number of populations = ',d

write (*,*) 'Neutral Fst= ',Fst

write (*,*) 'Inversion frequency= ',x

write (*,*) 'Gene conversion rate= ',g

write (*,*) 'Maximum number of times= ',ngen

write (*,*) 'Print interval= ',nint

write (*,*) 'Multiplication factor for changes= ',tfac

write (*,*) ''

y=1-x

xx1=1-2*y

xx2=1+2*y

xx3=1+2*x

!!!!!!!!!!!!!!!!!!!!!!!!!!!!!!!!!!!!!!!!!!!!!!!!!!!!!!!!!!!!!!!!!!!!!

rho=4*ANET*g

write (1,*) ' Scaled gene conversion rate= ',rho

T12=1+(2.0/rho)

write(1,*) ''

write(1,*) 'Equilibrium results for equivalent panmictic population'

write(1,*) 'Times are scaled by 2Nd'

rho1=rho*x*y

T11=(1+rho1+y*xx1)/(1+rho1)

T22=(1+rho1-x*xx1)/(1+rho1)

TT=2*x*y*T12+(x**2)*T11+(y**2)*T22

TS=x*T11+y*T22

FAT=1-(TS/TT)

RT=T11/T22

write(1,*) 'T11= ',T11,' T12= ',T12,' T22= ',T22

write(1,*) 'TT= ',TT,' TS= ',TS

write(1,*) 'FAT= ',FAT,' T11/T22= ',RT

write(1,*) ''

write(*,*) 'T11= ',T11,' T12= ',T12,' T22= ',T22

write(*,*) 'TT= ',TT,' TS= ',TS

write(*,*) 'FAT= ',FAT,' T11/T22= ',RT

write(*,*) ''

write (*,*) 'CONT?'

read (*,*) CONT

! allows program to be cancelled if desired

if(CONT.eq.1) go to 100

!!!!!!!!!!!!!!!!!!!!!!!!!!!!!!!!!!!!!!!!!!!!!!!!!!!!!!!!!!!!!!!!!!!!!!

write(1,*) 'Equilibrium results for subdivided population'

AM=(1-Fst)/Fst

m=AM/(4*ANE)

write(1,*) 'Scaled migration rate= ',AM,' Migration rate= ',m

write (1,*) ''

AN1=2*ANE*x

AN2=2*ANE*y

g2=d*g

AM1=AM*x+1

AM2=AM*y+1

a11=2*(m+g2*AM1*(y**2))

a22=2*(m+g2*AM2*(x**2))

a12=0-2*g2*AM1*(y**2)

a21=0-2*g2*AM2*(x**2)

det=(a11*a22-a12*a21)*2*ANET

b1=d*xx2*AM1

b2=d*xx3*AM2

if(abs(det).le.0.001) then

lambda=(a21*b1-a11*b2)/(a12*b2-a22*b1)

A1=AM2*(x**2)*xx2+AM1*(y**2)*xx3

A1=A1/d

A2=AM2*(x**2)+AM1*(y**2)*lambda

T11b=(AM1*AM2-A1)/(AM*A2)

T22b=lambda*T11b

go to 50

end if

! lambda is T22b/T11b

T11b=(a22*b1-a12*b2)/det

T22b=(a11*b2-a21*b1)/det

TSb=x*T11b+y*T22b

50 T12b=TSb+(2.0/rho)

TTb=2*x*y*T12b+(x**2)*T11b+(y**2)*T22b

FATb=1-(TSb/TTb)

RTb=T11b/T22b

write(1,*) 'Alleles sampled from different populations'

write(1,*) 'T11b= ',T11b,' T12b= ',T12b,' T22b= ',T22b

write(1,*) 'TTb= ',TTb,' TSb= ',TSb

write(1,*) 'FATb= ',FATb,' T11/T22= ',RTb

write(1,*) ''

T11w=((x*xx2/d)+AM*x*T11b)/AM1

T22w=((y*xx3/d)+AM*y*T22b)/AM2

T12w=(1/(d*AM))+T12b

TSw=x*T11w+y*T22w

TTw=2*x*y*T12w+(x**2)*T11w+(y**2)*T22w

FATw=1-(TSw/TTw)

RTw=T11w/T22w

write(1,*) 'Alleles sampled from the same population'

write(1,*) 'T11w= ',T11w,' T12w= ',T12w,' T22w= ',T22w

write(1,*) 'TTw= ',TTw,' TSw= ',TSw

write(1,*) 'FATw= ',FATw,' T11/T22= ',RTw

write(1,*) ''

write(*,*) 'Equilibrium results for subdivided population'

write(*,*) ''

write(*,*) 'Alleles sampled from different populations'

write(*,*) 'T11b= ',T11b,' T12b= ',T12b,' T22b= ',T22b

write(*,*) 'TTb= ',TTb,' TSb= ',TSb

write(*,*) 'FATb= ',FATb,' T11/T22= ',RTb

write(*,*) ''

write(*,*) 'Alleles sampled from the same population'

write(*,*) 'T11w= ',T11w,' T12w= ',T12w,' T22w= ',T22w

write(*,*) 'TTw= ',TTw,' TSw= ',TSw

write(*,*) 'FATw= ',FATw,' T11/T22= ',RTw

write(*,*) ''

T11we=T11w

T22we=T22w

T11be=T11b

T22be=T22b

T12we=T12w

T12be=T12b

write (*,*) 'CONT?'

read (*,*) CONT

! allows program to be cancelled if desired

if(CONT.eq.1) go to 100

!!!!!!!!!!!!!!!!!!!!!!!!!!!!!!!!!!!!!!!!!!!!!!!!!!!!!!!!!!!!!!!!!!!!!!

! Section on approach to equilibrium

! Matrix components

d1=d-1

a(1,1)=1-2*m-2*g*y-1.0/AN1

a(1,2)=2*m

a(1,3)=0

a(1,4)=0

a(1,5)=2*g*y

a(1,6)=0

a(2,1)=2*m/d1

a(2,2)=1.0-a(2,1)-2*g*y

a(2,3)=0

a(2,4)=0

a(2,5)=0

a(2,6)=a(1,5)

a(3,1)=0

a(3,2)=0

a(3,3)=1-a(1,2)-2*g*x-1.0/AN2

a(3,4)=a(1,2)

a(3,5)=2*g*x

a(3,6)=0

a(4,1)=0

a(4,2)=0

a(4,3)=a(2,1)

a(4,4)=1-a(2,1)-2*g*x

a(4,5)=0

a(4,6)=2*g*x

a(5,1)=g*x

a(5,2)=0

a(5,3)=g*y

a(5,4)=0

a(5,5)=1-a(1,2)-g

a(5,6)=a(1,2)

a(6,1)=0

a(6,2)=a(5,1)

a(6,3)=0

a(6,4)=a(5,3)

a(6,5)=a(2,1)

a(6,6)=1-a(2,1)-g

write(1,*) 'Initial state of subdivided population'

T11w=0

T22w=1

T12w=1

T11b=0

T22b=1+((d-1)/(AM*d))

T12b=T22b

TSb=x*T11b+y*T22b

TTb=2*x*y*T12b+(x**2)*T11b+(y**2)*T22b

FATb=1-(TSb/TTb)

RTb=T11b/T22b

write(1,*) ''

write(1,*) 'Alleles sampled from different populations'

write(1,*) 'T11b= ',T11b,' T12b= ',T12b,' T22b= ',T22b

write(1,*) 'TTb= ',TTb,' TSb= ',TSb

write(1,*) 'FATb= ',FATb,' T11/T22= ',RTb

write(1,*) ''

TSw=x*T11w+y*T22w

TTw=2*x*y*T12w+(x**2)*T11w+(y**2)*T22w

FATw=1-(TSw/TTw)

RTw=T11w/T22w

write(1,*) 'Alleles sampled from the same population'

write(1,*) 'T11w= ',T11w,' T12w= ',T12w,' T22w= ',T22w

write(1,*) 'TTw= ',TTw,' TSw= ',TSw

write(1,*) 'FATw= ',FATw,' T11/T22= ',RTw

write(1,*) ''

! initial deviations from equilibrium

x1(1)=0-T11we

x1(2)=0-T11be

x1(3)=1-T22we

x1(4)=T22b-T22be

x1(5)=1-T12we

x1(6)=T12b-T12be

icount=0

do 10 i=1,ngen

icount=icount+1

! counter for determining whether or not to print

if(icount.eq.nint) then

write(1,*) ''

at=i*tfac/(2*ANET)

write(1,*) 'Time (units of 2NT generations)= ',at

T11w=x1(1)+T11we

T11b=x1(2)+T11be

T22w=x1(3)+T22we

T22b=x1(4)+T22be

T12w=x1(5)+T12we

T12b=x1(6)+T12be

TTb=2*x*y*T12b+(x**2)*T11b+(y**2)*T22b

TSb=x*T11b+y*T22b

FATb=1-(TSb/TTb)

RTb=T11b/T22b

write(1,*) 'Alleles sampled from different populations'

write(1,*) 'T11b= ',T11b,' T12b= ',T12b,' T22b= ',T22b

write(1,*) 'TTb= ',TTb,' TSb= ',TSb

write(1,*) 'FATb= ',FATb,' T11/T22= ',RTb

write(1,*) ''

TTw=2*x*y*T12w+(x**2)*T11w+(y**2)*T22w

TSw=x*T11w+y*T22w

FATw=1-(TSw/TTw)

RTw=T11w/T22w

write(1,*) 'Alleles sampled from the same population'

write(1,*) 'T11w= ',T11w,' T12w= ',T12w,' T22w= ',T22w

write(1,*) 'TTw= ',TTw,' TSw= ',TSw

write(1,*) 'FATw= ',FATw,' T11/T22= ',RTw

write(1,*) ''

icount=0

end if

do 20 j1=1,6

x2(j1)=0

do 25 k1=1,6

! write(*,*) 'j1= ',j1,'k1=',k1,'a=',a(j1,k1)

x2(j1)=x2(j1)+x1(k1)*a(j1,k1)

! matrix multiplication

25 continue

20 continue

! write(*,*)

delsum=0.0

do 40 i1=1,6

del(i1)=(x2(i1)-x1(i1))*tfac

delsum=delsum+del(i1)**2

x1(i1)=x1(i1)+del(i1)

! write(*,*) 'i=',i1,' x(i)=',x1(i1)

! accelerates changes

40 continue

! write(*,*)

! delsum=sqrt(delsum)

! if(delsum.le.0.0001) go to 50

! terminates run if changes are small

10 continue

write(1,*) 'Final state of population'

write(1,*) 'Number of iterations)= ',i

write(1,*) 'Alleles sampled from different populations'

write(1,*) 'T11b= ',T11b,' T12b= ',T12b,' T22b= ',T22b

write(1,*) 'TTb= ',TTb,' TSb= ',TSb

write(1,*) 'FATb= ',FATb,' T11/T22= ',RTb

write(1,*) ''

write(1,*) 'Alleles sampled from the same population'

write(1,*) 'T11w= ',T11w,' T12w= ',T12w,' T22w= ',T22w

write(1,*) 'TTw= ',TTw,' TSw= ',TSw

write(1,*) 'FATw= ',FATw,' T11/T22= ',RTw

write(1,*) ''

100 end program Invdiv4

**program Invdiv5**

! PROGRAM FOR NEUTRAL LD IN SUBDIVIVED POPULATION

! Linkage disequilibrium as function of gene conversion/recombination rate and Fst

real :: AM,d,m,m1,ANE,ANET,Fst,Fst1,delf,g,g1,delg

CHARACTER*20 FINP

CHARACTER*20 FOUT

WRITE (*,*) 'Input file?'

READ (*,*) FINP

OPEN (2,FILE=FINP)

WRITE (*,*) 'Output file?'

READ (*,*) FOUT

OPEN (1,FILE=FOUT)

write (1,*) 'Neutral LD in a subdivided population'

write (1,*) ''

READ (2,*) ANE

READ (2,*) d

READ (2,*) Fst

READ (2,*) delf

READ (2,*) ninc1

READ (2,*) g

READ (2,*) delg

READ (2,*) ninc2

write (1,*) 'Local Ne= ',ANE

ANET=d*ANE

write (1,*) 'Total Ne= ',ANET

write (1,*) 'Number of populations = ',d

write (1,*) 'Initial neutral Fst= ',Fst

write (1,*) 'Steps of FST= ',delf

write (1,*) 'Number of increments= ',ninc1

write (1,*) 'Initial recombination rate= ',g

write (1,*) 'Steps of recombination rate (ratios)= ',delg

write (1,*) 'Number of increments= ',ninc2

write (1,*) ''

write (*,*) 'Local Ne= ',ANE

write (*,*) 'Number of populations = ',d

write (*,*) 'Initial neutral Fst= ',Fst

write (*,*) 'Steps of FST= ',delf

write (*,*) 'Number of increments= ',ninc1

write (*,*) 'Initial recombination rate= ',g

write (*,*) 'Steps of recombination rate (ratios)= ',delg

write (*,*) 'Number of increments= ',ninc2

write (*,*) ''

write (*,*) 'CONT?'

read (*,*) CONT

! allows program to be cancelled if desired

if(CONT.eq.1) go to 100

!!!!!!!!!!!!!!!!!!!!!!!!!!!!!!!!!!!!!!!!!!!!!!!!!!!!!!!!!!!!!!!!!!!!!

do 20 j=1,ninc2

if(j.eq.1) then

g1=g

else

g1=g1*delg

end if

write(1,*) ''

write(1,*) 'Recombination rate= ',g1

rho=4*ANET*g1

write(1,*) 'Scaled value (multiplied by 4Nd)= ',rho

write(1,*) ''

write(1,*) 'Results for equivalent panmictic population and for scattered samples'

FAT=(rho+10)/(rho*(rho+13)+22)

write(1,*) 'Sigma^2-d= ',FAT

write(1,*) ''

write(*,*) 'Recombination rate= ',g1

write(*,*) 'Scaled value (multiplied by 2Nd)= ',rho

write (*,*) 'Results for equivalent panmictic population'

write(*,*) 'Sigma^2-d= ',FAT

write(*,*) ''

!!!!!!!!!!!!!!!!!!!!!!!!!!!!!!!!!!!!!!!!!!!!!!!!!!!!!!!!!!!!!!!!!!!!!!

write(1,*) 'Results for samples from within populations'

do 10 i=1,ninc1

write(1,*) ''

Fst1=Fst+delf*(i-1)

write(1,*) 'Fst= ',Fst1

AM=(1-Fst1)/Fst1

m=AM/(4*ANE)

write(1,*) 'Scaled migration rate= ',AM,' Migration rate= ',m

write (1,*) ''

AM1=1+AM

AM2=2+AM

AM3=3+AM

V5=4*AM1*(36+rho*(rho+14))/AM

V5=V5/(18+rho*(rho+13))

V4=4*AM1*(36+rho*(rho+14)+AM*(24+rho*(rho+13)))/(AM*AM2)

V4=V4/(18+rho*(rho+13))

V3=4*AM1*((AM**2)*(22+rho*(rho+13))+4*AM*(24+rho*(rho+13))+2*(36+rho*(rho+14)))

V3=V3/(AM*AM2*AM3*(18+rho*(rho+13)))

FAT=(V5-2*V4+V3)/V3

write(1,*) 'Sigma^2-d= ',FAT

write(1,*) ''

10 continue

20 continue

100 end program Invdiv5
