# Supplementary material for: The effects of inversion polymorphisms on patterns of neutral genetic diversity
Source: Genetics. 2023 Jun 22;224(4):iyad116. doi: 10.1093/genetics/iyad116 (PMC10411593; doi:10.1093/genetics/iyad116)

Figure S1. The equilibrium population statistics for an inversion polymorphism where the inversion is maintained at a constant frequency of 0.5, under the same assumptions as in Figure 1. The X axis is the equilibrium F_{ST} for neutral sites unlinked to the inversion. Subscripts 1 and 2 denote alleles sampled from the inversion and standard arrangement, respectively; subscripts *w* and *b* denote alleles sampled from the same and from separate demes, respectively; subscript *T* denotes pairs of alleles sampled without regard to karyotype. In this case, $T_{11w} = T_{22w} = T_{Sw} = 1$ (blue dashed line) for all values of F_{ST}. The solid black curve is T_{12}, the sold blue curve is T_{11b} and the solid and dashed red curves are T_{Tb} and T_{Tw}, respectively.

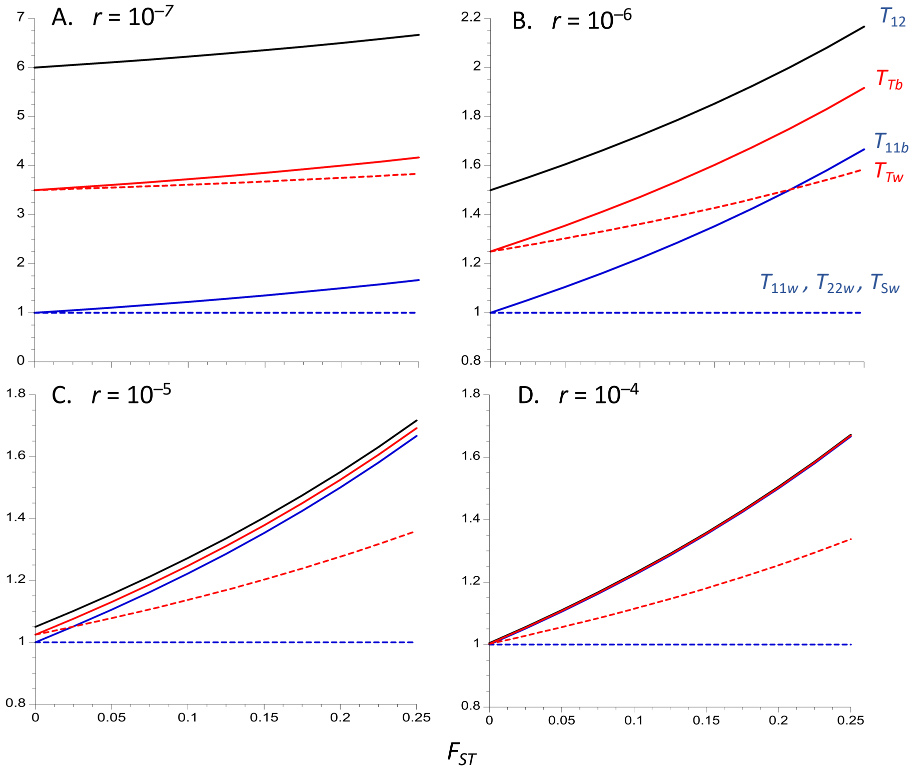

Figure S2. Equilibrium values of F_{ATw} (dashed curves) and F_{ATb} (blue solid curves), T_{11w}/T_{22w} (black dashed curves) and T_{11w}/T_{22w} (black solid curves), for an inversion polymorphism where the inversion is maintained at a constant frequency of 0.5. Four different recombination rates are modeled, with the higher rate for the panel corresponding to the black curves and the lower rate to the blue curves. The dashed curves are for within-deme samples and the solid curve for samples from separate demes. The population and recombination parameters in Figure 1 are used.

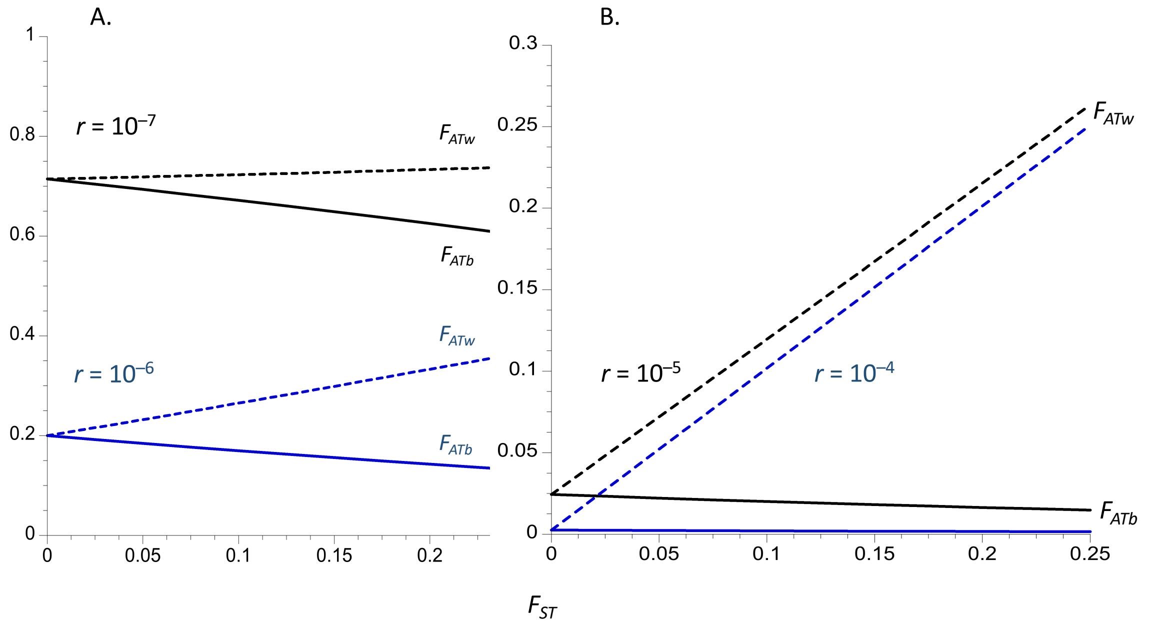

Supplement: iyad116_Supplementary_Data [file iyad116_supplementary_data.zip › Supplemental_Figures_GENETICS-2023-306220.pdf]
