# Supplementary material for: The effects of inversion polymorphisms on patterns of neutral genetic diversity
Source: Genetics. 2023 Jun 22;224(4):iyad116. doi: 10.1093/genetics/iyad116 (PMC10411593; doi:10.1093/genetics/iyad116)
Supplement: iyad116_Supplementary_Data [file iyad116_supplementary_data.zip › Supplemental_Tables_GENETICS-2023-306220.docx]

**SUPPLEMENTARY TABLES**

**Table S1**

**Equilibrium coalescent times for inversion and standard arrangements in a subdivided population**

**Inversion frequency= 0.1**

**Local Ne= 5000**

**Total Ne= 1000000**

**Number of populations = 200**

**Initial neutral Fst= 2.50E-002**

**Steps of FST= 2.5E-002**

**Initial gene conversion rate= 9.9999999999999995E-008**

**Steps of gene conversion rates (ratios)= 10.000000000000000**

**Number of increments= 4**

**Gene conversion rate= 9.9999999999999995E-008**

**Scaled value= 0.4**

Results for equivalent panmictic population

Times are scaled by 2Nd

T11= 0.305019289 T12= 6.00000000 T22= 1.07722008

TT= 1.95559847 TS= 1.00000000

FAT= 0.488647580 T11/T22= 0.283154100

Results for subdivided population

Fst= 2.5000000000000001E-002

Scaled migration rate= 39.000000000000000 Migration rate= 1.9499999999999999E-003

Alleles sampled from different populations

T11b= 0.38137720374659301 T12b= 6.0354084750480110 T22b= 1.1080786163037240

TTb= 1.9877309767521245 TSb= 1.0354084750480108

FATb= 0.47910029719422687 T11/T22= 0.344178826

Alleles sampled from the same population

T11w= 0.30383083563017776 T12w= 6.0355366801762163 T22w= 1.0775335022846340

TTw= 1.9622370476385744 TSw= 1.0001632356191883

FATw= 0.49029438781475454 T11/T22= 0.281968802

Fst= 5.0000000000000003E-002

Scaled migration rate= 18.999999999999996 Migration rate= 9.4999999999999978E-004

Alleles sampled from different populations

T11b= 0.46097604162330830 T12b= 6.0726091063843928 T22b= 1.1405683358022909

TTb= 2.0215397515652795 TSb= 1.0726091063843928

FATb= 0.46940983695528582 T11/T22= 0.404163450

Alleles sampled from the same population

T11w= 0.30250154450360134 T12w= 6.0728722642791295 T22w= 1.0778518531731356

TTw= 1.9692020240855193 TSw= 1.0003168223061822

FATw= 0.49201919860369792 T11/T22= 0.280652255

Fst= 7.5000000000000011E-002

Scaled migration rate= 12.333333333333332 Migration rate= 6.1666666666666662E-004

Alleles sampled from different populations

T11b= 0.54403543337370186 T12b= 6.1117432832507905 T22b= 1.1748219332371332

TTb= 2.0571599112409578 TSb= 1.1117432832507901

FATb= 0.45957371754335619 T11/T22= 0.463079065

Alleles sampled from the same population

T11w= 0.30106434379271213 T12w= 6.1121486886561955 T22w= 1.0781754924914673

TTw= 1.9765195563141309 TSw= 1.0004643776215918

FATw= 0.49382520682604025 T11/T22= 0.279235005

Fst= 0.10000000000000001

Scaled migration rate= 9.0000000000000000 Migration rate= 4.4999999999999999E-004

Alleles sampled from different populations

T11b= 0.63079614468311151 T12b= 6.1529677637253410 T22b= 1.2109868325078110

TTb= 2.0947414932487196 TSb= 1.1529677637253413

FATb= 0.44958947562679352 T11/T22= 0.520894289

Alleles sampled from the same population

T11w= 0.29953501588997822 T12w= 6.1535233192808967 T22w= 1.0785047630250379

TTw= 1.9842184056797421 TSw= 1.0006077883115321

FATw= 0.49571691027190645 T11/T22= 0.277731746

Fst= 0.12500000000000000

Scaled migration rate= 7.0000000000000000 Migration rate= 3.5000000000000000E-004

Alleles sampled from different populations

T11b= 0.72152279781838535 T12b= 6.1964568851710764 T22b= 1.2492273393213753

TTb= 2.1344516121592916 TSb= 1.1964568851710764

FATb= 0.43945466912660736 T11/T22= 0.577575266

Alleles sampled from the same population

T11w= 0.29792115202883995 T12w= 6.1971711708853618 T22w= 1.0788400325944165

TTw= 1.9923304486811313 TSw= 1.0007481445378590

FATw= 0.49769971883914788 T11/T22= 0.276149511

Fst= 0.14999999999999999

Scaled migration rate= 5.6666666666666670 Migration rate= 2.8333333333333335E-004

Alleles sampled from different populations

T11b= 0.81650702138672060 T12b= 6.2424051165106240 T22b= 1.2897271270799462

TTb= 2.1764769641205364 TSb= 1.2424051165106238

FATb= 0.42916688897157673 T11/T22= 0.633085072

Alleles sampled from the same population

T11w= 0.29622594389061691 T12w= 6.2432874694518006 T22w= 1.0791816964462790

TTw= 2.0008911780617167 TSw= 1.0008861211907130

FATw= 0.49977983202450749 T11/T22= 0.274491251

Fst= 0.17500000000000002

Scaled migration rate= 4.7142857142857135 Migration rate= 2.3571428571428569E-004

Alleles sampled from different populations

T11b= 0.91607112976378779 T12b= 6.2910300706023730 T22b= 1.3326921751399938

TTb= 2.2210267858694603 TSb= 1.2910300706023732

FATb= 0.41872377279908557 T11/T22= 0.687383890

Alleles sampled from the same population

T11w= 0.29444997359743752 T12w= 6.2920906766629789 T22w= 1.0795301799226118

TTw= 2.0099402672726265 TSw= 1.0010221592900943

FATw= 0.50196422471379021 T11/T22= 0.272757530

Fst= 0.20000000000000001

Scaled migration rate= 4.0000000000000000 Migration rate= 2.0000000000000001E-004

Alleles sampled from different populations

T11b= 1.0205724419113942 T12b= 6.3425760768012198 T22b= 1.3783542584556452

TTb= 2.2683363675924064 TSb= 1.3425760768012200

FATb= 0.40812302091412511 T11/T22= 0.740428269

Alleles sampled from the same population

T11w= 0.29259212624336850 T12w= 6.3438260768012196 T22w= 1.0798859414467172

TTw= 2.0195222276584945 TSw= 1.0011565599263825

FATw= 0.50426068789192835 T11/T22= 0.270947248

Fst= 0.22500000000000001

Scaled migration rate= 3.4444444444444446 Migration rate= 1.7222222222222224E-004

Alleles sampled from different populations

T11b= 1.1304083765953425 T12b= 6.3973184395208680 T22b= 1.4269751131792596

TTb= 2.3186712445549102 TSb= 1.3973184395208680

FATb= 0.39736241487348256 T11/T22= 0.792171061

Alleles sampled from the same population

T11w= 0.29065007993644509 T12w= 6.3987700524240934 T22w= 1.0802494758708006

TTw= 2.0296871856910501 TSw= 1.0012895362773651

FATw= 0.50667790419317504 T11/T22= 0.269058287

Fst= 0.25000000000000000

Scaled migration rate= 3.0000000000000000 Migration rate= 1.4999999999999999E-004

Alleles sampled from different populations

T11b= 1.2460224962953286 T12b= 6.4555685420506608 T22b= 1.4788514360234750

TTb= 2.3723322257110873 TSb= 1.4555685420506606

FATb= 0.38643983912735247 T11/T22= 0.842560947

Alleles sampled from the same population

T11w= 0.28862057604981289 T12w= 6.4572352087173277 T22w= 1.0806213182372864

TTw= 2.0404918111018193 TSw= 1.0014212440185390

FATw= 0.50922555112936507 T11/T22= 0.267087609

**Gene conversion rate= 9.9999999999999995E-007**

**Scaled value= 4**

Results for equivalent panmictic population

Times are scaled by 2Nd

T11= 0.470588237 T12= 1.50000000 T22= 1.05882359

TT= 1.13235295 TS= 1.00000000

FAT= 0.116883099 T11/T22= 0.444444418

Results for subdivided population

Fst= 2.5000000000000001E-002

Scaled migration rate= 39.000000000000000 Migration rate= 1.9499999999999999E-003

Alleles sampled from different populations

T11b= 0.56574155825635286 T12b= 1.5387529531343556 T22b= 1.0913097747874669

TTb= 1.1665938647245957 TSb= 1.0387529531343556

FATb= 0.10958476249180360 T11/T22= 0.518406034

Alleles sampled from the same population

T11w= 0.45056981166855803 T12w= 1.5388811582625608 T22w= 1.0612291716137028

TTw= 1.1410999356110458 TSw= 1.0001632356191883

FATw= 0.12350951533126464 T11/T22= 0.424573541

Fst= 5.0000000000000003E-002

Scaled migration rate= 18.999999999999996 Migration rate= 9.4999999999999978E-004

Alleles sampled from different populations

T11b= 0.65832852331905301 T12b= 1.5786582245972889 T22b= 1.1253615247393149

TTb= 1.2022846006995476 TSb= 1.0786582245972889

FATb= 0.10282621604762043 T11/T22= 0.584992945

Alleles sampled from the same population

T11w= 0.43180144630426170 T12w= 1.5789213824920256 T22w= 1.0634851974175064

TTw= 1.1499468732197875 TSw= 1.0003168223061818

FATw= 0.13011909888902073 T11/T22= 0.406024873

Fst= 7.5000000000000011E-002

Scaled migration rate= 12.333333333333332 Migration rate= 6.1666666666666662E-004

Alleles sampled from different populations

T11b= 0.74888586530068824 T12b= 1.6198965342727596 T22b= 1.1611199419363230

TTb= 1.2395773877905252 TSb= 1.1198965342727596

FATb= 9.6549723072223648E-002 T11/T22= 0.644968569

Alleles sampled from the same population

T11w= 0.41419070172254047 T12w= 1.6203019396781650 T22w= 1.0656058971659306

TTw= 1.1589370328636990 TSw= 1.0004643776215916

FATw= 0.13673965948825206 T11/T22= 0.388690323

Fst= 0.10000000000000001

Scaled migration rate= 9.0000000000000000 Migration rate= 4.4999999999999999E-004

Alleles sampled from different populations

T11b= 0.83791524787265947 T12b= 1.6626575463306998 T22b= 1.1987400239371486

TTb= 1.2786369302073430 TSb= 1.1626575463306998

FATb= 9.0705485769002503E-002 T11/T22= 0.698996663

Alleles sampled from the same population

T11w= 0.39764406476923775 T12w= 1.6632131018862555 T22w= 1.0676037575940089

TTw= 1.1681138426383657 TSw= 1.0006077883115319

FATw= 0.14339874095532978 T11/T22= 0.372464091

Fst= 0.12500000000000000

Scaled migration rate= 7.0000000000000000 Migration rate= 3.5000000000000000E-004

Alleles sampled from different populations

T11b= 0.92589287194396774 T12b= 1.7071429021188189 T22b= 1.2383929054715801

TTb= 1.3196429045328071 TSb= 1.2071429021188189

FATb= 8.5250337062825698E-002 T11/T22= 0.747656822

Alleles sampled from the same population

T11w= 0.38207353549231504 T12w= 1.7078571878331046 T22w= 1.0694897677651412

TTw= 1.1775217410546464 TSw= 1.0007481445378585

FATw= 0.15012342477724505 T11/T22= 0.357248425

Fst= 0.14999999999999999

Scaled migration rate= 5.6666666666666670 Migration rate= 2.8333333333333335E-004

Alleles sampled from different populations

T11b= 1.0132781434076163 T12b= 1.7535694354905325 T22b= 1.2802684679441898

TTb= 1.3627927388571659 TSb= 1.2535694354905325

FATb= 8.0146672529402996E-002 T11/T22= 0.791457534

Alleles sampled from the same population

T11w= 0.36739847738753667 T12w= 1.7544517884317090 T22w= 1.0712736371688434

TTw= 1.1872069527983462 TSw= 1.0008861211907127

FATw= 0.15694048216990275 T11/T22= 0.342954844

Fst= 0.17500000000000002

Scaled migration rate= 4.7142857142857135 Migration rate= 2.3571428571428569E-004

Alleles sampled from different populations

T11b= 1.1005217452258673 T12b= 1.8021727183541276 T22b= 1.3245783820350454

TTb= 1.4083047962043886 TSb= 1.3021727183541276

FATb= 7.5361582333813182E-002 T11/T22= 0.830846846

Alleles sampled from the same population

T11w= 0.35354580185227857 T12w= 1.8032333244147336 T22w= 1.0729639767831849

TTw= 1.1972182776075546 TSw= 1.0010221592900943

FATw= 0.16387664804911450 T11/T22= 0.329503894

Fst= 0.20000000000000001

Scaled migration rate= 4.0000000000000000 Migration rate= 2.0000000000000001E-004

Alleles sampled from different populations

T11b= 1.1880734150125345 T12b= 1.8532110592203399 T22b= 1.3715596863545405

TTb= 1.4564220707569646 TSb= 1.3532110592203399

FATb= 7.0866140804211719E-002 T11/T22= 0.866220713

Alleles sampled from the same population

T11w= 0.34044954712940856 T12w= 1.8544610592203399 T22w= 1.0745684502371573

TTw= 1.2076079308230527 TSw= 1.0011565599263825

FATw= 0.17095893926099170 T11/T22= 0.316824436

Fst= 0.22500000000000001

Scaled migration rate= 3.4444444444444446 Migration rate= 1.7222222222222224E-004

Alleles sampled from different populations

T11b= 1.2763897115508014 T12b= 1.9069700980303197 T22b= 1.4214790298613773

TTb= 1.5074165289486812 TSb= 1.4069700980303197

FATb= 6.6634821225169838E-002 T11/T22= 0.897930741

Alleles sampled from the same population

T11w= 0.32805025666057086 T12w= 1.9084217109335455 T22w= 1.0760939006792309

TTw= 1.2184324700848210 TSw= 1.0012895362773651

FATw= 0.17821499273762753 T11/T22= 0.304852813

Fst= 0.25000000000000000

Scaled migration rate= 3.0000000000000000 Migration rate= 1.4999999999999999E-004

Alleles sampled from different populations

T11b= 1.3659420538034992 T12b= 1.9637681699144667 T22b= 1.4746377383712408

TTb= 1.5615942592033440 TSb= 1.4637681699144667

FATb= 6.2645010835774895E-002 T11/T22= 0.926289916

Alleles sampled from the same population

T11w= 0.31629432009015995 T12w= 1.9654348365811334 T22w= 1.0775464577883584

TTw= 1.2297538445940761 TSw= 1.0014212440185386

FATw= 0.18567341877341870 T11/T22= 0.293531954

**Gene conversion rate= 9.9999999999999991E-006**

**Scaled value= 40**

Results for equivalent panmictic population

Times are scaled by 2Nd

T11= 0.843478262 T12= 1.04999995 T22= 1.01739132

TT= 1.02152169 TS= 1.00000000

FAT= 2.10682750E-02 T11/T22= 0.829059839

Results for subdivided population

Fst= 2.5000000000000001E-002

Scaled migration rate= 39.000000000000000 Migration rate= 1.9499999999999999E-003

Alleles sampled from different populations

T11b= 0.91963076207742589 T12b= 1.0951727126788890 T22b= 1.0591218174734314

TTb= 1.0642160680564536 TSb= 1.0451727119338310

FATb= 1.7894257279352122E-002 T11/T22= 0.868295550

Alleles sampled from the same population

T11w= 0.73223672899553449 T12w= 1.0953009178070943 T22w= 1.0299328474662610

TTw= 1.0387221389429038 TSw= 1.0001632356191883

FATw= 3.7121480209285296E-002 T11/T22= 0.710955799

Fst= 5.0000000000000003E-002

Scaled migration rate= 18.999999999999996 Migration rate= 9.4999999999999978E-004

Alleles sampled from different populations

T11b= 0.98689906185568399 T12b= 1.1387293529603277 T22b= 1.1000438289218903

TTb= 1.1058757755781472 TSb= 1.0887293522152697

FATb= 1.5504836747069040E-002 T11/T22= 0.897145212

Alleles sampled from the same population

T11w= 0.64707179913860624 T12w= 1.1389925108550645 T22w= 1.0395662693248011

TTw= 1.0535380480983867 TSw= 1.0003168223061816

FATw= 5.0516662296409942E-002 T11/T22= 0.622444034

Fst= 7.5000000000000011E-002

Scaled migration rate= 12.333333333333332 Migration rate= 6.1666666666666662E-004

Alleles sampled from different populations

T11b= 1.0487332071490028 T12b= 1.1818307575789446 T22b= 1.1410638179099848

TTb= 1.1474785609427878 TSb= 1.1318307568338866

FATb= 1.3636685373925150E-002 T11/T22= 0.919083774

Alleles sampled from the same population

T11w= 0.57977803975817688 T12w= 1.1822361629843501 T22w= 1.0472073040508603

TTw= 1.0668382060159616 TSw= 1.0004643776215920

FATw= 6.2215458745369046E-002 T11/T22= 0.553642094

Fst= 0.10000000000000001

Scaled migration rate= 9.0000000000000000 Migration rate= 4.4999999999999999E-004

Alleles sampled from different populations

T11b= 1.1073375644909476 T12b= 1.2252620999000052 T22b= 1.1828092696731694

TTb= 1.1896960620621777 TSb= 1.1752620991549472

FATb= 1.2132479351248060E-002 T11/T22= 0.936192811

Alleles sampled from the same population

T11w= 0.52526516211474261 T12w= 1.2258176554555609 T22w= 1.0534236356667306

TTw= 1.0791729744932002 TSw= 1.0006077883115319

FATw= 7.2801291394981371E-002 T11/T22= 0.498626709

Fst= 0.12500000000000000

Scaled migration rate= 7.0000000000000000 Migration rate= 3.5000000000000000E-004

Alleles sampled from different populations

T11b= 1.1642198205905956 T12b= 1.2696044426623936 T22b= 1.2257582887314178

TTb= 1.2330352117575853 TSb= 1.2196044419173355

FATb= 1.0892446308248926E-002 T11/T22= 0.949795604

Alleles sampled from the same population

T11w= 0.48020816140563233 T12w= 1.2703187283766793 T22w= 1.0585859204414396

TTw= 1.0909140482794248 TSw= 1.0007481445378590

FATw= 8.2651702839260599E-002 T11/T22= 0.453631729

Fst= 0.14999999999999999

Scaled migration rate= 5.6666666666666670 Migration rate= 2.8333333333333335E-004

Alleles sampled from different populations

T11b= 1.2204783583475174 T12b= 1.3153254767995568 T22b= 1.2703084891330523

TTb= 1.2779132456051678 TSb= 1.2653254760544987

FATb= 9.8502536020808051E-003 T11/T22= 0.960773230

Alleles sampled from the same population

T11w= 0.44234323598281999 T12w= 1.3162078297407334 T22w= 1.0629464417693679

TTw= 1.1023274595463484 TSw= 1.0008861211907132

FATw= 9.2024686019689939E-002 T11/T22= 0.416148186

Fst= 0.17500000000000002

Scaled migration rate= 4.7142857142857135 Migration rate= 2.3571428571428569E-004

Alleles sampled from different populations

T11b= 1.2769637031502592 T12b= 1.3628315644538418 T22b= 1.3168168815486196

TTb= 1.3247009926875761 TSb= 1.3128315637087837

FATb= 8.9600815914778753E-003 T11/T22= 0.969735205

Alleles sampled from the same population

T11w= 0.41007574953679243 T12w= 1.3638921705144478 T22w= 1.0666828714849057

TTw= 1.1136144740907423 TSw= 1.0010221592900945

FATw= 0.10110529040364580 T11/T22= 0.384440184

Fst= 0.20000000000000001

Scaled migration rate= 4.0000000000000000 Migration rate= 2.0000000000000001E-004

Alleles sampled from different populations

T11b= 1.3343750466778874 T12b= 1.4125000524520872 T22b= 1.3656250522658226

TTb= 1.3737500522434709 TSb= 1.3625000517070291

FATb= 8.1892630453912485E-003 T11/T22= 0.977116704

Alleles sampled from the same population

T11w= 0.38225001331950942 T12w= 1.4137500524520872 T22w= 1.0699239539938128

TTw= 1.1249359123095592 TSw= 1.0011565599263825

FATw= 0.11003235920262378 T11/T22= 0.357268393

Fst= 0.22500000000000001

Scaled migration rate= 3.4444444444444446 Migration rate= 1.7222222222222224E-004

Alleles sampled from different populations

T11b= 1.3933214948204005 T12b= 1.4647011257684095 T22b= 1.4170766394903458

TTb= 1.4254114955736981 TSb= 1.4147011250233514

FATb= 7.5138797348031083E-003 T11/T22= 0.983236492

Alleles sampled from the same population

T11w= 0.35800798625856733 T12w= 1.4661527386716353 T22w= 1.0727652640572314

TTw= 1.1364274367098375 TSw= 1.0012895362773651

FATw= 0.11891467599877825 T11/T22= 0.333724439

Fst= 0.25000000000000000

Scaled migration rate= 3.0000000000000000 Migration rate= 1.4999999999999999E-004

Alleles sampled from different populations

T11b= 1.4543634274213952 T12b= 1.5198140765479278 T22b= 1.4715308145119226

TTb= 1.4800501278074985 TSb= 1.4698140758028697

FATb= 6.9160171080098198E-003 T11/T22= 0.988333642

Alleles sampled from the same population

T11w= 0.33669925246352062 T12w= 1.5214807432145945 T22w= 1.0752792430802076

TTw= 1.1482097131982305 TSw= 1.0014212440185390

FATw= 0.12784116655033861 T11/T22= 0.313127279

**Gene conversion rate= 9.9999999999999991E-005**

**Scaled value= 400**

Results for equivalent panmictic population

Times are scaled by 2Nd

T11= 0.980540514 T12= 1.00500000 T22= 1.00216222

TT= 1.00245678 TS= 1.00000000

FAT= 2.45076418E-03 T11/T22= 0.978424966

Results for subdivided population

Fst= 2.5000000000000001E-002

Scaled migration rate= 39.000000000000000 Migration rate= 1.9499999999999999E-003

Alleles sampled from different populations

T11b= 1.0321719250317052 T12b= 1.0522142748688621 T22b= 1.0488856471971668

TTb= 1.0493176629564174 TSb= 1.0472142749806208

FATb= 2.0045292765494827E-003 T11/T22= 0.984065235

Alleles sampled from the same population

T11w= 0.82181030767343033 T12w= 1.0523424799970673 T22w= 1.0199802276131615

TTw= 1.0238237338428673 TSw= 1.0001632356191885

FATw= 2.3109933323063658E-002 T11/T22= 0.805711985

Fst= 5.0000000000000003E-002

Scaled migration rate= 18.999999999999996 Migration rate= 9.4999999999999978E-004

Alleles sampled from different populations

T11b= 1.0797019142532269 T12b= 1.0965738839777650 T22b= 1.0928929918491124

TTb= 1.0934236416563112 TSb= 1.0915738840895237

FATb= 1.6917116992143377E-003 T11/T22= 0.987930119

Alleles sampled from the same population

T11w= 0.70787366795078943 T12w= 1.0968370418725018 T22w= 1.0328105061234474

TTw= 1.0410859141765507 TSw= 1.0003168223061816

FATw= 3.9160160862050986E-002 T11/T22= 0.685385823

Fst= 7.5000000000000011E-002

Scaled migration rate= 12.333333333333332 Migration rate= 6.1666666666666662E-004

Alleles sampled from different populations

T11b= 1.1253962589364843 T12b= 1.1398820224649124 T22b= 1.1359359963144697

TTb= 1.1365408836477697 TSb= 1.1348820225766711

FATb= 1.4595700823136770E-003 T11/T22= 0.990721524

Alleles sampled from the same population

T11w= 0.62211435193932341 T12w= 1.1402874278703179 T22w= 1.0425032693640652

TTw= 1.0559005287209433 TSw= 1.0004643776215911

FATw= 5.2501300635301673E-002 T11/T22= 0.596750498

Fst= 0.10000000000000001

Scaled migration rate= 9.0000000000000000 Migration rate= 4.4999999999999999E-004

Alleles sampled from different populations

T11b= 1.1705967831583575 T12b= 1.1832215946533595 T22b= 1.1790687960547583

TTb= 1.1797315796735426 TSb= 1.1782215947651182

FATb= 1.2799393815008386E-003 T11/T22= 0.992814660

Alleles sampled from the same population

T11w= 0.55523005516772628 T12w= 1.1837771502089152 T22w= 1.0500942031052878

TTw= 1.0692084921045653 TSw= 1.0006077883115316

FATw= 6.4160268366372675E-002 T11/T22= 0.528743088

Fst= 0.12500000000000000

Scaled migration rate= 7.0000000000000000 Migration rate= 3.5000000000000000E-004

Alleles sampled from different populations

T11b= 1.2161888996821939 T12b= 1.2273217792744182 T22b= 1.2230032104643973

TTb= 1.2237124097423790 TSb= 1.2223217793861769

FATb= 1.1364029204335990E-003 T11/T22= 0.994428217

Alleles sampled from the same population

T11w= 0.50160719397276110 T12w= 1.2280360649887039 T22w= 1.0562082501562027

TTw= 1.0815912462642185 TSw= 1.0007481445378585

FATw= 7.4744596912733385E-002 T11/T22= 0.474913150

Fst= 0.14999999999999999

Scaled migration rate= 5.6666666666666670 Migration rate= 2.8333333333333335E-004

Alleles sampled from different populations

T11b= 1.2628175963166377 T12b= 1.2727277022104917 T22b= 1.2682732696562071

TTb= 1.2690205107825827 TSb= 1.2677277023222504

FATb= 1.0187451261406411E-003 T11/T22= 0.995698333

Alleles sampled from the same population

T11w= 0.45765742843973589 T12w= 1.2736100551516683 T22w= 1.0612448648297104

TTw= 1.0934347247237632 TSw= 1.0008861211907130

FATw= 8.4640263785687786E-002 T11/T22= 0.431245834

Fst= 0.17500000000000002

Scaled migration rate= 4.7142857142857135 Migration rate= 2.3571428571428569E-004

Alleles sampled from different populations

T11b= 1.3109978869022936 T12b= 1.3198875660674814 T22b= 1.3153197527655673

TTb= 1.3160987405012794 TSb= 1.3148875661792401

FATb= 9.2027618047718640E-004 T11/T22= 0.996714234

Alleles sampled from the same population

T11w= 0.42097990549618208 T12w= 1.3209481721280874 T22w= 1.0654712986005281

TTw= 1.1050122219044454 TSw= 1.0010221592900936

FATw= 9.4107613067961338E-002 T11/T22= 0.395111442

Fst= 0.20000000000000001

Scaled migration rate= 4.0000000000000000 Migration rate= 2.0000000000000001E-004

Alleles sampled from different populations

T11b= 1.3611765220585990 T12b= 1.3692017325718235 T22b= 1.3645378671974693

TTb= 1.3653437495134644 TSb= 1.3642017326835822

FATb= 8.3643172665426313E-004 T11/T22= 0.997536659

Alleles sampled from the same population

T11w= 0.38990757771399837 T12w= 1.3704517325718235 T22w= 1.0690731135055362

TTw= 1.1165296095795527 TSw= 1.0011565599263825

FATw= 0.10333183165345317 T11/T22= 0.364715546

Fst= 0.22500000000000001

Scaled migration rate= 3.4444444444444446 Migration rate= 1.7222222222222224E-004

Alleles sampled from different populations

T11b= 1.4137695802674959 T12b= 1.4210530644452839 T22b= 1.4163067850336588

TTb= 1.4171357432800900 TSb= 1.4160530645570426

FATb= 7.6399083727951567E-004 T11/T22= 0.998208582

Alleles sampled from the same population

T11w= 0.36324675195162487 T12w= 1.4225046773485097 T22w= 1.0721831789802243

TTw= 1.1281516844162298 TSw= 1.0012895362773644

FATw= 0.11245132183134676 T11/T22= 0.338791698

Fst= 0.25000000000000000

Scaled migration rate= 3.0000000000000000 Migration rate= 1.4999999999999999E-004

Alleles sampled from different populations

T11b= 1.4691876030438937 T12b= 1.4758276945370936 T22b= 1.4710099270494033

TTb= 1.4718589019571326 TSb= 1.4708276946488523

FATb= 7.0061560038747750E-004 T11/T22= 0.998761177

Alleles sampled from the same population

T11w= 0.34012021606871257 T12w= 1.4774943612037603 T22w= 1.0748991360129636

TTw= 1.1400184873478647 TSw= 1.0014212440185386

FATw= 0.12157455766507641 T11/T22= 0.316420585

**Inversion frequency= 0.5**

**Gene conversion rate= 9.9999999999999995E-008**

**Scaled value= 0.4**

Results for equivalent panmictic population

Times are scaled by 2Nd

T11= 1.00000000 T12= 6.00000000 T22= 1.00000000

TT= 3.50000000 TS= 1.00000000

FAT= 0.714285731 T11/T22= 1.00000000

Results for subdivided population

Fst= 2.5000000000000001E-002

Scaled migration rate= 39.000000000000000 Migration rate= 1.9499999999999999E-003

Alleles sampled from different populations

T11b= 1.0512820512820513 T12b= 6.0512820512820511 T22b= 1.0512820512820513

TTb= 3.5512820512820511 TSb= 1.0512820512820513

FATb= 0.70397111913357402 T11/T22= 1.00000000

Alleles sampled from the same population

T11w= 1.0002439024390244 T12w= 6.0514102564102563 T22w= 1.0002439024390244

TTw= 3.5258270794246402 TSw= 1.0002439024390244

FATw= 0.71630942757344518 T11/T22= 1.00000000

Fst= 5.0000000000000003E-002

Scaled migration rate= 18.999999999999996 Migration rate= 9.4999999999999978E-004

Alleles sampled from different populations

T11b= 1.1052631578947367 T12b= 6.1052631578947363 T22b= 1.1052631578947367

TTb= 3.6052631578947363 TSb= 1.1052631578947367

FATb= 0.69343065693430650 T11/T22= 1.00000000

Alleles sampled from the same population

T11w= 1.0004761904761903 T12w= 6.1055263157894730 T22w= 1.0004761904761903

TTw= 3.5530012531328317 TSw= 1.0004761904761903

FATw= 0.71841378057662431 T11/T22= 1.00000000

Fst= 7.5000000000000011E-002

Scaled migration rate= 12.333333333333332 Migration rate= 6.1666666666666662E-004

Alleles sampled from different populations

T11b= 1.1621621621621623 T12b= 6.1621621621621623 T22b= 1.1621621621621623

TTb= 3.6621621621621623 TSb= 1.1621621621621623

FATb= 0.68265682656826565 T11/T22= 1.00000000

Alleles sampled from the same population

T11w= 1.0006976744186047 T12w= 6.1625675675675673 T22w= 1.0006976744186047

TTw= 3.5816326209930862 TSw= 1.0006976744186047

FATw= 0.72060292600832432 T11/T22= 1.00000000

Fst= 0.10000000000000001

Scaled migration rate= 9.0000000000000000 Migration rate= 4.4999999999999999E-004

Alleles sampled from different populations

T11b= 1.2222222222222221 T12b= 6.2222222222222223 T22b= 1.2222222222222221

TTb= 3.7222222222222219 TSb= 1.2222222222222221

FATb= 0.67164179104477606 T11/T22= 1.00000000

Alleles sampled from the same population

T11w= 1.0009090909090907 T12w= 6.2227777777777780 T22w= 1.0009090909090907

TTw= 3.6118434343434340 TSw= 1.0009090909090907

FATw= 0.72288137370743000 T11/T22= 1.00000000

Fst= 0.12500000000000000

Scaled migration rate= 7.0000000000000000 Migration rate= 3.5000000000000000E-004

Alleles sampled from different populations

T11b= 1.2857142857142858 T12b= 6.2857142857142856 T22b= 1.2857142857142858

TTb= 3.7857142857142860 TSb= 1.2857142857142858

FATb= 0.66037735849056611 T11/T22= 1.00000000

Alleles sampled from the same population

T11w= 1.0011111111111111 T12w= 6.2864285714285710 T22w= 1.0011111111111111

TTw= 3.6437698412698412 TSw= 1.0011111111111111

FATw= 0.72525402132363359 T11/T22= 1.00000000

Fst= 0.14999999999999999

Scaled migration rate= 5.6666666666666670 Migration rate= 2.8333333333333335E-004

Alleles sampled from different populations

T11b= 1.3529411764705881 T12b= 6.3529411764705879 T22b= 1.3529411764705881

TTb= 3.8529411764705883 TSb= 1.3529411764705881

FATb= 0.64885496183206115 T11/T22= 1.00000000

Alleles sampled from the same population

T11w= 1.0013043478260868 T12w= 6.3538235294117644 T22w= 1.0013043478260868

TTw= 3.6775639386189258 TSw= 1.0013043478260868

FATw= 0.72772618925502153 T11/T22= 1.00000000

Fst= 0.17500000000000002

Scaled migration rate= 4.7142857142857135 Migration rate= 2.3571428571428569E-004

Alleles sampled from different populations

T11b= 1.4242424242424241 T12b= 6.4242424242424239 T22b= 1.4242424242424241

TTb= 3.9242424242424239 TSb= 1.4242424242424241

FATb= 0.63706563706563712 T11/T22= 1.00000000

Alleles sampled from the same population

T11w= 1.0014893617021274 T12w= 6.4253030303030298 T22w= 1.0014893617021274

TTw= 3.7133961960025785 TSw= 1.0014893617021274

FATw= 0.73030366035807937 T11/T22= 1.00000000

Fst= 0.20000000000000001

Scaled migration rate= 4.0000000000000000 Migration rate= 2.0000000000000001E-004

Alleles sampled from different populations

T11b= 1.5000000000000000 T12b= 6.5000000000000000 T22b= 1.5000000000000000

TTb= 4.0000000000000000 TSb= 1.5000000000000000

FATb= 0.62500000000000000 T11/T22= 1.00000000

Alleles sampled from the same population

T11w= 1.0016666666666667 T12w= 6.5012499999999998 T22w= 1.0016666666666667

TTw= 3.7514583333333333 TSw= 1.0016666666666667

FATw= 0.73299272505136892 T11/T22= 1.00000000

Fst= 0.22500000000000001

Scaled migration rate= 3.4444444444444446 Migration rate= 1.7222222222222224E-004

Alleles sampled from different populations

T11b= 1.5806451612903225 T12b= 6.5806451612903221 T22b= 1.5806451612903225

TTb= 4.0806451612903221 TSb= 1.5806451612903225

FATb= 0.61264822134387353 T11/T22= 1.00000000

Alleles sampled from the same population

T11w= 1.0018367346938775 T12w= 6.5820967741935474 T22w= 1.0018367346938775

TTw= 3.7919667544437128 TSw= 1.0018367346938775

FATw= 0.73580023255218430 T11/T22= 1.00000000

Fst= 0.25000000000000000

Scaled migration rate= 3.0000000000000000 Migration rate= 1.4999999999999999E-004

Alleles sampled from different populations

T11b= 1.6666666666666667 T12b= 6.6666666666666670 T22b= 1.6666666666666667

TTb= 4.1666666666666670 TSb= 1.6666666666666667

FATb= 0.60000000000000009 T11/T22= 1.00000000

Alleles sampled from the same population

T11w= 1.0020000000000000 T12w= 6.6683333333333339 T22w= 1.0020000000000000

TTw= 3.8351666666666673 TSw= 1.0020000000000000

FATw= 0.73873364912433193 T11/T22= 1.00000000

**Gene conversion rate= 9.9999999999999995E-007**

**Scaled value= 4**

Results for equivalent panmictic population

Times are scaled by 2Nd

T11= 1.00000000 T12= 1.50000000 T22= 1.00000000

TT= 1.25000000 TS= 1.00000000

FAT= 0.199999988 T11/T22= 1.00000000

Results for subdivided population

Fst= 2.5000000000000001E-002

Scaled migration rate= 39.000000000000000 Migration rate= 1.9499999999999999E-003

Alleles sampled from different populations

T11b= 1.0512820512820513 T12b= 1.5512820512820513 T22b= 1.0512820512820513

TTb= 1.3012820512820513 TSb= 1.0512820512820513

FATb= 0.19211822660098521 T11/T22= 1.00000000

Alleles sampled from the same population

T11w= 1.0002439024390244 T12w= 1.5514102564102565 T22w= 1.0002439024390244

TTw= 1.2758270794246405 TSw= 1.0002439024390244

FATw= 0.21600354893697327 T11/T22= 1.00000000

Fst= 5.0000000000000003E-002

Scaled migration rate= 18.999999999999996 Migration rate= 9.4999999999999978E-004

Alleles sampled from different populations

T11b= 1.1052631578947369 T12b= 1.6052631578947369 T22b= 1.1052631578947369

TTb= 1.3552631578947369 TSb= 1.1052631578947369

FATb= 0.18446601941747576 T11/T22= 1.00000000

Alleles sampled from the same population

T11w= 1.0004761904761905 T12w= 1.6055263157894737 T22w= 1.0004761904761905

TTw= 1.3030012531328321 TSw= 1.0004761904761905

FATw= 0.23217557306969161 T11/T22= 1.00000000

Fst= 7.5000000000000011E-002

Scaled migration rate= 12.333333333333332 Migration rate= 6.1666666666666662E-004

Alleles sampled from different populations

T11b= 1.1621621621621621 T12b= 1.6621621621621621 T22b= 1.1621621621621621

TTb= 1.4121621621621621 TSb= 1.1621621621621621

FATb= 0.17703349282296654 T11/T22= 1.00000000

Alleles sampled from the same population

T11w= 1.0006976744186045 T12w= 1.6625675675675675 T22w= 1.0006976744186045

TTw= 1.3316326209930860 TSw= 1.0006976744186045

FATw= 0.24851820341235076 T11/T22= 1.00000000

Fst= 0.10000000000000001

Scaled migration rate= 9.0000000000000000 Migration rate= 4.4999999999999999E-004

Alleles sampled from different populations

T11b= 1.2222222222222221 T12b= 1.7222222222222221 T22b= 1.2222222222222221

TTb= 1.4722222222222221 TSb= 1.2222222222222221

FATb= 0.16981132075471694 T11/T22= 1.00000000

Alleles sampled from the same population

T11w= 1.0009090909090907 T12w= 1.7227777777777777 T22w= 1.0009090909090907

TTw= 1.3618434343434342 TSw= 1.0009090909090907

FATw= 0.26503365536167933 T11/T22= 1.00000000

Fst= 0.12500000000000000

Scaled migration rate= 7.0000000000000000 Migration rate= 3.5000000000000000E-004

Alleles sampled from different populations

T11b= 1.2857142857142860 T12b= 1.7857142857142860 T22b= 1.2857142857142860

TTb= 1.5357142857142860 TSb= 1.2857142857142860

FATb= 0.16279069767441856 T11/T22= 1.00000000

Alleles sampled from the same population

T11w= 1.0011111111111113 T12w= 1.7864285714285717 T22w= 1.0011111111111113

TTw= 1.3937698412698416 TSw= 1.0011111111111113

FATw= 0.28172422629046501 T11/T22= 1.00000000

Fst= 0.14999999999999999

Scaled migration rate= 5.6666666666666670 Migration rate= 2.8333333333333335E-004

Alleles sampled from different populations

T11b= 1.3529411764705879 T12b= 1.8529411764705879 T22b= 1.3529411764705879

TTb= 1.6029411764705879 TSb= 1.3529411764705879

FATb= 0.15596330275229364 T11/T22= 1.00000000

Alleles sampled from the same population

T11w= 1.0013043478260868 T12w= 1.8538235294117644 T22w= 1.0013043478260868

TTw= 1.4275639386189258 TSw= 1.0013043478260868

FATw= 0.29859229367002449 T11/T22= 1.00000000

Fst= 0.17500000000000002

Scaled migration rate= 4.7142857142857135 Migration rate= 2.3571428571428569E-004

Alleles sampled from different populations

T11b= 1.4242424242424243 T12b= 1.9242424242424243 T22b= 1.4242424242424243

TTb= 1.6742424242424243 TSb= 1.4242424242424243

FATb= 0.14932126696832582 T11/T22= 1.00000000

Alleles sampled from the same population

T11w= 1.0014893617021277 T12w= 1.9253030303030303 T22w= 1.0014893617021277

TTw= 1.4633961960025790 TSw= 1.0014893617021277

FATw= 0.31564031364998657 T11/T22= 1.00000000

Fst= 0.20000000000000001

Scaled migration rate= 4.0000000000000000 Migration rate= 2.0000000000000001E-004

Alleles sampled from different populations

T11b= 1.5000000000000004 T12b= 2.0000000000000004 T22b= 1.5000000000000004

TTb= 1.7500000000000004 TSb= 1.5000000000000004

FATb= 0.14285714285714279 T11/T22= 1.00000000

Alleles sampled from the same population

T11w= 1.0016666666666669 T12w= 2.0012500000000006 T22w= 1.0016666666666669

TTw= 1.5014583333333338 TSw= 1.0016666666666669

FATw= 0.33287082003607604 T11/T22= 1.00000000

Fst= 0.22500000000000001

Scaled migration rate= 3.4444444444444446 Migration rate= 1.7222222222222224E-004

Alleles sampled from different populations

T11b= 1.5806451612903225 T12b= 2.0806451612903225 T22b= 1.5806451612903225

TTb= 1.8306451612903225 TSb= 1.5806451612903225

FATb= 0.13656387665198233 T11/T22= 1.00000000

Alleles sampled from the same population

T11w= 1.0018367346938775 T12w= 2.0820967741935483 T22w= 1.0018367346938775

TTw= 1.5419667544437128 TSw= 1.0018367346938775

FATw= 0.35028642361663320 T11/T22= 1.00000000

Fst= 0.25000000000000000

Scaled migration rate= 3.0000000000000000 Migration rate= 1.4999999999999999E-004

Alleles sampled from different populations

T11b= 1.6666666666666665 T12b= 2.1666666666666665 T22b= 1.6666666666666665

TTb= 1.9166666666666665 TSb= 1.6666666666666665

FATb= 0.13043478260869568 T11/T22= 1.00000000

Alleles sampled from the same population

T11w= 1.0020000000000000 T12w= 2.1683333333333330 T22w= 1.0020000000000000

TTw= 1.5851666666666664 TSw= 1.0020000000000000

FATw= 0.36788981179686664 T11/T22= 1.00000000

**Gene conversion rate= 9.9999999999999991E-006**

**Scaled value= 40**

Results for equivalent panmictic population

Times are scaled by 2Nd

T11= 1.00000000 T12= 1.04999995 T22= 1.00000000

TT= 1.02499998 TS= 1.00000000

FAT= 2.43902206E-02 T11/T22= 1.00000000

Results for subdivided population

Fst= 2.5000000000000001E-002

Scaled migration rate= 39.000000000000000 Migration rate= 1.9499999999999999E-003

Alleles sampled from different populations

T11b= 1.0512820512820511 T12b= 1.1012820520271092 T22b= 1.0512820512820511

TTb= 1.0762820516545801 TSb= 1.0512820512820511

FATb= 2.3228112309497506E-002 T11/T22= 1.00000000

Alleles sampled from the same population

T11w= 1.0002439024390242 T12w= 1.1014102571553144 T22w= 1.0002439024390242

TTw= 1.0508270797971693 TSw= 1.0002439024390242

FATw= 4.8136537714567385E-002 T11/T22= 1.00000000

Fst= 5.0000000000000003E-002

Scaled migration rate= 18.999999999999996 Migration rate= 9.4999999999999978E-004

Alleles sampled from different populations

T11b= 1.1052631578947367 T12b= 1.1552631586397948 T22b= 1.1052631578947367

TTb= 1.1302631582672658 TSb= 1.1052631578947367

FATb= 2.2118743046402489E-002 T11/T22= 1.00000000

Alleles sampled from the same population

T11w= 1.0004761904761903 T12w= 1.1555263165345315 T22w= 1.0004761904761903

TTw= 1.0780012535053609 TSw= 1.0004761904761903

FATw= 7.1915559260326112E-002 T11/T22= 1.00000000

Fst= 7.5000000000000011E-002

Scaled migration rate= 12.333333333333332 Migration rate= 6.1666666666666662E-004

Alleles sampled from different populations

T11b= 1.1621621621621629 T12b= 1.2121621629072210 T22b= 1.1621621621621629

TTb= 1.1871621625346920 TSb= 1.1621621621621629

FATb= 2.1058622959437945E-002 T11/T22= 1.00000000

Alleles sampled from the same population

T11w= 1.0006976744186054 T12w= 1.2125675683126265 T22w= 1.0006976744186054

TTw= 1.1066326213656159 TSw= 1.0006976744186054

FATw= 9.5727294588771383E-002 T11/T22= 1.00000000

Fst= 0.10000000000000001

Scaled migration rate= 9.0000000000000000 Migration rate= 4.4999999999999999E-004

Alleles sampled from different populations

T11b= 1.2222222222222221 T12b= 1.2722222229672802 T22b= 1.2222222222222221

TTb= 1.2472222225947511 TSb= 1.2222222222222221

FATb= 2.0044543722544050E-002 T11/T22= 1.00000000

Alleles sampled from the same population

T11w= 1.0009090909090907 T12w= 1.2727777785228358 T22w= 1.0009090909090907

TTw= 1.1368434347159633 TSw= 1.0009090909090907

FATw= 0.11957173666647891 T11/T22= 1.00000000

Fst= 0.12500000000000000

Scaled migration rate= 7.0000000000000000 Migration rate= 3.5000000000000000E-004

Alleles sampled from different populations

T11b= 1.2857142857142860 T12b= 1.3357142864593441 T22b= 1.2857142857142860

TTb= 1.3107142860868151 TSb= 1.2857142857142860

FATb= 1.9073569761086095E-002 T11/T22= 1.00000000

Alleles sampled from the same population

T11w= 1.0011111111111113 T12w= 1.3364285721736298 T22w= 1.0011111111111113

TTw= 1.1687698416423706 TSw= 1.0011111111111113

FATw= 0.14344888493671526 T11/T22= 1.00000000

Fst= 0.14999999999999999

Scaled migration rate= 5.6666666666666670 Migration rate= 2.8333333333333335E-004

Alleles sampled from different populations

T11b= 1.3529411764705885 T12b= 1.4029411772156466 T22b= 1.3529411764705885

TTb= 1.3779411768431176 TSb= 1.3529411764705885

FATb= 1.8143009870569604E-002 T11/T22= 1.00000000

Alleles sampled from the same population

T11w= 1.0013043478260872 T12w= 1.4038235301568232 T22w= 1.0013043478260872

TTw= 1.2025639389914553 TSw= 1.0013043478260872

FATw= 0.16735874462871125 T11/T22= 1.00000000

Fst= 0.17500000000000002

Scaled migration rate= 4.7142857142857135 Migration rate= 2.3571428571428569E-004

Alleles sampled from different populations

T11b= 1.4242424242424243 T12b= 1.4742424249874824 T22b= 1.4242424242424243

TTb= 1.4492424246149533 TSb= 1.4242424242424243

FATb= 1.7250392306981555E-002 T11/T22= 1.00000000

Alleles sampled from the same population

T11w= 1.0014893617021277 T12w= 1.4753030310480884 T22w= 1.0014893617021277

TTw= 1.2383961963751080 TSw= 1.0014893617021277

FATw= 0.19130132615589990 T11/T22= 1.00000000

Fst= 0.20000000000000001

Scaled migration rate= 4.0000000000000000 Migration rate= 2.0000000000000001E-004

Alleles sampled from different populations

T11b= 1.4999999999999996 T12b= 1.5500000007450576 T22b= 1.4999999999999996

TTb= 1.5250000003725286 TSb= 1.4999999999999996

FATb= 1.6393442863227525E-002 T11/T22= 1.00000000

Alleles sampled from the same population

T11w= 1.0016666666666663 T12w= 1.5512500007450576 T22w= 1.0016666666666663

TTw= 1.2764583337058619 TSw= 1.0016666666666663

FATw= 0.21527664459004325 T11/T22= 1.00000000

Fst= 0.22500000000000001

Scaled migration rate= 3.4444444444444446 Migration rate= 1.7222222222222224E-004

Alleles sampled from different populations

T11b= 1.5806451612903223 T12b= 1.6306451620353803 T22b= 1.5806451612903223

TTb= 1.6056451616628513 TSb= 1.5806451612903223

FATb= 1.5570065522221843E-002 T11/T22= 1.00000000

Alleles sampled from the same population

T11w= 1.0018367346938772 T12w= 1.6320967749386062 T22w= 1.0018367346938772

TTw= 1.3169667548162416 TSw= 1.0018367346938772

FATw= 0.23928471920032246 T11/T22= 1.00000000

Fst= 0.25000000000000000

Scaled migration rate= 3.0000000000000000 Migration rate= 1.4999999999999999E-004

Alleles sampled from different populations

T11b= 1.6666666666666676 T12b= 1.7166666674117257 T22b= 1.6666666666666676

TTb= 1.6916666670391967 TSb= 1.6666666666666676

FATb= 1.4778325340112475E-002 T11/T22= 1.00000000

Alleles sampled from the same population

T11w= 1.0020000000000004 T12w= 1.7183333340783924 T22w= 1.0020000000000004

TTw= 1.3601666670391965 TSw= 1.0020000000000004

FATw= 0.26332557304822901 T11/T22= 1.00000000

**Gene conversion rate= 9.9999999999999991E-005**

**Scaled value= 400**

Results for equivalent panmictic population

Times are scaled by 2Nd

T11= 1.00000000 T12= 1.00500000 T22= 1.00000000

TT= 1.00250006 TS= 1.00000000

FAT= 2.49379873E-03 T11/T22= 1.00000000

Results for subdivided population

Fst= 2.5000000000000001E-002

Scaled migration rate= 39.000000000000000 Migration rate= 1.9499999999999999E-003

Alleles sampled from different populations

T11b= 1.0512820512820491 T12b= 1.0562820511702904 T22b= 1.0512820512820491

TTb= 1.0537820512261697 TSb= 1.0512820512820491

FATb= 2.3724070278210441E-003 T11/T22= 1.00000000

Alleles sampled from the same population

T11w= 1.0002439024390222 T12w= 1.0564102562984956 T22w= 1.0002439024390222

TTw= 1.0283270793687589 TSw= 1.0002439024390222

FATw= 2.7309576391760126E-002 T11/T22= 1.00000000

Fst= 5.0000000000000003E-002

Scaled migration rate= 18.999999999999996 Migration rate= 9.4999999999999978E-004

Alleles sampled from different populations

T11b= 1.1052631578947376 T12b= 1.1102631577829789 T22b= 1.1052631578947376

TTb= 1.1077631578388583 TSb= 1.1052631578947376

FATb= 2.2568000446936409E-003 T11/T22= 1.00000000

Alleles sampled from the same population

T11w= 1.0004761904761912 T12w= 1.1105263156777156 T22w= 1.0004761904761912

TTw= 1.0555012530769534 TSw= 1.0004761904761912

FATw= 5.2131688560629774E-002 T11/T22= 1.00000000

Fst= 7.5000000000000011E-002

Scaled migration rate= 12.333333333333332 Migration rate= 6.1666666666666662E-004

Alleles sampled from different populations

T11b= 1.1621621621621583 T12b= 1.1671621620503996 T22b= 1.1621621621621583

TTb= 1.1646621621062789 TSb= 1.1621621621621583

FATb= 2.1465451746104369E-003 T11/T22= 1.00000000

Alleles sampled from the same population

T11w= 1.0006976744186014 T12w= 1.1675675674558050 T22w= 1.0006976744186014

TTw= 1.0841326209372033 TSw= 1.0006976744186014

FATw= 7.6960092250037304E-002 T11/T22= 1.00000000

Fst= 0.10000000000000001

Scaled migration rate= 9.0000000000000000 Migration rate= 4.4999999999999999E-004

Alleles sampled from different populations

T11b= 1.2222222222222190 T12b= 1.2272222221104603 T22b= 1.2222222222222190

TTb= 1.2247222221663396 TSb= 1.2222222222222190

FATb= 2.0412791560999732E-003 T11/T22= 1.00000000

Alleles sampled from the same population

T11w= 1.0009090909090883 T12w= 1.2277777776660159 T22w= 1.0009090909090883

TTw= 1.1143434342875520 TSw= 1.0009090909090883

FATw= 0.10179477878019461 T11/T22= 1.00000000

Fst= 0.12500000000000000

Scaled migration rate= 7.0000000000000000 Migration rate= 3.5000000000000000E-004

Alleles sampled from different populations

T11b= 1.2857142857142825 T12b= 1.2907142856025238 T22b= 1.2857142857142825

TTb= 1.2882142856584031 TSb= 1.2857142857142825

FATb= 1.9406708743668899E-003 T11/T22= 1.00000000

Alleles sampled from the same population

T11w= 1.0011111111111086 T12w= 1.2914285713168094 T22w= 1.0011111111111086

TTw= 1.1462698412139591 TSw= 1.0011111111111086

FATw= 0.12663574045455117 T11/T22= 1.00000000

Fst= 0.14999999999999999

Scaled migration rate= 5.6666666666666670 Migration rate= 2.8333333333333335E-004

Alleles sampled from different populations

T11b= 1.3529411764705910 T12b= 1.3579411763588323 T22b= 1.3529411764705910

TTb= 1.3554411764147116 TSb= 1.3529411764705910

FATb= 1.8444178822525359E-003 T11/T22= 1.00000000

Alleles sampled from the same population

T11w= 1.0013043478260890 T12w= 1.3588235293000088 T22w= 1.0013043478260890

TTw= 1.1800639385630489 TSw= 1.0013043478260890

FATw= 0.15148297045212100 T11/T22= 1.00000000

Fst= 0.17500000000000002

Scaled migration rate= 4.7142857142857135 Migration rate= 2.3571428571428569E-004

Alleles sampled from different populations

T11b= 1.4242424242424292 T12b= 1.4292424241306705 T22b= 1.4242424242424292

TTb= 1.4267424241865498 TSb= 1.4242424242424292

FATb= 1.7522433634410417E-003 T11/T22= 1.00000000

Alleles sampled from the same population

T11w= 1.0014893617021310 T12w= 1.4303030301912765 T22w= 1.0014893617021310

TTw= 1.2158961959467036 TSw= 1.0014893617021310

FATw= 0.17633646273367465 T11/T22= 1.00000000

Fst= 0.20000000000000001

Scaled migration rate= 4.0000000000000000 Migration rate= 2.0000000000000001E-004

Alleles sampled from different populations

T11b= 1.5000000000000056 T12b= 1.5049999998882468 T22b= 1.5000000000000056

TTb= 1.5024999999441262 TSb= 1.5000000000000056

FATb= 1.6638934736862288E-003 T11/T22= 1.00000000

Alleles sampled from the same population

T11w= 1.0016666666666703 T12w= 1.5062499998882468 T22w= 1.0016666666666703

TTw= 1.2539583332774586 TSw= 1.0016666666666703

FATw= 0.20119621195975157 T11/T22= 1.00000000

Fst= 0.22500000000000001

Scaled migration rate= 3.4444444444444446 Migration rate= 1.7222222222222224E-004

Alleles sampled from different populations

T11b= 1.5806451612903316 T12b= 1.5856451611785729 T22b= 1.5806451612903316

TTb= 1.5831451612344523 TSb= 1.5806451612903316

FATb= 1.5791350062752674E-003 T11/T22= 1.00000000

Alleles sampled from the same population

T11w= 1.0018367346938832 T12w= 1.5870967740817987 T22w= 1.0018367346938832

TTw= 1.2944667543878410 TSw= 1.0018367346938832

FATw= 0.22606221341879407 T11/T22= 1.00000000

Fst= 0.25000000000000000

Scaled migration rate= 3.0000000000000000 Migration rate= 1.4999999999999999E-004

Alleles sampled from different populations

T11b= 1.6666666666666630 T12b= 1.6716666665549043 T22b= 1.6666666666666630

TTb= 1.6691666666107836 TSb= 1.6666666666666630

FATb= 1.4977533365178042E-003 T11/T22= 1.00000000

Alleles sampled from the same population

T11w= 1.0019999999999978 T12w= 1.6733333332215710 T22w= 1.0019999999999978

TTw= 1.3376666666107844 TSw= 1.0019999999999978

FATw= 0.25093446296397415 T11/T22= 1.00000000

**Table S2**

**Neutral coalescent times for inversion and standard arrangements in a coalescent simulation of approach to recombination-drift equilibrium in a single population.**

**Times scaled by 2 x population size**

The italic numbers in brackets are the *T_ij_* values from the iterations of Equations (A3)

The standard deviations for the equilibrium results were obtained by the procedure described in section 8 of the Supplementary Information. These can be compared with the first set of results from the coalescent simulations, where the time from the initial state is chosen to be close to equilibrium.

For an inversion frequency of 0.5, only the equilibrium results are displayed.

**Inversion frequency= 0.1**

**Scaled recombination rate= 40**

**Number of replicates= 1000000**

Initial state of population

T11= 0.00000000 T12= 1.00000000 T22b= 1.00000000

Equilibrium results for panmictic population

T11= 0.843478322 T12= 1.04999995 T22= 1.01739132

s.d.= 1.00314 s.d.= 1.02252 s.d.= 1.02181

Time to origin of inversion= 4

Mean of T11= 0.842840254 (*0.84298*) s.d.= 1.00316882

s.e.= 1.00316887E-03

Mean of T12= 1.04935372 (*1.04937*) s.d.= 1.01939905

s.e.= 1.01939903E-03

Mean of T22= 1.01615655 (*1.01679*) s.d.= 1.01933658

s.e.= 1.01933663E-03

Time to origin of inversion= 2

Mean of T11= 0.839714766 (*0.83988*) s.d.= 0.992495000

s.e.= 9.92494985E-04

Mean of T12= 1.04427683 (*1.04550*) s.d.= 1.00812614

s.e.= 1.00812619E-03

Mean of T22= 1.01287293 (*1.01304*) s.d.= 1.00943804

s.e.= 1.00943807E-03

Time to origin of inversion= 1

Mean of T11= 0.832606018 (*0.83413*) s.d.= 0.983978033

s.e.= 9.83978040E-04

Mean of T12= 1.03788888 (*1.03832*) s.d.= 1.00350165

s.e.= 1.00350170E-03

Mean of T22= 1.00672770 (*1.00608*) s.d.= 1.00149477

s.e.= 1.00149482E-03

Time to origin of inversion= 0.5

Mean of T11= 0.827529371 (*0.82824*) s.d.= 0.982901752

s.e.= 9.82901780E-04

Mean of T12= 1.03096688 (*1.03094*) s.d.= 1.00062251

s.e.= 1.00062252E-03

Mean of T22= 0.998158276 (*0.99895*) s.d.= 0.997037828

s.e.= 9.97037860E-04

Time to origin of inversion= 0.25

Mean of T11= 0.822732747 (*0.82352*) s.d.= 0.980459869

s.e.= 9.80459852E-04

Mean of T12= 1.02539289 (*1.02532*) s.d.= 0.998387218

s.e.= 9.98387230E-04

Mean of T22= 0.993745387 (*0.99392*) s.d.= 0.998511076

s.e.= 9.98511096E-04

Time to origin of inversion= 0.125

Mean of T11= 0.811701477 (*0.81268*) s.d.= 0.976616263

s.e.= 9.76616284E-04

Mean of T12= 1.01626980 (*1.01772*) s.d.= 0.994884372

s.e.= 9.94884409E-04

Mean of T22= 0.991116047 (*0.99480*) s.d.= 0.998467326

s.e.= 9.98467323E-04

Time to origin of inversion= 6.25E-02

Mean of T11= 0.758589447 (*0.758545*) s.d.= 0.961394668

s.e.= 9.61394690E-04

Mean of T12= 1.00485098 (*1.00452*) s.d.= 0.997913659

s.e.= 9.97913652E-04

Mean of T22= 0.993107736 (*0.99345*) s.d.= 0.998382032

s.e.= 9.98381991E-04

**Inversion frequency= 0.1**

**Scaled recombination rate= 4.0**

**Number of replicates= 1000000**

Initial state of population

T11= 0.00000000 T12= 1.00000000 T22b= 1.00000000

Equilibrium results for panmictic population

T11= 0.470588297 T12= 1.50000000 T22= 1.05882359

s.d.= 0.91885 s.d.= 1.23073 s.d.= 1.12996

Time to origin of inversion= 20

Mean of T11= 0.469264179 s.d.= 0.915292263

s.e.= 9.15292243E-04

Mean of T12= 1.50086200 s.d.= 1.23265827

s.e.= 1.23265828E-03

Mean of T22= 1.05968940 s.d.= 1.13081312

Time to origin of inversion= 1

Mean of T11= 0.434916764 (*0.43413*) s.d.= 0.809637189

s.e.= 8.09637189E-04

Mean of T12= 1.37598729 (*1.37391*) s.d.= 1.05607760

s.e.= 1.05607766E-03

Mean of T22= 1.00097787 (*1.00080*) s.d.= 1.01011753

Time to origin of inversion= 0.5

Mean of T11= 0.401043385 (*0.40122*) s.d.= 0.756564558

s.e.= 7.56564550E-04

Mean of T12= 1.26687658 (*1.26656*) s.d.= 1.01439202

s.e.= 1.01439201E-03

Mean of T22= 0.984599292 (*0.98489*) s.d.= 0.996770203

s.e.= 9.96770221E-04

Time to origin of inversion= 0.25

Mean of T11= 0.359776527 (*0.35102*) s.d.= 0.706095636

s.e.= 7.06095656E-04

Mean of T12= 1.16544998 (*1.15114*) s.d.= 1.00426865

s.e.= 1.00426865E-03

Mean of T22= 0.983902514 (*0.985288*) s.d.= 0.997553110

s.e.= 9.97553114E-04

Time to origin of inversion= 0.125

Mean of T11= 0.288712919 (*0.28931*) s.d.= 0.632238030

s.e.= 6.32238050E-04

Mean of T12= 1.09267497 (*1.09138*) s.d.= 1.00096273

s.e.= 1.00096269E-03

Mean of T22= 0.988179803 (*0.98956*) s.d.= 0.996701419

s.e.= 9.96701419E-04

Time to origin of inversion= 6.25E-02

Mean of T11= 0.198425397 (*0.19780*) s.d.= 0.533404410

s.e.= 5.33404411E-04

Mean of T12= 1.04694235 (*1.04796*) s.d.= 0.999398291

s.e.= 9.99398297E-04

Mean of T22= 0.993636668 (*0.99397*) s.d.= 0.999168098

s.e.= 9.99168144E-04

**Inversion frequency= 0.1**

**Scaled recombination rate= 0.4**

**Number of replicates= 1000000**

Initial state of population

T11= 0.00000000 T12= 1.00000000 T22b= 1.00000000

Equilibrium results for panmictic population

T11= 0.305019408 T12= 6.00000000 T22= 1.07722008

s.d.= 1.47875 s.d.= 5.28310 s.d.= 1.71233

Time to origin of inversion= 100

Mean of T11= 0.305183679 s.d.= 1.48238468

s.e.= 1.48238463E-03

Mean of T12= 6.00524044 s.d.= 5.29317570

s.e.= 5.29317558E-03

Mean of T22= 1.07521260 s.d.= 1.70390737

s.e.= 1.70390739E-03

Time to origin of inversion= 4

Mean of T11= 0.221483573 (*0.22321*) s.d.= 0.742023706

s.e.= 7.42023694E-04

Mean of T12= 3.68989038 (*3.68930*) s.d.= 1.76187861

s.e.= 1.76187861E-03

Mean of T22= 0.981181800 (*0.98215*) s.d.= 1.08416986

s.e.= 1.08416984E-03

Time to origin of inversion= 2

Mean of T11= 0.184408382 (*0.18488*) s.d.= 0.520189285

s.e.= 5.20189293E-04

Mean of T12= 2.60776067 (*2.60694*) s.d.= 1.18764746

s.e.= 1.18764746E-03

Mean of T22= 0.947988510 (*0.94868*) s.d.= 0.982948661

s.e.= 9.82948695E-04

Time to origin of inversion= 1

Mean of T11= 0.159522906 (*0.15929*) s.d.= 0.393521845

s.e.= 3.93521856E-04

Mean of T12= 1.88445938 (*1.88484*) s.d.= 1.03488922

s.e.= 1.03488925E-03

Mean of T22= 0.946950376 (*0.94684*) s.d.= 0.978458345

s.e.= 9.78458324E-04

Time to origin of inversion= 0.5

Mean of T11= 0.143433765 (*0.143705*) s.d.= 0.326370001

s.e.= 3.26370005E-04

Mean of T12= 1.46712434 (*1.46544*) s.d.= 1.00711167

s.e.= 1.00711163E-03

Mean of T22= 0.961967230 (*0.96170*) s.d.= 0.992745221

s.e.= 9.92745277E-04

Time to origin of inversion= 0.25

Mean of T11= 0.126929134 (*0.12679*) s.d.= 0.280853540

s.e.= 2.80853536E-04

Mean of T12= 1.24095392 (*1.23881*) s.d.= 1.00302839

s.e.= 1.00302836E-03

Mean of T22= 0.976458669 (*0.97696*) s.d.= 0.997133672

s.e.= 9.97133669E-04

Time to origin of inversion= 0.125

Mean of T11= 9.67181697E-02 (*0.097163*) s.d.= 0.231224895

s.e.= 2.31224898E-04

Mean of T12= 1.12050974 (*1.12093*) s.d.= 1.00053322

s.e.= 1.00053323E-03

Mean of T22= 0.986687541 (*0.98736*) s.d.= 0.996315181

s.e.= 9.96315153E-04

Time to origin of inversion= 6.25E-02

Mean of T11= 6.32091165E-02 (0.06311) s.d.= 0.183888689

s.e.= 1.83888682E-04

Mean of T12= 1.06051600 (*1.06083*) s.d.= 0.997987986

s.e.= 9.97988041E-04

Mean of T22= 0.992533922 (*0.99337*) s.d.= 0.998949587

s.e.= 9.98949632E-04

**Inversion frequency= 0.5**

**Scaled recombination rate= 40**

**Number of replicates= 1000000**

Initial state of population

T11= 0.00000000 T12= 1.00000000 T22b= 1.00000000

Equilibrium results

T11= 1.00000000 T12= 1.04999995 T22= 1.00000000

s.d. = 1.02470 s.d. = 1.02590 s.d. = 1.02470

Time to origin of inversion= 10

Mean of T11= 1.00014043 s.d.= 1.02391338

s.e.= 1.02391338E-03

Mean of T12= 1.05008972 s.d.= 1.02473772

s.e.= 1.02473772E-03

Mean of T22= 1.00084615 s.d.= 1.02373850

s.e.= 1.02373853E-03

**Scaled recombination rate= 4**

**Number of replicates= 1000000**

Initial state of population

T11= 0.00000000 T12= 1.00000000 T22b= 1.00000000

Equilibrium results

T11= 1.00000000 T12= 1.50000000 T22= 1.00000000

s.d. = 1.22474 s.d. = 1.32288 s.d. = 1.22474

Time to origin of inversion= 20.0000000

Mean of T11= 1.00003147 s.d.= 1.22361922

s.e.= 1.22361921E-03

Mean of T12= 1.49854517 s.d.= 1.32191551

s.e.= 1.32191554E-03

Mean of T22= 0.998929322 s.d.= 1.22275007

**Scaled recombination rate= 0.4**

**Number of replicates= 1000000**

Initial state of population

T11= 0.00000000 T12= 1.00000000 T22b= 1.00000000

Equilibrium results

T11= 1.00000000 T12= 6.00000000 T22= 1.00000000

s.d. = 2.44949 s.d. = 5.56776 s.d. = 2.44949

Time to origin of inversion= 100.000000

Mean of T11= 0.999278069 s.d.= 2.44362330

s.e.= 2.44362326E-03

Mean of T12= 6.01205254 s.d.= 5.57754183

s.e.= 5.57754189E-03

Mean of T22= 1.00279987 s.d.= 2.45711279

s.e.= 2.45711277E-03

**Table S3**

**Coalescent times for inversion and standard arrangements in a panmictic population**

**Time course of approach to equilibrium: instantaneous sweep to equilibrium frequency**

**Times scaled by 2x population size**

**Inversion frequency = 0.1**

**Initial state of population**

T11= 0 T12= 1 T22= 1

TT= 0.99 TS= 0.9

FAT= 0.0909 T11/T22= 0

**Scaled recombination rate= 40**

Equilibrium results for panmictic population

T11= 0.84347826086956512 T12= 1.0500000000000000 T22= 1.0173913043478262

TT= 1.0215217391304350 TS= 1.0000000000000002

FAT= 2.1068312406895107E-002 T11/T22= 0.829059839

Time (relative to 2Ne) = 2.49999994E-03

T11= 8.5726814840451304E-002 T12= 0.99780845886065672 T22= 0.99971854494364520

TT= 0.99023481214767539 TS= 0.90831937193332579

FAT= 8.2723248273494754E-002 T11/T22= 8.57509524E-02

Time (relative to 2Ne) = 4.99999989E-03

T11= 0.16361435090221210 T12= 0.99606660101951916 T22= 0.99941541048695215

TT= 0.99045461418696679 TS= 0.91583530452847817

FAT= 7.5338444174689712E-002 T11/T22= 0.163710058

Time (relative to 2Ne) = 7.49999983E-03

T11= 0.23290197718569094 T12= 0.99475505471399739 T22= 0.99910096911829749

TT= 0.99065671460619742 TS= 0.92248106992503687

FAT= 6.8818636845621617E-002 T11/T22= 0.233111545

Time (relative to 2Ne) = 9.99999978E-03

T11= 0.29455892399112360 T12= 0.99381284030198780 T22= 0.99877933641844285

TT= 0.99084316299320774 TS= 0.92835729517571097

FAT= 6.3063328437101163E-002 T11/T22= 0.294918925

Time (relative to 2Ne) = 1.25000002E-02

T11= 0.34944415596201400 T12= 0.99318657855016379 T22= 0.99845400759923375

TT= 0.99101577185402900 TS= 0.93355302243551186

FAT= 5.7983688101162834E-002 T11/T22= 0.349985242

Time (relative to 2Ne) = 1.49999997E-02

T11= 0.39831897135329936 T12= 0.99282958725101933 T22= 0.99812793641015540

TT= 0.99117614391094233 TS= 0.93814703990446990

FAT= 5.3501190814815525E-002 T11/T22= 0.399066061

Time (relative to 2Ne) = 1.75000001E-02

T11= 0.44185815878531814 T12= 0.99270108310330007 T22= 0.99780360458980444

TT= 0.99132569626418887 TS= 0.94220906000935578

FAT= 4.9546416924255210E-002 T11/T22= 0.442830801

Time (relative to 2Ne) = 1.99999996E-02

T11= 0.48065987679121175 T12= 0.99276547669695592 T22= 0.99748308296784505

TT= 0.99146568177731864 TS= 0.94580076235018173

FAT= 4.6057992996063390E-002 T11/T22= 0.481872708

Time (relative to 2Ne) = 2.25000009E-02

T11= 0.51525440251372479 T12= 0.99299174984272298 T22= 0.99716808519511302

TT= 0.99159720800486906 TS= 0.94897671692697427

FAT= 4.2981657001282647E-002 T11/T22= 0.516717732

Time (relative to 2Ne) = 2.50000004E-02

T11= 0.54611187912881620 T12= 0.99335290572473900 T22= 0.99686001496636045

TT= 0.99172125394449318 TS= 0.95178520138260614

FAT= 4.0269432971255292E-002 T11/T22= 0.547832072

Time (relative to 2Ne) = 2.74999999E-02

T11= 0.57364917671887905 T12= 0.99382548345065569 T22= 0.99656000749999019

TT= 0.99183868486329896 TS= 0.95426892442187905

FAT= 3.7878902098477885E-002 T11/T22= 0.575629354

Time (relative to 2Ne) = 2.99999993E-02

T11= 0.59823596816702584 T12= 0.99438912954385117 T22= 0.99626896595052827

TT= 0.99195026541949149 TS= 0.95646566617217799

FAT= 3.5772558851332348E-002 T11/T22= 0.600476384

Time (relative to 2Ne) = 3.24999988E-02

T11= 0.62020011000053987 T12= 0.99502621978102890 T22= 0.99598759335118836

TT= 0.99205667127505326 TS= 0.95840884501612345

FAT= 3.3917242062072472E-002 T11/T22= 0.622698605

Time (relative to 2Ne) = 3.50000001E-02

T11= 0.63983240780325579 T12= 0.99572152553850002 T22= 0.99571642061452781

TT= 0.99215849937273015 TS= 0.96012801933340064

FAT= 3.2283632161171916E-002 T11/T22= 0.642584980

Time (relative to 2Ne) = 3.75000015E-02

T11= 0.65739083669026221 T12= 0.99646191948311369 T22= 0.99545583105783708

TT= 0.99225627703071129 TS= 0.96164933162107968

FAT= 3.0845806792194508E-002 T11/T22= 0.660391748

Time (relative to 2Ne) = 3.99999991E-02

T11= 0.67310427925833771 T12= 0.99723611603915108 T22= 0.99520608186562654

TT= 0.99235046999078813 TS= 0.96299590160489767

FAT= 2.9580847970135937E-002 T11/T22= 0.676346600

Time (relative to 2Ne) = 4.25000004E-02

T11= 0.68717583627197942 T12= 0.99803444258941043 T22= 0.99496732285356138

TT= 0.99244148954019851 TS= 0.96418817419540315

FAT= 2.8468494760215290E-002 T11/T22= 0.690651655

Time (relative to 2Ne) = 4.50000018E-02

T11= 0.69978575901149753 T12= 0.99884863783503786 T22= 0.99473961285572399

TT= 0.99252969881355835 TS= 0.96524422747130134

FAT= 2.7490836168301391E-002 T11/T22= 0.703486383

Time (relative to 2Ne) = 4.74999994E-02

T11= 0.71109404660240794 T12= 0.99967167415135738 T22= 0.99452293401952330

TT= 0.99261541836908229 TS= 0.96618004527781176

FAT= 2.6632039561409582E-002 T11/T22= 0.715010226

Time (relative to 2Ne) = 5.00000007E-02

T11= 0.72124274668096389 T12= 1.0004976011421889 T22= 0.99431720425934633

TT= 0.99269893112247432 TS= 0.96700975850150805

FAT= 2.5878110488059769E-002 T11/T22= 0.725364864

Time (relative to 2Ne) = 7.50000030E-02

T11= 0.78083186370440705 T12= 1.0081449104967588 T22= 0.99281607100865399

TT= 0.99345542004347043 TS= 0.97161765027822922

FAT= 2.1981630302329691E-002 T11/T22= 0.786481917

Time (relative to 2Ne) = 0.100000001

T11= 0.80316560291563410 T12= 1.0138366644900647 T22b= 0.99212304923408579

TT= 0.99414192551697755 TS= 0.97322730460224061

FAT= 2.1037862278930453E-002 T11/T22= 0.809542298

Time (relative to 2Ne) = 0.200000003

T11= 0.82156346127234769 T12= 1.0234661375495657 T22= 0.99289221368984448

TT= 0.99668223246041943 TS= 0.97575933844809482

FAT= 2.0992542388032942E-002 T11/T22= 0.827444732

Time (relative to 2Ne) = 0.300000012

T11= 0.82477839311595191 T12= 1.0267098071118019 T22= 0.99498708188995566

TT= 0.99899508554214800 TS= 0.97796621301255526

FAT= 2.1050026005063338E-002 T11/T22= 0.828933775

Time (relative to 2Ne) = 0.400000006

T11= 0.82665421811487083 T12= 1.0289710259826508 T22= 0.99705342140387965

TT= 1.0010945981951684 TS= 0.98001350107497875

FAT= 2.1058047019927861E-002 T11/T22= 0.829097211

Time (relative to 2Ne) = 0.500000000

T11= 0.82823793111655786 T12= 1.0309417748340661 T22= 0.99894663940391648

TT= 1.0029986766984700 TS= 0.98187576857518066

FAT= 2.1059756721533041E-002 T11/T22= 0.829111278

Time (relative to 2Ne) = 1.00000000

T11= 0.83413601959982364 T12= 1.0383166122559258 T22= 1.0060828945181461

TT= 1.0101654949617633 TS= 0.98888820702631397

FAT= 2.1063170383041840E-002 T11/T22= 0.829092741

Time (relative to 2Ne) = 2.00000000

T11= 0.83996674456235199 T12= 1.0456085049078498 T22= 1.0131407541192006

TT= 1.0172532091655890 TS= 0.99582335316351567

FAT= 2.1066393115290682E-002 T11/T22= 0.829072118

Time (relative to 2Ne) = 3.00000000

T11= 0.84215836915566711 T12= 1.0483493461295206 T22= 1.0157936281488731

TT= 1.0199173047954577 TS= 0.99843010224955253

FAT= 2.1067592877262120E-002 T11/T22= 0.829064429

Time (relative to 2Ne) = 4.00000000

T11= 0.84298214637542535 T12= 1.0493795602310934 T22= 1.0167907775690785

TT= 1.0209186721363048 TS= 0.99940991444971317

FAT= 2.1068042218861494E-002 T11/T22= 0.829061568

Time (relative to 2Ne) = 5.00000000

T11= 0.84329178376034175 T12= 1.0497667921096450 T22= 1.0171655812556069

TT= 1.0212950612343812 TS= 0.99977820150608032

FAT= 2.1068210887355776E-002 T11/T22= 0.829060495

**Scaled recombination rate= 4**

**Equilibrium results for panmictic population**

T11= 0.47058823529411764 T12= 1.5000000000000000 T22= 1.0588235294117647

TT= 1.1323529411764706 TS= 1.0000000000000000

FAT= 0.11688311688311681 T11/T22= 0.444444448

Time (relative to 2Ne) = 2.49999994E-03

T11= 1.1096388746625274E-002 T12= 1.0019573916219786 T22= 0.99972920969789181

TT= 0.99024395423471490 TS= 0.90086592760276518

FAT= 9.0258593601839521E-002 T11/T22= 1.10993944E-02

Time (relative to 2Ne) = 4.99999989E-03

T11= 2.2061826857566136E-002 T12= 1.0039491496460526 T22= 0.99945589562227066

TT= 0.99049074065890441 TS= 0.90171648874580013

FAT= 8.9626533867493752E-002 T11/T22= 2.20738370E-02

Time (relative to 2Ne) = 9.99999978E-03

T11= 4.2957098298150698E-002 T12= 1.0079152229510406 T22= 0.99891829789310815

TT= 0.99097813240758648 TS= 0.90332217793361247

FAT= 8.8453974520117273E-002 T11/T22= 4.30036150E-02

Time (relative to 2Ne) = 1.49999997E-02

T11= 6.2547103243070057E-002 T12= 1.0118572106048804 T22= 0.99839262578598165

TT= 0.99145779582795435 TS= 0.90480807353169057

FAT= 8.7396279156697343E-002 T11/T22= 6.26478046E-02

Time (relative to 2Ne) = 1.99999996E-02

T11= 8.0917250165951804E-002 T12= 1.0157742022575573 T22= 0.99787874062625104

TT= 0.99193030881528321 TS= 0.90618259158022108

FAT= 8.6445304143872082E-002 T11/T22= 8.10892582E-02

Time (relative to 2Ne) = 2.50000004E-02

T11= 9.8147315928966861E-002 T12= 1.0196653775491908 T22= 0.99737650305918069

TT= 0.99239620859608046 TS= 0.90745358434615930

FAT= 8.5593459058139199E-002 T11/T22= 9.84054804E-02

Time (relative to 2Ne) = 2.99999993E-02

T11= 0.11431181763479226 T12= 1.0235299997910292 T22= 0.99688577322805361

TT= 0.99285599445345663 TS= 0.90862837766872750

FAT= 8.4833668986502353E-002 T11/T22= 0.114668921

Time (relative to 2Ne) = 3.99999991E-02

T11= 0.14371795930429515 T12= 1.0311770217392162 T22= 0.99593827581189653

TT= 0.99375904691373806 TS= 0.91071624416113639

FAT= 8.3564323776978977E-002 T11/T22= 0.144304082

Time (relative to 2Ne) = 5.00000007E-02

T11= 0.16963925249512640 T12= 1.0387108335837751 T22= 0.99503512543134032

TT= 0.99464279416941659 TS= 0.91249553813771889

FAT= 8.2589706086691472E-002 T11/T22= 0.170485690

Time (relative to 2Ne) = 5.99999987E-02

T11= 0.19251508886647206 T12= 1.0461280009197025 T22= 0.99417520040601193

TT= 0.99551010338308088 TS= 0.91400918925205787

FAT= 8.1868495210701830E-002 T11/T22= 0.193643019

Time (relative to 2Ne) = 7.00000003E-02

T11= 0.21272886993363155 T12= 1.0534259508902317 T22= 0.99335738399240858

TT= 0.99636344089342910 TS= 0.91529453258653093

FAT= 8.1364796197464395E-002 T11/T22= 0.214151397

Time (relative to 2Ne) = 7.99999982E-02

T11= 0.23061515578359398 T12= 1.0606028515965615 T22= 0.99258056748593937

TT= 0.99720492450882792 TS= 0.91638402631570492

FAT= 8.1047431883603305E-002 T11/T22= 0.232338980

Time (relative to 2Ne) = 9.00000036E-02

T11= 0.24646590070432836 T12= 1.0676575071793162 T22= 0.99184365282943165

TT= 0.99803636909115989 TS= 0.91730587761692128

FAT= 8.0889328259404092E-002 T11/T22= 0.248492688

Time (relative to 2Ne) = 0.100000001

T11= 0.26053589236279495 T12= 1.0745892665629801 T22= 0.99114555479365429

TT= 0.99885932628782448 TS= 0.91808458855056840

FAT= 8.0866980576182357E-002 T11/T22= 0.262863398

Time (relative to 2Ne) = 0.125000000

T11= 0.28931064339766055 T12= 1.0913807367104837 T22= 0.98956314665074274

TT= 1.0008877878289653 TS= 0.91953789632543459

FAT= 8.1277734120412726E-002 T11/T22= 0.292361975

Time (relative to 2Ne) = 0.150000006

T11= 0.31104005589704598 T12= 1.1074107960196478 T22= 0.98820043141187752

TT= 1.0028866932861278 TS= 0.92048439386039438

FAT= 8.2165113942960333E-002 T11/T22= 0.314754009

Time (relative to 2Ne) = 0.200000003

T11= 0.34069054043478508 T12= 1.1372620828375040 T22= 0.98607270202473729

TT= 1.0068329689551359 TS= 0.92153448586574205

FAT= 8.4719596715147616E-002 T11/T22= 0.345502466

Time (relative to 2Ne) = 0.250000000

T11= 0.35940459663122504 T12= 1.1643454646626152 T22= 0.98464752588842397

TT= 1.0107407255752063 TS= 0.92212323296270415

FAT= 8.7675791001763148E-002 T11/T22= 0.365008384

Time (relative to 2Ne) = 0.300000012

T11= 0.37223517319045663 T12= 1.1889030065057464 T22= 0.98382278648362020

TT= 1.0146213499546715 TS= 0.92266402515430390

FAT= 9.0632160267942075E-002 T11/T22= 0.378355920

Time (relative to 2Ne) = 0.400000006

T11= 0.38936411401406135 T12= 1.2314107142395252 T22= 0.98362599538920847

TT= 1.0222846259685141 TS= 0.92419980725169382

FAT= 9.5946682778188630E-002 T11/T22= 0.395845681

Time (relative to 2Ne) = 0.500000000

T11= 0.40121561499171421 T12= 1.2665651575900454 T22= 0.98489003475322467

TT= 1.0297548126662375 TS= 0.92652259277707372

FAT= 0.10024932014823540 T11/T22= 0.407370985

Time (relative to 2Ne) = 0.600000024

T11= 0.41044710066362017 T12= 1.2958318227580001 T22= 0.98716589404185329

TT= 1.0369585732769775 TS= 0.92949401470403004

FAT= 0.10363437975476675 T11/T22= 0.415783316

Time (relative to 2Ne) = 0.699999988

T11= 0.41799191972638633 T12= 1.3203758383637625 T22= 0.99011608719144228

TT= 1.0438416007278095 TS= 0.93290367044493672

FAT= 0.10627851026968294 T11/T22= 0.422164559

Time (relative to 2Ne) = 0.800000012

T11= 0.42428825490456684 T12= 1.3411150229725710 T22= 0.99348884295676865

TT= 1.0503695494790912 TS= 0.93656878415154843

FAT= 0.10834354954785186 T11/T22= 0.427068979

Time (relative to 2Ne) = 1.00000000

T11= 0.43413407272776527 T12= 1.3739150447213688 T22= 1.0008069464758820

TT= 1.0622996754225884 TS= 0.94413965910107034

FAT= 0.11123039859210482 T11/T22= 0.433784038

Time (relative to 2Ne) = 1.50000000

T11= 0.44913323565582680 T12= 1.4248435669841952 T22= 1.0182733227874903

TT= 1.0857645658715804 TS= 0.96135931407432396

FAT= 0.11457847834386836 T11/T22= 0.441073358

Time (relative to 2Ne) = 2.00000000

T11= 0.45721668779238622 T12= 1.4528724214711399 T22= 1.0316116156145867

TT= 1.1016946113905444 TS= 0.97417212283236676

FAT= 0.11575121384792875 T11/T22= 0.443206221

Time (relative to 2Ne) = 3.00000000

T11= 0.46504524975310890 T12= 1.4803878342590027 T22= 1.0470295291892042

TT= 1.1192141813074070 TS= 0.98883110124559470

FAT= 0.11649520014972081 T11/T22= 0.444156766

Time (relative to 2Ne) = 4.00000000

T11= 0.46823519454943191 T12= 1.4916686502297680 T22= 1.0537775155125058

TT= 1.1267424965519823 TS= 0.99522328341619848

FAT= 0.11672517326563459 T11/T22= 0.444339722

Time (relative to 2Ne) = 5.00000000

T11= 0.46958511488444071 T12= 1.4964478413701185 T22= 1.0566694263506828

TT= 1.1299586979395189 TS= 0.99796099520405868

FAT= 0.11681639601178184 T11/T22= 0.444401145

**Scaled recombination rate= 0.4**

Equilibrium results for panmictic population

T11= 0.30501930501930496 T12= 6.0000000000000000 T22= 1.0772200772200773

TT= 1.9555984555984556 TS= 1.0000000000000000

FAT= 0.48864758144126352 T11/T22= 0.283154130

Time (relative to 2Ne) = 4.99999989E-03

T11= 6.5681621938282198E-003 T12= 1.0048487063379463 T22= 0.99945202261081834

TT= 0.99049458707753157 TS= 0.90016363656911935

FAT= 9.1197823478202933E-002 T11/T22= 6.57176320E-03

Time (relative to 2Ne) = 9.99999978E-03

T11= 1.2877860134889951E-002 T12= 1.0097416687766518 T22= 0.99890264236831872

TT= 0.99099341929948448 TS= 0.90030016414497582

FAT= 9.1517515039220010E-002 T11/T22= 1.28920069E-02

Time (relative to 2Ne) = 1.49999997E-02

T11= 1.8877537330213467E-002 T12= 1.0146298634998638 T22= 0.99835739046173688

TT= 0.99149163707728460 TS= 0.90040940514858447

FAT= 9.1863842843084353E-002 T11/T22= 1.89085975E-02

Time (relative to 2Ne) = 1.99999996E-02

T11= 2.4582839580652660E-002 T12= 1.0195132687698507 T22= 0.99781624224476839

TT= 0.99198937299264212 TS= 0.90049290197835685

FAT= 9.2235333870823499E-002 T11/T22= 2.46366393E-02

Time (relative to 2Ne) = 2.99999993E-02

T11= 3.5168992067134253E-002 T12= 1.0292656315407873 T22= 0.99674615898029162

TT= 0.99298389237204931 TS= 0.90058844228897583

FAT= 9.3048286878408826E-002 T11/T22= 3.52838002E-02

Time (relative to 2Ne) = 3.99999991E-02

T11= 4.4746387007337740E-002 T12= 1.0389986122841179 T22= 0.99569219814084375

TT= 0.99397789457529817 TS= 0.90059761702749308

FAT= 9.3946030447391582E-002 T11/T22= 4.49399799E-02

Time (relative to 2Ne) = 5.00000007E-02

T11= 5.3414258304859463E-002 T12= 1.0487120870320421 T22= 0.99465416746437540

TT= 0.99497219389496028 TS= 0.90053017654842382

FAT= 9.4919252945984134E-002 T11/T22= 5.37013374E-02

Time (relative to 2Ne) = 7.50000030E-02

T11= 7.1670426643324681E-002 T12= 1.0729097856881555 T22= 0.99212754291431959

TT= 0.99746377545090015 TS= 0.90008183128722008

FAT= 9.7629554636867732E-002 T11/T22= 7.22391233E-02

Time (relative to 2Ne) = 0.100000001

T11= 8.5951407166925314E-002 T12= 1.0969837432950174 T22= 0.98969638720620945

TT= 0.99997066150180203 TS= 0.89932188920228107

FAT= 0.10065172526998145 T11/T22= 8.68462399E-02

Time (relative to 2Ne) = 0.150000006

T11= 0.10600603519374022 T12= 1.1447579933086063 T22= 0.98510923944092610

TT= 1.0050549830946367 TS= 0.89719891901620752

FAT= 0.10731359566651033 T11/T22= 0.107608408

Time (relative to 2Ne) = 0.200000003

T11= 0.11861868257528732 T12= 1.1920333118979096 T22= 0.98087102487541622

TT= 1.0102577131164638 TS= 0.89464579064540339

FAT= 0.11443804978674044 T11/T22= 0.120931990

Time (relative to 2Ne) = 0.300000012

T11= 0.13231328454328592 T12= 1.2850981444780887 T22= 0.97336060939407909

TT= 1.0210628924606930 TS= 0.88925587690899977

FAT= 0.12908804788121053 T11/T22= 0.135934502

Time (relative to 2Ne) = 0.400000006

T11= 0.13923279693312712 T12= 1.3762161961623480 T22= 0.96701472625515317

TT= 1.0323931715452281 TS= 0.88423653332295060

FAT= 0.14350796024786272 T11/T22= 0.143982083

Time (relative to 2Ne) = 0.500000000

T11= 0.14370566326046796 T12= 1.4654371585132902 T22= 0.96169948723753129

TT= 1.0441923298273974 TS= 0.87990010483982506

FAT= 0.15733904597319681 T11/T22= 0.149428859

Time (relative to 2Ne) = 1.00000000

T11= 0.15929185847124055 T12= 1.8848428657086771 T22= 0.94684427984976427

TT= 1.1078085010905836 TS= 0.86808903771191193

FAT= 0.21639070574262564 T11/T22= 0.168234482

Time (relative to 2Ne) = 2.00000000

T11= 0.18488077151046656 T12= 2.6069355457021461 T22= 0.94867900991965426

TT= 1.2395272039764109 TS= 0.87229918607873547

FAT= 0.29626458920756693 T11/T22= 0.194882318

Time (relative to 2Ne) = 3.00000000

T11= 0.20589528178091823 T12= 3.2003175836660005 T22= 0.96454529302112002

TT= 1.3593978052247966 TS= 0.88868029189709985

FAT= 0.34626914323276892 T11/T22= 0.213463575

Time (relative to 2Ne) = 4.00000000

T11= 0.22320931647323239 T12= 3.6892986617781132 T22= 0.98214778235533862

TT= 1.4618455559926171 TS= 0.90625393576712798

FAT= 0.38006177735255575 T11/T22= 0.227266535

Time (relative to 2Ne) = 5.00000000

T11= 0.23749136337677768 T12= 4.0926785184864967 T22= 0.99809253689438038

TT= 1.5475120018457855 TS= 0.92203241954262005

FAT= 0.40418399440981945 T11/T22= 0.237945229

**Inversion frequency= 0.5**

**Scaled gene conversion rate= 40**

**Initial state of population**

T11= 0 T12= 1 T22= 1

TT= 0.75 TS= 0.5

FAT= 0.3333 T11/T22= 0

**Scaled recombination rate= 40**

**Equilibrium results for panmictic population**

T11= 1 T12= 1.05 T22= 1

TT= 1.025 TS= 1

FAT= 0.02439 T11/T22= 1

Time (relative to 2Ne) = 2.49999994E-03

T11= 4.9611521938714209E-002 T12= 0.97903210528902196 T22= 0.99711026687333160

TT= 0.75119649984752246 TS= 0.52336089440602285

FAT= 0.30329694758661097 T11/T22= 4.97553013E-02

Time (relative to 2Ne) = 4.99999989E-03

T11= 9.6572137562790483E-002 T12= 0.95976708345804351 T22= 0.99333847995891533

TT= 0.75236119610944818 TS= 0.54495530876085296

FAT= 0.27567329152688425 T11/T22= 9.72197726E-02

Time (relative to 2Ne) = 7.49999983E-03

T11= 0.14012817344260553 T12= 0.94244557507855931 T22= 0.98887850388237519

TT= 0.75347445687052483 TS= 0.56450333866249036

FAT= 0.25079963426086893 T11/T22= 0.141704142

Time (relative to 2Ne) = 9.99999978E-03

T11= 0.18055187689970686 T12= 0.92687778991963188 T22= 0.98385714089612197

TT= 0.75454114940877315 TS= 0.58220450889791442

FAT= 0.22839926045901471 T11/T22= 0.183514327

Time (relative to 2Ne) = 1.25000002E-02

T11= 0.21809213350672840 T12= 0.91289245658478158 T22= 0.97838561914224664

TT= 0.75556566645463452 TS= 0.59823887632448747

FAT= 0.20822384752919854 T11/T22= 0.222910196

Time (relative to 2Ne) = 1.49999997E-02

T11= 0.25297657512510996 T12= 0.90033501556753237 T22= 0.97256128371278527

TT= 0.75655197249323991 TS= 0.61276892941894756

FAT= 0.19005045033515799 T11/T22= 0.260113776

Time (relative to 2Ne) = 1.75000001E-02

T11= 0.28541349143436479 T12= 0.88906598861646868 T22= 0.96646911352796483

TT= 0.75750364554881677 TS= 0.62594130248116486

FAT= 0.17367882496767928 T11/T22= 0.295315683

Time (relative to 2Ne) = 1.99999996E-02

T11= 0.31559356363535618 T12= 0.87895950720670302 T22= 0.96018308151945642

TT= 0.75842391489205463 TS= 0.63788832257740635

FAT= 0.15892905003108182 T11/T22= 0.328680605

Time (relative to 2Ne) = 2.25000009E-02

T11= 0.34369143721345397 T12= 0.86990198459322310 T22= 0.95376737387307897

TT= 0.75931569506824470 TS= 0.64872940554326641

FAT= 0.14563940959371213 T11/T22= 0.360351443

Time (relative to 2Ne) = 2.50000004E-02

T11= 0.36986714902473705 T12= 0.86179091743635650 T22= 0.94727748252327282

TT= 0.76018161660518069 TS= 0.65857231577400488

FAT= 0.13366450675950658 T11/T22= 0.390452802

Time (relative to 2Ne) = 2.74999999E-02

T11= 0.39426742250270796 T12= 0.85453380435658211 T22= 0.94076118368329931

TT= 0.76102405372479287 TS= 0.66751430309300364

FAT= 0.12287358089946121 T11/T22= 0.419094056

Time (relative to 2Ne) = 2.99999993E-02

T11= 0.41702684345903562 T12= 0.84804717000950269 T22= 0.93425941392543599

TT= 0.76184514935086933 TS= 0.67564312869223575

FAT= 0.11314900505973169 T11/T22= 0.446371585

Time (relative to 2Ne) = 3.24999988E-02

T11= 0.43826892775586890 T12= 0.84225568438503284 T22= 0.92780705418083576

TT= 0.76264683767669261 TS= 0.68303799096835238

FAT= 0.10438494303714485 T11/T22= 0.472370774

Time (relative to 2Ne) = 3.50000001E-02

T11= 0.45810709104687752 T12= 0.83709136803946027 T22= 0.92143363099692588

TT= 0.76343086453068099 TS= 0.68977036102190170

FAT= 9.6486148164918739E-002 T11/T22= 0.497167766

Time (relative to 2Ne) = 3.99999991E-02

T11= 0.49398002199795343 T12= 0.82840484490462296 T22= 0.90901862335383699

TT= 0.76495208379025914 TS= 0.70149932267589521

FAT= 8.2949981389634275E-002 T11/T22= 0.543421209

Time (relative to 2Ne) = 4.25000004E-02

T11= 0.51019865488866589 T12= 0.82477732016061178 T22= 0.90301463536450544

TT= 0.76569198264359883 TS= 0.70660664512658566

FAT= 7.7165934679134796E-002 T11/T22= 0.564994872

Time (relative to 2Ne) = 4.50000018E-02

T11= 0.52538248689218547 T12= 0.82156521760681867 T22= 0.89716572347090440

TT= 0.76641966139418172 TS= 0.71127410518154499

FAT= 7.1952167970641967E-002 T11/T22= 0.585602522

Time (relative to 2Ne) = 5.00000007E-02

T11= 0.55293839528680711 T12= 0.81622851399767482 T22= 0.88597434555598653

TT= 0.76784244220953579 TS= 0.71945637042139676

FAT= 6.3015625508930917E-002 T11/T22= 0.624102056

Time (relative to 2Ne) = 5.49999997E-02

T11= 0.57717719667685663 T12= 0.81211461765706416 T22= 0.87550410671281054

TT= 0.76922763467594879 TS= 0.72634065169483364

FAT= 5.5753305065778136E-002 T11/T22= 0.659251273

Time (relative to 2Ne) = 5.99999987E-02

T11= 0.59854918977114802 T12= 0.80899541458105406 T22= 0.86578443923795811

TT= 0.77058111454280354 TS= 0.73216681450455301

FAT= 4.9851079027601441E-002 T11/T22= 0.691337407

Time (relative to 2Ne) = 6.49999976E-02

T11= 0.61743822185223451 T12= 0.80668514015320314 T22= 0.85682218674156108

TT= 0.77190767222505041 TS= 0.73713020429689780

FAT= 4.5053921834855459E-002 T11/T22= 0.720614195

Time (relative to 2Ne) = 7.00000003E-02

T11= 0.63417223852172322 T12= 0.80503251796934172 T22= 0.84860758251346513

TT= 0.77321121424346795 TS= 0.74138991051759418

FAT= 4.1154736428659633E-002 T11/T22= 0.747309208

Time (relative to 2Ne) = 7.50000030E-02

T11= 0.64903206120709434 T12= 0.80391435790120536 T22= 0.84111893208048638

TT= 0.77449492727249791 TS= 0.74507549664379036

FAT= 3.7985310933297600E-002 T11/T22= 0.771629333

Time (relative to 2Ne) = 7.99999982E-02

T11= 0.66225870201151038 T12= 0.80323034252619996 T22= 0.83432625988271703

TT= 0.77576141173665680 TS= 0.74829248094711365

FAT= 3.5408993504910113E-002 T11/T22= 0.793764651

Time (relative to 2Ne) = 8.50000009E-02

T11= 0.67405947004277467 T12= 0.80289878133162185 T22= 0.82819412972639128

TT= 0.77701279060810235 TS= 0.75112679988458297

FAT= 3.3314754964664850E-002 T11/T22= 0.813890636

Time (relative to 2Ne) = 9.00000036E-02

T11= 0.68461307799372673 T12= 0.80285315304920568 T22= 0.82268380794252316

TT= 0.77825079800866526 TS= 0.75364844296812494

FAT= 3.1612373676315086E-002 T11/T22= 0.832170367

Time (relative to 2Ne) = 0.100000001

T11= 0.70257566630899737 T12= 0.80341308406616352 T22= 0.81336660624238433

TT= 0.78069211017092721 TS= 0.75797113627569090

FAT= 2.9103629458048119E-002 T11/T22= 0.863787174

Time (relative to 2Ne) = 0.125000000

T11= 0.73426899701534187 T12= 0.80704897664278041 T22= 0.79817046157412397

TT= 0.78663435296875672 TS= 0.76621972929473292

FAT= 2.5951858823580531E-002 T11/T22= 0.919940054

Time (relative to 2Ne) = 0.150000006

T11= 0.75428798276719267 T12= 0.81207490036992369 T22= 0.79114476162665881

TT= 0.79239563628342469 TS= 0.77271637219692568

FAT= 2.4835149495270659E-002 T11/T22= 0.953413367

Time (relative to 2Ne) = 0.174999997

T11= 0.76787114092980913 T12= 0.81750905794664763 T22= 0.78912921626931909

TT= 0.79800461827310576 TS= 0.77850017859956411

FAT= 2.4441512275642618E-002 T11/T22= 0.973061323

Time (relative to 2Ne) = 0.200000003

T11= 0.77781484264791478 T12= 0.82300193388889120 T22b= 0.79007597292366094

TT= 0.80347367083733956 TS= 0.78394540778578792

FAT= 2.4304795241392618E-002 T11/T22= 0.984481096

Time (relative to 2Ne) = 0.300000012

T11= 0.80339612411134487 T12= 0.84403568995348521 T22= 0.80475305199488112

TT= 0.82405513900329908 TS= 0.80407458805311305

FAT= 2.4246618951193843E-002 T11/T22= 0.998313844

Time (relative to 2Ne) = 0.400000006

T11= 0.82220216002195046 T12= 0.86316912770957788 T22= 0.82235232996874208

TT= 0.84272318635246213 TS= 0.82227724499534627

FAT= 2.4261752480801535E-002 T11/T22= 0.999817371

Time (relative to 2Ne) = 0.500000000

T11= 0.83877961311918359 T12= 0.88052593032160587 T22= 0.83879623228752243

TT= 0.85965692651247949 TS= 0.83878792270335301

FAT= 2.4275967732603987E-002 T11/T22= 0.999980211

Time (relative to 2Ne) = 0.600000024

T11= 0.85376380325673085 T12= 0.89627027922494917 T22b=0.85376564248463349

TT= 0.87501750104781573 TS= 0.85376472287068217

FAT= 2.4288403548139104E-002 T11/T22= 0.999997854

Time (relative to 2Ne) = 0.699999988

T11= 0.86735005788964803 T12= 0.91055195902459340 T22= 0.86735026143528948

TT= 0.88895105934353102 TS= 0.86735015966246876

FAT= 2.4299312604468937E-002 T11/T22= 0.999999762

Time (relative to 2Ne) = 0.800000012

T11= 0.87967348142935020 T12= 0.92350685324028003 T22= 0.87967350395555721

TT= 0.90159017296636690 TS= 0.87967349269245365

FAT= 2.4308916546643466E-002 T11/T22= 1.00000000

Time (relative to 2Ne) = 0.899999976

T11= 0.89085197170727604 T12= 0.93525822187785557 T22= 0.89085197420023032

TT= 0.91305509741580437 TS= 0.89085197295375318

FAT= 2.4317398287236003E-002 T11/T22= 1.00000000

Time (relative to 2Ne) = 1.00000000

T11= 0.90099195853630643 T12= 0.94591787394110649 T22= 0.90099195881219929

TT= 0.92345491630767962 TS= 0.90099195867425286

FAT= 2.4324909897325719E-002 T11/T22= 1.00000000

Time (relative to 2Ne) = 1.50000000

T11= 0.93919442991471080 T12= 0.98607819197354518 T22= 0.93919442991471536

TT= 0.96263631094412916 TS= 0.93919442991471302

FAT= 2.4351752331495669E-002 T11/T22= 1.00000000

Time (relative to 2Ne) = 2.00000000

T11= 0.96265639332029473 T12= 1.0107425626657656 T22= 0.96265639332029473

TT= 0.98669947799303004 TS= 0.96265639332029473

FAT= 2.4367180898523966E-002 T11/T22= 1.00000000

Time (relative to 2Ne) = 3.00000000

T11= 0.98591483134930114 T12= 1.0351929774649367 T22= 0.98591483134930114

TT= 1.0105539044071188 TS= 0.98591483134930114

FAT= 2.4381750394871893E-002 T11/T22= 1.00000000

Time (relative to 2Ne) = 4.00000000

T11= 0.99468739113443883 T12= 1.0444151240824200 T22= 0.99468739113443883

TT= 1.0195512576084294 TS= 0.99468739113443883

FAT= 2.4387068613219043E-002 T11/T22= 1.00000000

Time (relative to 2Ne) = 5.00000000

T11= 0.99799620340669204 T12= 1.0478935103974547 T22= 0.99799620340669204

TT= 1.0229448569020734 TS= 0.99799620340669204

FAT= 2.4389050227924147E-002 T11/T22= 1.00000000

**Scaled recombination rate= 4**

**Equilibrium results for panmictic population**

T11= 1 T12= 1.5 T22= 1

TT= 1.25 TS= 1

FAT= 0.2 T11/T22= 1

Time (relative to 2Ne) = 2.49999994E-03

T11= 7.3148393266577738E-003 T12= 1.0000058524484028 T22= 0.99756173239565249

TT= 0.75122206915477907 TS= 0.50243828586115513

FAT= 0.33117209079538557 T11/T22= 7.33271847E-03

Time (relative to 2Ne) = 4.99999989E-03

T11= 1.4705483837823330E-002 T12= 1.0000240212241656 T22= 0.99509827529125083

TT= 0.75246295039435129 TS= 0.50490187956453703

FAT= 0.32900101021594785 T11/T22= 1.47779211E-02

Time (relative to 2Ne) = 7.49999983E-03

T11= 2.2022703007873012E-002 T12= 1.0000543274159619 T22= 0.99265945255117649

TT= 0.75369770259774338 TS= 0.50734107777952475

FAT= 0.32686397207940254 T11/T22= 2.21855566E-02

Time (relative to 2Ne) = 9.99999978E-03

T11= 2.9267287450317481E-002 T12= 1.0000965895481704 T22= 0.99024507898148262

TT= 0.75492638638203524 TS= 0.50975618321590010

FAT= 0.32476041053632643 T11/T22= 2.95556001E-02

Time (relative to 2Ne) = 1.49999997E-02

T11= 4.3541670882651484E-002 T12= 1.0002162677270774 T22= 0.98548894330910641

TT= 0.75736578741147820 TS= 0.51451530709587900

FAT= 0.32065150598578340 T11/T22= 4.41828109E-02

Time (relative to 2Ne) = 1.99999996E-02

T11= 5.7534785527347032E-002 T12= 1.0003816515025010 T22= 0.98082840568263108

TT= 0.75978162355374512 TS= 0.51918159560498900

FAT= 0.31666997527972807 T11/T22= 5.86593784E-02

Time (relative to 2Ne) = 2.99999993E-02

T11= 8.4701139372310053E-002 T12= 1.0008441028681534 T22= 0.97178835047338397

TT= 0.76454442389550015 TS= 0.52824474492284701

FAT= 0.30907252945311015 T11/T22= 8.71600658E-02

Time (relative to 2Ne) = 3.99999991E-02

T11= 0.11081290367374597 T12= 1.0014734325597010 T22= 0.96311351847354609

TT= 0.76921832181667360 TS= 0.53696321107364597

FAT= 0.30193652979365793 T11/T22= 0.115056947

Time (relative to 2Ne) = 5.00000007E-02

T11= 0.13591460683518541 T12= 1.0022596768833969 T22= 0.95479276097571364

TT= 0.77380668039442324 TS= 0.54535368390544958

FAT= 0.29523264954565531 T11/T22= 0.142349854

Time (relative to 2Ne) = 5.99999987E-02

T11= 0.16004884089004545 T12= 1.0031933915246636 T22= 0.94681517624709610

TT= 0.77831270004661712 TS= 0.55343200856857078

FAT= 0.28893360144910529 T11/T22= 0.169039160

Time (relative to 2Ne) = 7.00000003E-02

T11= 0.18325634735549423 T12= 1.0042656249654214 T22= 0.93917010976480930

TT= 0.78273942676278652 TS= 0.56121322856015177

FAT= 0.28301397710194742 T11/T22= 0.195125833

Time (relative to 2Ne) = 7.99999982E-02

T11= 0.20557609921181830 T12= 1.0054678932583054 T22= 0.93184715393417294

TT= 0.78708975991565044 TS= 0.56871162657299568

FAT= 0.27745010094662848 T11/T22= 0.220611393

Time (relative to 2Ne) = 9.00000036E-02

T11= 0.22704537918518408 T12= 1.0067921560885336 T22= 0.92483614733620034

TT= 0.79136645967461283 TS= 0.57594076326069221

FAT= 0.27221989734376340 T11/T22= 0.245497957

Time (relative to 2Ne) = 0.100000001

T11= 0.24769985450331711 T12= 1.0082307940577298 T22= 0.91812717354734341

TT= 0.79557215404153003 TS= 0.58291351402533031

FAT= 0.26730276937910347 T11/T22= 0.269788176

Time (relative to 2Ne) = 0.109999999

T11= 0.26757364828577734 T12= 1.0097765871273459 T22= 0.91171055957160563

TT= 0.79970934552801876 TS= 0.58964210392869143

FAT= 0.26267948820909126 T11/T22= 0.293485314

Time (relative to 2Ne) = 0.119999997

T11= 0.28669940772294167 T12= 1.0114226941625246 T22= 0.90557687392237540

TT= 0.80378041749259155 TS= 0.59613814082265848

FAT= 0.25833209188857464 T11/T22= 0.316593111

Time (relative to 2Ne) = 0.129999995

T11= 0.30510836919063833 T12= 1.0131626335202448 T22= 0.89971692438873518

TT= 0.80778764015496574 TS= 0.60241264678968676

FAT= 0.25424379274468711 T11/T22= 0.339115947

Time (relative to 2Ne) = 0.140000001

T11= 0.32283042044056143 T12= 1.0149902646284741 T22= 0.89412175551856854

TT= 0.81173317630401964 TS= 0.60847608797956498

FAT= 0.25039889246602443 T11/T22= 0.361058682

Time (relative to 2Ne) = 0.150000006

T11= 0.33989416000005912 T12= 1.0168997705057534 T22= 0.88878264584849831

TT= 0.81561908671501604 TS= 0.61433840292427866

FAT= 0.24678270416820991 T11/T22= 0.382426649

Time (relative to 2Ne) = 0.159999996

T11= 0.35632695390868718 T12= 1.0188856411732352 T22= 0.88369110490855185

TT= 0.81944733529092739 TS= 0.62000902940861957

FAT= 0.24338148077753097 T11/T22= 0.403225690

Time (relative to 2Ne) = 0.170000002

T11= 0.37215498991302232 T12= 1.0209426579136378 T22= 0.87883887002742911

TT= 0.82321979394193179 TS= 0.62549692997022577

FAT= 0.24018234914508507 T11/T22= 0.423462152

Time (relative to 2Ne) = 0.180000007

T11= 0.38740332923558485 T12= 1.0230658783338973 T22= 0.87421790296237589

TT= 0.82693824721643883 TS= 0.63081061609898037

FAT= 0.23717324936613426 T11/T22= 0.443142742

Time (relative to 2Ne) = 0.189999998

T11= 0.40209595602833803 T12= 1.0252506221905076 T22= 0.86982038637588854

TT= 0.83060439669631037 TS= 0.63595817120211329

FAT= 0.23434287883424798 T11/T22= 0.462274700

Time (relative to 2Ne) = 0.200000003

T11= 0.41625582461615329 T12= 1.0274924579386415 T22= 0.86563872017982679

TT= 0.83421986516831570 TS= 0.64094727239799010

FAT= 0.23168064060825277 T11/T22= 0.480865538

Time (relative to 2Ne) = 0.224999994

T11= 0.44946654767128347 T12= 1.0333198386147062 T22= 0.85608094040985117

TT= 0.84304679132763682 TS= 0.65277374404056732

FAT= 0.22569689991634512 T11/T22= 0.525028110

Time (relative to 2Ne) = 0.250000000

T11= 0.47980062892884623 T12= 1.0394187651899907 T22= 0.84771686698408089

TT= 0.85158875657322719 TS= 0.66375874795646350

FAT= 0.22056421854673980 T11/T22= 0.565991580

Time (relative to 2Ne) = 0.275000006

T11= 0.50754348128857996 T12= 1.0457372514787169 T22= 0.84044453113347950

TT= 0.85986562884487328 TS= 0.67399400621102967

FAT= 0.21616356835141781 T11/T22= 0.603898823

Time (relative to 2Ne) = 0.300000012

T11= 0.53295104668747173 T12= 1.0522300436580150 T22= 0.83416936087125204

TT= 0.86789512371868849 TS= 0.68356020377936189

FAT= 0.21239308172340332 T11/T22= 0.638900280

Time (relative to 2Ne) = 0.349999994

T11= 0.57765502471512131 T12= 1.0655862976455972 T22= 0.82426678960845656

TT= 0.88327360240369313 TS= 0.70096090716178894

FAT= 0.20640568759868771 T11/T22= 0.700810730

Time (relative to 2Ne) = 0.400000006

T11= 0.61548062625603861 T12= 1.0792309559937487 T22= 0.81738522408437497

TT= 0.89783194058197768 TS= 0.71643292517020685

FAT= 0.20204116963603169 T11/T22= 0.752987206

Time (relative to 2Ne) = 0.449999988

T11= 0.64769151381139123 T12= 1.0929714628684057 T22= 0.81299371114108576

TT= 0.91165703767232209 TS= 0.73034261247623844

FAT= 0.19888446828537920 T11/T22= 0.796674669

Time (relative to 2Ne) = 0.500000000

T11= 0.67530720707725755 T12= 1.1066643468978119 T22= 0.81064249283066858

TT= 0.92481959842588757 TS= 0.74297484995396301

FAT= 0.19662726523252527 T11/T22= 0.833051801

Time (relative to 2Ne) = 0.600000024

T11= 0.71988962743390617 T12= 1.1335125288946917 T22= 0.81060395373929151

TT= 0.94937965974064520 TS= 0.76524679058659884

FAT= 0.19395072062566454 T11/T22= 0.888090432

Time (relative to 2Ne) = 0.699999988

T11= 0.75410509409336812 T12= 1.1592345688057293 T22= 0.81491029320481090

TT= 0.97187113122740942 TS= 0.78450769364908957

FAT= 0.19278629805753378 T11/T22= 0.925384164

Time (relative to 2Ne) = 0.800000012

T11= 0.78116005916124909 T12= 1.1835673823631789 T22= 0.82191737269224951

TT= 0.99255304914496412 TS= 0.80153871592674930

FAT= 0.19244748014503033 T11/T22= 0.950411916

Time (relative to 2Ne) = 0.899999976

T11= 0.80317571862184756 T12= 1.2064082494550084 T22= 0.83049507010065349

TT= 1.0116218219081294 TS= 0.81683539436125052

FAT= 0.19254866129664061 T11/T22= 0.967104733

Time (relative to 2Ne) = 1.00000000

T11= 0.82156567935667058 T12= 1.2277455334031240 T22= 0.83987765579875973

TT= 1.0292336004904197 TS= 0.83072166757771515

FAT= 0.19287354476001906 T11/T22= 0.978196859

Time (relative to 2Ne) = 1.50000000

T11= 0.88367449202415815 T12= 1.3139057821840123 T22= 0.88615225293735145

TT= 1.0994095773323835 TS= 0.88491337248075475

FAT= 0.19510127005813804 T11/T22= 0.997203887

Time (relative to 2Ne) = 2.00000000

T11= 0.92131689269515360 T12= 1.3729680316253119 T22= 0.92165215411067403

TT= 1.1472262775141129 TS= 0.92148452340291387

FAT= 0.19677177775281740 T11/T22= 0.999636233

Time (relative to 2Ne) = 3.00000000

T11= 0.96342430829046877 T12= 1.4408243005991705 T22= 0.96343044636175212

TT= 1.2021258389626404 TS= 0.96342737732611039

FAT= 0.19856362279219608 T11/T22= 0.999993622

Time (relative to 2Ne) = 4.00000000

T11= 0.98296353437066308 T12= 1.4724345110376140 T22= 0.98296364674839976

TT= 1.2276990507985728 TS= 0.98296359055953142

FAT= 0.19934483135736725 T11/T22= 0.999999881

Time (relative to 2Ne) = 5.00000000

T11= 0.99206402428306961 T12= 1.4871593233670048 T22= 0.99206402634051649

TT= 1.2396116743393990 TS= 0.99206402531179305

FAT= 0.19969773934206170 T11/T22= 1.00000000

**Scaked recombination rate= 0.4**

Equilibrium results for panmictic population

Times are scaled by 2Ne

T11= 1 T12= 6 T22= 1

TT= 3.5 TS= 1

FAT= 0.71428571428571430 T11/T22= 1.00000000

Time (relative to 2Ne) = 4.99999989E-03

T11= 5.9102713051500944E-003 T12= 1.0044530600706141 T22= 0.99507876043654164

TT= 0.75247378797072995 TS= 0.50049451587084581

FAT= 0.33486784008705661 T11/T22= 5.93950087E-03

Time (relative to 2Ne) = 9.99999978E-03

T11= 1.1819703700804651E-002 T12= 1.0089471234833187 T22= 0.99016637362076798

TT= 0.75497008107205255 TS= 0.50099303866078637

FAT= 0.33640676469009267 T11/T22= 1.19370883E-02

Time (relative to 2Ne) = 1.99999996E-02

T11= 2.3458672844973494E-002 T12= 1.0179232711620969 T22= 0.98051559154815349

TT= 0.75995520167933017 TS= 0.50198713219656343

FAT= 0.33945167940520082 T11/T22= 2.39248332E-02

Time (relative to 2Ne) = 2.99999993E-02

T11= 3.4862108448943774E-002 T12= 1.0268834667285063 T22= 0.97109256110899889

TT= 0.76493040075373875 TS= 0.50297733477897133

FAT= 0.34245346467684767 T11/T22= 3.58998813E-02

Time (relative to 2Ne) = 3.99999991E-02

T11= 4.6035104412177841E-002 T12= 1.0358277343339104 T22= 0.96189229467809623

TT= 0.76989571693952374 TS= 0.50396369954513709

FAT= 0.34541303652332944 T11/T22= 4.78588976E-02

Time (relative to 2Ne) = 5.00000007E-02

T11= 5.6982643833890956E-002 T12= 1.0447560981864727 T22= 0.95290991321352625

TT= 0.77485118835509059 TS= 0.50494627852370866

FAT= 0.34833128462299368 T11/T22= 5.97985648E-02

Time (relative to 2Ne) = 5.99999987E-02

T11= 6.7709601424279953E-002 T12= 1.0536685825488510 T22= 0.94414064389389241

TT= 0.77979685260396858 TS= 0.50592512265908618

FAT= 0.35120907327382123 T11/T22= 7.17155859E-02

Time (relative to 2Ne) = 7.00000003E-02

T11= 7.8220745863273433E-002 T12= 1.0625652117359810 T22= 0.93557981780696742

TT= 0.78473274678555072 TS= 0.50690028183512048

FAT= 0.35404724231083406 T11/T22= 8.36067051E-02

Time (relative to 2Ne) = 7.99999982E-02

T11= 8.8520742107957018E-002 T12= 1.0714460101128154 T22= 0.92722286768863249

TT= 0.78965890750555512 TS= 0.50787180489829475

FAT= 0.35684660798343248 T11/T22= 9.54686776E-02

Time (relative to 2Ne) = 9.00000036E-02

T11= 9.8614153649770553E-002 T12= 1.0803110020922304 T22b= 0.91906532571101485

TT= 0.79457537088631158 TS= 0.50883973968039276

FAT= 0.35960796379479287 T11/T22= 0.107298307

Time (relative to 2Ne) = 0.100000001

T11= 0.10850544472259804 T12= 1.0891602121329038 T22b= 0.91110282131875364

TT= 0.79948217257678977 TS= 0.50980413302067584

FAT= 0.36233208130515326 T11/T22= 0.119092427

Time (relative to 2Ne) = 0.125000000

T11= 0.13237782144380705 T12= 1.1112143521519604 T22= 0.89202202104242545

TT= 0.81170713669753836 TS= 0.51219992124311631

FAT= 0.36898433180343670 T11/T22= 0.148401961

Time (relative to 2Ne) = 0.150000006

T11= 0.15507966145998475 T12= 1.1331703922599221 T22= 0.87406943991048935

TT= 0.82387247147257958 TS= 0.51457455068523705

FAT= 0.37541965716430259 T11/T22= 0.177422583

Time (relative to 2Ne) = 0.159999996

T11= 0.16384668653258172 T12= 1.1419254266122492 T22= 0.86719056564980301

TT= 0.82872202635172076 TS= 0.51551862609119237

FAT= 0.37793541175602907 T11/T22= 0.188939661

Time (relative to 2Ne) = 0.170000002

T11= 0.17244023720798007 T12= 1.1506648513522988 T22= 0.86047868659702731

TT= 0.83356215662740130 TS= 0.51645946190250369

FAT= 0.38041877525714152 T11/T22= 0.200400352

Time (relative to 2Ne) = 0.180000007

T11= 0.18086405759800284 T12= 1.1593886912350566 T22= 0.85393013792254258

TT= 0.83839289449766463 TS= 0.51739709776027265

FAT= 0.38287036882596825 T11/T22= 0.211801931

Time (relative to 2Ne) = 0.189999998

T11= 0.18912181038805276 T12= 1.1680969710444433 T22= 0.84754133459589687

TT= 0.84321427176820907 TS= 0.51833157249197481

FAT= 0.38529079755132656 T11/T22= 0.223141700

Time (relative to 2Ne) = 0.200000003

T11= 0.19721707860908033 T12= 1.1767897155916476 T22b= 0.84130876964935752

TT= 0.84802631986043320 TS= 0.51926292412921893

FAT= 0.38768065097946680 T11/T22= 0.234417006

Time (relative to 2Ne) = 0.250000000

T11= 0.23537619672069165 T12= 1.2200212794929985 T22= 0.81237203018187798

TT= 0.87194769647214176 TS= 0.52387411345128476

FAT= 0.39919089691864074 T11/T22= 0.289739400

Time (relative to 2Ne) = 0.300000012

T11= 0.26996778316429049 T12= 1.2628681964507713 T22= 0.78685722058873742

TT= 0.89564034916364266 TS= 0.52841250187651401

FAT= 0.41001708736107012 T11/T22= 0.343096286

Time (relative to 2Ne) = 0.349999994

T11= 0.30135984467516452 T12= 1.3053335894307123 T22= 0.76440424643154536

TT= 0.91910781749203363 TS= 0.53288204555335494

FAT= 0.42021813392097096 T11/T22= 0.394241452

Time (relative to 2Ne) = 0.400000006

T11= 0.32988208186347712 T12= 1.3474205877496646 T22= 0.74469055437993481

TT= 0.94235345293568529 TS= 0.53728631812170602

FAT= 0.42984628915200118 T11/T22= 0.442978740

Time (relative to 2Ne) = 0.449999988

T11= 0.35582987965460733 T12= 1.3891323234086217 T22= 0.72742722154832218

TT= 0.96538043700504317 TS= 0.54162855060146475

FAT= 0.43894807700703875 T11/T22= 0.489162177

Time (relative to 2Ne) = 0.500000000

T11= 0.37946788200746995 T12= 1.4304719278378544 T22= 0.71235545217877794

TT= 0.98819179746548913 TS= 0.54591166709312389

FAT= 0.44756506935872553 T11/T22= 0.532694578

Time (relative to 2Ne) = 0.550000012

T11= 0.40103319422084960 T12= 1.4714425290107025 T22= 0.69924343923991084

TT= 1.0107904228705413 TS= 0.55013831673038016

FAT= 0.45573453776100914 T11/T22= 0.573524415

Time (relative to 2Ne) = 0.600000024

T11= 0.42073825162563405 T12= 1.5120472488870469 T22= 0.68788355293130965

TT= 1.0331790755827595 TS= 0.55431090227847180

FAT= 0.46349000344803193 T11/T22= 0.611641705

Time (relative to 2Ne) = 0.649999976

T11= 0.43877338941895938 T12= 1.5522892011514289 T22= 0.67808982204231993

TT= 1.0553604034410342 TS= 0.55843160573063966

FAT= 0.47086170382188242 T11/T22= 0.647072673

Time (relative to 2Ne) = 0.699999988

T11= 0.45530914477529483 T12= 1.5921714892148628 T22= 0.66969567766077964

TT= 1.0773369502164500 TS= 0.56250241121803723

FAT= 0.47787699001224859 T11/T22= 0.679874718

Time (relative to 2Ne) = 0.750000000

T11= 0.47049831912590445 T12= 1.6316972044522702 T22= 0.66255193190473705

TT= 1.0991111649837955 TS= 0.56652512551532075

FAT= 0.48456066723362490 T11/T22= 0.710130453

Time (relative to 2Ne) = 0.800000012

T11= 0.48447782559233676 T12= 1.6708694246505305 T22= 0.65652496719663045

TT= 1.1206854105225070 TS= 0.57050139639448361

FAT= 0.49093528742513592 T11/T22= 0.737942755

Time (relative to 2Ne) = 0.850000024

T11= 0.49737034395665736 T12= 1.7096912126449322 T22= 0.65149511414931660

TT= 1.1420619708489597 TS= 0.57443272905298692

FAT= 0.49702140188944532 T11/T22= 0.763429105

Time (relative to 2Ne) = 0.899999976

T11= 0.50928580321918138 T12= 1.7481656151238099 T22= 0.64735519841770817

TT= 1.1632430579711273 TS= 0.57832050081844477

FAT= 0.50283778024248549 T11/T22= 0.786717713

Time (relative to 2Ne) = 1.00000000

T11= 0.53056933681491292 T12= 1.8240843634181525 T22= 0.64137128163496604

TT= 1.2050273363215460 TS= 0.58597030922493953

FAT= 0.51372861713356111 T11/T22= 0.827242136

Time (relative to 2Ne) = 1.25000000

T11= 0.57244691572414341 T12= 2.0080117438276197 T22= 0.63637214367621664

TT= 1.3062106367638997 TS= 0.60440952970018003

FAT= 0.53728019609640620 T11/T22= 0.899547398

Time (relative to 2Ne) = 1.50000000

T11= 0.60351046952524623 T12= 2.1838456940744582 T22= 0.64039100240182201

TT= 1.4028982150189961 TS= 0.62195073596353412

FAT= 0.55666724121171363 T11/T22= 0.942409337

Time (relative to 2Ne) = 1.75000000

T11= 0.62803123142323258 T12= 2.3519391088851380 T22= 0.64930880443838013

TT= 1.4953045634079722 TS= 0.63867001793080636

FAT= 0.57288298747968547 T11/T22= 0.967230439

Time (relative to 2Ne) = 2.00000000

T11= 0.64848669360297251 T12= 2.5126308788596776 T22= 0.66076241410110159

TT= 1.5836277163558574 TS= 0.65462455385203699

FAT= 0.58662976967944402 T11/T22= 0.981421888

Time (relative to 2Ne) = 2.50000000

T11= 0.68237216009670154 T12= 2.8130950711955633 T22= 0.68645814535761052

TT= 1.7487551119613596 TS= 0.68441515272715603

FAT= 0.60862721827327027 T11/T22= 0.994047701

Time (relative to 2Ne) = 3.00000000

T11= 0.71093657960949841 T12= 3.0876738538576416 T22= 0.71229660369565195

TT= 1.8996452227551084 TS= 0.71161659165257518

FAT= 0.62539500369416468 T11/T22= 0.998090625

Time (relative to 2Ne) = 3.50000000

T11= 0.73624064993205518 T12= 3.3385959928835738 T22= 0.73669333524460756

TT= 2.0375314927359525 TS= 0.73646699258833137

FAT= 0.63854939410069211 T11/T22= 0.999385536

Time (relative to 2Ne) = 4.00000000

T11= 0.75909853751511691 T12= 3.5678992251670483 T22= 0.75924921425260028

TT= 2.1635365505254534 TS= 0.75917387588385865

FAT= 0.64910513034804995 T11/T22= 0.999801517

Time (relative to 2Ne) = 4.50000000

T11= 0.77989848191275324 T12= 3.7774460477787204 T22= 0.77994863480835053

TT= 2.2786848030696363 TS= 0.77992355836055194

FAT= 0.65773082906863201 T11/T22= 0.999935687

Time (relative to 2Ne) = 5.00000000

T11= 0.79887686185175388 T12= 3.9689385957635070 T22= 0.79889355529082762

TT= 2.3839119021673989 TS= 0.79888520857129075

FAT= 0.66488476027786003 T11/T22= 0.999979079

**Table S4**

**Coalescence times for inversion and standard arrangements in a subdivided population**

**Time course of approach to equilibrium**

**Times scaled by 2x population size**

**Inversion frequency= 0.1**

**Local N= 500**

**Total N= 100000**

**Number of populations = 200**

**Neutral Fst= 0.05**

**Scaled migration rate= 19 Migration rate= 0.0095**

Initial state of subdivided population

Alleles sampled from different populations

T11b= 0 T12b= 1.0523684210526316 T22b= 1.0523684210526316

TTb= 1.0418447368421053 TSb= 0.94713157894736844

FATb= 0.0909 T11/T22= 0

Alleles sampled from the same population

T11w= 0 T12w= 1 T22w= 1

TTw= 0.99 TSw= 0.9

FATw= 0.0909 T11/T22= 0

**Scaled gene recombination rate= 40**

Equilibrium results for subdivided population

Alleles sampled from different populations

T11b= 0.98689902793589634 T12b= 1.1387293107977932 T22b= 1.1000437866713373

TTb= 1.1058757334267451 TSb= 1.0887293107977931

FATb= 1.5504836674389066E-002 T11/T22= 0.897145212

Alleles sampled from the same population

T11w= 0.64707177692351836 T12w= 1.1389924686925299 T22w= 1.0395662293966779

TTw= 1.0535380079451997 TSw= 1.0003167841493619

FATw= 5.0516662326819617E-002 T11/T22= 0.622444034

Time (units of 2NT generations) = 2.49999994E-03

Alleles sampled from different populations

T11b= 9.0470778058974188E-002 T12b= 1.0496957230670723 T22b= 1.0521244381570143

TTb= 1.0420707328398444 TSb= 0.95595907214721032

FATb= 8.2635139802807012E-002 T11/T22= 8.59886706E-02

Alleles sampled from the same population

T11w= 5.8903433078786316E-002 T12w= 1.0501319171837042 T22w= 0.99427794860199548

TTw= 0.99497791779147104 TSw= 0.90074049704967463

FATw= 9.4713077603745255E-002 T11/T22= 5.92424199E-02

Time (units of 2NT generations) = 4.99999989E-03

Alleles sampled from different populations

T11b= 0.17336087601959593 T12b= 1.0477666110871331 T22b= 1.0518308263196747

TTb= 1.0423145680748165 TSb= 0.96398383128966691

FATb= 7.5150764638959755E-002 T11/T22= 0.164818212

Alleles sampled from the same population

T11w= 0.11326810589247005 T12w= 1.0481893820694277 T22w= 0.99400029174213245

TTw= 0.99494700614254894 TSw= 0.90592707315716625

FATw= 8.9472034626765340E-002 T11/T22= 0.113951780

Time (units of 2NT generations) = 7.49999983E-03

Alleles sampled from different populations

T11b= 0.24772926823943830 T12b= 1.0463013012228561 T22b= 1.0515240394185894

TTb= 1.0425459988315660 TSb= 0.97114456230067436

FATb= 6.8487564683874624E-002 T11/T22= 0.235590681

Alleles sampled from the same population

T11w= 0.16204423441218752 T12w= 1.0467106660487946 T22w= 0.99371028322323540

TTw= 0.99493369164372569 TSw= 0.91054367834213068

FATw= 8.4819736240085097E-002 T11/T22= 0.163069904

Time (units of 2NT generations) = 9.99999978E-03

Alleles sampled from different populations

T11b= 0.31447412533363805 T12b= 1.0452373701969842 T22b= 1.0512085426045419

TTb= 1.0427663873984725 TSb= 0.97753510087745155

FATb= 6.2555992702988950E-002 T11/T22= 0.299154848

Alleles sampled from the same population

T11w= 0.20582083564037623 T12w= 1.0456347143175924 T22w= 0.99341206739270616

TTw= 0.99493623152166244 TSw= 0.91465294421747312

FATw= 8.0691892365205686E-002 T11/T22= 0.207185760

Time (units of 2NT generations) = 1.25000002E-02

Alleles sampled from different populations

T11b= 0.37439782880788863 T12b= 1.0445197697530839 T22b= 1.0508881608740943

TTb= 1.0429769471516503 TSb= 0.98323912766747379

FATb= 5.7276260656881628E-002 T11/T22= 0.356268018

Alleles sampled from the same population

T11w= 0.24512408549924869 T12w= 1.0449063320381333 T22w= 0.99310925568562980

TTw= 0.99495287772721674 TSw= 0.91831073866699175

FATw= 7.7030923550168162E-002 T11/T22= 0.246824890

Time (units of 2NT generations) = 1.49999997E-02

Alleles sampled from different populations

T11b= 0.42821724585284993 T12b= 1.0440999917447931 T22b= 1.0505661565586388

TTb= 1.0431787577850888 TSb= 0.98833126548805994

FATb= 5.2577271045551988E-002 T11/T22= 0.407606155

Alleles sampled from the same population

T11w= 0.28042408381189321 T12w= 1.0444768801148983 T22w= 0.99280492819580513

TTw= 0.99498207109740289 TSw= 0.92156684375741393

FATw= 7.3785477620733952E-002 T11/T22= 0.282456368

Time (units of 2NT generations) = 1.75000001E-02

Alleles sampled from different populations

T11b= 0.47657289899592614 T12b= 1.0439353317837168 T22b= 1.0502452977350358

TTb= 1.0433727798764074 TSb= 0.99287805786112493

FATb= 4.8395667386745389E-002 T11/T22= 0.453772932

Alleles sampled from the same population

T11w= 0.31214086723677315 T12w= 1.0443035371961642 T22w= 0.99250169828090617

TTw= 0.99502242097521143 TSw= 0.92446561517649284

FATw= 7.0909764756422855E-002 T11/T22= 0.314499080

Time (units of 2NT generations) = 1.99999996E-02

Alleles sampled from different populations

T11b= 0.52003714958527680 T12b= 1.0439882338945441 T22b= 1.0499279188608608

TTb= 1.0435598678741680 TSb= 0.99693884193330251

FATb= 4.4674989309273605E-002 T11/T22= 0.495307475

Alleles sampled from the same population

T11w= 0.34064977553292020 T12w= 1.0443486428278757 T22w= 0.99220176982510910

TTw= 0.99507268702268525 TSw= 0.92704657039589033

FATw= 6.8362962338292088E-002 T11/T22= 0.343327105

Time (units of 2NT generations) = 2.25000009E-02

Alleles sampled from different populations

T11b= 0.55912150131590010 T12b= 1.0442257073591183 T22b= 1.0496159744932438

TTb= 1.0437407816773279 TSb= 1.0005665271755095

FATb= 4.1364920543236594E-002 T11/T22= 0.532691479

Alleles sampled from the same population

T11w= 0.36628624080226435 T12w= 1.0445791129579991 T22w= 0.99190698796986132

TTw= 0.99513176299605022 TSw= 0.92934491325310153

FATw= 6.6108682477266867E-002 T11/T22= 0.369274795

Time (units of 2NT generations) = 2.50000004E-02

Alleles sampled from different populations

T11b= 0.59428311856136840 T12b= 1.0446188078865632 T22b= 1.0493110868565909

TTb= 1.0439161969590338 TSb= 1.0038082900270688

FATb= 3.8420619441293047E-002 T11/T22= 0.566355526

Alleles sampled from the same population

T11w= 0.38935006184904197 T12w= 1.0449659199110757 T22w= 0.99161888403553999

TTw= 0.99519866227127163 TSw= 0.93139200181689019

FATw= 6.4114495802034099E-002 T11/T22= 0.392640829

Time (units of 2NT generations) = 2.74999999E-02

Alleles sampled from different populations

T11b= 0.62593064406202548 T12b= 1.0451421760984978 T22b= 1.0490145879409567

TTb= 1.0440867143705248 TSb= 1.0067061935530637

FATb= 3.5802122853366369E-002 T11/T22= 0.596684396

Alleles sampled from the same population

T11w= 0.41010921909944570 T12w= 1.0454836298064578 T22w= 0.99133871527786899

TTw= 0.99527250493123087 TSw= 0.93321576566002673

FATw= 6.2351505706964128E-002 T11/T22= 0.413692325

Time (units of 2NT generations) = 2.99999993E-02

Alleles sampled from different populations

T11b= 0.65442939140902323 T12b= 1.0457736270782663 T22b= 1.0487275567383629

TTb= 1.0442528677462521 TSb= 1.0092977402054291

FATb= 3.3473815222805792E-002 T11/T22= 0.624022305

Alleles sampled from the same population

T11w= 0.42880327954991498 T12w= 1.0461099911553280 T22w= 0.99106750005262712

TTw= 0.99535250624608629 TSw= 0.93484107800235594

FATw= 6.0793967829493534E-002 T11/T22= 0.432668090

Time (units of 2NT generations) = 3.24999988E-02

Alleles sampled from different populations

T11b= 0.68010597963426012 T12b= 1.0464937854108252 T22b= 1.0484508521579663

TTb= 1.0444151314182439 TSb= 1.0116163649055956

FATb= 3.1403955693470209E-002 T11/T22= 0.648677051

Alleles sampled from the same population

T11w= 0.44564643588182268 T12w= 1.0468255690507660 T22w= 0.99080604889948309

TTw= 0.99543796639653748 TSw= 0.93629008759771704

FATw= 5.9418950045610930E-002 T11/T22= 0.449781716

Time (units of 2NT generations) = 3.50000001E-02

Alleles sampled from different populations

T11b= 0.70325246996328195 T12b= 1.0472857607444015 T22b= 1.0481851421017929

TTb= 1.0445739267360774 TSb= 1.0136918748879418

FATb= 2.9564256830180602E-002 T11/T22= 0.670923889

Alleles sampled from the same population

T11w= 0.46083021912421834 T12w= 1.0476134199709528 T22w= 0.99055499199989561

TTw= 0.99552826130592920 TSw= 0.93758251471232790

FATw= 5.8206028744566596E-002 T11/T22= 0.465224266

Time (units of 2NT generations) = 3.75000015E-02

Alleles sampled from different populations

T11b= 0.72413005831715671 T12b= 1.0481348594441098 T22b= 1.0479309291300074

TTb= 1.0447296278784175 TSb= 1.0155508420487223

FATb= 2.7929509273083464E-002 T11/T22= 0.691009343

Alleles sampled from the same population

T11w= 0.47452592000302135 T12w= 1.0484588027558692 T22w= 0.99031480341419575

TTw= 0.99562283446158528 TSw= 0.93873591507307841

FATw= 5.7137017572794324E-002 T11/T22= 0.479166746

Time (units of 2NT generations)= 3.99999991E-02

Alleles sampled from different populations

T11b= 0.74297237137581906 T12b= 1.0490283283885478 T22b= 1.0476885730976506

TTb= 1.0448825670327939 TSb= 1.0172169529254675

FATb= 2.6477247281376126E-002 T11/T22= 0.709153831

Alleles sampled from the same population

T11w= 0.48688675032911910 T12w= 1.0493489217997185 T22w= 0.99008582245855636

TTw= 0.99572118961867129 TSw= 0.93976591524561259

FATw= 5.6195725225540061E-002 T11/T22= 0.491762161

Time (units of 2NT generations) = 4.25000004E-02

Alleles sampled from different populations

T11b= 0.75998840886416563 T12b= 1.0499551273891234 T22b= 1.0474583111028573

TTb= 1.0450330390119984 TSb= 1.0187113208789882

FATb= 2.5187450683755830E-002 T11/T22= 0.725554824

Alleles sampled from the same population

T11w= 0.49804977240001075 T12w= 1.0502726989310018 T22w= 0.98986827254295362

TTw= 0.99582288429137300 TSw= 0.94068642252865942

FATw= 5.5367739215943623E-002 T11/T22= 0.503147542

Time (units of 2NT generations) = 4.50000018E-02

Alleles sampled from different populations

T11b= 0.77536517012624073 T12b= 1.0509057270943245 T22b= 1.0472402750492256

TTb= 1.0451813053681136 TSb= 1.0200527645569271

FATb= 2.4042279250618859E-002 T11/T22= 0.740388989

Alleles sampled from the same population

T11w= 0.50813762137589591 T12w= 1.0512205708354649 T22w= 0.98966227775596716

TTw= 0.99592752394647621 TSw= 0.94150981211796003

FATw= 5.4640232868431782E-002 T11/T22= 0.513445497

Time (units of 2NT generations) = 4.74999994E-02

Alleles sampled from different populations

T11b= 0.78926999895241490 T12b= 1.0518719295820933 T22b= 1.0470345070916971

TTb= 1.0453275980585757 TSb= 1.0212580562777689

FATb= 2.3025835944166850E-002 T11/T22= 0.753814697

Alleles sampled from the same population

T11w= 0.51726004290230443 T12w= 1.0521843092188430 T22w= 0.98946787745080267

TTw= 0.99603475682356502 TSw= 0.94224709399595286

FATw= 5.4001793069094695E-002 T11/T22= 0.522765875

Time (units of 2NT generations) = 5.00000007E-02

Alleles sampled from different populations

T11b= 0.80185267696573104 T12b= 1.0528467091476230 T22b= 1.0468409732056425

TTb= 1.0454721227128001 TSb= 1.0223421435816513

FATb= 2.2123955893850966E-002 T11/T22= 0.765973747

Alleles sampled from the same population

T11w= 0.52551526585220232 T12w= 1.0531568612111590 T22w= 0.98928503905890475

TTw= 0.99614426931424349 TSw= 0.94290806173823449

FATw= 5.3442266563112795E-002 T11/T22= 0.531207144

Time (units of 2NT generations) = 5.24999984E-02

Alleles sampled from different populations

T11b= 0.81324729260946271 T12b= 1.0538240710650513 T22b= 1.0466595750924210

TTb= 1.0456150615426649 TSb= 1.0233183468441251

FATb= 2.1324018291821401E-002 T11/T22= 0.776993096

Alleles sampled from the same population

T11w= 0.53299122792008424 T12w= 1.0541322077860842 T22w= 0.98911366933256895

TTw= 0.99625578184007690 TSw= 0.94350142519132052

FATw= 5.2952622820737316E-002 T11/T22= 0.538857400

Time (units of 2NT generations) = 5.49999997E-02

Alleles sampled from different populations

T11b= 0.82357390986548062 T12b= 1.0547989263433333 T22b= 1.0464901606111014

TTb= 1.0457565759354470 TSb= 1.0241985355365395

FATb= 2.0614778711406601E-002 T11/T22= 0.786986768

Alleles sampled from the same population

T11w= 0.54591211270177054 T12w= 1.0560716387612392 T22w= 0.98880471736205167

TTw= 0.99648383716730271 TSw= 0.94451545689602356

FATw= 5.2151754331519640E-002 T11/T22= 0.552092969

Time (units of 2NT generations) = 5.99999987E-02

Alleles sampled from different populations

T11b= 0.84144206319015646 T12b= 1.0567246362665428 T22b= 1.0461864583735252

TTb= 1.0460358864424348 TSb= 1.0257120188551885

FATb= 1.9429417145876093E-002 T11/T22= 0.804294527

Alleles sampled from the same population

T11w= 0.55149072989715953 T12w= 1.0570277941186503 T22w= 0.98866672786262200

TTw= 0.99659995980905258 TSw= 0.94494912806607578

FATw= 5.1827045781612346E-002 T11/T22= 0.557812572

Time (units of 2NT generations) = 6.49999976E-02

Alleles sampled from different populations

T11b= 0.85618992597643406 T12b= 1.0585973285591748 T22b= 1.0459278915051708

TTb= 1.0463110105196043 TSb= 1.0269540949522973

FATb= 1.8500154708010030E-002 T11/T22= 0.818593621

Alleles sampled from the same population

T11w= 0.56116803584393304 T12w= 1.0588978861663725 T22w= 0.98842248209867178

TTw= 0.99683551036831064 TSw= 0.94569703747319789

FATw= 5.1300813788443533E-002 T11/T22= 0.567741096

Time (units of 2NT generations) = 7.00000003E-02

Alleles sampled from different populations

T11b= 0.86840609889184406 T12b= 1.0603990790511684 T22b= 1.0457120986573969

TTb= 1.0465826951306203 TSb= 1.0279814986808415

FATb= 1.7773269648278633E-002 T11/T22= 0.830444753

Alleles sampled from the same population

T11w= 0.56918462084199861 T12w= 1.0606974840403942 T22w= 0.98821865433222922

TTw= 0.99707450334479664 TSw= 0.94631525098320612

FATw= 5.0908184083850339E-002 T11/T22= 0.575970352

Time (units of 2NT generations) = 7.50000030E-02

Alleles sampled from different populations

T11b= 0.87856388075845115 T12b= 1.0621184792786358 T22b= 1.0455364902233419

TTb= 1.0468515221586461 TSb= 1.0288392292768529

FATb= 1.7206158180532727E-002 T11/T22= 0.840299606

Alleles sampled from the same population

T11w= 0.57585090380272164 T12w= 1.0624150941140629 T22w= 0.98805279666848411

TTw= 0.99731599128003068 TSw= 0.94683260738190789

FATw= 5.0619246396850226E-002 T11/T22= 0.582813919

Time (units of 2NT generations) = 7.99999982E-02

Alleles sampled from different populations

T11b= 0.88704431443783394 T12b= 1.0637490204213309 T22b= 1.0453983662301403

TTb= 1.0471179434666316 TSb= 1.0295629610509096

FATb= 1.6765047839409242E-002 T11/T22= 0.848522782

Alleles sampled from the same population

T11w= 0.58141680211189017 T12w= 1.0640441391722182 T22w= 0.98792235720413768

TTw= 0.99755922240746986 TSw= 0.94727180169491298

FATw= 5.0410461437262022E-002 T11/T22= 0.588524818

Time (units of 2NT generations) = 8.50000009E-02

Alleles sampled from different populations

T11b= 0.89415457848012214 T12b= 1.0652878357322400 T22b= 1.0452950030228554

TTb= 1.0473823086651173 TSb= 1.0301809605685821

FATb= 1.6423179916470243E-002 T11/T22= 0.855408847

Alleles sampled from the same population

T11w= 0.58608379228913454 T12w= 1.0655816974803878 T22w= 0.98782476187092083

TTw= 0.99780360058480710 TSw= 0.94765066491274230

FATw= 5.0263334029532869E-002 T11/T22= 0.593307436

Time (units of 2NT generations) = 9.00000036E-02

Alleles sampled from different populations

T11b= 0.90014266939354570 T12b= 1.0667347254272788 T22b= 1.0452237158254498

TTb= 1.0476448870894601 TSb= 1.0307156111822595

FATb= 1.6159364796055176E-002 T11/T22= 0.861196160

Alleles sampled from the same population

T11w= 0.59001453821537964 T12w= 1.0670275249936578 T22w= 0.98775747349918996

TTw= 0.99804865341535609 TSw= 0.94798317997080894

FATw= 5.0163359544869279E-002 T11/T22= 0.597327352

Time (units of 2NT generations) = 9.49999988E-02

Alleles sampled from different populations

T11b= 0.90520912707825385 T12b= 1.0680914038064127 T22b= 1.0451819027464406

TTb= 1.0479058851805538 TSb= 1.0311846251796219

FATb= 1.5956833755209621E-002 T11/T22= 0.866078079

Alleles sampled from the same population

T11w= 0.59334058040397053 T12w= 1.0683833003384744 T22w= 0.98771803335955632

TTw= 0.99829400688620584 TSw= 0.94828028806399778

FATw= 5.0099187691416303E-002 T11/T22= 0.600718558

Time (units of 2NT generations) = 0.100000001

Alleles sampled from different populations

T11b= 0.90951640284813451 T12b= 1.0693609209272172 T22b= 1.0451670745960733

TTb= 1.0481654602181998 TSb= 1.0316020074212795

FATb= 1.5802326469975703E-002 T11/T22= 0.870211482

Alleles sampled from the same population

T11w= 0.59616847938428275 T12w= 1.0696520447784534 T22w= 0.98770408930647280

TTw= 0.99853936519220743 TSw= 0.94855052831425379

FATw= 5.0061959117987609E-002 T11/T22= 0.603590190

Time (units of 2NT generations) = 0.150000006

Alleles sampled from different populations

T11b= 0.93070648605327999 T12b= 1.0782581247651568 T22b= 1.0460556270549819

TTb= 1.0506985852327964 TSb= 1.0345207129548117

FATb= 1.5397253318277015E-002 T11/T22= 0.889729440

Alleles sampled from the same population

T11w= 0.61008624508874321 T12w= 1.0785452079202469 T22w= 0.98854425132880464

TTw= 1.0009598434528637 TSw= 0.95069845070479853

FATw= 5.0213195940694177E-002 T11/T22= 0.617156208

Time (units of 2NT generations) = 0.200000003

Alleles sampled from different populations

T11b= 0.93765203199979130 T12b= 1.0831027966114239 T22b= 1.0478946750258054

TTb= 1.0531297104809567 TSb= 1.0368704107232039

FATb= 1.5439028636204166E-002 T11/T22= 0.894796073

Alleles sampled from the same population

T11w= 0.61465220450263358 T12w= 1.0833881620863208 T22w= 0.99028241076125667

TTw= 1.0032851439371822 TSw= 0.95271939013539442

FATw= 5.0400181949623035E-002 T11/T22= 0.620683730

Time (units of 2NT generations) = 0.250000000

Alleles sampled from different populations

T11b= 0.94120551823829013 T12b= 1.0863322410405130 T22b= 1.0500091539073417

TTb= 1.0554592732346220 TSb= 1.0391287903404365

FATb= 1.5472395106386427E-002 T11/T22= 0.896378398

Alleles sampled from the same population

T11w= 0.61698983075015590 T12w= 1.0866164271600707 T22w= 0.99228076414768929

TTw= 1.0055082741559427 TSw= 0.95475167080793599

FATw= 5.0478553635586398E-002 T11/T22= 0.621789575

Time (units of 2NT generations) = 0.500000000

Alleles sampled from different populations

T11b= 0.95105296840551912 T12b= 1.0972904745945269 T22b= 1.0600514079534005

TTb= 1.0656644555533246 TSb= 1.0491515639986124

FATb= 1.5495394885942981E-002 T11/T22= 0.897176266

Alleles sampled from the same population

T11w= 0.62347211492932830 T12w= 1.0975703190785335 T22w= 1.0017712676274020

TTw= 1.0152321053616249 TSw= 0.96394135235759471

FATw= 5.0521208631163717E-002 T11/T22= 0.622369707

Time (units of 2NT generations) = 0.750000000

Alleles sampled from different populations

T11b= 0.95831303085108877 T12b= 1.1056815884754432 T22b= 1.0681474613468760

TTb= 1.0738052599250603 TSb= 1.0571640182972972

FATb= 1.5497448419021898E-002 T11/T22= 0.897172987

Alleles sampled from the same population

T11w= 0.62825185627997349 T12w= 1.1059580543209853 T22w= 1.0094224762618225

TTw= 1.0229871741126535 TSw= 0.97130541426363759

FATw= 5.0520437750204539E-002 T11/T22= 0.622387409

Time (units of 2NT generations) = 1.00000000

Alleles sampled from different populations

T11b= 0.96410030939513913 T12b= 1.1123721415570265 T22b= 1.0746049016869017

TTb= 1.0802979589406068 TSb= 1.0635544424577255

FATb= 1.5498980021494080E-002 T11/T22= 0.897167265

Alleles sampled from the same population

T11w= 0.63206197779174278 T12w= 1.1126459131926336 T22w= 1.0155251067539692

TTw= 1.0291722206233067 TSw= 0.97717879385774653

FATw= 5.0519656208822816E-002 T11/T22= 0.622399151

Time (units of 2NT generations) = 2.00000000

Alleles sampled from different populations

T11b= 0.97767458900944271 T12b= 1.1280651106146165 T22b= 1.0897511273883405

TTb= 1.0955268789852812 TSb= 1.0785434735504507

FATb= 1.5502499993940155E-002 T11/T22= 0.897154033

Alleles sampled from the same population

T11w= 0.64099876123437161 T12w= 1.1283325628649572 T22w= 1.0298391095054762

TTw= 1.0436795276274720 TSw= 0.99095507467836574

FATw= 5.0517856826186147E-002 T11/T22= 0.622426093

Time (units of 2NT generations) = 3.00000000

Alleles sampled from different populations

T11b= 0.98316678861207518 T12b= 1.1344145391788265 T22b= 1.0958793417626578

TTb= 1.1016885517660624 TSb= 1.0846080864475995

FATb= 1.5503896533264339E-002 T11/T22= 0.897148728

Alleles sampled from the same population

T11w= 0.64461461414647225 T12w= 1.1346794345844280 T22w= 1.0356306036469798

TTw= 1.0495492333207155 TSw= 0.99652900469692907

FATw= 5.0517142922427150E-002 T11/T22= 0.622436821

Time (units of 2NT generations) = 4.00000000

Alleles sampled from different populations

T11b= 0.98538895122756309 T12b= 1.1369835395440602 T22b= 1.0983588381152261

TTb= 1.1041815855035397 TSb= 1.0870618494264599

FATb= 1.5504457148932338E-002 T11/T22= 0.897146642

Alleles sampled from the same population

T11w= 0.64607760051727392 T12w= 1.1372474004415949 T22w= 1.0379738618495584

TTw= 1.0519241361828022 TSw= 0.99878423571633002

FATw= 5.0516856338428617E-002 T11/T22= 0.622441113

Time (units of 2NT generations) = 5.00000000

Alleles sampled from different populations

T11b= 0.98628804577017737 T12b= 1.1380229658183827 T22b= 1.0993620507619912

TTb= 1.1051902754222236 TSb= 1.0880546502628099

FATb= 1.5504683257249297E-002 T11/T22= 0.897145808

Alleles sampled from the same population

T11w= 0.64666952977246839 T12w= 1.1382864081504396 T22w= 1.0389219520780550

TTw= 1.0528850299480286 TSw= 0.99969670984749648

FATw= 5.0516740752936218E-002 T11/T22= 0.622442842

**Scaled gene conversion rate= 4**

Equilibrium results for subdivided population

Alleles sampled from different populations

T11b= 0.65832851359167155 T12b= 1.5786581839213418 T22b= 1.1253614806246386

TTb= 1.2022845575477157 TSb= 1.0786581839213418

FATb= 0.10282621767889377 T11/T22= 0.584992945

Alleles sampled from the same population

T11w= 0.43180143993937109 T12w= 1.5789213418160786 T22w= 1.0634851557282499

TTw= 1.1499468320661703 TSw= 1.0003167841493621

FATw= 0.13011910093961476 T11/T22= 0.406024873

Time (units of 2NT generations) = 2.49999994E-03

Alleles sampled from different populations

T11b= 1.1620979840086809E-002 T12b= 1.0540391898923172 T22b= 1.0521388899114541

TTb= 1.0420757648072958 TSb= 0.94808709890431742

FATb= 9.0193697116024096E-002 T11/T22= 1.10451002E-02

Alleles sampled from the same population

T11w= 7.5042152747256674E-003 T12w= 1.0543142873459301 T22w= 0.99428717262944577

TTw= 0.99522422370486585 TSw= 0.89560887689397384

FATw= 0.10009337035633992 T11/T22= 7.54733197E-03

Time (units of 2NT generations) = 4.99999989E-03

Alleles sampled from different populations

T11b= 2.3201186595337431E-002 T12b= 1.0560075183332016 T22b= 1.0518810434850940

TTb= 1.0423370103888558 TSb= 0.94901305779611844

FATb= 8.9533377077267717E-002 T11/T22= 2.20568534E-02

Alleles sampled from the same population

T11w= 1.5100273978622680E-002 T12w= 1.0562840322698954 T22w= 0.99404343381983085

TTw= 0.99545730994243042 TSw= 0.89614911783570994

FATw= 9.9761377122705275E-002 T11/T22= 1.51907587E-02

Time (units of 2NT generations) = 7.49999983E-03

Alleles sampled from different populations

T11b= 3.4508084892729274E-002 T12b= 1.0579705854448260 T22b= 1.0516260926588619

TTb= 1.0425969212826742 TSb= 0.94991429188224863

FATb= 8.8895936203610804E-002 T11/T22= 3.28140259E-02

Alleles sampled from the same population

T11w= 2.2517061416104101E-002 T12w= 1.0582469019180243 T22w= 0.99380251643402095

TTw= 0.99568965127096254 TSw= 0.89667397093222934

FATw= 9.9444320037215594E-002 T11/T22= 2.26574801E-02

Time (units of 2NT generations) = 9.99999978E-03

Alleles sampled from different populations

T11b= 4.5548490717030043E-002 T12b= 1.0599282879645253 T22b= 1.0513740220454082

TTb= 1.0428555345975656 TSb= 0.95079146891257038

FATb= 8.8280747074447330E-002 T11/T22= 4.33228239E-02

Alleles sampled from the same population

T11w= 2.9759051182332574E-002 T12w= 1.0602044116713807 T22w= 0.99356432081627455

TTw= 0.99592148447385431 TSw= 0.89718379385288038

FATw= 9.9142042982572121E-002 T11/T22= 2.99518108E-02

Time (units of 2NT generations) = 1.25000002E-02

Alleles sampled from different populations

T11b= 5.6329048066894316E-002 T12b= 1.0618805344785984 T22b= 1.0511248158189339

TTb= 1.0431128875001532 TSb= 0.95164523904373000

FATb= 8.7687200064824999E-002 T11/T22= 5.35893030E-02

Alleles sampled from the same population

T11w= 3.6830601159208998E-002 T12w= 1.0621564700369781 T22w= 0.99332883200969535

TTw= 0.99615282454610155 TSw= 0.89767900892464669

FATw= 9.8854124783839814E-002 T11/T22= 3.70779559E-02

Time (units of 2NT generations) = 1.49999997E-02

Alleles sampled from different populations

T11b= 6.6856233299915635E-002 T12b= 1.0638272372254585 T22b= 1.0508784581214323

TTb= 1.0433690161119420 TSb= 0.95247623563928063

FATb= 8.7114701576407105E-002 T11/T22= 6.36193752E-02

Alleles sampled from the same population

T11w= 4.3735959270986835E-002 T12w= 1.0641029891366873 T22w= 0.99309603502800603

TTw= 0.99638368600999849 TSw= 0.89816002745230406

FATw= 9.8580155352632648E-002 T11/T22= 4.40400094E-02

Time (units of 2NT generations) = 1.75000001E-02

Alleles sampled from different populations

T11b= 7.7136359374530006E-002 T12b= 1.0657683119946213 T22b= 1.0506349330664089

TTb= 1.0436239555365685 TSb= 0.95328507569722098

FATb= 8.6562673614463681E-002 T11/T22= 7.34188035E-02

Alleles sampled from the same population

T11w= 5.0479266266642564E-002 T12w= 1.0660438846464351 T22w= 0.99286591485802633

TTw= 0.99661408293402609 TSw= 0.89862724999888788

FATw= 9.8319735405168585E-002 T11/T22= 5.08419760E-02

Time (units of 2NT generations) = 1.99999996E-02

Alleles sampled from different populations

T11b= 8.7175579984404283E-002 T12b= 1.0677036780285485 T22b= 1.0503942247424818

TTb= 1.0438777398863932 TSb= 0.95407236026667408

FATb= 8.6030553376387542E-002 T11/T22= 8.29932019E-02

Alleles sampled from the same population

T11w= 5.7064558431666468E-002 T12w= 1.0679790756979703 T22w= 0.99263845646307858

TTw= 0.99684402894504498 TSw= 0.89908106665993737

FATw= 9.8072476181223278E-002 T11/T22= 5.74877560E-02

Time (units of 2NT generations)= 1.25000002E-02

Alleles sampled from different populations

T11b= 5.6329048066894316E-002 T12b= 1.0618805344785984 T22b= 1.0511248158189339

TTb= 1.0431128875001532 TSb= 0.95164523904373000

FATb= 8.7687200064824999E-002 T11/T22= 5.35893030E-02

Alleles sampled from the same population

T11w= 3.6830601159208998E-002 T12w= 1.0621564700369781 T22w= 0.99332883200969535

TTw= 0.99615282454610155 TSw= 0.89767900892464669

FATw= 9.8854124783839814E-002 T11/T22= 3.70779559E-02

Time (units of 2NT generations) = 1.49999997E-02

Alleles sampled from different populations

T11b= 6.6856233299915635E-002 T12b= 1.0638272372254585 T22b= 1.0508784581214323

TTb= 1.0433690161119420 TSb= 0.95247623563928063

FATb= 8.7114701576407105E-002 T11/T22= 6.36193752E-02

Alleles sampled from the same population

T11w= 4.3735959270986835E-002 T12w= 1.0641029891366873 T22w= 0.99309603502800603

TTw= 0.99638368600999849 TSw= 0.89816002745230406

FATw= 9.8580155352632648E-002 T11/T22= 4.40400094E-02

Time (units of 2NT generations) = 1.75000001E-02

Alleles sampled from different populations

T11b= 7.7136359374530006E-002 T12b= 1.0657683119946213 T22b= 1.0506349330664089

TTb= 1.0436239555365685 TSb= 0.95328507569722098

FATb= 8.6562673614463681E-002 T11/T22= 7.34188035E-02

Alleles sampled from the same population

T11w= 5.0479266266642564E-002 T12w= 1.0660438846464351 T22w= 0.99286591485802633

TTw= 0.99661408293402609 TSw= 0.89862724999888788

FATw= 9.8319735405168585E-002 T11/T22= 5.08419760E-02

Time (units of 2NT generations) = 1.99999996E-02

Alleles sampled from different populations

T11b= 8.7175579984404283E-002 T12b= 1.0677036780285485 T22b= 1.0503942247424818

TTb= 1.0438777398863932 TSb= 0.95407236026667408

FATb= 8.6030553376387542E-002 T11/T22= 8.29932019E-02

Alleles sampled from the same population

T11w= 5.7064558431666468E-002 T12w= 1.0679790756979703 T22w= 0.99263845646307858

TTw= 0.99684402894504498 TSw= 0.89908106665993737

FATw= 9.8072476181223278E-002 T11/T22= 5.74877560E-02

Time (units of 2NT generations) = 2.50000004E-02

Alleles sampled from different populations

T11b= 0.10655514733736160 T12b= 1.0715569775539389 T22b= 1.0499211945387903

TTb= 1.0443819750095027 TSb= 0.95558458981864747

FATb= 8.5023858430769317E-002 T11/T22= 0.101488709

Alleles sampled from the same population

T11w= 6.9776736886457136E-002 T12w= 1.0718320376607440 T22w= 0.99219146475375330

TTw= 0.99730262059833874 TSw= 0.89994999196702374

FATw= 9.7615935845939750E-002 T11/T22= 7.03258812E-02

Time (units of 2NT generations) = 2.99999993E-02

Alleles sampled from different populations

T11b= 0.12504113021347130 T12b= 1.0753865552272543 T22b= 1.0494592398515576

TTb= 1.0448819755228023 TSb= 0.95701742888774899

FATb= 8.4090403216201204E-002 T11/T22= 0.119148150

Alleles sampled from the same population

T11w= 8.1902794430157022E-002 T12w= 1.0756612936197443 T22w= 0.99175493925944380

TTw= 0.99775956159600510 TSw= 0.90076972477651518

FATw= 9.7207624514613622E-002 T11/T22= 8.25837031E-02

Time (units of 2NT generation s)= 3.50000001E-02

Alleles sampled from different populations

T11b= 0.14267742274858830 T12b= 1.0791918798521030 T22b= 1.0490082328363208

TTb= 1.0453779811982844 TSb= 0.95837515182754762

FATb= 8.3226192760448403E-002 T11/T22= 0.136011735

Alleles sampled from the same population

T11w= 9.3471521791083645E-002 T12w= 1.0794663115892464 T22w= 0.99132875916438690

TTw= 0.99821494622712870 TSw= 0.90154303542705660

FATw= 9.6844783947039659E-002 T11/T22= 9.42891240E-02

Time (units of 2NT generations) = 3.99999991E-02

Alleles sampled from different populations

T11b= 0.15950573330363205 T12b= 1.0829724668891898 T22b= 1.0485680455600177

TTb= 1.0458702182767050 TSb= 0.95966181433437914

FATb= 8.2427439309222028E-002 T11/T22= 0.152117670

Alleles sampled from the same population

T11w= 0.10451027602396135 T12w= 1.0832466062800936 T22w= 0.99091280356935196

TTw= 0.99866886278183165 TSw= 0.90227255081481295

FATw= 9.6524799720402732E-002 T11/T22= 0.105468690

Time (units of 2NT generations) = 5.00000007E-02

Alleles sampled from different populations

T11b= 0.19089496276331441 T12b= 1.0904577079707887 T22b= 1.0477196185896986

TTb= 1.0468442281200310 TSb= 0.96203715300706016

FATb= 8.1012124664689655E-002 T11/T22= 0.182200432

Alleles sampled from the same population

T11w= 0.12510055111516871 T12w= 1.0907313027827117 T22w= 0.99011108237599210

TTw= 0.99957261673659348 TSw= 0.90361002924990985

FATw= 9.6003617826168952E-002 T11/T22= 0.126350015

Time (units of 2NT generations) = 5.99999987E-02

Alleles sampled from different populations

T11b= 0.21950278513828025 T12b= 1.0978392404501756 T22b= 1.0469129370253161

TTb= 1.0478055701229205 TSb= 0.96417192183661249

FATb= 7.9817907702568136E-002 T11/T22= 0.209666699

Alleles sampled from the same population

T11w= 0.14386643625311613 T12w= 1.0981123398237154 T22w= 0.98934880995417074

TTw= 1.0004714215936783 TSw= 0.90480057258406532

FATw= 9.5625768957214419E-002 T11/T22= 0.145415276

Time (units of 2NT generations) = 7.00000003E-02

Alleles sampled from different populations

T11b= 0.24559463884190724 T12b= 1.1051146207743714 T22b= 1.0461469827982290

TTb= 1.0487556341943716 TSb= 0.96609174840259693

FATb= 7.8820921763414376E-002 T11/T22= 0.234761119

Alleles sampled from the same population

T11w= 0.16098203607125994 T12w= 1.1053872690831996 T22w= 0.98862502421706222

TTw= 1.0013657984115090 TSw= 0.90586072540248197

FATw= 9.5374810244696850E-002 T11/T22= 0.162834272

Time (units of 2NT generations) = 7.99999982E-02

Alleles sampled from different populations

T11b= 0.26941021282061883 T12b= 1.1122819345696935 T22b= 1.0454207438608383

TTb= 1.0496956528780301 TSb= 0.96781969075681640

FATb= 7.7999715342945586E-002 T11/T22= 0.257705063

Alleles sampled from the same population

T11w= 0.17660456584282613 T12w= 1.1125541718853955 T22w= 0.98793876877064901

TTw= 1.0022561993020251 TSw= 0.90680534847786676

FATw= 9.5235979473742072E-002 T11/T22= 0.178760633

Time (units of 2NT generations) = 9.00000036E-02

Alleles sampled from different populations

T11b= 0.29116595432493453 T12b= 1.1193397379022878 T22b= 1.0447332160835483

TTb= 1.0506267173933352 TSb= 0.96937648990768699

FATb= 7.7335009799898047E-002 T11/T22= 0.278698862

Alleles sampled from the same population

T11w= 0.19087599635404545 T12w= 1.1196116004138625 T22w= 0.98728909470602455

TTw= 1.0031430147499156 TSw= 0.90764778487082665

FATw= 9.5196027360960112E-002 T11/T22= 0.193333432

Time (units of 2NT generations) = 0.100000001

Alleles sampled from different populations

T11b= 0.31105733220211762 T12b= 1.1262870044454369 T22b= 1.0440834049029857

TTb= 1.0515497920936183 TSb= 0.97078079763289882

FATb= 7.6809481650802036E-002 T11/T22= 0.297923833

Alleles sampled from the same population

T11w= 0.20392453842150021 T12w= 1.1265585248378938 T22w= 0.98667506215666134

TTw= 1.0040265802019317 TSw= 0.90840000978314528

FATw= 9.5243066572554103E-002 T11/T22= 0.206678510

Time (units of 2NT generations) = 0.125000000

Alleles sampled from different populations

T11b= 0.35374603445557318 T12b= 1.1431680620428089 T22b= 1.0426174626994884

TTb= 1.0538278562988470 TSb= 0.97373031987509695

FATb= 7.6006281239386575E-002 T11/T22= 0.339286506

Alleles sampled from the same population

T11w= 0.23192831403130748 T12w= 1.1434388501329549 T22w= 0.98528984138025655

TTw= 1.0062230476822527 TSw= 0.90995368864536164

FATw= 9.5673975326483696E-002 T11/T22= 0.235390946

Time (units of 2NT generations) = 0.150000006

Alleles sampled from different populations

T11b= 0.38816301880345405 T12b= 1.1593529973666525 T22b= 1.0413661214366221

TTb= 1.0560717280776959 TSb= 0.97604581117330524

FATb= 7.5776971181736874E-002 T11/T22= 0.372744054

Alleles sampled from the same population

T11w= 0.25450639381602164 T12w= 1.1596231969378503 T22w= 0.98410741431744697

TTw= 1.0084042449841055 TSw= 0.91114731226730450

FATw= 9.6446373763860893E-002 T11/T22= 0.258616477

Time (units of 2NT generations)= 0.174999997

Alleles sampled from different populations

T11b= 0.41611064897149830 T12b= 1.1748491855035095 T22b= 1.0403149306839665

TTb= 1.0582890537343597 TSb= 0.97789450251271970

FATb= 7.5966533848151996E-002 T11/T22= 0.399985284

Alleles sampled from the same population

T11w= 0.27284097754614128 T12w= 1.1751189081671960 T22w= 0.98311412537489351

TTw= 1.0105722547992206 TSw= 0.91208681059201835

FATw= 9.7455123806826971E-002 T11/T22= 0.277527273

Time (units of 2NT generations) = 0.200000003

Alleles sampled from different populations

T11b= 0.43898743007837221 T12b= 1.1896707655138969 T22b= 1.0394500622869491

TTb= 1.0604851625457141 TSb= 0.97940379906609154

FATb= 7.6456857995999994E-002 T11/T22= 0.422326624

Alleles sampled from the same population

T11w= 0.28784936933681754 T12w= 1.1899400979864758 T22w= 0.98229690704645378

TTw= 1.0127282060385614 TSw= 0.91285215327549019

FATw= 9.8620787065614945E-002 T11/T22= 0.293037027

Time (units of 2NT generations) = 0.250000000

Alleles sampled from different populations

T11b= 0.47362941257297730 T12b= 1.2173687565726818 T22b= 1.0382272754220103

TTb= 1.0648267634006410 TSb= 0.98176748913710710

FATb= 7.8002617062586710E-002 T11/T22= 0.456190497

Alleles sampled from the same population

T11w= 0.31057748174470962 T12w= 1.2176374961086953 T22w= 0.98114152655370068

TTw= 1.0170051606255099 TSw= 0.91408512207280157

FATw= 0.10119913107363909 T11/T22= 0.316547066

Time (units of 2NT generations) = 0.300000012

Alleles sampled from different populations

T11b= 0.49818425443773601 T12b= 1.2426294260357365 T22b= 1.0376004367284797

TTb= 1.0691114929808785 TSb= 0.98365881849940529

FATb= 7.9928683811279155E-002 T11/T22= 0.480131119

Alleles sampled from the same population

T11w= 0.32668876353533605 T12w= 1.2428977392584493 T22w= 0.98054930271495944

TTw= 1.0212334159009915 TSw= 0.91516324879699718

FATw= 0.10386476338557038 T11/T22= 0.333169132

Time (units of 2NT generations) = 0.349999994

Alleles sampled from different populations

T11b= 0.51634469383483950 T12b= 1.2656553447589578 T22b= 1.0374827871846182

TTb= 1.0733424666145015 TSb= 0.98536897784964028

FATb= 8.1962180293065345E-002 T11/T22= 0.497689903

Alleles sampled from the same population

T11w= 0.33860538185393557 T12w= 1.2659233338644418 T22w= 0.98043824951237990

TTw= 1.0254072360191666 TSw= 0.91625496274653551

FATw= 0.10644773065614577 T11/T22= 0.345361263

Time (units of 2NT generations) = 0.400000006

Alleles sampled from different populations

T11b= 0.53036006990414419 T12b= 1.2866503570330543 T22b= 1.0377975799415187

TTb= 1.0775167047176215 TSb= 0.98705382893778126

FATb= 8.3954963652788428E-002 T11/T22= 0.511043847

Alleles sampled from the same population

T11w= 0.34780275640993019 T12w= 1.2869180855953346 T22w= 0.98073584242267897

TTw= 1.0295193153336295 TSw= 0.91744253382140417

FATw= 0.10886321397078924 T11/T22= 0.354634494

Time (units of 2NT generations) = 0.449999988

Alleles sampled from different populations

T11b= 0.54161113484223533 T12b= 1.3058095757741963 T22b= 1.0384772962738673

TTb= 1.0816284449696103 TSb= 0.98879068013070404

FATb= 8.5831475004815250E-002 T11/T22= 0.521543562

Alleles sampled from the same population

T11w= 0.35518658546379989 T12w= 1.3060770841905369 T22w= 0.98137827743638895

TTw= 1.0335621457324098 TSw= 0.91875910823913010

FATw= 0.11107511818936366 T11/T22= 0.361926287

Time (units of 2NT generations) = 0.500000000

Alleles sampled from different populations

T11b= 0.55095470401794799 T12b= 1.3233145158376374 T22b= 1.0394627779416987

TTb= 1.0856710100237303 TSb= 0.99061197054932371

FATb= 8.7557868448867260E-002 T11/T22= 0.530037940

Alleles sampled from the same population

T11w= 0.36131890612281881 T12w= 1.3235818305725298 T22w= 0.98230965111410640

TTw= 1.0375287359667098 TSw= 0.92021057661497763

FATw= 0.11307461209006597 T11/T22= 0.367825866

Time (units of 2NT generations) = 0.750000000

Alleles sampled from different populations

T11b= 0.58256972088072767 T12b= 1.3912969587428829 T22b= 1.0473859104492429

TTb= 1.1046417372464130 TSb= 1.0009042914923914

FATb= 9.3910489035669009E-002 T11/T22= 0.556213081

Time (units of 2NT generations) = 1.00000000

Alleles sampled from different populations

T11b= 0.60189249423715685 T12b= 1.4367940467145277 T22b= 1.0575228752203918

TTb= 1.1212353822795040 TSb= 1.0119598371220684

FATb= 9.7459950769012704E-002 T11/T22= 0.569153190

Alleles sampled from the same population

T11w= 0.39475396469454521 T12w= 1.4370599773150619 T22w= 0.99937702744046231

TTw= 1.0721137277904311 TSw= 0.93891472116587060

FATw= 0.12423962418527790 T11/T22= 0.395000041

Time (units of 2NT generations) = 1.50000000

Alleles sampled from different populations

T11b= 0.62442098276893632 T12b= 1.4918743671977910 T22b= 1.0771167233215408

TTb= 1.1472461418137399 TSb= 1.0318471492662804

FATb= 0.10058782360777374 T11/T22= 0.579715252

Alleles sampled from the same population

T11w= 0.40954248149085998 T12w= 1.4921393849640723 T22w= 1.0178934773608050

TTw= 1.0971742307706938 TSw= 0.95705837777381053

FATw= 0.12770610999354304 T11/T22= 0.402343154

Time (units of 2NT generations) = 2.00000000

Alleles sampled from different populations

T11b= 0.63672529980558479 T12b= 1.5228931523552991 T22b= 1.0923081172320868

TTb= 1.1652575953800000 TSb= 1.0467498354894365

FATb= 0.10170091176442164 T11/T22= 0.582917273

Alleles sampled from the same population

T11w= 0.41761968036106217 T12w= 1.5231575552173371 T22w= 1.0322494852262798

TTw= 1.1144666397760181 TSw= 0.97078650473975803

FATw= 0.12892277786362139 T11/T22= 0.404572427

Time (units of 2NT generations) = 3.00000000

Alleles sampled from different populations

T11b= 0.64895780512021262 T12b= 1.5543384222054311 T22b= 1.1103835399313426

TTb= 1.1856811613925673 TSb= 1.0642409664502297

FATb= 0.10242230280500342 T11/T22= 0.584444702

Alleles sampled from the same population

T11w= 0.42564986744045480 T12w= 1.5546021367215765 T22w= 1.0493308972420983

TTw= 1.1340429100503879 TSw= 0.98696279426193401

FATw= 0.12969537085851435 T11/T22= 0.405639321

Time (units of 2NT generations) = 4.00000000

Alleles sampled from different populations

T11b= 0.65416437923053483 T12b= 1.5678405690398736 T22b= 1.1186544478542344

TTb= 1.1948630489814125 TSb= 1.0722054409918644

FATb= 0.10265411428875493 T11/T22= 0.584777892

Alleles sampled from the same population

T11w= 0.42906781576069375 T12w= 1.5681039756107116 T22w= 1.0571469633474504

TTw= 1.1428384340789699 TSw= 0.99433904858877475

FATw= 0.12993908943032084 T11/T22= 0.405873388

Time (units of 2NT generations) = 5.00000000

Alleles sampled from different populations

T11b= 0.65647012046141662 T12b= 1.5738296232334745 T22b= 1.1223642387448927

TTb= 1.1989690667700028 TSb= 1.0757748269165450

FATb= 0.10275014032291963 T11/T22= 0.584899366

Alleles sampled from the same population

T11w= 0.43058146270627307 T12w= 1.5740928922121580 T22w= 1.0606527414415412

TTw= 1.1467712557928997 TSw= 0.99764561356801440

FATw= 0.13003957107538155 T11/T22= 0.405958951

**Scaled gene conversion rate= 0.4**

Equilibrium results for subdivided population

Alleles sampled from different populations

T11b= 0.46097604595137653 T12b= 6.0726090661392638 T22b= 1.1405682906045846

TTb= 2.0215397077542949 TSb= 1.0726090661392638

FATb= 0.46940984536444608 T11/T22= 0.404163480

Alleles sampled from the same population

T11w= 0.30250154734745360 T12w= 6.0728722240340005 T22w= 1.0778518104606849

TTw= 1.9692019822727496 TSw= 1.0003167841493619

FATw= 0.49201920719435355 T11/T22= 0.280652255

Time (units of 2NT generations) = 2.49999994E-03

Alleles sampled from different populations

T11b= 3.3669678394421254E-003 T12b= 1.0545033399372157 T22b= 1.0521382639147969

TTb= 1.0420762646380788 TSb= 0.94726113430726144

FATb= 9.0986747849733818E-002 T11/T22= 3.20011913E-03

Alleles sampled from the same population

T11w= 2.2417407886499152E-003 T12w= 1.0547659235233589 T22w= 0.99428295363745867

TTw= 0.99524947608843262 TSw= 0.89507883235257779

FATw= 0.10064877816318907 T11/T22= 2.25463067E-03

Time (units of 2NT generations) = 4.99999989E-03

Alleles sampled from different populations

T11b= 6.7464766976160773E-003 T12b= 1.0569491871585575 T22b= 1.0518778264635178

TTb= 1.0423393578909661 TSb= 0.94736469148692759

FATb= 9.1116837990466992E-002 T11/T22= 6.41374569E-03

Alleles sampled from the same population

T11w= 4.4584653106743977E-003 T12w= 1.0572133932301888 T22w= 0.99403675549843551

TTw= 0.99551276738827355 TSw= 0.89507892647965948

FATw= 0.10088654229126781 T11/T22= 4.48521180E-03

Time (units of 2NT generations) = 7.49999983E-03

Alleles sampled from different populations

T11b= 1.0070110694841428E-002 T12b= 1.0593938702377566 T22b= 1.0516183418008593

TTb= 1.0426024546084407 TSb= 0.94746351869025758

FATb= 9.1251402198082698E-002 T11/T22= 9.57582239E-03

Alleles sampled from the same population

T11w= 6.6385403280067035E-003 T12w= 1.0596580710242822 T22w= 0.99379154430497163

TTw= 0.99577598907467801 TSw= 0.89507624390727514

FATw= 0.10112690632456189 T11/T22= 6.68001268E-03

Time (units of 2NT generations) = 9.99999978E-03

Alleles sampled from different populations

T11b= 1.3338828704771988E-002 T12b= 1.0618373792850706 T22b= 1.0513598076241786

TTb= 1.0428655607339452 TSb= 0.94755770973223796

FATb= 9.1390352304501987E-002 T11/T22= 1.26872156E-02

Alleles sampled from the same population

T11w= 8.7825950752806747E-003 T12w= 1.0621015748424458 T22w= 0.99354723131943679

TTw= 0.99603936679113692 TSw= 0.89507076769502114

FATw= 0.10137008883634646 T11/T22= 8.83963518E-03

Time (units of 2NT generations) = 1.49999997E-02

Alleles sampled from different populations

T11b= 1.9715270168932930E-002 T12b= 1.0667208685157332 T22b= 1.0508455799173255

TTb= 1.0433918287675552 TSb= 0.94773254894248626

FATb= 9.1681070512178331E-002 T11/T22= 1.87613387E-02

Alleles sampled from the same population

T11w= 1.2965105676667199E-002 T12w= 1.0669850538979944 T22w= 0.99306128975211483

TTw= 0.99656660545761877 TSw= 0.89505167134456998

FATw= 0.10186467573477798 T11/T22= 1.30556952E-02

Time (units of 2NT generations) = 1.99999996E-02

Alleles sampled from different populations

T11b= 2.5883147648977922E-002 T12b= 1.0715996417725355 T22b= 1.0503351217919308

TTb= 1.0439182156470102 TSb= 0.94788992437763553

FATb= 9.1988328041442635E-002 T 11/T22= 2.46427525E-02

Alleles sampled from the same population

T11w= 1.7010815836103754E-002 T12w= 1.0718638173463848 T22w= 0.99257891042969593

TTw= 0.99709451272876415 TSw= 0.89502210097033674

FATw= 0.10236984604306387 T11/T22= 1.71379987E-02

Time (units of 2NT generations) = 2.99999993E-02

Alleles sampled from different populations

T11b= 3.7621354019372610E-002 T12b= 1.0813429915999757 T22b= 1.0493254286540543

TTb= 1.0449715492379734 TSb= 0.94815502119058614

FATb= 9.2649917711146279E-002 T11/T22= 3.58528942E-02

Alleles sampled from the same population

T11w= 2.4710294494734719E-002 T12w= 1.0816071486065439 T22w= 0.99162475759796798

TTw= 0.99815244334847941 TSw= 0.89493331128764475

FATw= 0.10341018824195614 T11/T22= 2.49189977E-02

Time (units of 2NT generations) = 3.99999991E-02

Alleles sampled from different populations

T11b= 4.8607366148727404E-002 T12b= 1.0910673379847209 T22b= 1.0483305580861702

TTb= 1.0460259465485349 TSb= 0.94835823889242588

FATb= 9.3370253365485989E-002 T11/T22= 4.63664494E-02

Alleles sampled from the same population

T11w= 3.1916396917245904E-002 T12w= 1.0913314777433349 T22w= 0.99068461205698122

TTw= 0.99921336572912767 TSw= 0.89480779054300763

FATw= 0.10448776884598132 T11/T22= 3.22165079E-02

Time (units of 2NT generations) = 5.00000007E-02

Alleles sampled from different populations

T11b= 5.8891491227946458E-002 T12b= 1.1007726004132197 T22b= 1.0473503417746532

TTb= 1.0470817598241282 TSb= 0.94850445671998251

FATb= 9.4144800230980152E-002 T11/T22= 5.62290289E-02

Alleles sampled from the same population

T11w= 3.8662120366150798E-002 T12w= 1.1010367241528698 T22w= 0.98975831475062659

TTw= 1.0002774664991856 TSw= 0.89464869531217905

FATw= 0.10559947087150801 T11/T22= 3.90621834E-02

Time (units of 2NT generations) = 7.50000030E-02

Alleles sampled from different populations

T11b= 8.1831394055967288E-002 T12b= 1.1249518202394944 T22b= 1.0449628249641314

TTb= 1.0497295298046152 TSb= 0.94864968187331500

FATb= 9.6291325585662113E-002 T11/T22= 7.83103406E-02

Alleles sampled from the same population

T11w= 5.3709272537144637E-002 T12w= 1.1252159087691318 T22w= 0.98750212897188350

TTw= 1.0029526807710409 TSw= 0.89412284332840963

FATw= 0.10850944369475746 T11/T22= 5.43890186E-02

Time (units of 2NT generations) = 0.100000001

Alleles sampled from different populations

T11b= 0.10132093562612965 T12b= 1.1490104681087905 T22b= 1.0426633078531093

TTb= 1.0523923729768623 TSb= 0.94852907063041147

FATb= 9.8692564687310136E-002 T11/T22= 9.71751213E-02

Alleles sampled from the same population

T11w= 6.6493276849236282E-002 T12w= 1.1492745274284513 T22w= 0.98532910267990503

TTw= 1.0056509208763367 TSw= 0.89344552009683820

FATw= 0.11157489984866842 T11/T22= 6.74833208E-02

Time (units of 2NT generations) = 0.125000000

Alleles sampled from different populations

T11b= 0.11790809410286873 T12b= 1.1729479569662500 T22b= 1.0404493048602468

TTb= 1.0550736501317537 TSb= 0.94819518378450895

FATb= 0.10129953139659786 T11/T22= 0.113324210

Alleles sampled from the same population

T11w= 7.7373560639361905E-002 T12w= 1.1732119920932362 T22w= 0.98323688700461576

TTw= 1.0083737726569151 TSw= 0.89265055436809038

FATw= 0.11476222550286208 T11/T22= 7.86926970E-02

Time (units of 2NT generations) = 0.150000006

Alleles sampled from different populations

T11b= 0.13205365639991251 T12b= 1.1967639422087446 T22b= 1.0383183967851957

TTb= 1.0577759475575816 TSb= 0.94769192274666736

FATb= 0.10407121192829139 T11/T22= 0.127180308

Alleles sampled from the same population

T11w= 8.6652361160363400E-002 T12w= 1.1970279573348943 T22w= 0.98122319580599004

TTw= 1.0111223445347366 TSw= 0.89176611234142733

FATw= 0.11804331378734445 T11/T22= 8.83105546E-02

Time (units of 2NT generations) = 0.174999997

Alleles sampled from different populations

T11b= 0.14414509279300491 T12b= 1.2204582809069704 T22b= 1.0362682292421215

TTb= 1.0605012071773032 TSb= 0.94705591559720981

FATb= 0.10697327906117760 T11/T22= 0.139100179

Alleles sampled from the same population

T11w= 9.4583826153097905E-002 T12w= 1.2207222795307473 T22w= 0.97928580419370781

TTw= 1.0138973499739690 TSw= 0.89081560638964685

FATw= 0.12139467924191949 T11/T22= 9.65844989E-02

Time (units of 2NT generations) = 0.200000003

Alleles sampled from different populations

T11b= 0.15450822366840700 T12b= 1.2440309975679549 T22b= 1.0342965110984244

TTb= 1.0632508357886399 TSb= 0.94631768235542268

FATb= 0.10997701529807402 T11/T22= 0.149384841

Alleles sampled from the same population

T11w= 0.10138166624641232 T12w= 1.2442949826057017 T22w= 0.97742254705172904

TTw= 1.0166991766433910 TSw= 0.88981845897119749

FATw= 0.12479671527923075 T11/T22= 0.103723481

Time (units of 2NT generations) = 0.300000012

Alleles sampled from different populations

T11b= 0.18354396307121601 T12b= 1.3371105084476804 T22b= 1.0271504373820397

TTb= 1.0745071854307469 TSb= 0.94278978995095741

FATb= 0.12258400619907139 T11/T22= 0.178692386

Alleles sampled from the same population

T11w= 0.12042864015872651 T12w= 1.3373744596334927 T22w= 0.97066957136775711

TTw= 1.0281740419434993 TSw= 0.88564547824685402

FATw= 0.13862299365896413 T11/T22= 0.124067597

Time (units of 2NT generations) = 0.400000006

Alleles sampled from different populations

T11b= 0.20045399473187009 T12b= 1.4282735112757523 T22b= 1.0210916712834137

TTb= 1.0861780257165194 TSb= 0.93902790362825939

FATb= 0.13547514183155152 T11/T22= 0.196313411

Alleles sampled from the same population

T11w= 0.13152213406493557 T12w= 1.4285374476777815 T22w= 0.96494409831682160

TTw= 1.0400566815592756 TSw= 0.88160190189163301

FATw= 0.15235206164925907 T11/T22= 0.136300266

Time (units of 2NT generations) = 0.500000000

Alleles sampled from different populations

T11b= 0.21125199748814771 T12b= 1.5175589124637296 T22b= 1.0160007033715208

TTb= 1.0982336939492847 TSb= 0.93552583278318358

FATb= 0.14815413337119376 T11/T22= 0.207925051

Alleles sampled from the same population

T11w= 0.13860661371155519 T12w= 1.5178228426047138 T22w= 0.96013319172994960

TTw= 1.0523020631072233 TSw= 0.87798053392811015

FATw= 0.16565731009248319 T11/T22= 0.144361854

Time (units of 2NT generations) = 1.00000000

Alleles sampled from different populations

T11b= 0.23957773776084629 T12b= 1.9374073079670113 T22b= 1.0016871093641289

TTb= 1.1624956513966149 TSb= 0.92547617220380063

FATb= 0.20388848672944337 T11/T22= 0.239174232

Alleles sampled from the same population

T11w= 0.15719485178812359 T12w= 1.9376712242319751 T22w= 0.94660710064699960

TTw= 1.1171045204037064 TSw= 0.86766587576111209

FATw= 0.22329033683656618 T11/T22= 0.166061342

Time (units of 2NT generations) = 1.50000000

Alleles sampled from different populations

T11b= 0.26008355029049385 T12b= 2.3168754098339157 T22b= 0.99951774776806368

TTb= 1.2292477849651415 TSb= 0.92557432802030670

FATb= 0.24704006845409632 T11/T22= 0.260209024

Alleles sampled from the same population

T11w= 0.17065301783823927 T12w= 2.3171392948359184 T22w= 0.94455726461575307

TTw= 1.1838829875876078 TSw= 0.86716683993800170

FATw= 0.26752318512066542 T11/T22= 0.180669844

Time (units of 2NT generations) = 2.00000000

Alleles sampled from different populations

T11b= 0.27848545533982638 T12b= 2.6605415644119130 T22b= 1.0038059248915985

TTb= 1.2947651353097376 TSb= 0.93127387793642125

FATb= 0.28073914523992871 T11/T22= 0.277429581

Alleles sampled from the same population

T11w= 0.18273043548912446 T12w= 2.6608054051927867 T22w= 0.94860977116758594

TTw= 1.2491461919353375 TSw= 0.87202183759973984

FATw= 0.30190569908499520 T11/T22= 0.192629725

Time (units of 2NT generations) = 2.50000000

Alleles sampled from different populations

T11b= 0.29516222184379493 T12b= 2.9721942242999573 T22b= 1.0113184217935225

TTb= 1.3571145042451835 TSb= 0.93970280179854981

FATb= 0.30757294328586871 T11/T22= 0.291858852

Alleles sampled from the same population

T11w= 0.19367562587669515 T12w= 2.9724580156026139 T22w= 0.95570923647972339

TTw= 1.3111036806158136 TSw= 0.87950587541942049

FATw= 0.32918663228347855 T11/T22= 0.202651203

Time (units of 2NT generations) = 3.00000000

Alleles sampled from different populations

T11b= 0.31029463010489505 T12b= 3.2550537354883620 T22b= 1.0202341977960945

TTb= 1.4154023189037908 TSb= 0.94924024102697457

FATb= 0.32934952250032501 T11/T22= 0.304140598

Alleles sampled from the same population

T11w= 0.20360723422434598 T12w= 3.2553174764522543 T22w= 0.96413478225241689

TTw= 1.3689423917281069 TSw= 0.88808202744960973

FATw= 0.35126413440340265 T11/T22= 0.211181298

Time (units of 2NT generations) = 4.00000000

Alleles sampled from different populations

T11b= 0.33651447494142350 T12b= 3.7452561041275851 T22b= 1.0387011122194600

TTb= 1.5188591443901422 TSb= 0.96848244849165643

FATb= 0.36236190691630921 T11/T22= 0.323976249

Alleles sampled from the same population

T11w= 0.22081568217043684 T12w= 3.7455197500428197 T22w= 0.98158624897813351

TTw= 1.4714865735017002 TSw= 0.90550919229736382

FATw= 0.38462966050548364 T11/T22= 0.224958003

Time (units of 2NT generations) = 5.00000000

Alleles sampled from different populations

T11b= 0.35815693729852965 T12b= 4.1499311136255574 T22b= 1.0555901447046452

TTb= 1.6055971870363481 TSb= 0.98584682396403367

FATb= 0.38599367766473569 T11/T22= 0.339295447

Alleles sampled from the same population

T11w= 0.23501993156700443 T12w= 4.1501946768174482 T22w= 0.99754657363251187

TTw= 1.5573979657851456 TSw= 0.92129390942596112

FATw= 0.40844027688099582 T11/T22= 0.235597953

**Table S5 Coalescence times for inversion and standard arrangements in a subdivided population**

**Time course of approach to equilibrium**

**Times scaled by 2x total population size**

**Inversion frequency= 0.5**

**Local N= 500**

**Total N= 10000**

**Number of populations = 200**

**Neutral Fst= 0.05**

**Scaled migration rate= 19 Migration rate= 0.0095**

Initial state of subdivided population

Alleles sampled from different populations

T11b= 0 T12b= 1.0524 T22b= 1.0524

TTb= 0.7893 TSb= 0.5263

FATb= 0.3333 T11/T22= 0

Alleles sampled from the same population

T11w= 0 T12w= 1 T22w= 1

TTw= 0.75 TSw= 0.5

FATw= 0.3333 T11/T22= 0

**Scaled recombination rate= 40**

Equilibrium results for subdivided population

Alleles sampled from different populations

T11b= 1.1052631578947374 T12b= 1.1552631578947374 T22b= 1.1052631578947374

TTb= 1.1302631578947373 TSb= 1.1052631578947374

FATb= 2.2118742724097751E-002 T11/T22= 1.00000000

Alleles sampled from the same population

T11w= 1.0004761904761910 T12w= 1.1555263157894742 T22w= 1.0004761904761910

TTw= 1.0780012531328327 TSw= 1.0004761904761910

FATw= 7.1915558939604440E-002 T11/T22= 1.00000000

Time (units of 2NT generations) = 2.49999994E-03

Alleles sampled from different populations

T11b= 5.2083751635259024E-002 T12b= 1.0299456493807646 T22b= 1.0498671724787803

TTb= 0.79046055571889218 TSb= 0.55097546205701964

FATb= 0.30296906269291379 T11/T22= 4.96098474E-02

Alleles sampled from the same population

T11w= 4.7134560465934561E-002 T12w= 1.0304821410766314 T22w= 0.95028085894582726

TTw= 0.76459492539125617 TSw= 0.49870770970588091

FATw= 0.34774912421674298 T11/T22= 4.96006645E-02

Time (units of 2NT generations) = 4.99999989E-03

Alleles sampled from different populations

T11b= 0.10140042620929490 T12b= 1.0095864870186666 T22b= 1.0462054464220933

TTb= 0.79169471166718042 TSb= 0.57380293631569412

FATb= 0.27522196642269037 T11/T22= 9.69220996E-02

Alleles sampled from the same population

T11w= 9.1764451539401160E-002 T12w= 1.0101130517181780 T22w= 0.94695682533440628

TTw= 0.76473684507754092 TSw= 0.51936063843690372

FATw= 0.32086358623894617 T11/T22= 9.69045758E-02

Time (units of 2NT generations) = 7.49999983E-03

Alleles sampled from different populations

T11b= 0.14715725029783910 T12b= 0.99128126930575478 T22b= 1.0417968142776135

TTb= 0.79287915079674054 TSb= 0.59447703228772630

FATb= 0.25022996040398571 T11/T22= 0.141253307

Alleles sampled from the same population

T11w= 0.13317301559050976 T12w= 0.99179746847834616 T22w= 0.94295801465413320

TTw= 0.76493149180033382 TSw= 0.53806551512232148

FATw= 0.29658339225132857 T11/T22= 0.141229004

Time (units of 2NT generations) = 9.99999978E-03

Alleles sampled from different populations

T11b= 0.18963837799664263 T12b= 0.97482988146787342 T22b= 1.0367760755267068

TTb= 0.79401855411477407 TSb= 0.61320722676167472

FATb= 0.22771675348906695 T11/T22= 0.182911605

Alleles sampled from the same population

T11w= 0.17161741841353551 T12w= 0.97533669036768877 T22w= 0.93840599698868987

TTw= 0.76517419903440065 TSw= 0.55501170770111274

FATw= 0.27465966782269857 T11/T22= 0.182881847

Time (units of 2NT generations)= 1.25000002E-02

Alleles sampled from different populations

T11b= 0.22910360664525675 T12b= 0.96005169683633140 T22b= 1.0312616012952232

TTb= 0.79511715040328568 TSb= 0.63018260397023995

FATb= 0.20743427097427147 T11/T22= 0.222158581

Alleles sampled from the same population

T11w= 0.20733273404807906 T12w= 0.96054999588477374 T22w= 0.93340783412177486

TTw= 0.76546013998485041 TSw= 0.57037028408492696

FATw= 0.25486612000957298 T11/T22= 0.222124487

Time (units of 2NT generations)= 1.49999997E-02

Alleles sampled from different populations

T11b= 0.26579057223879654 T12b= 0.94678367285228759 T22b= 1.0253571105441193

TTb= 0.79617875712187280 TSb= 0.64557384139145790

FATb= 0.18915967599391936 T11/T22= 0.259217560

Alleles sampled from the same population

T11w= 0.24053398513454671 T12w= 0.94727425683748967 T22w= 0.92805733339130059

TTw= 0.76578495805020663 TSw= 0.58429565926292359

FATw= 0.23699773269166802 T11/T22= 0.259180099

Time (units of 2NT generations) = 1.75000001E-02

Alleles sampled from different populations

T11b= 0.29991674065355078 T12b= 0.93487863927684900 T22b= 1.0191532625869797

TTb= 0.79720682044855717 TSb= 0.65953500162026524

FATb= 0.17269272577325590 T11/T22= 0.294280320

Alleles sampled from the same population

T11w= 0.27141794450004308 T12w= 0.93536222568978833 T22w= 0.92243648838097914

TTw= 0.76614472106514964 TSw= 0.59692721644051105

FATw= 0.22086885150024949 T11/T22= 0.294240266

Time (units of 2NT generations) = 1.99999996E-02

Alleles sampled from different populations

T11b= 0.33168121385146909 T12b= 0.92420375304173008 T22b= 1.0127290857562110

TTb= 0.79820445142278507 TSb= 0.67220514980384005

FATb= 0.15785341887576987 T11/T22= 0.327512294

Alleles sampled from the same population

T11w= 0.30016476934603409 T12w= 0.92468098960083722 T22w= 0.91661677137836739

TTw= 0.76653587998151895 TSw= 0.60839077036220068

FATw= 0.20631142487828635 T11/T22= 0.327470303

Time (units of 2NT generations)= 2.50000004E-02

Alleles sampled from different populations

T11b= 0.38883934361364947 T12b= 0.90607645356045308 T22b= 0.99948525866518301

TTb= 0.80011937734993466 TSb= 0.69416230113941624

FATb= 0.13242658434477306 T11/T22= 0.389039606

Alleles sampled from the same population

T11w= 0.35189332495910053 T12w= 0.90654268777629077 T22w= 0.90462083853840403

TTw= 0.76739988476252152 TSw= 0.62825708174875228

FATw= 0.18131720603115309 T11/T22= 0.388995379

Time (units of 2NT generations)= 2.74999999E-02

Alleles sampled from different populations

T11b= 0.41455338931886132 T12b= 0.89841810310850301 T22b= 0.99277639142006358

TTb= 0.80104149673898273 TSb= 0.70366489036946245

FATb= 0.12156249927867369 T11/T22= 0.417569757

Alleles sampled from the same population

T11w= 0.37516496306349789 T12w= 0.89887957666985896 T22w= 0.89854480458554353

TTw= 0.76786723024718984 TSw= 0.63685488382452071

FATw= 0.17061848879850483 T11/T22= 0.417525053

Time (units of 2NT generations)= 2.99999993E-02

Alleles sampled from different populations

T11b= 0.43854909343857607 T12b= 0.89157586341537520 T22b= 0.98607071262554291

TTb= 0.80194288322371732 TSb= 0.71230990303205943

FATb= 0.11176978069977217 T11/T22= 0.444744051

Alleles sampled from the same population

T11w= 0.39688161142349410 T12w= 0.89203300657502838 T22w= 0.89247202764463229

TTw= 0.76835491305454573 TSw= 0.64467681953406319

FATw= 0.16096479819307485 T11/T22= 0.444699228

Time (units of 2NT generations)= 3.24999988E-02

Alleles sampled from different populations

T11b= 0.46095549389125301 T12b= 0.88547013113366146 T22b= 0.97940585367935684

TTb= 0.80282540245948320 TSb= 0.72018067378530493

FATb= 0.10294234390316159 T11/T22= 0.470648080

Alleles sampled from the same population

T11w= 0.41716003281531910 T12w= 0.88592333233327014 T22w= 0.88643653367554298

TTw= 0.76886080778935062 TSw= 0.65179828324543099

FATw= 0.15225450869384405 T11/T22= 0.470603377

Time (units of 2NT generations)= 3.50000001E-02

Alleles sampled from different populations

T11b= 0.48189108974619921 T12b= 0.88002905253776009 T22b= 0.97281376045927215

TTb= 0.80369073882024789 TSb= 0.72735242510273568

FATb= 9.4984687554780844E-002 T11/T22= 0.495358020

Alleles sampled from the same population

T11w= 0.43610745427493547 T12w= 0.88047866247795947 T22w= 0.88046720589965233

TTw= 0.76938299628262663 TSw= 0.65828733008729390

FATw= 0.14439579082473331 T11/T22= 0.495313674

Time (units of 2NT generations)= 3.75000015E-02

Alleles sampled from different populations

T11b= 0.50146475977923988 T12b= 0.87518776910807994 T22b= 0.96632135418599308

TTb= 0.80454041304534818 TSb= 0.73389305698261653

FATb= 8.7810823318765441E-002 T11/T22= 0.518941998

Alleles sampled from the same population

T11w= 0.45382239818786640 T12w= 0.87563410442235001 T22w= 0.87458838263845284

TTw= 0.76991974741775482 TSw= 0.66420539041315962

FATw= 0.13730568329900894 T11/T22= 0.518898249

Time (units of 2NT generations) = 3.99999991E-02

Alleles sampled from different populations

T11b= 0.51977659731008319 T12b= 0.87088773655656027 T22b= 0.95995112225016888

TTb= 0.80537579816834315 TSb= 0.73986385978012603

FATb= 8.1343316420992684E-002 T11/T22= 0.541461527

Alleles sampled from the same population

T11w= 0.47039543763455760 T12w= 0.87133108312746033 T22w= 0.86882039179240467

TTw= 0.77046949892047067 TSw= 0.66960791471348113

FATw= 0.13090924994215858 T11/T22= 0.541418493

Time (units of 2NT generations) = 4.25000004E-02

Alleles sampled from different populations

T11b= 0.53691866916846798 T12b= 0.86707611014413755 T22b= 0.95372164612418819

TTb= 0.80619813389523276 TSb= 0.74532015764632809

FATb= 7.5512425158771102E-002 T11/T22= 0.562972069

Alleles sampled from the same population

T11w= 0.48590988309086258 T12w= 0.86751672609670172 T22w= 0.86318002840206876

TTw= 0.77103084092158380 TSw= 0.67454495574646567

FATw= 0.12513881423963824 T11/T22= 0.562929928

Time (units of 2NT generations) = 4.50000018E-02

Alleles sampled from different populations

T11b= 0.55297570588835443 T12b= 0.86370518983671696 T22b= 0.94764807277009899

TTb= 0.80700853958297181 TSb= 0.75031188932922666

FATb= 7.0255328751593682E-002 T11/T22= 0.583524346

Alleles sampled from the same population

T11w= 0.50044240690666930 T12w= 0.86414330824045449 T22w= 0.85768098109183333

TTw= 0.77160250111985296 TSw= 0.67906169399925131

FATw= 0.11993326484335398 T11/T22= 0.583483160

Time (units of 2NT generations) = 4.74999994E-02

Alleles sampled from different populations

T11b= 0.56802572955357955 T12b= 0.86073191947443994 T22b= 0.94174253531570751

TTb= 0.80780802595454171 TSb= 0.75488413243464358

FATb= 6.5515434137165096E-002 T11/T22= 0.603164554

Alleles sampled from the same population

T11w= 0.51406361137412648 T12w= 0.86116775078228924 T22w= 0.85233421261816811

TTw= 0.77218333138921835 TSw= 0.68319891199614724

FATw= 0.11523742584935259 T11/T22= 0.603124440

Time (units of 2NT generations) = 5.00000007E-02

Alleles sampled from different populations

T11b= 0.58214062510749010 T12b= 0.85811743469614998 T22b= 0.93601452819453790

TTb= 0.80859750567358202 TSb= 0.75907757665101405

FATb= 6.1241753375576691E-002 T11/T22= 0.621935487

Alleles sampled from the same population

T11w= 0.52683854564435750 T12w= 0.85855116894601979 T22w= 0.84714829922291091

TTw= 0.77277229568982708 TSw= 0.68699342243363426

FATw= 0.11100148612292182 T11/T22= 0.621896505

Time (units of 2NT generations) = 5.99999987E-02

Alleles sampled from different populations

T11b= 0.63049967872365331 T12b= 0.85059500715590475 T22b= 0.91499306075910258

TTb= 0.81167068844864132 TSb= 0.77274636974137789

FATb= 4.7955801855627089E-002 T11/T22= 0.689075887

Alleles sampled from the same population

T11w= 0.57060740565926249 T12w= 0.85102191087224521 T22w= 0.82811722568147683

TTw= 0.77519211327130744 TSw= 0.69936231567036966

FATw= 9.7820651555569071E-002 T11/T22= 0.689041853

Time (units of 2NT generations) = 7.00000003E-02

Alleles sampled from different populations

T11b= 0.66836822165132370 T12b= 0.84654804035709896 T22b= 0.89708382830079714

TTb= 0.81463703266657972 TSb= 0.78272602497606036

FATb= 3.9172056278934386E-002 T11/T22= 0.745045424

Alleles sampled from the same population

T11w= 0.60488233650300816 T12w= 0.84696996423376270 T22w= 0.81190477858300869

TTw= 0.77768176088838548 TSw= 0.70839355754300848

FATw= 8.9095831778574275E-002 T11/T22= 0.745016336

Time (units of 2NT generations) = 7.99999982E-02

Alleles sampled from different populations

T11b= 0.69831179461861681 T12b= 0.84479996409521241 T22b= 0.88218541709366360

TTb= 0.81752428497567631 TSb= 0.79024860585614021

FATb= 3.3363753983586708E-002 T11/T22= 0.791570306

Alleles sampled from the same population

T11w= 0.63198500828123338 T12w= 0.84521814067005230 T22w= 0.79841862825374244

TTw= 0.78020997946877002 TSw= 0.71520181826748797

FATw= 8.3321365929649893E-002 T 11/T22= 0.791545928

Time (units of 2NT generations) = 9.00000036E-02

Alleles sampled from different populations

T11b= 0.72222042828719690 T12b= 0.84456976744984302 T22b= 0.87004378646281588

TTb= 0.82035093741242471 TSb= 0.79613210737500639

FATb= 2.9522523755272423E-002 T11/T22= 0.830096662

Alleles sampled from the same population

T11w= 0.65362583145642972 T12w= 0.84498501885358202 T22w= 0.78742847419818540

TTw= 0.78275608584044476 TSw= 0.72052715282730762

FATw= 7.9499775394683780E-002 T11/T22= 0.830076456

Time (units of 2NT generations) = 0.125000000

Alleles sampled from different populations

T11b= 0.77573769028189077 T12b= 0.84965176325765934 T22b= 0.84460700730281646

TTb= 0.82991205602500651 TSb= 0.81017234879235356

FATb= 2.3785300007809651E-002 T11/T22= 0.918459952

Alleles sampled from the same population

T11w= 0.70206932373204189 T12w= 0.85006006203488382 T22w= 0.76440653981850115

TTw= 0.79164899690507773 TSw= 0.73323793177527152

FATw= 7.3784044896364587E-002 T11/T22= 0.918450177

Time (units of 2NT generations) = 0.150000006

Alleles sampled from different populations

T11b= 0.79760803920473855 T12b= 0.85545518701349788 T22b= 0.83751832668300197

TTb= 0.83650918497868409 TSb= 0.81756318294387031

FATb= 2.2648887035587761E-002 T11/T22= 0.952346981

Alleles sampled from the same population

T11w= 0.72186789637115056 T12w= 0.85585984141753524 T22w= 0.75799278220164623

TTw= 0.79789509035196682 TSw= 0.73993033928639840

FATw= 7.2647083265043100E-002 T11/T22= 0.952341378

Time (units of 2NT generations) = 0.174999997

Alleles sampled from different populations

T11b= 0.81263220654527069 T12b= 0.86170074113403039 T22b= 0.83576051847717747

TTb= 0.84294855182262718 TSb= 0.82419636251122408

FATb= 2.2245947597699822E-002 T11/T22= 0.972326636

Alleles sampled from the same population

T11w= 0.73546983475853367 T12w= 0.86210212990285939 T22w= 0.75640447794914678

TTw= 0.80401964312834984 TSw= 0.74593715635384017

FATw= 7.2240134020255087E-002 T11/T22= 0.972323477

Time (units of 2NT generations) = 0.200000003

Alleles sampled from different populations

T11b= 0.82376883497614184 T12b= 0.86801393789540426 T22b= 0.83717186578896241

TTb= 0.84924214413897825 TSb= 0.83047035038255212

FATb= 2.2104171214275214E-002 T11/T22= 0.983990133

Alleles sampled from the same population

T11w= 0.74555299066319913 T12w= 0.86841223987845018 T22w= 0.75768477393234912

TTw= 0.81001556108811212 TSw= 0.75161888229777407

FATw= 7.2093280173398777E-002 T11/T22= 0.983988345

Time (units of 2NT generations) = 0.250000000

Alleles sampled from different populations

T11b= 0.84017909075097374 T12b= 0.88040020298959842 T22b= 0.84468021680945116

TTb= 0.86141492838490541 TSb= 0.84242965378021251

FATb= 2.2039639642987163E-002 T11/T22= 0.994671226

Alleles sampled from the same population

T11w= 0.76041228856877052 T12w= 0.88079262277321735 T22w= 0.76448649287160131

TTw= 0.82162100674670158 TSw= 0.76244939072018592

FATw= 7.2018139191465158E-002 T11/T22= 0.994670689

Time (units of 2NT generations) = 0.300000012

Alleles sampled from different populations

T11b= 0.85306503752469620 T12b= 0.89229789475776300 T22b= 0.85457664606138661

TTb= 0.87305936827540220 TSb= 0.85382084179304141

FATb= 2.2035759744911343E-002 T11/T22= 0.998231173

Alleles sampled from the same population

T11w= 0.77208133307913562 T12w= 0.89268471226535440 T22w= 0.77344956892153749

TTw= 0.83272508163284553 TSw= 0.77276545100033656

FATw= 7.2004112707806711E-002 T11/T22= 0.998230994

Time (units of 2NT generations) = 0.400000006

Alleles sampled from different populations

T11b= 0.87504574748456021 T12b= 0.91458255031192248 T22b= 0.87521622835565283

TTb= 0.89485676911601453 TSb= 0.87513098792010657

FATb= 2.2043506711575844E-002 T11/T22= 0.999805212

Alleles sampled from the same population

T11w= 0.79198715082130255 T12w= 0.91495888745545528 T22w= 0.79214146196245516

TTw= 0.85351159692366707 TSw= 0.79206430639187886

FATw= 7.1993503958545091E-002 T11/T22= 0.999805212

Time (units of 2NT generations) = 0.500000000

Alleles sampled from different populations

T11b= 0.89462451727924086 T12b= 0.93497961309833078 T22b= 0.89464374429913796

TTb= 0.91480687194376009 TSb= 0.89463413078918941

FATb= 2.2051366002212136E-002 T11/T22= 0.999978483

Alleles sampled from the same population

T11w= 0.80971800604186162 T12w= 0.93534635857504567 T22w= 0.80973540942185551

TTw= 0.87253653315345203 TSw= 0.80972670773185862

FATw= 7.1985324436320330E-002 T11/T22= 0.999978483

Time (units of 2NT generations) = 0.750000000

Alleles sampled from different populations

T11b= 0.93646385887186046 T12b= 0.97872671597655581 T22b= 0.93646394100204344

TTb= 0.95759530795675385 TSb= 0.93646389993695189

FATb= 2.2067159105959466E-002 T11/T22= 0.999999940

Alleles sampled from the same population

T11w= 0.84760847127002992 T12w= 0.97907288952782967 T22w= 0.84760854561034871

TTw= 0.91334069898400949 TSw= 0.84760850844018931

FATw= 7.1968971290713335E-002 T11/T22= 0.999999940

Time (units of 2NT generations) = 1.00000000

Alleles sampled from different populations

T11b= 0.96998650605415071 T12b= 1.0137858845435095 T22b= 0.96998650640497985

TTb= 0.99188619538653733 TSb= 0.96998650622956528

FATb= 2.2078832489888400E-002 T11/T22= 1.00000000

Alleles sampled from the same population

T11w= 0.87796719102537768 T12w= 1.0141155716409691 T22w= 0.87796719134293200

TTw= 0.94604138141256200 TSw= 0.87796719118415489

FATw= 7.1956884303267521E-002 T11/T22= 1.00000000

Time (units of 2NT generations) = 1.50000000

Alleles sampled from different populations

T11b= 1.0183816261088150 T12b= 1.0643992721439750 T22b= 1.0183816261088197

TTb= 1.0413904491263961 TSb= 1.0183816261088174

FATb= 2.2094328824390885E-002 T11/T22= 1.00000000

Alleles sampled from the same population

T11w= 0.92179469307782758 T12w= 1.0647051584694491 T22w= 0.92179469307783191

TTw= 0.99324992577363935 TSw= 0.92179469307782980

FATw= 7.1940838696920362E-002 T11/T22= 1.00000000

Time (units of 2NT generations) = 2.00000000

Alleles sampled from different populations

T11b= 1.0494634284325481 T12b= 1.0969057585884152 T22b= 1.0494634284325481

TTb= 1.0731845935104818 TSb= 1.0494634284325481

FATb= 2.2103527409333745E-002 T11/T22= 1.00000000

Alleles sampled from the same population

T11w= 0.94994293929771645 T12w= 1.0971963588500910 T22w= 0.94994293929771656

TTw= 1.0235696490739037 TSw= 0.94994293929771656

FATw= 7.1931313948984776E-002 T11/T22= 1.00000000

Time (units of 2NT generations) = 3.00000000

Alleles sampled from different populations

T11b= 1.0822465488219428 T12b= 1.1311915457330914 T22b= 1.0822465488219428

TTb= 1.1067190472775172 TSb= 1.0822465488219428

FATb= 2.2112656790154395E-002 T11/T22= 1.00000000

Alleles sampled from the same population

T11w= 0.97963193014083538 T12w= 1.1314660232209151 T22w= 0.97963193014083538

TTw= 1.0555489766808752 TSw= 0.97963193014083538

FATw= 7.1921860773109247E-002 T11/T22= 1.00000000

Time (units of 2NT generations) = 4.00000000

Alleles sampled from different populations

T11b= 1.0957691281150981 T12b= 1.1453339540757126 T22b= 1.0957691281150981

TTb= 1.1205515410954052 TSb= 1.0957691281150981

FATb= 2.2116263350172027E-002 T11/T22= 1.00000000

Alleles sampled from the same population

T11w= 0.99187822421050276 T12w= 1.1456017811445021 T22w= 0.99187822421050276

TTw= 1.0687400026775025 TSw= 0.99187822421050276

FATw= 7.1918126274340577E-002 T11/T22= 1.00000000

Time (units of 2NT generations) = 5.00000000

Alleles sampled from different populations

T11b= 1.1013470032095882 T12b= 1.1511674999962267 T22b= 1.1013470032095880

TTb= 1.1262572516029075 TSb= 1.1013470032095882

FATb= 2.2117725198098848E-002 T11/T22= 1.00000000

Alleles sampled from the same population

T11w= 0.99692964947562024 T12w= 1.1514325838600479 T22w= 0.99692964947562024

TTw= 1.0741811166678341 TSw= 0.99692964947562024

FATw= 7.1916612565162130E-002 T11/T22= 1.00000000

**Scaled recombination rate= 4**

Equilibrium results for subdivided population

Alleles sampled from different populations

T11b= 1.1052631578947369 T12b= 1.6052631578947369 T22b= 1.1052631578947369

TTb= 1.3552631578947369 TSb= 1.1052631578947369

FATb= 0.18446601941747576 T11/T22= 1.00000000

Alleles sampled from the same population

T11w= 1.0004761904761905 T12w= 1.6055263157894737 T22w= 1.0004761904761905

TTw= 1.3030012531328321 TSw= 1.0004761904761905

FATw= 0.23217557306969161 T11/T22= 1.00000000

Time (units of 2NT generations) = 2.49999994E-03

Alleles sampled from different populations

T11b= 7.5735331463877564E-003 T12b= 1.0519867248969148 T22b= 1.0503733529514205

TTb= 0.79048008397290948 TSb= 0.52897344304890415

FATb= 0.33082002472432614 T11/T22= 7.21032498E-03

Alleles sampled from the same population

T11w= 6.8751087957386048E-003 T12w= 1.0522768759780301 T22w= 0.95076812472941996

TTw= 0.76554924637030464 TSw= 0.47882161676257928

FATw= 0.37453845192479229 T11/T22= 7.23110978E-03

Time (units of 2NT generations) = 4.99999989E-03

Alleles sampled from different populations

T11b= 1.5228585495341562E-002 T12b= 1.0518799318867653 T22b= 1.0481389514391710

TTb= 0.79178185017701086 TSb= 0.53168376846725629

FATb= 0.32849715063764973 T11/T22= 1.45291667E-02

Alleles sampled from the same population

T11w= 1.3804097506943114E-002 T12w= 1.0521715656921351 T22w= 0.94874522172968123

TTw= 0.76672311265522364 TSw= 0.48127465961831217

FATw= 0.37229665876170159 T11/T22= 1.45498468E-02

Time (units of 2NT generations) = 7.49999983E-03

Alleles sampled from different populations

T11b= 2.2810543286337914E-002 T12b= 1.0517871274496247 T22b= 1.0459252406773387

TTb= 0.79307750971573143 TSb= 0.53436789198183832

FATb= 0.32620975196563606 T11/T22= 2.18089614E-02

Alleles sampled from the same population

T11w= 2.0666919237205539E-002 T12w= 1.0520786279060736 T22w= 0.94674147219257332

TTw= 0.76789141181048159 TSw= 0.48370419571488943

FATw= 0.37008776465614468 T11/T22= 2.18295269E-02

Time (units of 2NT generations) = 9.99999978E-03

Alleles sampled from different populations

T11b= 3.0320168808246617E-002 T12b= 1.0517081039232865 T22b= 1.0437320954674920

TTb= 0.79436711803057791 TSb= 0.53702613213786932

FATb= 0.32395724854613461 T11/T22= 2.90497616E-02

Alleles sampled from the same population

T11w= 2.7464269873437308E-002 T12w= 1.0519994722775141 T22w= 0.94475633823469862

TTw= 0.76905488816579104 TSw= 0.48611030405406797

FATw= 0.36791208074439352 T11/T22= 2.90702153E-02

Time (units of 2NT generations) = 1.25000002E-02

Alleles sampled from different populations

T11b= 3.7758216159404778E-002 T12b= 1.0516426643009911 T22b= 1.0415593889058608

TTb= 0.79565073341681192 TSb= 0.53965880253263276

FATb= 0.32173907486373732 T11/T22= 3.62516195E-02

Alleles sampled from the same population

T11w= 3.4196831980739528E-002 T12w= 1.0519339018243292 T22w= 0.94278970497705505

TTw= 0.77021358515161320 TSw= 0.48849326847889729

FATw= 0.36576908289310484 T11/T22= 3.62719633E-02

Time (units of 2NT generations) = 1.49999997E-02

Alleles sampled from different populations

T11b= 4.5125431312036390E-002 T12b= 1.0515906141125151 T22b= 1.0394069943175013

TTb= 0.79692841346364196 TSb= 0.54226621281476883

FATb= 0.31955467561013440 T11/T22= 4.34145927E-02

Alleles sampled from the same population

T11w= 4.0865280768770385E-002 T12w= 1.0518817220608991 T22w= 0.94084145775077366

TTw= 0.77136754566033561 TSw= 0.49085336925977202

FATw= 0.36365825601390467 T11/T22= 4.34348211E-02

Time (units of 2NT generations) = 1.75000001E-02

Alleles sampled from different populations

T11b= 5.2422552201683015E-002 T12b= 1.0515517613917700 T22b= 1.0372747852666919

TTb= 0.79820021506297878 TSb= 0.54484866873418747

FATb= 0.31740350547111995 T11/T22= 5.05387336E-02

Alleles sampled from the same population

T11w= 4.7470284172901334E-002 T12w= 1.0518427410059374 T22w= 0.93891148210362962

TTw= 0.77251681207210143 TSw= 0.49319088313826548

FATw= 0.36157909390296294 T11/T22= 5.05588502E-02

Time (units of 2NT generations) = 1.99999996E-02

Alleles sampled from different populations

T11b= 5.9650308816008302E-002 T12b= 1.0515259166450239 T22b= 1.0351626355670520

TTb= 0.79946619441827704 TSb= 0.54740647219153016

FATb= 0.31528502891877175 T11/T22= 5.76240942E-02

Alleles sampled from the same population

T11w= 5.4012502934535078E-002 T12w= 1.0518167691506954 T22w= 0.93699966380920152

TTw= 0.77366142626128187 TSw= 0.49550608337186830

FATw= 0.35953109906693814 T11/T22= 5.76440990E-02

Time (units of 2NT generations) = 2.50000004E-02

Alleles sampled from different populations

T11b= 7.3900609953931884E-002 T12b= 1.0515125052722250 T22b= 1.0309980107814232

TTb= 0.80198090781995124 TSb= 0.55244931036767753

FATb= 0.31114406218295509 T11/T22= 7.16787130E-02

Alleles sampled from the same population

T11w= 6.6911194000691676E-002 T12w= 1.0518031071789649 T22w= 0.93323004355518369

TTw= 0.77593686297845132 TSw= 0.50007061877793768

FATw= 0.35552666378240461 T11/T22= 7.16985017E-02

Time (units of 2NT generations) = 2.99999993E-02

Alleles sampled from different populations

T11b= 8.7882018971749964E-002 T12b= 1.0515489123075537 T22b= 1.0269121158251859

TTb= 0.80447298985301074 TSb= 0.55739706739846795

FATb= 0.30712767932666984 T11/T22= 8.55789110E-02

Alleles sampled from the same population

T11w= 7.9566499009029457E-002 T12w= 1.0518392683427837 T22w= 0.92953168802717223

TTw= 0.77819418093044224 TSw= 0.50454909351810084

FATw= 0.35164113805780406 T11/T22= 8.55984762E-02

Time (units of 2NT generations) = 3.99999991E-02

Alleles sampled from different populations

T11b= 0.11506029198399270 T12b= 1.0517654978504229 T22b= 1.0189725221177615

TTb= 0.80939095245064996 TSb= 0.56701640705087708

FATb= 0.29945299569499562 T11/T22= 0.112917952

Alleles sampled from the same population

T11w= 0.10416698282521342 T12w= 1.0520553758739439 T22w= 0.92234515636396341

TTw= 0.78265572273426620 TSw= 0.51325606959458847

FATw= 0.34421220635621230 T11/T22= 0.112937093

Time (units of 2NT generations) = 5.00000007E-02

Alleles sampled from different populations

T11b= 0.14122821611901482 T12b= 1.0521646666395377 T22b= 1.0113359182291699

TTb= 0.81422336690681507 TSb= 0.57628206717409236

FATb= 0.29223098894428345 T11/T22= 0.139645204

Alleles sampled from the same population

T11w= 0.12785295532765195 T12w= 1.0524540842361221 T22w= 0.91543288484042273

TTw= 0.78704850216007971 TSw= 0.52164292008403734

FATw= 0.33721629778549644 T11/T22= 0.139663935

Time (units of 2NT generations) = 5.99999987E-02

Alleles sampled from different populations

T11b= 0.16642705764749022 T12b= 1.0527359692305047 T22b= 1.0039944541627310

TTb= 0.81897336256780773 TSb= 0.58521075590511062

FATb= 0.28543371170187781 T11/T22= 0.165764913

Alleles sampled from the same population

T11w= 0.15066176830262012 T12w= 1.0530249431541052 T22w= 0.90878776754375312

TTw= 0.79137485553864595 TSw= 0.52972476792318668

FATw= 0.33062724419942335 T11/T22= 0.165783226

Time (units of 2NT generations) = 7.00000003E-02

Alleles sampled from different populations

T11b= 0.19069633964784560 T12b= 1.0534694855866376 T22b= 0.99694037358068166

TTb= 0.82364392110045048 TSb= 0.59381835661426363

FATb= 0.27903510072547189 T11/T22= 0.191281587

Alleles sampled from the same population

T11w= 0.17262919570588786 T12w= 1.0537580318014164 T22w= 0.90240278336058732

TTw= 0.79563701066732695 TSw= 0.53751598953323754

FATw= 0.32442058083446224 T11/T22= 0.191299498

Time (units of 2NT generations) = 7.99999982E-02

Alleles sampled from different populations

T11b= 0.21407391733083481 T12b= 1.0543557986792071 T22b= 0.99016602043417401

TTb= 0.82823788378085572 TSb= 0.60211996888250441

FATb= 0.27301083339261989 T11/T22= 0.216200024

Alleles sampled from the same population

T11w= 0.19378950184174015 T12w= 1.0546439323984949 T22w= 0.89627100197695309

TTw= 0.79983709215392085 TSw= 0.54503025190934662

FATw= 0.31857342294340496 T11/T22= 0.216217533

Time (units of 2NT generations) = 9.00000036E-02

Alleles sampled from different populations

T11b= 0.23659605004186735 T12b= 1.0553859694012977 T22b= 0.98366384483999247

TTb= 0.83275795842111378 TSb= 0.61012994744092985

FATb= 0.26733819680604498 T11/T22= 0.240525305

Alleles sampled from the same population

T11w= 0.21417550653491046 T12w= 1.0556737051246459 T22w= 0.89038558919646904

TTw= 0.80397712649516784 TSw= 0.55228054786568981

FATw= 0.31306435262246335 T11/T22= 0.240542427

Time (units of 2NT generations) = 0.100000001

Alleles sampled from different populations

T11b= 0.25829747009029735 T12b= 1.0565515127297429 T22b= 0.97742640825735627

TTb= 0.83720672595178480 TSb= 0.61786193917382681

FATb= 0.26199596823424254 T11/T22= 0.264262825

Alleles sampled from the same population

T11w= 0.23381864743049152 T12w= 1.0568388642781115 T22w= 0.88473981162506299

TTw= 0.80805904690294428 TSw= 0.55927922952777731

FATw= 0.30787331486314984 T11/T22= 0.264279544

Time (units of 2NT generations) = 0.125000000

Alleles sampled from different populations

T11b= 0.30917531737170822 T12b= 1.0600057782125325 T22b= 0.96294329058788253

TTb= 0.84803254109616399 TSb= 0.63605930397979538

FATb= 0.24995884809133939 T11/T22= 0.321073234

Alleles sampled from the same population

T11w= 0.27987099906486179 T12w= 1.0602922256342775 T22w= 0.87163053988330930

TTw= 0.81802149755418152 TSw= 0.57575076947408554

FATw= 0.29616670075843476 T11/T22= 0.321089029

Time (units of 2NT generations) = 0.150000006

Alleles sampled from different populations

T11b= 0.35561067087572562 T12b= 1.0641418670700828 T22b= 0.94995827687335632

TTb= 0.85846317047231191 TSb= 0.65278447387454097

FATb= 0.23958942406883921 T11/T22= 0.374343455

Alleles sampled from the same population

T11w= 0.32190224827144398 T12w= 1.0644274835581160 T22w= 0.85987731101653320

TTw= 0.82765863160105235 TSw= 0.59088977964398859

FATw= 0.28607066116020519 T11/T22= 0.374358356

Time (units of 2NT generations) = 0.200000003

Alleles sampled from different populations

T11b= 0.43684521770118501 T12b= 1.0740682555313095 T22b= 0.92806321492917698

TTb= 0.87826123592324523 TSb= 0.68245421631518099

FATb= 0.22294849368164138 T11/T22= 0.470706314

Alleles sampled from the same population

T11w= 0.39543232488890911 T12w= 1.0743523981065564 T22w= 0.84005939196807011

TTw= 0.84604912826752299 TSw= 0.61774585842848961

FATw= 0.26984635077461283 T11/T22= 0.470719486

Time (units of 2NT generations) = 0.250000000

Alleles sampled from different populations

T11b= 0.50497119107722477 T12b= 1.0856636144945628 T22b= 0.91095435373449207

TTb= 0.89681319345021071 TSb= 0.70796277240585836

FATb= 0.21057944109609816 T11/T22= 0.554332018

Alleles sampled from the same population

T11w= 0.45709722223529203 T12w= 1.0859464900253426 T22w= 0.82457378847364327

TTw= 0.86339099768990513 TSw= 0.64083550535446765

FATw= 0.25776906746874650 T11/T22= 0.554343641

Time (units of 2NT generations) = 0.300000012

Alleles sampled from different populations

T11b= 0.56239012723222181 T12b= 1.0984159909317368 T22b= 0.89792817610523312

TTb= 0.91428757130023208 TSb= 0.73015915166872747

FATb= 0.20139004992668863 T11/T22= 0.626319706

Alleles sampled from the same population

T11w= 0.50907067312427889 T12w= 1.0986977640703923 T22w= 0.81278368731709261

TTw= 0.87981247214553904 TSw= 0.66092718022068575

FATw= 0.24878630259818046 T11/T22= 0.626329839

Time (units of 2NT generations) = 0.349999994

Alleles sampled from different populations

T11b= 0.61104608495399448 T12b= 1.1119334478700065 T22b= 0.88836246798968777

TTb= 0.93081886217092380 TSb= 0.74970427647184112

FATb= 0.19457554316924131 T11/T22= 0.687834203

Alleles sampled from the same population

T11w= 0.55311232904952379 T12w= 1.1122142504056374 T22w= 0.80412587240089284

TTw= 0.89541667556542281 TSw= 0.67861910072520826

FATw= 0.24211920634973083 T11/T22= 0.687842965

Time (units of 2NT generations) = 0.400000006

Alleles sampled from different populations

T11b= 0.65251462993885012 T12b= 1.1259168773039494 T22b= 0.88171181505184348

TTb= 0.94651504989964808 TSb= 0.76711322249534675

FATb= 0.18953932895554271 T11/T22= 0.740054309

Alleles sampled from the same population

T11w= 0.59064830334210172 T12w= 1.1261968154147053 T22w= 0.79810664724372138

TTw= 0.91028714535380839 TSw= 0.69437747529291149

FATw= 0.23718853019392860 T11/T22= 0.740061879

Time (units of 2NT generations) = 0.449999988

Alleles sampled from different populations

T11b= 0.68807420165026389 T12b= 1.1401389400021165 T22b= 0.87750170351178070

TTb= 0.96146344629156943 TSb= 0.78278795258102229

FATb= 0.18583701169265543 T11/T22= 0.784128606

Alleles sampled from the same population

T11w= 0.62283576809323782 T12w= 1.1404180998797744 T22w= 0.79429649439420680

TTw= 0.92449211556174826 TSw= 0.70856613124372236

FATw= 0.23356173696172955 T11/T22= 0.784135103

Time (units of 2NT generations) = 0.500000000

Alleles sampled from different populations

T11b= 0.71876341032787061 T12b= 1.1544277525496232 T22b= 0.87532195812940516

TTb= 0.97573521838913047 TSb= 0.79704268422863789

FATb= 0.18313629639760387 T11/T22= 0.821141779

Alleles sampled from the same population

T11w= 0.65061481593503112 T12w= 1.1547062047627463 T22w= 0.79232413507233579

TTw= 0.93808784013321489 TSw= 0.72146947550368346

FATw= 0.23091479855316122 T11/T22= 0.821147263

Time (units of 2NT generations) = 0.600000024

Alleles sampled from different populations

T11b= 0.76875332383894435 T12b= 1.1827224140244095 T22b= 0.87569433569142585

TTb= 1.0024731218947973 TSb= 0.82222382976518515

FATb= 0.17980461340341858 T11/T22= 0.877878606

Alleles sampled from the same population

T11w= 0.69586456935521290 T12w= 1.1829996162915020 T22w= 0.79266246503242943

TTw= 0.96363156674266159 TSw= 0.74426351719382122

FATw= 0.22764722236151358 T11/T22= 0.877882600

Time (units of 2NT generations) = 0.699999988

Alleles sampled from different populations

T11b= 0.80753543104290171 T12b= 1.2101210473723238 T22b= 0.88058401367622074

TTb= 1.0270903848659425 TSb= 0.84405972235956117

FATb= 0.17820307268310254 T11/T22= 0.917045295

Alleles sampled from the same population

T11w= 0.73096951171590707 T12w= 1.2103971658875943 T22w= 0.79708959940371260

TTw= 0.98721336072370203 TSw= 0.76402955555980978

FATw= 0.22607453874032013 T11/T22= 0.917048097

Time (units of 2NT generations) = 0.800000012

Alleles sampled from different populations

T11b= 0.83847786792073331 T12b= 1.2362680216252184 T22b= 0.88837542669918101

TTb= 1.0498473344675878 TSb= 0.86342664730995722

FATb= 0.17756932940366099 T11/T22= 0.943832815

Alleles sampled from the same population

T11w= 0.75897830278552592 T12w= 1.2365431790192147 T22w= 0.80414318953493036

TTw= 1.0090519625897214 TSw= 0.78156074616022808

FATw= 0.22545044741366882 T11/T22= 0.943834782

Time (units of 2NT generations) = 0.899999976

Alleles sampled from different populations

T11b= 0.86384043843932390 T12b= 1.2609987433024936 T22b= 0.89792414386242070

TTb= 1.0709405172266828 TSb= 0.88088229115087224

FATb= 0.17746851764277916 T11/T22= 0.962041676

Alleles sampled from the same population

T11w= 0.78193640174228007 T12w= 1.2612730344839846 T22w= 0.81278734381480300

TTw= 1.0293174536312630 TSw= 0.79736187277854154

FATw= 0.22534892421615926 T11/T22= 0.962043047

Time (units of 2NT generations) = 1.00000000

Alleles sampled from different populations

T11b= 0.88515005108665057 T12b= 1.2842599630063116 T22b= 0.90843173066793981

TTb= 1.0905254269418034 TSb= 0.89679089087729524

FATb= 0.17765247033973741 T11/T22= 0.974371552

Alleles sampled from the same population

T11w= 0.80122589080209916 T12w= 1.2845334647810698 T22w= 0.82229935506301965

TTw= 1.0481480438568147 TSw= 0.81176262293255941

FATw= 0.22552674911689041 T11/T22= 0.974372506

Time (units of 2NT generations) = 1.25000000

Alleles sampled from different populations

T11b= 0.92674766776547424 T12b= 1.3361353942417780 T22b= 0.93572573947112736

TTb= 1.1336860489300393 TSb= 0.93123670361830080

FATb= 0.17857619885400200 T11/T22= 0.990405202

Alleles sampled from the same population

T11w= 0.83888039826428540 T12w= 1.3364071868424996 T22w= 0.84700691991666344

TTw= 1.0896754229664869 TSw= 0.84294365909047442

FATw= 0.22642684112698463 T11/T22= 0.990405619

Time (units of 2NT generations) = 1.50000000

Alleles sampled from different populations

T11b= 0.95796400749372845 T12b= 1.3798195638300950 T22b= 0.96142620493831177

TTb= 1.1697573350230575 TSb= 0.95969510621602017

FATb= 0.17957761196932065 T11/T22= 0.996398866

Alleles sampled from the same population

T11w= 0.86713790153481707 T12w= 1.3800899443882957 T22w= 0.87027171728285768

TTw= 1.1243973768985664 TSw= 0.86870480940883743

FATw= 0.22740409462267486 T11/T22= 0.996399045

Time (units of 2NT generations) = 1.75000000

Alleles sampled from different populations

T11b= 0.98274925612165132 T12b= 1.4164665901435221 T22b= 0.98408437701823948

TTb= 1.1999417033567337 TSb= 0.98341681656994540

FATb= 0.18044617182741340 T11/T22= 0.998643279

Alleles sampled from the same population

T11w= 0.88957397201349098 T12w= 1.4167357936949077 T22w= 0.89078245967435843

TTw= 1.1534570047694161 TSw= 0.89017821584392465

FATw= 0.22825193122661969 T11/T22= 0.998643339

Time (units of 2NT generations) = 2.00000000

Alleles sampled from different populations

T11b= 1.0029922339922224 T12b= 1.4471714074902371 T22b= 1.0035070942325852

TTb= 1.2252105358013203 TSb= 1.0032496641124038

FATb= 0.18116141283730325 T11/T22= 0.999486923

Alleles sampled from the same population

T11w= 0.90789832886414601 T12w= 1.4474396269928675 T22w= 0.90836435576039198

TTw= 1.1777854846525684 TSw= 0.90813134231226900

FATw= 0.22895013213704518 T11/T22= 0.999486983

Time (units of 2NT generations) = 2.50000000

Alleles sampled from different populations

T11b= 1.0337053314692866 T12b= 1.4944203652427031 T22b= 1.0337818958684388

TTb= 1.2640819894557827 TSb= 1.0337436136688627

FATb= 0.18221790810110827 T11/T22= 0.999925911

Alleles sampled from the same population

T11w= 0.93570048154195795 T12w= 1.4946870717253369 T22w= 0.93576978397881871

TTw= 1.2152111022428627 TSw= 0.93573513276038833

FATw= 0.22998141554718976 T11/T22= 0.999925911

Time (units of 2NT generations) = 3.00000000

Alleles sampled from different populations

T11b= 1.0551143400098140 T12b= 1.5275494587424150 T22b= 1.0551257258321360

TTb= 1.2913347458316951 TSb= 1.0551200329209749

FATb= 0.18292291264770710 T11/T22= 0.999989212

Alleles sampled from the same population

T11w= 0.95508038263319794 T12w= 1.5278151045904258 T22w= 0.95509068853569734

TTw= 1.2414503200874367 TSw= 0.95508553558444764

FATw= 0.23066954824484642 T11/T22= 0.999989212

Time (units of 2NT generations) = 4.00000000

Alleles sampled from different populations

T11b= 1.0806145996447023 T12b= 1.5670620536113411 T22b= 1.0806148514352649

TTb= 1.3238383895756625 TSb= 1.0806147255399836

FATb= 0.18372609976482157 T11/T22= 0.999999762

Alleles sampled from the same population

T11w= 0.97816377573664282 T12w= 1.5673264344882496 T22w= 0.97816400364543288

TTw= 1.2727451620896437 TSw= 0.97816388969103785

FATw= 0.23145346073439443 T11/T22= 0.999999762

Time (units of 2NT generations) = 5.00000000

Alleles sampled from different populations

T11b= 1.0931469251010963 T12b= 1.5864849506193919 T22b= 1.0931469306692914

TTb= 1.3398159392522930 TSb= 1.0931469278851940

FATb= 0.18410664042760749 T11/T22= 1.00000000

Alleles sampled from the same population

T11w= 0.98950831119773486 T12w= 1.5867487096855573 T22w= 0.98950831623779922

TTw= 1.2881285117016621 TSw= 0.98950831371776704

FATw= 0.23182484920654967 T11/T22= 1.00000000

**Scaled recombination rate= 0.4**

Equilibrium results for subdivided population

Alleles sampled from different populations

T11b= 1.1052631578947369 T12b= 6.1052631578947372 T22b= 1.1052631578947369

TTb= 3.6052631578947367 TSb= 1.1052631578947369

FATb= 0.69343065693430650 T11/T22= 1.00000000

Alleles sampled from the same population

T11w= 1.0004761904761905 T12w= 6.1055263157894739 T22w= 1.0004761904761905

TTw= 3.5530012531328321 TSw= 1.0004761904761905

FATw= 0.71841378057662431 T11/T22= 1.00000000

Time (units of 2NT generations) = 2.49999994E-03

Alleles sampled from different populations

T11b= 2.9621816027880499E-003 T12b= 1.0542973071743118 T22b= 1.0503715762159567

TTb= 0.79048209304184214 TSb= 0.52666687890937236

FATb= 0.33373964629266484 T11/T22= 2.82012741E-03

Alleles sampled from the same population

T11w= 2.7032803389637206E-003 T12w= 1.0545617100166762 T22w= 0.95076866561592654

TTw= 0.76564884149706069 TSw= 0.47673597297744513

FATw= 0.37734383291785423 T11/T22= 2.84325774E-03

Time (units of 2NT generations) = 4.99999989E-03

Alleles sampled from different populations

T11b= 5.9710119171541010E-003 T12b= 1.0565331667619544 T22b= 1.0481270834587249

TTb= 0.79179110722494694 TSb= 0.52704904768793948

FATb= 0.33435846540998670 T11/T22= 5.69683965E-03

Alleles sampled from the same population

T11w= 5.4267406509178606E-003 T12w= 1.0567991954804992 T22w= 0.94873663594111701

TTw= 0.76694044188825827 TSw= 0.47708168829601744

FATw= 0.37794167286131031 T11/T22= 5.71996532E-03

Time (units of 2NT generations) = 7.49999983E-03

Alleles sampled from different populations

T11b= 8.9658667585945651E-003 T12b= 1.0587681075783868 T22b= 1.0458949615360937

TTb= 0.79309926086286553 TSb= 0.52743041414734415

FATb= 0.33497553184765616 T11/T22= 8.57243501E-03

Alleles sampled from the same population

T11w= 8.1375502508707065E-003 T12w= 1.0590341344436141 T22w= 0.94671623584813303

TTw= 0.76823051374655793 TSw= 0.47742689304950187

FATw= 0.37853693063927585 T11/T22= 8.59555416E-03

Time (units of 2NT generations) = 9.99999978E-03

Alleles sampled from different populations

T11b= 1.1946815755699003E-002 T12b= 1.0610021215489480 T22b= 1.0436751498866816

TTb= 0.79440655218506917 TSb= 0.52781098282119032

FATb= 0.33559084908173231 T11/T22= 1.14468718E-02

Alleles sampled from the same population

T11w= 1.0835772940586130E-002 T12w= 1.0612681465240961 T22w= 0.94470697842536988

TTw= 0.76951976110353715 TSw= 0.47777137568297801

FATw= 0.37913046573641540 T11/T22= 1.14699826E-02

Time (units of 2NT generations) = 1.49999997E-02

Alleles sampled from different populations

T11b= 1.7867273000917727E-002 T12b= 1.0654673692161127 T22b= 1.0392722088989867

TTb= 0.79701855508303243 TSb= 0.52856974094995224

FATb= 0.33681626659885422 T11/T22= 1.71921011E-02

Alleles sampled from the same population

T11w= 1.6194708121018975E-002 T12w= 1.0657333904229063 T22w= 0.94072166664316870

TTw= 0.77209578890250008 TSw= 0.47845818738209384

FATw= 0.38031239872166522 T11/T22= 1.72151960E-02

Time (units of 2NT generations) = 1.99999996E-02

Alleles sampled from different populations

T11b= 2.3732933756757113E-002 T12b= 1.0699289103084082 T22b= 1.0349177665893932

TTb= 0.79962713024074161 TSb= 0.52932535017307514

FATb= 0.33803477876780819 T11/T22= 2.29321923E-02

Alleles sampled from the same population

T11w= 2.1504044120062016E-002 T12w= 1.0701949277624880 T22w= 0.93678025353107741

TTw= 0.77466853829402882 TSw= 0.47914214882556971

FATw= 0.38148753287343495 T11/T22= 2.29552705E-02

Time (units of 2NT generations) = 2.99999993E-02

Alleles sampled from different populations

T11b= 3.5302038752325071E-002 T12b= 1.0788408750863061 T22b= 1.0263524270427806

TTb= 0.80483405399192942 TSb= 0.53082723289755285

FATb= 0.34045132625206265 T11/T22= 3.43956314E-02

Alleles sampled from the same population

T11w= 3.1975885426361872E-002 T12w= 1.0791068850813925 T22w= 0.92902735739986897

TTw= 0.77980425324725389 TSw= 0.48050162141311542

FATw= 0.38381764473300206 T11/T22= 3.44186686E-02

Time (units of 2NT generations) = 3.99999991E-02

Alleles sampled from different populations

T11b= 4.6658422486818285E-002 T12b= 1.0877380207871363 T22b= 1.0179752779614442

TTb= 0.81002743550563383 TSb= 0.53231685022413122

FATb= 0.34284096205723036 T11/T22= 4.58345339E-02

Alleles sampled from the same population

T11w= 4.2255181541540621E-002 T12w= 1.0880040233841495 T22w= 0.92144480222188252

TTw= 0.78492700763293055 TSw= 0.48184999188171157

FATw= 0.38612127344833613 T11/T22= 4.58575301E-02

Time (units of 2NT generations) = 5.00000007E-02

Alleles sampled from different populations

T11b= 5.7806291258628573E-002 T12b= 1.0966203527391256 T22b= 1.0097825427957192

TTb= 0.81520738488314981 TSb= 0.53379441702717390

FATb= 0.34520414445989478 T11/T22= 5.72462752E-02

Alleles sampled from the same population

T11w= 5.2345739806005653E-002 T12w= 1.0968863479979554 T22w= 0.91402916964478020

TTw= 0.79003690136167415 TSw= 0.48318745472539293

FATw= 0.38839887871997947 T11/T22= 5.72692230E-02

Time (units of 2NT generations) = 5.99999987E-02

Alleles sampled from different populations

T11b= 6.8749767622480196E-002 T12b= 1.1054878766846494 T22b= 1.0017705202045666

TTb= 0.82037401029908641 TSb= 0.53526014391352339

FATb= 0.34754131994213000 T11/T22= 6.86282590E-02

Alleles sampled from the same population

T11w= 6.2251291759396477E-002 T12w= 1.1057538646641110 T22w= 0.90677710939148048

TTw= 0.79513403261977467 TSw= 0.48451420057543848

FATw= 0.39065090827633031 T11/T22= 6.86511472E-02

Time (units of 2NT generations) = 7.00000003E-02

Alleles sampled from different populations

T11b= 7.9492892057628772E-002 T12b= 1.1143405987710748 T22b= 0.99393558255884140

TTb= 0.82552741803965501 TSb= 0.53671423730823509

FATb= 0.34985292362215215 T11/T22= 7.99779147E-02

Alleles sampled from the same population

T11w= 7.1975494650564631E-002 T12w= 1.1146065795289797 T22w= 0.89968533790539018

TTw= 0.80021849790347854 TSw= 0.48583041627797741

FATw= 0.39287779831280811 T11/T22= 8.00007433E-02

Time (units of 2NT generations) = 7.99999982E-02

Alleles sampled from different populations

T11b= 9.0039624602931134E-002 T12b= 1.1231785255415412 T22b= 0.98627417447436794

TTb= 0.83066771254009542 TSb= 0.53815689953864954

FATb= 0.35213937966479791 T11/T22= 9.12926942E-02

Alleles sampled from the same population

T11w= 8.1521932917550943E-002 T12w= 1.1234444991346884 T22w= 0.89275063702261281

TTw= 0.80529039205238506 TSw= 0.48713628497008188

FATw= 0.39507997391033933 T11/T22= 9.13154557E-02

Time (units of 2NT generations) = 9.00000036E-02

Alleles sampled from different populations

T11b= 0.10039384645937455 T12b= 1.1320016639262915 T22b= 0.97878281137418077

TTb= 0.83579499642153454 TSb= 0.53958832891677766

FATb= 0.35440110167322014 T11/T22= 0.102570094

Alleles sampled from the same population

T11w= 9.0894119638121884E-002 T12w= 1.1322676304104853 T22w= 0.88596985267055839

TTw= 0.81034980828241276 TSw= 0.48843198615434014

FATw= 0.39725784943467513 T11/T22= 0.102592789

Time (units of 2NT generations) = 0.100000001

Alleles sampled from different populations

T11b= 0.11055936156062929 T12b= 1.1408100212338796 T22b= 0.97145807807938778

TTb= 0.84090937052694414 TSb= 0.54100871982000853

FATb= 0.35663849306258422 T11/T22= 0.113807648

Alleles sampled from the same population

T11w= 0.10009549795137906 T12w= 1.1410759806639739 T22w= 0.87933989359245990

TTw= 0.81539683821794673 TSw= 0.48971769577191948

FATw= 0.39941182891731641 T11/T22= 0.113830268

Time (units of 2NT generations) = 0.125000000

Alleles sampled from different populations

T11b= 0.13517184132375570 T12b= 1.1627662985655425 T22b= 0.95385344821836093

TTb= 0.85363947166830045 TSb= 0.54451264477105832

FATb= 0.36212808469728286 T11/T22= 0.141711324

Alleles sampled from the same population

T11w= 0.12237363772741205 T12w= 1.1630322405941786 T22w= 0.86340512232151689

TTw= 0.82796081030932145 TSw= 0.49288938002446447

FATw= 0.40469479486556403 T11/T22= 0.141733736

Time (units of 2NT generations) = 0.150000006

Alleles sampled from different populations

T11b= 0.15868425609594894 T12b= 1.1846303728283010 T22b= 0.93721901356169379

TTb= 0.86629100382856117 TSb= 0.54795163482882137

FATb= 0.36747394073451456 T11/T22= 0.169313952

Alleles sampled from the same population

T11w= 0.14365605080575494 T12w= 1.1848962977781188 T22w= 0.84834852499153834

TTw= 0.84044929283838277 TSw= 0.49600228789864664

FATw= 0.40983674788572022 T11/T22= 0.169336125

Time (units of 2NT generations) = 0.174999997

Alleles sampled from different populations

T11b= 0.18115010183534341 T12b= 1.2064023870425684 T22b= 0.92150674778634711

TTb= 0.87886540592670681 TSb= 0.55132842481084521

FATb= 0.37268161757999230 T11/T22= 0.196580335

Alleles sampled from the same population

T11w= 0.16399115917301255 T12w= 1.2066682952226273 T22w= 0.83412663046962487

TTw= 0.85286359502197306 TSw= 0.49905889482131871

FATw= 0.41484324370949255 T11/T22= 0.196602240

Time (units of 2NT generations) = 0.200000003

Alleles sampled from different populations

T11b= 0.20262025187205546 T12b= 1.2280824968144950 T22b= 0.90667098043305150

TTb= 0.89136405648352413 TSb= 0.55464561615255348

FATb= 0.37775635878716241 T11/T22= 0.223477155

Alleles sampled from the same population

T11w= 0.18342501093823516 T12w= 1.2283483885208870 T22w= 0.82069810003828425

TTw= 0.86520497200457336 TSw= 0.50206155548825970

FATw= 0.41971952111527411 T11/T22= 0.223498762

Time (units of 2NT generations) = 0.224999994

Alleles sampled from different populations

T11b= 0.22314308557721096 T12b= 1.2496708696241638 T22b= 0.89266828143812860

TTb= 0.90378827656591687 TSb= 0.55790568350766978

FATb= 0.38270311977544280 T11/T22= 0.249973133

Alleles sampled from the same population

T11w= 0.20200139679798390 T12w= 1.2499367451407419 T22w= 0.80802362287915286

TTw= 0.87747462748965521 TSw= 0.50501250983856838

FATw= 0.42447052710419009 T11/T22= 0.249994427

Time (units of 2NT generations)= 0.224999994

Alleles sampled from different populations

T11b= 0.22314308557721096 T12b= 1.2496708696241638 T22b= 0.89266828143812860

TTb= 0.90378827656591687 TSb= 0.55790568350766978

FATb= 0.38270311977544280 T11/T22= 0.249973133

Alleles sampled from the same population

T11w= 0.20200139679798390 T12w= 1.2499367451407419 T22w= 0.80802362287915286

TTw= 0.87747462748965521 TSw= 0.50501250983856838

FATw= 0.42447052710419009 T11/T22= 0.249994427

Time (units of 2NT generations) = 0.250000000

Alleles sampled from different populations

T11b= 0.24276461071905397 T12b= 1.2711676841503001 T22b= 0.87945735132426739

TTb= 0.91613933258598046 TSb= 0.56111098102166068

FATb= 0.38752659004627998 T11/T22= 0.276039094

Alleles sampled from the same population

T11w= 0.21976196078722365 T12w= 1.2714335437491666 T22w= 0.79606581667892162

TTw= 0.88967371624111968 TSw= 0.50791388873307264

FATw= 0.42910093952307160 T11/T22= 0.276060045

Time (units of 2NT generations) = 0.275000006

Alleles sampled from different populations

T11b= 0.26152857981564637 T12b= 1.2925731296286687 T22b= 0.86699891677302010

TTb= 0.92841843896150089 TSb= 0.56426374829433323

FATb= 0.39223121319574328 T11/T22= 0.301648110

Alleles sampled from the same population

T11w= 0.23674630559622600 T12w= 1.2928389735708503 T22w= 0.78478913310655229

TTw= 0.90180334646111970 TSw= 0.51076771935138909

FATw= 0.43361518743996996 T11/T22= 0.301668674

Time (units of 2NT generations) = 0.300000012

Alleles sampled from different populations

T11b= 0.27947660077867975 T12b= 1.3138874052429790 T22b= 0.85525563131517446

TTb= 0.94062676064495310 TSb= 0.56736611604692710

FATb= 0.39682120498261708 T11/T22= 0.326775521

Alleles sampled from the same population

T11w= 0.25299209272006573 T12w= 1.3141532337788888 T22w= 0.77415976792293772

TTw= 0.91386458205019527 TSw= 0.51357593032150173

FATw= 0.43801746953653919 T11/T22= 0.326795727

Time (units of 2NT generations) = 0.324999988

Alleles sampled from different populations

T11b= 0.29664824212881524 T12b= 1.3351107195454466 T22b= 0.84419198088820713

TTb= 0.95276541552697880 TSb= 0.57042011150851124

FATb= 0.40130056967589511 T11/T22= 0.351399034

Alleles sampled from the same population

T11w= 0.26853513769450554 T12w= 1.3353765329153902 T22w= 0.76414557549601769

TTw= 0.92585844475532586 TSw= 0.51634035659526156

FATw= 0.44231177074621442 T11/T22= 0.351418823

Time (units of 2NT generations) = 0.349999994

Alleles sampled from different populations

T11b= 0.31308113304842955 T12b= 1.3562432899073036 T22b= 0.83377419402229491

TTb= 0.96483547672133296 TSb= 0.57342766353536223

FATb= 0.40567311487761393 T11/T22= 0.375498712

Alleles sampled from the same population

T11w= 0.28340950065898762 T12w= 1.3565090883420385 T22w= 0.75471598750545033

TTw= 0.93778591621212881 TSw= 0.51906274408221897

FATw= 0.44650187733806179 T11/T22= 0.375518084

Time (units of 2NT generations) = 0.375000000

Alleles sampled from different populations

T11b= 0.32881105852547998 T12b= 1.3772853419964877 T22b= 0.82397015642809390

TTb= 0.97683797473663736 TSb= 0.57639060747678694

FATb= 0.40994246499048514 T11/T22= 0.399057001

Alleles sampled from the same population

T11w= 0.29764757247627505 T12w= 1.3775511257176500 T22w= 0.74584193563153489

TTw= 0.94964793988577745 TSw= 0.52174475405390497

FATw= 0.45059139061928588 T11/T22= 0.399075955

Time (units of 2NT generations)= 0.400000006

Alleles sampled from different populations

T11b= 0.34387204982905151 T12b= 1.3982371092817720 T22b= 0.81474932977045122

TTb= 0.98877389954076167 TSb= 0.57931068979975131

FATb= 0.41411207347927215 T11/T22= 0.422058702

Alleles sampled from the same population

T11w= 0.31128015662662323 T12w= 1.3985028785023204 T22w= 0.73749577803306710

TTw= 0.96144542291608270 TSw= 0.52438796732984516

FATw= 0.45458373940835228 T11/T22= 0.422077209

Time (units of 2NT generations) = 0.425000012

Alleles sampled from different populations

T11b= 0.35829647054596014 T12b= 1.4190988325621374 T22b= 0.80608267442301607

TTb= 1.0006442025233127 TSb= 0.58218957248448810

FATb= 0.41818523405583374 T11/T22= 0.444490969

Alleles sampled from the same population

T11w= 0.32433654708398985 T12w= 1.4193645874867347 T22w= 0.72965122942849403

TTw= 0.97317923787148830 TSw= 0.52699388825624194

FATw= 0.45848219141124613 T11/T22= 0.444509000

Time (units of 2NT generations) = 0.449999988

Alleles sampled from different populations

T11b= 0.37211509839579326 T12b= 1.4398707595186151 T22b= 0.79794257600862295

TTb= 1.0124497983604117 TSb= 0.58502883720220811

FATb= 0.42216509090167287 T11/T22= 0.466343194

Alleles sampled from the same population

T11w= 0.33684460237109914 T12w= 1.4401365003441260 T22w= 0.72228329460377205

TTw= 0.98485022441578074 TSw= 0.52956394848743560

FATw= 0.46228986361700208 T11/T22= 0.466360778

Time (units of 2NT generations) = 0.474999994

Alleles sampled from different populations

T11b= 0.38535720303192855 T12b= 1.4605531442905813 T22b= 0.79030277553976858

TTb= 1.0241915667882149 TSb= 0.58782998928584851

FATb= 0.42605464802914006 T11/T22= 0.487607062

Alleles sampled from the same population

T11w= 0.34883081598120813 T12w= 1.4608188712063654 T22w= 0.71536820517887056

TTw= 0.99645919089320245 TSw= 0.53209951058003935

FATw= 0.46600973181543148 T11/T22= 0.487624168

Time (units of 2NT generations) = 0.500000000

Alleles sampled from different populations

T11b= 0.39805062002520208 T12b= 1.4811462470719468 T22b= 0.78313830298301979

TTb= 1.0358703542880290 TSb= 0.59059446150411099

FATb= 0.42985677786865861 T11/T22= 0.508276284

Alleles sampled from the same population

T11w= 0.36032038334458338 T12w= 1.4814119602602140 T22w= 0.70888335947343895

TTw= 1.0080069158346125 TSw= 0.53460187140901116

FATw= 0.46964463932633838 T11/T22= 0.508292913

Time (units of 2NT generations) = 0.574999988

Alleles sampled from different populations

T11b= 0.43309704305239283 T12b= 1.5423925483248953 T22b= 0.76426518151192346

TTb= 1.0705368303035268 TSb= 0.59868111228215815

FATb= 0.44076551564095601 T11/T22= 0.566684246

Alleles sampled from the same population

T11w= 0.39204300689146154 T12w= 1.5426582213578435 T22w= 0.69180058691551860

TTw= 1.0422900091306668 TSw= 0.54192179690349007

FATw= 0.48006620791128396 T11/T22= 0.566699445

Time (units of 2NT generations) = 0.600000024

Alleles sampled from different populations

T11b= 0.44384777460014124 T12b= 1.5626312331730370 T22b= 0.75877595203161463

TTb= 1.0819715482444574 TSb= 0.60131186331587794

FATb= 0.44424429247559161 T11/T22= 0.584952354

Alleles sampled from the same population

T11w= 0.40177414659168131 T12w= 1.5628968931427991 T22w= 0.68683208962740872

TTw= 1.0536000056261721 TSw= 0.54430311810954501

FATw= 0.48338732421887509 T11/T22= 0.584967077

Time (units of 2NT generations) = 0.625000000

Alleles sampled from different populations

T11b= 0.45416983018533030 T12b= 1.5827820150741951 T22b= 0.75365442855777964

TTb= 1.0933470722228751 TSb= 0.60391212937155503

FATb= 0.44764828597039530 T11/T22= 0.602623463

Alleles sampled from the same population

T11w= 0.41111726889177969 T12w= 1.5830476621322500 T22w= 0.68219642185676710

TTw= 1.0648522537532616 TSw= 0.54665684537427339

FATw= 0.48663596902999096 T11/T22= 0.602637708

Time (units of 2NT generations) = 0.649999976

Alleles sampled from different populations

T11b= 0.46408380325292709 T12b= 1.6028451831530672 T22b= 0.74888215122489898

TTb= 1.1046640801959902 TSb= 0.60648297723891309

FATb= 0.45097972486684779 T11/T22= 0.619702041

Alleles sampled from the same population

T11w= 0.42009101399575588 T12w= 1.6031108174455291 T22w= 0.67787687461640100

TTw= 1.0760473808758038 TSw= 0.54898394430607844

FATw= 0.48981433897524485 T11/T22= 0.619715810

Time (units of 2NT generations) = 0.675000012

Alleles sampled from different populations

T11b= 0.47360927879784076 T12b= 1.6228210302823838 T22b= 0.74444156682987606

TTb= 1.1159232265481211 TSb= 0.60902542281385841

FATb= 0.45424075032674671 T11/T22= 0.636194050

Alleles sampled from the same population

T11w= 0.42871310930679751 T12w= 1.6230866519503842 T22w= 0.67385755958664184

TTw= 1.0871859931985519 TSw= 0.55128533444671968

FATw= 0.49292454290657983 T11/T22= 0.636207342

Time (units of 2NT generations) = 0.699999988

Alleles sampled from different populations

T11b= 0.48276488283533481 T12b= 1.6427098528169966 T22b= 0.74031598438839397

TTb= 1.1271251432144305 TSb= 0.61154043361186439

FATb= 0.45743342050925961 T11/T22= 0.652106524

Alleles sampled from the same population

T11w= 0.43700041420554725 T12w= 1.6429754619967820 T22w= 0.67012336888710000

TTw= 1.0982686767715528 TSw= 0.55356189154632363

FATw= 0.49596860654028452 T11/T22= 0.652119339

Time (units of 2NT generations) = 0.725000024

Alleles sampled from different populations

T11b= 0.49156832944429851 T12b= 1.6625119503419912 T22b= 0.73648953286943108

TTb= 1.1382704407494280 TSb= 0.61402893115686474

FATb= 0.46055971483139535 T11/T22= 0.667447805

Alleles sampled from the same population

T11w= 0.44496896263141905 T12w= 1.6627775471652875 T22w= 0.66665993681995439

TTw= 1.1092959984454871 TSw= 0.55581444972568672

FATw= 0.49894847677754384 T11/T22= 0.667460203

Time (units of 2NT generations) = 0.750000000

Alleles sampled from different populations

T11b= 0.50003646550219494 T12b= 1.6822276254321595 T22b= 0.73294712100122517

TTb= 1.1493597093419348 TSb= 0.61649179325171000

FATb= 0.46362153793899563 T11/T22= 0.682227194

Alleles sampled from the same population

T11w= 0.45263400357451800 T12w= 1.6824932100263315 T22w= 0.66345360348822813

TTw= 1.1202685067788523 TSw= 0.55804380353137306

FATw= 0.50186602572990635 T11/T22= 0.682239115

Time (units of 2NT generations) = 0.774999976

Alleles sampled from different populations

T11b= 0.50818531322540217 T12b= 1.7018571834245284 T22b= 0.72967439904712450

TTb= 1.1603935197803958 TSb= 0.61892985613626328

FATb= 0.46662072341338512 T11/T22= 0.696454883

Alleles sampled from the same population

T11w= 0.46001003958116127 T12w= 1.7021227559127663 T22w= 0.66049138019712461

TTw= 1.1311867329009546 TSw= 0.56025070988914294

FATw= 0.50472305447539423 T11/T22= 0.696466386

Time (units of 2NT generations) = 0.800000012

Alleles sampled from different populations

T11b= 0.51603011062218429 T12b= 1.7214009322032515 T22b= 0.72665772245483662

TTb= 1.1713724243708810 TSb= 0.62134391653851040

FATb= 0.46955903723598336 T11/T22= 0.710141897

Alleles sampled from the same population

T11w= 0.46711086336993057 T12w= 1.7216664927047933 T22w= 0.65776091655105229

TTw= 1.1420511913326423 TSw= 0.56243588996049143

FATw= 0.50752129656798184 T11/T22= 0.710152984

Time (units of 2NT generations) = 0.824999988

Alleles sampled from different populations

T11b= 0.52358534996084560 T12b= 1.7408591819943782 T22b= 0.72388411728719748

TTb= 1.1822969578092000 TSb= 0.62373473362402154

FATb= 0.47243818103042068 T11/T22= 0.723299980

Alleles sampled from the same population

T11w= 0.47394959265124381 T12w= 1.7411247306246693 T22w= 0.65525046916320884

TTw= 1.1528623807659479 TSw= 0.56460003090722632

FATw= 0.51026242132030286 T11/T22= 0.723310590

Time (units of 2NT generations) = 0.850000024

Alleles sampled from different populations

T11b= 0.53086481435073662 T12b= 1.7602322451714931 T22b= 0.72134124734722926

TTb= 1.1931676380102381 TSb= 0.62610303084898300

FATb= 0.47525979510046801 T11/T22= 0.735941291

Alleles sampled from the same population

T11w= 0.48053870323870762 T12w= 1.7604977820424086 T22w= 0.65294887189875905

TTw= 1.1636207848055709 TSw= 0.56674378756873334

FATw= 0.51294803687832857 T11/T22= 0.735951483

Time (units of 2NT generations) = 0.875000000

Alleles sampled from different populations

T11b= 0.53788161252819044 T12b= 1.7795204360713663 T22b= 0.71901738291439765

TTb= 1.2039849668963303 TSb= 0.62844949772129399

FATb= 0.47802546128019308 T11/T22= 0.748078704

Alleles sampled from the same population

T11w= 0.48689006053570405 T12w= 1.7797859612913438 T22w= 0.65084550757638460

TTw= 1.1743268726736942 TSw= 0.56886778405604432

FATw= 0.51557969310465279 T11/T22= 0.748088539

Time (units of 2NT generations) = 0.899999976

Alleles sampled from different populations

T11b= 0.54464821193590640 T12b= 1.7987240708198700 T22b= 0.71690137101311291

TTb= 1.2147494311471898 TSb= 0.63077479147450966

FATb= 0.48073670561111848 T11/T22= 0.759725451

Alleles sampled from the same population

T11w= 0.49301494947724844 T12w= 1.7989895844941053 T22w= 0.64893028105674322

TTw= 1.1849810998805506 TSw= 0.57097261526699583

FATw= 0.51815888428553714 T11/T22= 0.759734869

Time (units of 2NT generations) = 0.925000012

Alleles sampled from different populations

T11b= 0.55117647017926208 T12b= 1.8178434671649386 T22b= 0.71498260713837425

TTb= 1.2254615029118785 TSb= 0.63307953865881816

FATb= 0.48339500085924592 T11/T22= 0.770894945

Alleles sampled from the same population

T11w= 0.49892410300275580 T12w= 1.8181089693954835 T22w= 0.64719359364988560

TTw= 1.1955839088609019 TSw= 0.57305884832632070

FATw= 0.52068705167477103 T11/T22= 0.770903945

Time (units of 2NT generations)= 0.949999988

Alleles sampled from different populations

T11b= 0.55747766493927320 T12b= 1.8368789443202500 T22b= 0.71325100836717015

TTb= 1.2361216404867359 TSb= 0.63536433665322167

FATb= 0.48600176888494540 T11/T22= 0.781600952

Alleles sampled from the same population

T11w= 0.50462772913182075 T12w= 1.8371444352061985 T22w= 0.64562631877696774

TTw= 1.2061357295802964 TSw= 0.57512702395439419

FATw= 0.52316558588764850 T11/T22= 0.781609595

Time (units of 2NT generations) = 0.975000024

Alleles sampled from different populations

T11b= 0.56356252241808513 T12b= 1.8558308228158422 T22b= 0.71169698778770174

TTb= 1.2467302889593679 TSb= 0.63762975510289344

FATb= 0.48855838287596587 T11/T22= 0.791857421

Alleles sampled from the same population

T11w= 0.51013553671170719 T12w= 1.8560963024534463 T22w= 0.64421977882480985

TTw= 1.2166369801108523 TSw= 0.57717765776825858

FATw= 0.52559582915548908 T11/T22= 0.791865706

Time (units of 2NT generations) = 1.00000000

Alleles sampled from different populations

T11b= 0.56944124438851995 T12b= 1.8746994243565025 T22b= 0.71031143018188425

TTb= 1.2572878808208521 TSb= 0.63987633728520210

FATb= 0.49106616945401338 T11/T22= 0.801678300

Alleles sampled from the same population

T11w= 0.51545675990130768 T12w= 1.8749648928393405 T22w= 0.64296572313485334

TTw= 1.2270880671787106 TSw= 0.57921124151808057

FATw= 0.52797907745139416 T11/T22= 0.801686227

Time (units of 2NT generations) = 1.10000002

Alleles sampled from different populations

T11b= 0.59108186323924672 T12b= 1.9493475288757507 T22b= 0.70628677066600876

TTb= 1.2990159229141891 TSb= 0.64868431695262774

FATb= 0.50063405266243310 T11/T22= 0.836886525

Alleles sampled from the same population

T11w= 0.53504515014438980 T12w= 1.9496129536253672 T22w= 0.63932312935391966

TTw= 1.2683985466872609 TSw= 0.58718413974915473

FATw= 0.53706653063996912 T11/T22= 0.836893141

Time (units of 2NT generations) = 1.20000005

Alleles sampled from different populations

T11b= 0.61012054667459736 T12b= 2.0226891586316205 T22b= 0.70433615816444650

TTb= 1.3399587555255712 TSb= 0.65722835241952193

FATb= 0.50951598345149240 T11/T22= 0.866234899

Alleles sampled from the same population

T11w= 0.55227838685966313 T12w= 2.0229545409590024 T22w= 0.63755785798286491

TTw= 1.3089363316901332 TSw= 0.59491812242126407

FATw= 0.54549498855067302 T11/T22= 0.866240442

Time (units of 2NT generations) = 1.29999995

Alleles sampled from different populations

T11b= 0.62700785857583208 T12b= 2.0947452113210359 T22b= 0.70405823469844153

TTb= 1.3801391289790863 TSb= 0.66553304663713675

FATb= 0.51777829302655631 T11/T22= 0.890562475

Alleles sampled from the same population

T11w= 0.56756429904434191 T12w= 2.0950105524149389 T22w= 0.63730661904496833

TTw= 1.3487230057297972 TSw= 0.60243545904465512

FATw= 0.55332899603156405 T11/T22= 0.890567064

Time (units of 2NT generations) = 1.39999998

Alleles sampled from different populations

T11b= 0.64211265334709167 T12b= 2.1655366156955109 T22b= 0.70512514529782033

TTb= 1.4195777575089836 TSb= 0.67361889932245600

FATb= 0.52547939289743839 T11/T22= 0.910636425

Alleles sampled from the same population

T11w= 0.58123675618011572 T12w= 2.1658019166428124 T22w= 0.63827265397601463

TTw= 1.3877783108604387 TSw= 0.60975470507806517

FATw= 0.56062528120935240 T11/T22= 0.910640240

Time (units of 2NT generations) = 1.50000000

Alleles sampled from different populations

T11b= 0.65573696248980184 T12b= 2.2350842574056591 T22b= 0.70726914625905091

TTb= 1.4582936558900428 TSb= 0.68150305437442638

FATb= 0.53267090505273884 T11/T22= 0.927139223

Alleles sampled from the same population

T11w= 0.59356914284887374 T12w= 2.2353495192080466 T22w= 0.64021361405881372

TTw= 1.4261204488309451 TSw= 0.61689137845384368

FATw= 0.56743388753766399 T11/T22= 0.927142322

Time (units of 2NT generations) = 1.75000000

Alleles sampled from different populations

T11b= 0.68483609602318307 T12b= 2.4036479124793582 T22b= 0.71600404875747103

TTb= 1.5520339924348425 TSb= 0.70042007239032711

FATb= 0.54870829131035792 T11/T22= 0.956469595

Alleles sampled from the same population

T11w= 0.61990904674405545 T12w= 2.4039130803593958 T22w= 0.64812078875943802

TTw= 1.5189639990555712 TSw= 0.63401491775174668

FATw= 0.58260043151387997 T11/T22= 0.956471503

Time (units of 2NT generations) = 2.00000000

Alleles sampled from different populations

T11b= 0.70890607369286873 T12b= 2.5648874729559763 T22b= 0.72775723051550512

TTb= 1.6416095625300815 TSb= 0.71833165210418692

FATb= 0.56242235151391307 T11/T22= 0.974096894

Alleles sampled from the same population

T11w= 0.64169676603543613 T12w= 2.5651525519064524 T22w= 0.65875993272801958

TTw= 1.6076904506440903 TSw= 0.65022834938172780

FATw= 0.59555127722427770 T11/T22= 0.974098027

Time (units of 2NT generations) = 2.25000000

Alleles sampled from different populations

T11b= 0.72965300416491685 T12b= 2.7191141523152780 T22b= 0.74105465497534284

TTb= 1.7272339909427039 TSb= 0.73535382957012985

FATb= 0.57425928772465717 T11/T22= 0.984614313

Alleles sampled from the same population

T11w= 0.66047659019163485 T12w= 2.7193791467524910 T22w= 0.67079681988254314

TTw= 1.6925079258947902 TSw= 0.66563670503708905

FATw= 0.60671575308270287 T11/T22= 0.984614968

Time (units of 2NT generations) = 2.50000000

Alleles sampled from different populations

T11b= 0.74812080185909069 T12b= 2.8666287442561851 T22b= 0.75501680524105397

TTb= 1.8090987739031286 TSb= 0.75156880355007227

FATb= 0.58456176390603409 T11/T22= 0.990866423

Alleles sampled from the same population

T11w= 0.67719342203370392 T12w= 2.8668936581881113 T22w= 0.68343535489364338

TTw= 1.7736040233258925 TSw= 0.68031438846367365

FATw= 0.61642261772278995 T11/T22= 0.990866840

Time (units of 2NT generations) = 2.75000000

Alleles sampled from different populations

T11b= 0.76495244072221968 T12b= 3.0077208258930783 T22b= 0.76912331592569838

TTb= 1.8873793521085187 TSb= 0.76703787832395909

FATb= 0.59359633903642672 T11/T22= 0.994577110

Alleles sampled from the same population

T11w= 0.69242925216788320 T12w= 3.0079856630231996 T22w= 0.69620452917186149

TTw= 1.8511512768465361 TSw= 0.69431689066987234

FATw= 0.62492698497734256 T11/T22= 0.994577348

Time (units of 2NT generations) = 3.00000000

Alleles sampled from different populations

T11b= 0.78054794798199767 T12b= 3.1426684940543366 T22b= 0.78307059755150854

TTb= 1.9622388834105446 TSb= 0.78180927276675316

FATb= 0.60157283632668634 T11/T22= 0.996778488

Alleles sampled from the same population

T11w= 0.70654616738344833 T12w= 3.1429332578466287 T22w= 0.70882954927435948

TTw= 1.9253105580877663 TSw= 0.70768785832890391

FATw= 0.63242924350252094 T11/T22= 0.996778667

Time (units of 2NT generations) = 3.50000000

Alleles sampled from different populations

T11b= 0.80895152019584915 T12b= 3.3951860396040185 T22b= 0.80987433889212879

TTb= 2.1022994845740040 TSb= 0.80941292954398891

FATb= 0.61498685820778620 T11/T22= 0.998860538

Alleles sampled from the same population

T11w= 0.73225686002192536 T12w= 3.3954506663483599 T22w= 0.73309215142551509

TTw= 2.0640625860360400 TSw= 0.73267450572372028

FATw= 0.64503280536139362 T11/T22= 0.998860598

Time (units of 2NT generations) = 4.00000000

Alleles sampled from different populations

T11b= 0.83447352891560633 T12b= 3.6261822097339476 T22b= 0.83481110824355564

TTb= 2.2304122641567643 TSb= 0.83464231857958104

FATb= 0.62579011423472064 T11/T22= 0.999595642

Alleles sampled from the same population

T11w= 0.75535920222605635 T12w= 3.6264467112182954 T22w= 0.75566476290856333

TTw= 2.1909793468928029 TSw= 0.75551198256730978

FATw= 0.65517156351165085 T11/T22= 0.999595642

Time (units of 2NT generations) = 4.50000000

Alleles sampled from different populations

T11b= 0.85765174344444661 T12b= 3.8374899198441454 T22b= 0.85777523444341486

TTb= 2.3476017043940378 TSb= 0.85771348894393074

FATb= 0.63464267071431379 T11/T22= 0.999856055

Alleles sampled from the same population

T11w= 0.77633996532589455 T12w= 3.8377543067837729 T22w= 0.77645174347936607

TTw= 2.3070750805932017 TSw= 0.77639585440263037

FATw= 0.66347178688133490 T11/T22= 0.999856055

Time (units of 2NT generations) = 5.00000000

Alleles sampled from different populations

T11b= 0.87879276045688737 T12b= 4.0307868057639258 T22b= 0.87883793510344121

TTb= 2.4548010767720450 TSb= 0.87881534778016435

FATb= 0.64200140040032583 T11/T22= 0.999948621

Alleles sampled from the same population

T11w= 0.79547667353201401 T12w= 4.0310510879361585 T22w= 0.79551756346421720

TTw= 2.4132741032171370 TSw= 0.79549711849811566

FATw= 0.67036603200704059 T11/T22= 0.999948621
